# Entner-Doudoroff pathway in *Synechocystis* PCC 6803: Proposed regulatory roles and enzyme multifunctionalities

**DOI:** 10.3389/fmicb.2022.967545

**Published:** 2022-08-16

**Authors:** Anushree Bachhar, Jiri Jablonsky

**Affiliations:** Institute of Complex Systems, FFPW, University of South Bohemia, CENAKVA, Nove Hrady, Czechia

**Keywords:** Entner-Doudoroff pathway, kinetic model, metabolic regulation, glycolysis, cyanobacteria

## Abstract

The Entner-Doudoroff pathway (ED-P) was established in 2016 as the fourth glycolytic pathway in *Synechocystis sp*. PCC 6803. ED-P consists of two reactions, the first catalyzed by 6-phosphogluconate dehydratase (EDD), the second by keto3-deoxygluconate-6-phosphate aldolase/4-hydroxy-2-oxoglutarate aldolase (EDA). ED-P was previously concluded to be a widespread (∼92%) pathway among cyanobacteria, but current bioinformatic analysis estimated the occurrence of ED-P to be either scarce (∼1%) or uncommon (∼47%), depending if dihydroxy-acid dehydratase (ilvD) also functions as EDD (currently assumed). Thus, the biochemical characterization of ilvD is a task pending to resolve this uncertainty. Next, we have provided new insights into several single and double glycolytic mutants based on kinetic model of central carbon metabolism of *Synechocystis*. The model predicted that silencing 6-phosphogluconate dehydrogenase (*gnd*) could be coupled with ∼90% down-regulation of G6P-dehydrogenase, also limiting the metabolic flux *via* ED-P. Furthermore, our metabolic flux estimation implied that growth impairment linked to silenced EDA under mixotrophic conditions is not caused by diminished carbon flux *via* ED-P but rather by a missing mechanism related to the role of EDA in metabolism. We proposed two possible, mutually non-exclusive explanations: (i) Δ*eda* leads to disrupted carbon catabolite repression, regulated by 2-keto3-deoxygluconate-6-phosphate (ED-P intermediate), and (ii) EDA catalyzes the interconversion between glyoxylate and 4-hydroxy-2-oxoglutarate + pyruvate in the proximity of TCA cycle, possibly effecting the levels of 2-oxoglutarate under Δ*eda*. We have also proposed a new pathway from EDA toward proline, which could explain the proline accumulation under Δ*eda*. In addition, the presented *in silico* method provides an alternative to ^13^C metabolic flux analysis for marginal metabolic pathways around/below the threshold of ultrasensitive LC-MS. Finally, our *in silico* analysis provided alternative explanations for the role of ED-P in *Synechocystis* while identifying some severe uncertainties.

## Introduction

*Synechocystis* sp. PCC 6803 (*Synechocystis*) is a model cyanobacterial organism with complex carbon metabolism, reported to contain all known glycolytic routes found so far in cyanobacteria: (i) Embden-Meyerhof-Parnas pathway (EMP-P), (ii) oxidative pentose phosphate pathway (OPP-P), (iii) lately characterized (Xiong et al., 2015) phosphoketolase pathway (PKET-P) and (iv) recently identified and quantified (Chen et al., 2016; Makowka et al., 2020) Entner-Doudoroff pathway (ED-P). These four glycolytic pathways, doing a similar job, could be seen as contra-productive, like having a substantial number of isozymes. Nevertheless, the cellular resources are not being wasted, as all these “redundancies” allow enhanced robustness of metabolism, including adaptability to the changing environment (Xiong et al., 2017) and reduction of total protein cost (Jablonsky et al., 2016). This metabolic plasticity found in *Synechocystis* is clearly an evolutionary advantage under turbulent environmental conditions but makes the understanding of metabolic regulation rather challenging.

**FIGURE 1:**
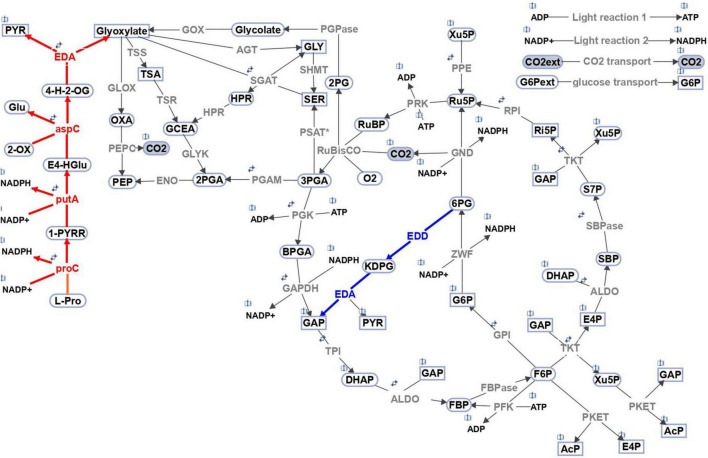
Schematic representation of the central carbon metabolism network implemented in the multi-level kinetic model of *Synechocystis sp*. PCC 6803. The blue highlights the reactions of the Entner-Doudoroff pathway. Red indicates the proposed secondary role or EDA and related pathway to proline; annotated gene IDs are included for the convenience. The model includes the Calvin-Benson cycle, glycogen synthesis (sink from glucose-6-phosphate), photorespiratory pathways, phosphoketolase pathway, glycolysis, the oxidative pentose pathway, Entner–Doudoroff pathway, and sink reactions (representing the adjacent pathway and the calculation of biomass production, indicated by metabolites in rectangular shapes). The reversibility of a particular reaction is indicated by two small arrows. Gray indicates the involved enzymes: *RuBisCO*, ribulose-1,5-bisphosphate carboxylase oxygenase; *PGK*, phosphoglycerate kinase; *GAP*, glyceraldehyde-3-phosphate dehydrogenase; *TPI*, triose-phosphate isomerase; *ALDO*, aldolase; *FBPase*, fructose-1,6 bisphosphatase; *PFK*, phosphofructokinase; *TKT*, transketolase; *SBPase*, sedoheptulose-1,7 bisphosphatase; *RPI*, phosphopentose isomerase; *PPE*, phosphopentose epimerase; *PRK*, phosphoribulokinase; *GPI*, glucose-6-phosphate isomerase; *G6PD*, glucose-6-phosphate dehydrogenase; *PGD*, phosphogluconate dehydrogenase; *PGPase*, phosphoglycolate phosphatase; *PKET*, phosphoketolase; *GOX*, glycolate oxidase; *SGAT*, serineglyoxylate transaminase; *HPR*, hydroxypyruvate reductase; *GLYK*, glycerate kinase; *AGT*, alanineglyoxylate transaminase; *TSS*, tartronatesemialdehyde synthase; *TSR*, tartronatesemialdehyde reductase; *SHMT*, serine hydroxymethyltransferase; *GLOX*, glyoxylate oxidase; PSAT*, phosphoserine transaminase; *PPC*, phosphoenolpyruvate carboxylase; *PGM*, phosphoglycerate mutase; *ENO*, enolase; *GND*, 6-phosphogluconate dehydrogenase; *ZWF*, glucose-6-phosphate dehydrogenase; *EDD*, 6P-gluconate dehydratase; *EDA*, 2-keto-3-deoxygluconate-6- phosphate aldolase; *aspC*, L-erythro-4-hydroxyglutamate:2-oxoglutarate aminotransferase (activity of aspartate aminotransferase); *putA*, delta-1-pyrroline-5-carboxylate dehydrogenase; *proC*, pyrroline-5-carboxylate reductase. Names of metabolites in the suggested (red) pathway: 4-H-2-OG 4-hydroxy-2-oxoglutarate, E4-HGlu L-erythro-4-hydroxyglutamate, 1-PYRR L-1-pyrroline-5-carboxylate. The open book symbol indicates the involvement of the metabolite in other reaction(s). The scheme was created in SimBiology toolbox of MATLAB 2021b (The MathWorks, Inc., Natick, Massachusetts, United States of America), http://www.mathworks.com.

Entner-Doudoroff pathway (ED-P) is the shortest glycolytic route, consisting of only two reactions. The first one is catalyzed by 6-phosphogluconate dehydratase (EDD, *slr0452*, currently annotated as ilvD – dihydroxy-acid dehydratase), producing 2-keto3-deoxygluconate-6-phosphate (KDPG). The second reaction is catalyzed by keto3-deoxygluconate-6-phosphate aldolase (EDA, *sll0107*), producing pyruvate and glyceraldehyde 3-phosphate (Figure 1, blue). It should be noted that while only phosphorylated ED-P has been reported in *Synechocystis* (Chen et al., 2016), there are multiple branches of ED-P (namely phosphorylated, semi-phosphorylated, and non-phosphorylated) that can co-exist in a single organism as shown for *Sulfolobus solfataricus* (Figueiredo et al., 2017). More details about the different branches of ED-P and the enzymes involved can be found in a recently published comprehensive review (Kopp et al., 2020).

The protein cost of ED-P is 72% less while providing one less ATP (Flamholz et al., 2013) in comparison to EMP-P. Further, ED-P provides less NADPH than OPP-P but is more carbon-efficient (Asplund-Samuelsson and Hudson, 2021). Significant growth impairment was reported for EDA knockout (Δ*eda*), both under autotrophic and mixotrophic conditions, up to 18% and 57% (Makowka et al., 2020), respectively. However, the exact factors behind observed growth impairment are somewhat unclear. The current lack of information on ED-P is partially due to unknown metabolic flux and the neglected possible signaling role of KDPG, identified in other organisms (Fuhrman et al., 1998; Kim et al., 2009). Therefore, we aim to address the current issues related to ED-P with the help of *in silico* analysis.

## Materials and methods

### Bioinformatic analysis: Standard and alternative calculation

To quantify the occurrence of each known glycolytic pathway, we chose four enzymes specific to those pathways based on their position and essentiality within the metabolic network: (i) 6-phosphogluconate dehydrogenase (GND) for OPP pathway, (ii) phosphoketolase (PKET) for PKET pathway, (iii) keto3-deoxygluconate-6-phosphate aldolase (EDA) for ED pathway and (iv) phosphofructokinase (PFK) for upper EMP pathway. The raw data for the occurrence of the marker enzymes were collected from UniProt (Supplementary file 1)^1^ and later on verified using NCBI^2^ and KEGG^3^ databases. The collected data were then curated by discarding repetitive and discontinued amino acid sequences. In the case of isoenzymes, only one of them was counted. Finally, the percentage of occurrence of each enzyme was calculated against the list of total species of cyanobacteria found to date (Supplementary file 1). Along with the standard approach, an alternative approach was also employed to remove the influence of the heterogeneity in the size of different genera and marker occurrence within a particular genus. The alternative approach for the occurrence of enzyme markers was calculated as the sum of the first positive hits from each genus, divided by the sum of the first positive and first negative hits for each genus. An example of alternative calculation: based on our database (Supplementary file 1), the genus *Anabena* has 11 species, 10 of them with PFK. Thus, the particular result for *Anabena* will increase the numerator by one and the denominator by two in the alternative calculation for PFK among cyanobacteria.

### Sources of experimental data for kinetic parameters estimation

^13^C labeling data for autotrophic growth (Hing et al., 2019).

^13^C labeling data for mixotrophic growth (Nakajima et al., 2014).

### General information about the model

The multi-level kinetic model for *Synechocystis* was developed and simulations were executed using the SimBiology, Optimization, Global optimization and Parallel computing toolboxes of MATLAB (MathWorks, Inc., Natick, Massachusetts, United States of America). The routine for parameter estimation was a hybrid genetic algorithm. The model versions for tested growth conditions (Supplementary files 5, 6) are available in the SBML format L2V4, compatible with MATLAB 2010b–2014.

The scope of the model includes the following parts of central carbon metabolism: Calvin-Benson cycle, photorespiration, all glycolytic pathways (Embden–Meyerhof–Parnas pathway, Entner–Doudoroff pathway, phosphoketolase pathway and oxidative pentose phosphate) with simplified carbohydrate and biomass synthesis (weighted sum of sink reactions). These metabolic reactions were coupled with simplified light reactions, Ci and glucose uptake as the primary input parameters. This model is an updated version of the previous model employed for the analysis of PKET pathway (Bachhar and Jablonsky, 2020). The current model consists of 60 reactions, 49 metabolites and 199 kinetic parameters. The enzymatic reactions were described by Michaelis-Menten kinetics with an exception for the light reactions, Ci and glucose uptake (mass action kinetics). The list of all parameters within the model can be found in Supplementary file 2.

### General information about the model

The constraint of model parameters occurred in several steps. Firstly, the original model of *Synechocystis* without ED-P (Jablonsky et al., 2016) was fitted on available fluxomic data from cells grown autotrophically at high CO_2_. Then, we applied transcriptomic data as weight factors for each estimated V_max_ in simulated shifts to autotrophic ambient CO_2_, followed by mixotrophic ambient CO_2_. Thus, we were searching for a single set of kinetic parameters describing all growth conditions. However, we are currently able to describe only autotrophic growth conditions (ambient and high CO_2_) with a single set of parameters; mixotrophic growth conditions are verified separately to respective fluxomic data. The justification and broader description of this part of our Methodology can be found in our previous work (Jablonsky et al., 2016).

Next, we have implemented a simplified, single reaction version of ED-P within the highly constrained model and fitted the flux *via* ED-P to match the reported levels of growth impairment. Additional constraining of parameter space was achieved by matching reported growth changes for single and double mutants from all glycolytic pathways, including silenced *eda* (keto3-deoxygluconate-6-phosphate aldolase), which represented blocked ED-P. After a confirmation that the metabolic fluxes available *via* ED-P cannot explain the reported level of growth impairment under mixotrophic conditions, we have incorporated a full version of ED-P (EDA and EDD) and tested the impact of accumulation and possible excretion of KDPG (intermediate of ED-P) as well as other scenarios under autotrophic conditions. Since all these scenarios are based on carbon availability which plays a marginal role under mixotrophic conditions, we proposed that the missing link, allowing us to explain the experimental data, is the signaling role of KDPG (carbon catabolite repression). We did not perform any simulations based on the signaling role of KDPG as the growth impairment caused by Δ*eda* provides a sufficient description only of its impact, but new experimental data are needed to determine the kinetics of the signaling process. The current version of the model assumes a very low level of KDPG for WT, based on reported undetectable levels (Will et al., 2019; Schulze et al., 2022).

## Results and discussion

### Occurrence of glycolytic pathways in cyanobacteria: Standard vs. alternative view

Cyanobacteria possess four glycolytic pathways, which play an essential role in metabolic adaptation and can even alternate with each other (Xiong et al., 2017). Previously, ED-P was concluded to be a widespread pathway in cyanobacteria, almost twice as common as upper EMP-P (Chen et al., 2016). However, our current bioinformatic analyses of all glycolytic pathways showed a very different result (Table 1). This discrepancy was probably caused by the influx of new species into the databases. Current data implies that the occurrence of EDD/ilvD (see the explanation below) in cyanobacteria is approximately 92% (Table 1) which is comparable to PKET and GND occurrences. Nevertheless, the occurrence of ED-P (based on EDA) drops to 46.9% (Table 1), which is only half of the previous value (Chen et al., 2016). We note that the occurrence of EDA calculated in November 2015 (Chen et al., 2016) matches the occurrence for EDD/ilvD in 2021 (Table 1). Thus, ED-P goes down from one of the most common (Chen et al., 2016) to the rarest glycolytic pathway among cyanobacteria. Finally, going back to EDD, the previous (Chen et al., 2016) and our calculation of its occurrence is based on an assumption of dual functionality of EDD and ilvD (dihydroxy-acid dehydratase) in a single enzyme EDD/ilvD (Chen et al., 2016) but the corresponding gene is annotated to express ilvD only. However, there is less than 50% similarity of ilvD (*Synechocystis*) toward EDD in *E.coli* (k 12) or *Pseudomonas* while ilvD from *E. coli* and *Pseudomonas* showed between 55 and 65% similarity with ilvD from *Synechocystis*, respectively. Currently, there are only four cyanobacteria annotated with a native (single functioning) EDD (Supplementary file 4). This fact may imply two origins of EDD among cyanobacteria, either associated with ilvD as assumed before (Chen et al., 2016) (4.2.1.9) or standalone enzyme (4.2.1.12), which would imply that ED-P is extremely rare (below 1% occurrence, Table 1) among cyanobacteria.

**TABLE 1:** Occurrence of marker enzymes among cyanobacteria [%].

Pathway	upper EMP	ED			PKET	OPP
Enzyme	PFK	EDA	EDD/ilvD	EDD	PKET	GND
Chen et al., 2016	52.0	NA	92.0	NA	NA	NA
2021 standard	64.1	46.9	91.9	0.7	81.6	89.3
2021 alternative	70.3	65.2	80.1	3.6	89.6	90

The key enzymes were selected based on their position and role within a particular glycolytic pathway: upper Embden-Meyerhof-Parnas pathway – PFK, phosphoketolase pathway – PKET, oxidative pentose phosphate pathway – GND and Entner-Doudoroff pathway – EDA. EDD is shown either as a native enzyme or as the dual function enzyme annotated as dihydroxy-acid dehydratase (ilvD), involved in the synthesis of valine and isoleucine (Chen et al., 2016). The percentages were calculated based on the total species of cyanobacteria identified in Uniprot and the number of cyanobacteria identified with the annotated enzyme.

In the case of *Synechocystis*, there is no mention of gluconate as a substrate for ilvD in recent biochemical analysis (Zhang et al., 2020). Nevertheless, a product of EDD, KDPG, has been detected in a single study (Chen et al., 2016) while determined to be undetectable in others (Will et al., 2019; Schulze et al., 2022). The situation is further complicated by an approximately 30-fold difference in 6-phosphogluconate (a substrate of EDD) level under autotrophic vs. mixotrophic conditions (Yoshikawa et al., 2013), which might be the reason for the elusiveness of KDPG in metabolic profiling. However, KDPG was previously detected not only for WT but also for silenced *zwf* (glucose-6-phosphate dehydrogenase) (Chen et al., 2016). At that time, it was suspected that ZWF was not the only source of 6-phosphogluconate (Chen et al., 2016), but no evidence was found to support it. The possible remaining explanations for detected KDPG are rather questionable: (i) silencing *zwf* has either low efficiency or gets repaired fast; however, no accumulation of 6-phosphogluconate was reported for Δ*zwf* (Makowka et al., 2020) or (ii) EDA conducts reversible reaction and thus can produce a significant amount of KDPG from pyruvate and glyceraldehyde 3-phosphate without a functional EDD. All these questions and uncertainties show that more experiments are needed to verify the status of ED-P not only in *Synechocystis* but generally in cyanobacteria. For the sake of further analysis, we will operate with EDA as a marker enzyme for ED-P occurrence.

Another point one should consider in any bioinformatic analysis is substantial heterogeneity in the number of species per genus as well as in the occurrence of marker enzymes within a particular genus, e.g., genus *Prochlorococcus* does not contain either PKET or PFK (Supplementary file 1). Therefore, we also present an alternative bioinformatic analysis of the occurrence of glycolytic marker enzymes, which shifts the focus from occurrence among species toward occurrence among the genera (see Methodology). This alternative calculation showed an approximate 18% and 11% change in the occurrence of EDA and EDD, respectively, in comparison to the standard calculation (Table 1). A lower level of correction could also be observed for PFK and PKET in contrast to minor change for GND (Table 1). Interestingly, the alternative calculation did not change the ranking in the occurrence of glycolytic enzymes but lowered the occurrence of EDD/ilvD among cyanobacteria. This finding contradicts the known essentiality of ilvD for amino acid synthesis (Leyval et al., 2003; Kim and Lee, 2006). The cumulated bioinformatic data (Supplementary file 1) indicate that difference between the standard and alternative analysis is caused by a higher amount of negative occurrence of EDD/ilvD, primarily due to the single species genera (only one species identified in the genus). This problem could be due to the lack of experimental characterization and annotation of the enzyme-specific genes for those species. Currently, we cannot say what the possible implications of this finding are, but ED-P remains to be one of the most overlooked pathways in cyanobacteria. Finally, ED-P is known to regulate organic carbon intake in other species such as *Pseudomonas* (Daddaoua et al., 2009), so one could speculate that ED-P is found mostly or only in mixotrophic cyanobacteria. However, this is not the case as EDA is missing in many mixotrophic species, e.g., genera *Microcystis* and *Moorea*, yet could be found in obligatory photoautotrophs, e.g., genus *Prochlorococcus*. The observed low occurrence of ED-P, as well as the missing link to any growth conditions, open a question regarding its evolutionary importance among cyanobacteria. The distribution of EDA, EDD/ilvD and EDD in cyanobacteria, as well as the other glycolytic markers, is provided in Supplementary file 1.

Furthermore, the alternative approach grouped the upper EMP and ED pathways on one side (around 70%, Table 1) and OPP and PKET pathways on the other side (around 90%, Table 1). To detect a possible correlation, we searched for the occurrence of combinations of any two glycolytic pathways among cyanobacteria. The highest positive correlation was found between OPP and PKET pathways (84.8%, Table 2), further supporting our recent finding of the higher importance of PKET pathway (Bachhar and Jablonsky, 2020). Surprisingly, the lowest correlation was found between upper EMP and ED-P which also corresponds with single marker presence of EDA vs other markers (around 44%) (Table 2). Also, the number of cyanobacterial species without either of these pathways is, on average, three times higher than other combinations of glycolytic pathways. Approximately 40% of cyanobacteria is “alternating” between upper EMP and ED-P (Table 2), e.g., *Calothrix desertica* PCC 7102 does not have any annotated *pfk* but has by two EDD/ilvD isozymes, DSM106972_077550 and DSM106972_030530 (Supplementary file 3). Having two EDD isozymes could increase the flux *via* ED-P, which may compensate for the incomplete upper EMP (Will et al., 2019) (Figure 1). On the other hand, genera *Microcystis*, *Moorea* and *Symploca*, known for their production of important secondary metabolites (Linington et al., 2008; Engene et al., 2012; Pimentel and Giani, 2014), do not have annotated ED-P (UniPathway) but contain more than one PFK isozyme (Uniprot). Finally, ED-P is the least preferred to supplement any other glycolytic pathways (Table 2). This result further questions the role and benefits of ED-P in cyanobacteria; hence we decided to run a comprehensive analysis based on mutants from all four glycolytic pathways for model cyanobacterium *Synechocystis*.

**TABLE 2:** Occurrence of marker enzyme couples among cyanobacteria [%].

Occurrence	PFK vs. EDA	GND vs. PKET	PFK vs. GND	PFK vs. PKET	PKET vs. EDA	EDA vs. GND
**++**	39.7	84.79	67.72	65.86	47.31	49.72
**–**	19.48	0	1.67	8.35	8.35	2.23
**+–**	40.82	15.21	30.61	25.79	44.34	48.05

“**+–**” denotes the presence of one of the markers within a particular couple, regardless of their order. “**++**” and “**–**” indicate both present and both absent, respectively (all data are available in Supplementary file 1). The percentages were calculated based on the total species of cyanobacteria found (UniProt, 2021) against the number of cyanobacteria with marker enzyme. ED pathway consists of only two enzymes, EDD and EDA, and thus the less occurring enzyme should be the marker. Due to extremely low occurrence of native EDD among cyanobacteria (around 100-fold difference vs. other markers) and the fact of ilvd functioning as EDD is the current opinion (Chen et al., 2016), we considered EDA (less common than ilvD) as the marker enzyme for ED-P in this analysis.

### Glycolytic mutants and their impact on growth

The growth impact for all single and some of the double mutants of glycolytic pathways have been studied in recent years (Xiong et al., 2015; Chen et al., 2016; Bachhar and Jablonsky, 2020; Makowka et al., 2020). Thus, our initial step was to mimic the reported growth impairments related to ED-P as well as for other glycolytic pathways and their combinations. The model was constrained not only by previously reported growth impairments found for different mutants but also by fluxomic data (WTs only). These constraints significantly limit the parameter space as the model was required to match all available data simultaneously. Finally, gathering data from all mutants of enzyme markers and their combinations provided a more accurate analysis rather than focusing only on the mutant of EDA.

**FIGURE 2:**
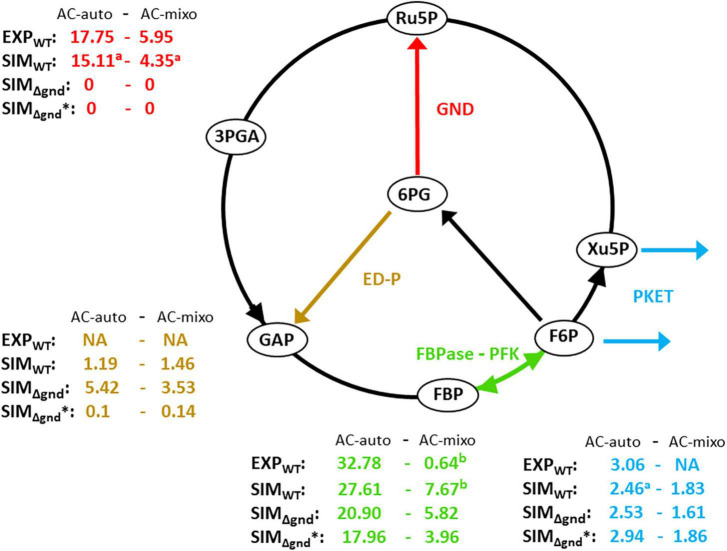
Experimental and simulated flux distribution *via* glycolytic pathways under autotrophic and mixotrophic conditions. Brown color shows the flux *via* Entner–Doudoroff pathway, blue color shows the flux *via* phosphoketolase (PKET) pathway, green color indicates the net flux *via* futile cycle of fructose-1,6 bisphosphatase (FBPas) and phosphofructokinase (PFK), red color shows the flux *via* 6-phosphogluconate dehydrogenase (GND). Index “**a**” denotes the cases of possible match between experiments and simulations if flux *via* ED-P is not considered in model; flux *via* ED-P was not considered in experimental-based calculations (Nakajima et al., 2014; Hing et al., 2019). Index “**b**” shows the case of simulated result being in the confidence interval of experimental value. All data were taken or simulated under ambient CO_2_ condition. (i) lower flux FBPase – PFK (green) under mixotrophic conditions is caused by redistribution of carbon flow, i.e., higher flux *via* EMP-P in expense of Calvin-Benson cycle (ii) flux values presented in this figure and Table 4 vary to some degree because they represent two independent and alternative calculations (two best fits). Units of metabolic fluxes: 10^– 2^ mmol h^– 1^ gDW^– 1^.

First, let us have a look at the results of single mutants under autotrophic (AC-auto) conditions. Simulated Δ*eda* and double mutant of PFK1,2 (Δ*pfk*) followed the reported mean growth impacts (around –2%) (Makowka et al., 2020), see Table 3. Whereas for Δ*pket* (double mutant of PKET1 and PKET2), no experimental data are available, but previously reported 11% growth impairment for Δ*pket1* (Xiong et al., 2015) is in line with the simulated 14% growth loss for Δ*pket*; this result is based on predicted supremacy of PKET1 (Bachhar and Jablonsky, 2020). Additionally, *in silico* silencing of GND (Δ*gnd*) leads to a massive 43% growth increase under AC-auto and a significant carbon flux redistribution within the central carbon metabolism (Figure 2, Δ*gnd*), which is in disagreement with previously reported statistically insignificant impact (around + 2%) in comparison to WT (Chen et al., 2016; Makowka et al., 2020). The predicted growth increase, triggered by Δ*gnd*, is mostly a consequence of reduced decarboxylation *via* OPP-P. Such discrepancy of the predicted vs. reported growth, not resolved by model parameters tuning, can be explained only by a conclusion that silencing *gnd* is coupled with other changes in metabolic regulation, so far not considered in the model. After testing various scenarios, the easiest and most reasonable way to negate this massive *in silico* growth increase is to couple silencing *gnd* with >90% down-regulation of G6P-dehydrogenase (*zwf*). We denoted this scenario as Δgnd* (asterisk differentiates from simple *in silico gnd* mutant discussed above) and the flux values and growth impact for Δgnd* are shown in Figure 2 and Table 3, respectively. Very significant down-regulation of *zwf* as a consequence of Δ*gnd* was reported previously for *Gluconobacter oxydans* (Richhardt et al., 2012), which supports our model prediction related to the real impact of silencing *gnd* in *Synechocystis*. An important consequence of *zwf* inhibition is a significant reduction in the production of 6-phosphogluconate, which is the key substrate for ED-P (Figure 1). When we accumulate all the effects of *in silico* Δgnd*, i.e., Δ*gnd*, down-regulated *zwf* and limited EDD, the result is in agreement with the previous experimental value for what is believed to be simply Δ*gnd* (Makowka et al., 2020) (Table 3).

**TABLE 3:** Simulated growth rate changes (%) caused by single and double mutants of marker glycolytic enzymes under autotrophic and mixotrophic conditions.

	Δ*pfk*	Δ*eda*	Δgnd*	Δ*pket*
**Δ*pfk***	–**1.8** –**0.2**	–11.2	3.2	–14.5
**Δ*eda***	**–5.4**	–**1.3** –**11.0**	0	–29.6
Δgnd*	**2.9**	**5.4**	**5.9** 1.7	–49.5
**Δ*pket***	**1.5**	**–1.0**	**6.1**	**0.5** –**14.0**

Roman font indicates autotrophic and bold shows mixotrophic results. Asterisk denotes Δgnd, including the assumed inhibition of zwf. Δpfk and Δpket indicate double mutants of isozymes. The results were rounded to the first decimal place. The available experimental values are shown and discussed in the text.

Secondly, the double mutants under AC-auto implied some significant growth impairment which demands a closer look. The highest simulated negative growth impact was found for double mutant Δgnd*Δ*pket*, i.e., 49.5% (Table 3). This prediction is not surprising based on the fact that GND and PKET were the only two tested glycolytic markers not missing together in any analyzed cyanobacteria (Table 2), emphasizing the essential role of PKET-P and OPP-P under AC-auto. Moreover, this double mutant is close to the triple mutant (Δ*gnd*Δ*pket*Δ*eda*) due to the above predicted and discussed the real impact of Δgnd*, which was greatly limiting the flux *via* ED-P (Figure 2). Another significant predicted growth impairment was found for Δ*eda*Δ*pket* (29.6%, Table 3), which is not a simple addition of respective single mutants as it surpasses the cumulative impact of both single mutants almost by 20%. This implies that blocking both glycolytic pathways protecting against decarboxylation *via* OPP-P and EMP-P might have an additional negative influence on the sustainability of the Calvin-Benson cycle, at least in the model.

Finally, the model failed to match the reported (∼60%) growth impairment (Makowka et al., 2020) for Δ*eda*Δ*gnd* under autotrophic conditions (Table 3). If we consider our prediction of what the meaning of Δgnd is, i.e., Δgnd*, one may expect an accumulation of 6-phosphogluconate due to some degree still functioning ZWF. Inhibition of sugar-phosphate metabolism by 6-phosphogluconate was already emphasized for other species (Richhardt et al., 2012) and its accumulation was confirmed for Δ*eda*Δ*gnd* in *Synechocystis* (Makowka et al., 2020). Other co-explanations of significant growth impairment could be i) partial accumulation of KDPG due to functioning EDD, although possibly inhibited by Δ*eda* and ii) other metabolic functions of EDA, which are further discussed in the next section. However, an experimental verification for the role of 6-phosphogluconate accumulation, level of KDPG and preferably also fluxomic data for these mutants are needed.

Next, we focused on growth data for AC-mixo. Due to the unavailability of experimental data for Δ*pket* and for most of the double mutants, it is difficult to discuss the results of simulations. However, the highest growth impairment caused by Δ*eda*Δ*pfk* (5.4%, Table 3) can be justified easily as both EDA and PFK supports the flux *via* the dominant glycolytic route under AC-mixo, EMP glycolysis (Nakajima et al., 2014). In the case of Δgnd*, the simulated 5.9% growth increase (Table 3) is in agreement with the recently reported value (Makowka et al., 2020). The only serious discrepancy between experiments and simulations for single mutant was found for Δ*eda*; the predicted growth impairment under AC-mixo is around 1.3% (Table 3) which is in contrast to previously reported up to 57% (Makowka et al., 2020) growth impairment. The possible explanation of growth impairment caused by Δ*eda* is elaborated in the next section.

Lastly, one can observe a relatively lower *in silico* flux *via* OPP-P (Figure 2) for WT compared to the experimental results (Figure 2). However, for Δ*eda*, the model matches the reported flux *via* OPP-P (as well as *via* PKET-P) (WT) (Figure 2 vs. Table 4). Since ED-P and OPP-P are closely connected (Figure 1) and ED-P was either not considered in the previous fluxomic analyses (Nakajima et al., 2014), or the flux *via* ED-P was not detected and assumed too low (Schulze et al., 2022). Thus, our simulation offers both an alternative solution and a possible revision of the available fluxomic data.

**TABLE 4:** Comparison of experimental and simulated metabolic fluxes.

	Mixotrophic			Autotrophic		
	^13^C exp	simulatio		^13^C exp	simulation	
				
Enzymatic reaction	fit	WT	Δ*eda*	fit	WT	Δ*eda*
ext.G6P → G6P	9.12	8.97	0.00%	0	0	0
KDPG → PYR + GAP	NA	1.50	–100%	NA	1.33	–100%
G6P → 6PG	6.36	**6.15**	< ± 5%	17.75	**16.15**	< ± 5%
6PG → Ru5P	6.36	**4.65**	22.63%	17.75	**14.82**	9.74%
3PGA ↔ 2PGA	19.62	17.96	5.57%	1.27	**1.11**	< ± 5%
2PGA → PEP	19.62	17.96	5.57%	1.27	**1.11**	< ± 5%
F6P ↔ G6P	–0.52	**–0.92**	< ± 5%	18.36	**49.26**	< ± 5%
3PGA ↔ BPGA	42.17	**41.08**	< ± 5%	83.76	**84.74**	< ± 5%
BPGA ↔ GAP	42.17	**41.08**	< ± 5%	83.76	**84.74**	< ± 5%
GAP ↔ DHAP	16.69	**17.07**	< ± 5%	39.78	**38.68**	< ± 5%
F6P + GAP ↔ E4P + Xu5P	8.95	**9.36**	< ± 5%	11.04	**10.52**	< ± 5%
DHAP + E4P ↔ SBP	16.01	**9.14**	< ± 5%	0.00	**11.12**	< ± 5%
SBP ↔ S7P	16.01	**9.14**	< ± 5%	6.98	**11.12**	< ± 5%
S7P + GAP ↔ Ri5P + Xu5P	8.26	**9.14**	< ± 5%	6.98	**11.12**	< ± 5%
Ri5P ↔ Ru5P	7.57	8.59	< ± 5%	6.76	**10.39**	–5.27%
Xu5P ↔ Ru5P	17.04	**17.06**	< ± 5%	11.04	**19.71**	–5.80%
Ru5P → RuBP	31.15	**30.31**	< ± 5%	42.77	**44.93**	< ± 5%
F6P/Xu5P → E4P/GAP + AceP	NA	1.46	< ± 5%	3.06	**2.55**	16.94%

Reaction catalyzed by EDA is in underlined. Simulated fluxes (WT and Δeda) correspond to day 5 for mixotrophic and day 7 for autotrophic growth experiments (^13^C exp), respectively, for each particular end of experiments (Makowka et al., 2020). The bold font highlight the simulated values within the experimental lower and upper bounds, for mixotrophic (Nakajima et al., 2014) and autotrophic (Hing et al., 2019) conditions. We note that flux values presented in Figure 2 and this table vary to some degree because they represent two independent and alternative calculations (two best fits). Units of metabolic fluxes: 10^–2^ mmol h^–1^ gDW^–1^.

### Two components of growth impairment caused by Δ*eda*

**FIGURE 3:**
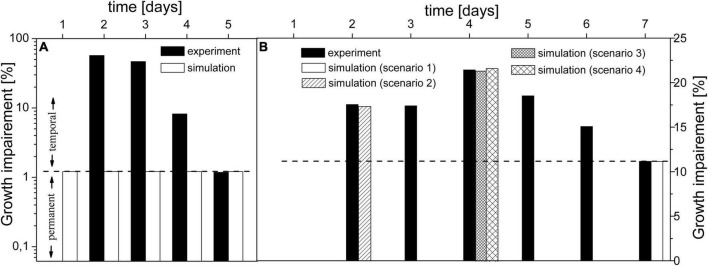
Comparison of experimental and simulated growth impairment caused by Δ*eda* under mixotrophic **(A)** and autotrophic **(B)** conditions. Original experimental growth data (Makowka et al., 2020) for WT and Δ*eda* were normalized to illustrate the growth impairment. Dashed line designates the possible division between the temporal and permanent components of growth impairment. Part **(A)** shows results for mixotrophic conditions (experimental data point for day 1 is not visible as it equals 0). Part **(B)** shows results for autotrophic conditions and elaboration of tested scenarios: **1** – flux *via* ED-P estimated to fit experimental growth impairment, assumed higher decarboxylation and KDPG accumulation from the previous days; **2** – initial usage of intracellular glycogen; **3** – 100% up-regulation of *gnd* and **4** – increased accumulation followed by excretion of KDPG.

Besides the reported mean growth impairment, there are also time-series data (Chen et al., 2016), (Makowka et al., 2020) for Δ*eda*, which can shed new light on the role of EDA. However, the model is able to mimic only the last day of the time-series experiment under both conditions (Figures 3A day 5, 3B day 7). When we take a closer look at gaps between experimental and simulated results under AC-auto, the maximal difference is ∼2-fold (Figure 3B, day 4). Such difference could be eliminated by tuning parameters within the model, followed by a justifiable explanation of the physiological meaning behind an observed change in metabolic fluxes triggered by Δ*eda*. On the other hand, the maximal difference under AC-mixo is ∼50-fold (Figure 3A, day 2) and clearly diminishes by the end of the experiment (Makowka et al., 2020). Since the simulated growth impairment (1.3%) under AC-mixo is the maximum possible value achievable by the model within given constraints (fluxomic and mutant growth data), an essential function of ED-P is missing in the model. By comparing experimental and simulated results, two classes of effects could be distinguished in the growth impairment, one class not reflected whereas the other one included in the model. Since the un-reflected class diminishes over time, we call it a “temporal component.” The second class of effects is based only on metabolic flux differences between WT and Δ*eda*, which could be stable unless the mutation is reversed; thus we call it a “permanent component.” Our model predicted that the permanent component is negligible under AC-mixo (Figure 3A) but is comparable with the impact of the temporal component under AC-auto (Figure 3B). Since the permanent component is related to metabolic flux *via* ED-P, its observed significant variation under AC-auto and AC-mixo could be explained either by a major change in flux *via* ED-P in one of the growth conditions or by carbon scarcity (auto- vs. mixotrophic).

The flux *via* ED-P has been so far unknown due to difficulties in measuring labeled metabolites produced by ED-P, i.e., pyruvate and glyceraldehyde-phosphate, as more prominent metabolic pathways (Calvin-Benson cycle and EMP glycolysis) produce them in higher quantities. By looking at metabolic fluxes estimated by our model, the flux *via* ED-P is slightly higher under mixotrophic conditions (Figure 2) and Δ*eda* leads to an expected increase of flux *via* GND (Table 4). Thus, ED-P is almost unaffected by the major flux redistribution trigged by autotrophic and mixotrophic conditions (Table 4). Hence, we could conclude that the key factor influencing the permanent component variability is carbon scarcity. A connection between ED-P and carbon scarcity is by Δ*eda* enhanced decarboxylation (Figure 1) under AC-auto, which was hypothesized previously (Makowka et al., 2020).

Now, the question remains is the origin behind the temporal component of growth impairment, which is not reflected in the model. If we start with AC-auto, we could assume that cultures used in growth experiments (Makowka et al., 2020) have started from a dormant state which explains zero growth impairment (Doello et al., 2018) on day 1 (Makowka et al., 2020) (Figures 3A,B). Thus, we hypothesize that both WT and Δ*eda* initially utilized stored glycogen to restore the metabolism (Doello et al., 2018). It has been determined that the rate of glycogen depletion is several-fold slower for Δ*eda* compared to WT under AC-auto (Makowka et al., 2020). So, we can speculate that glycogen lasted under Δ*eda* till the end of day 3 (Figure 3B). As glycogen is processed mainly *via* OPP-P and ED-P (Doello et al., 2018), silencing *eda* would increase the flux *via* OPP-P, further enhancing the decarboxylation for the first three days. Our simulations indicated that *in silico* addition of glycogen amplifies the growth impairment (Figure 3B, scenario 2), but it is clear now that decarboxylation level based on the current activity of *gnd* (*sll0329*) itself could not explain the sudden boost in growth impairment on day 4 (presumably the day of depleted glycogen). The required increase in GND activity (∼100% upregulation; Figure 3B, scenario 3) could be explained by posttranslational modification as predicted before for *Synechococcus* PCC *7942* (Jablonsky et al., 2016). The other potential explanation, besides the upregulation of *gnd*, is an accumulation of KDPG produced by EDD under Δ*eda*. The flux *via* ED-P under AC-auto (WT) is less than 2% of flux *via* glyceraldehyde-3-phosphate dehydrogenase (GAP in Figure 1 and Table 4). Hence, the slow accumulation of KDPG, although enhanced by glycogen, may take several days to activate at least a partial biochemical inhibition of EDD. Also, KDPG has been reported to be bacteriostatic at higher concentrations in *E.coli* (Fuhrman et al., 1998), thus *Synechocystis* may need a mechanism to prevent or limit its accumulation, possibly by excreting KDPG (Fuhrman et al., 1998). This prediction could explain the peak in growth impairment at day 4 (Figure 3B, scenario 3) caused by additional loss of carbon due to KDPG excretion, along with decarboxylation. Lastly, we can speculate that reaching the final level of reported growth impairment (day 7) takes up to 2 days due to continuous metabolic adaptation in response to changes in intracellular concentration of KDPG and enhanced decarboxylation via OPP-P.

In the case of mixotrophic conditions, none of the mechanisms within the model or from the tested scenarios could explain the temporal component – carbon loss due to decarboxylation and possible KDPG excretion under Δ*eda*; these mechanisms do not have a significant impact on growth rate due to abundance of carbon, in contrast to AC-auto. Therefore, we propose that the substantial difference between the growth rate of WT and Δ*eda* (Figure 3A) under AC-mixo should be seen as a simple growth delay since the temporal component diminishes by the fifth day of the experiment (Makowka et al., 2020) where both samples growth autotrophically due to depleted glucose. A possible explanation for delayed growth could be the suppressed carbon catabolite repression mechanism (Deutscher, 2008), responsible for rapid sensing and utilization of organic carbon, which is under normal circumstances (WT) controlled by the metabolic level of KDPG. Thus, too low (temporal product-inhibition of EDD) or too high (bacteriostatic) levels of KDPG caused by Δ*eda* is slowing down the utilization of organic carbon in *Synechocystis*, explaining the temporal component of growth impairment under AC-mixo. The role of KDPG in carbon catabolite repression was reported in other organisms (Kim et al., 2009; Campilongo et al., 2017). However, such role of KDPG in cyanobacteria needs to be experimentally verified.

There is another possibility or rather a speculation on how to explain the temporal component under mixotrophic conditions and that is the other functionality of EDA, so far not analyzed in *Synechocystis*. Based on the current annotation (although experimental verification is still needed), EDA should catalyze the interconversion of 4-hydroxy-2-oxoglutarate and pyruvate from glyoxylate (KEGG), see Figure 1 (red). On the other hand, either glyoxylate or 4-hydroxy-2-oxoglutarate (+ L-erythro-4-hydroxyglutamate) could be converted into 2-oxoglutarate. 2-oxoglutarate is known to have a signaling role in glucose metabolism in both prokaryotes (Daniel and Danchin, 1986) and eukaryotes (Dang et al., 2009) and is also a signaling molecule regulating the activity of TCA cycle in *Synechocystis* (Orthwein et al., 2021). Although the impact of Δ*eda* on the metabolism of 2-oxoglutarate is unknown, it regulates the glucose uptake (Doucette et al., 2011) and has a very significant impact on surrounding compounds such as proline in *Synechocystis* (Lucius et al., 2021). In order to explain the buildup of proline, we propose a pathway, fully annotated for *Synechocystis* (Uniprot), from proline to pyruvate and glyoxylate, producing two molecules of NAPDH (Figure 1, red) which supports previously speculated link among proline, NAPDH and Δ*eda* (Lucius et al., 2021). Furthermore, our assumptions related to Δ*eda* leading to bacteriostasis linked to an accumulation of KDPG might be alternatively explained by an accumulation of 2-oxoglutarate which was reported to reduce the growth rate in *E.coli* (Bren et al., 2016). We did not simulate any of these options as we currently do not have experimental data needed and thus, such simulations would not be reliable.

Finally, a new labeling experiment has been published recently. This study (Schulze et al., 2022) compared WT and Δ*eda*, however, the authors could not detect any flux *via* ED-P. The possible explanations are: i) a minor flux *via* ED-P, as suggested in our own flux estimation, ii) significant flux under certain conditions, such as fluctuating light (Schulze et al., 2022), which may explain previously reported significant level of KDPG (Chen et al., 2016), otherwise undetectable (Will et al., 2019; Schulze et al., 2022); we note that 10% fluctuations in light (i.e., ATP and NADPH regeneration in the model) did not influence our flux estimations (data not shown) for Δ*eda*, or iii) ED-P is missing in *Synechocystis*. Furthermore, Δ*eda* was reported to deactivate OPP shunt (Schulze et al., 2022), which disagrees with our model prediction purely based on the metabolic flux *via* ED-P. Thus, Δ*eda* mediated deactivation of OPP could support either of the presented hypotheses, i.e., role of KDPG in carbon catabolite repression or the other role of EDA in the proximity of TCA cycle might play a role in regulating OPP as neither of these scenarios are in the model. The complexity of the whole problem makes it very difficult to understand, from the multifunctionality of EDA, uncertainty related to ilvD functionality as EDD and KDPG metabolism, the unclear identity of metabolite(s) behind the temporal component of growth impairment under both discussed conditions, to metabolic plasticity of the central carbon metabolism of *Synechocystis*. Therefore, a thorough experimental and theoretical analysis is required to shed more light on the depths of *Synechocystis* metabolism.

## Data availability statement

The original contributions presented in the study are included in the article/Supplementary material, further inquiries can be directed to the corresponding author. The list of names, IDs and UniProt links of specific genes or enzymes mentioned or analysed in our work, is summarized in Supplementary file 7 (Table 3).

## Author contributions

JJ and AB jointly discussed ideas and concepts and wrote the manuscript. JJ was done modeling. AB was done bioinformatic analysis. Both authors contributed to the article and approved the submitted version.

## Footnotes

1
https://www.uniprot.org

2
https://www.ncbi.nlm.nih.gov/protein

3
https://www.genome.jp/kegg

## Supplementary material

Supplementary file 1 (Table 1) Occurrence of marker enzymes among cyanobacteria.

Supplementary file 2 (Table 2) List of kinetic parameters employed in the simulations. Data for autotrophic and mixotrophic conditions.

Supplementary file 3 (Data sheet 1) Pair wise sequence alignment of annotated EDD isoenzymes (UNIPROT) of *Calothrix desertica*. Tool used: EMBOSS Matcher.

Supplementary file 4 (Data sheet 2) Amino acid sequences for native EDD (6-phosphogluconate dehydratase) found among cyanobacteria.

Supplementary file 5 (Data sheet 3) Kinetic model of central carbon metabolism of *Synechocystis* sp. PCC 6803 under autotrophic conditions. Model description can be found in the section “General information about the model”.

Supplementary file 6 (Data sheet 4) Kinetic model of central carbon metabolism of *Synechocystis* sp. PCC 6803 under mixotrophic conditions. Model description can be found in the section “General information about the model”.

Supplementary File 7 (Table 3) List of names, IDs and UniProt links of specific genes or enzymes mentioned or analysed in our work.
