# Supplementary material for: Entner-Doudoroff pathway in Synechocystis PCC 6803: Proposed regulatory roles and enzyme multifunctionalities
Source: Front Microbiol. 2022 Aug 16;13:967545. doi: 10.3389/fmicb.2022.967545 (PMC9424857; doi:10.3389/fmicb.2022.967545)
Supplement: Supplementary file 3 (Data sheet 1) — Pair wise sequence alignment of annotated EDD isoenzymes (UNIPROT) of Calothrix desertica. Tool used: EMBOSS Matcher. [file Data_Sheet_1.pdf]

```
########################################
# Program: matcher
# Rundate: Fri 26 Feb 2021 20:07:56
# Commandline: matcher
#    -auto
#    -stdout
#    -asequence emboss_matcher-I20210226-200753-0205-41364580-p2m.asequence
#    -bsequence emboss_matcher-I20210226-200753-0205-41364580-p2m.bsequence
#    -datafile EBLOSUM62
#    -gapopen 14
#    -gapextend 4
#    -alternatives 1
#    -aformat3 pair
#    -sprotein1
#    -sprotein2
# Align_format: pair
# Report_file: stdout
########################################

#=======================================
#
# Aligned_sequences: 2
# 1: DSM106972_077550_Calothrix
# 2: DSM106972_030530_Calothrix
# Matrix: EBLOSUM62
# Gap_penalty: 14
# Extend_penalty: 4
#
# Length: 597
# Identity:     234/597 (39.2%)
# Similarity:   336/597 (56.3%)
# Gaps:          55/597 ( 9.2%)
# Score: 866
#
#
#=======================================

DSM106972_077      5 RALWRATGMHTEDFEKPIIAVANSFTQFVPGHVHLKDLGQLVCREIEASG     54
                     ||:.||||...|||.|.|:.|||::::..|.::.|.||.......|.|||
DSM106972_030     20 RAMLRATGFQDEDFNKAIVGVANAYSTITPCNMGLNDLAVRAQEGIRASG     69

DSM106972_077     55 AVAKEFNTIAVDDGIAMGHDGMLYSLPSREIIADSVEYMVNAHCADALVC    104
                     .:.:.|.||.|.|||:||.:||.|||.|||:||||:|...|....|.::.
DSM106972_030     70 GMPQVFGTITVSDGISMGTEGMKYSLVSREVIADSIETACNGESMDGVLT    119

DSM106972_077    105 ISNCDKITPGMLMAALRLNIPTVFVSGGPMEAGKTKLSEHKL-DLVDAMV    153
                     |..|||..||.::|..|:|||.:||.||.::.|..:..:..: ...:|:.
DSM106972_030    120 IGGCDKNMPGSMIAIARMNIPAIFVYGGTIKPGSYRECDLTVVSSFEAVG    169

DSM106972_077    154 VAADNSISDEVAEEYERSACPTCGSCSGMFTANSMNCLTEAIGLSLPGNG    203
                     ......|.:....|.|:.|||..|||.||:|||:|:...||:|:|||.:.
DSM106972_030    170 AFKAGKIPESELIEIEKRACPGAGSCGGMYTANTMSSAFEAMGMSLPYSS    219

DSM106972_077    204 TVLATHFDRKDLFLNAARTIVNITRRYYEQDDESILPRSIASFKAFVNAM    253
                     |:.|...::.:....:...:|...::       .:|||.|.:.|:..||:
DSM106972_030    220 TMSAEDAEKAESAEKSGYVLVEAIKK-------QLLPRHIITRKSIENAI    262

DSM106972_077    254 TLDIAMGGSTNTILHLLAAAHEANVNFTLTDIDRLSRKVPQLCKVAPNTP    303
                     ::.:|:|||||.:||.||.||.|.|...|.|.:.:..:||.||.:.|:.
DSM106972_030    263 SVIMAIGGSTNAVLHFLAIAHAAEVPLVLDDFETIRARVPVLCDLKPSG-    311

DSM106972_077    304 KYHVEDVHRAGGIYSILGELDRAELLHTDVPTVHSKTLKEALDHWDIMRT    353
                     :|...|:||||||..::..|...:|||:|..|:..:|:.|.|..
DSM106972_030    312 RYVATDLHRAGGIPQVMKMLLDHDLLHSDCITITGQTVAETLKD------    355
```

```
DSM106972_077    354 NSETVHTFFKAGPAGIPTQQAFSQSTRWASLDLDRENGCIRSIKNAFSTE    403
                                    ||::...:|..             ||........:
DSM106972_030    356 ---------------IPSEPPANQDI-------------IRPWSKPMYAQ    377

DSM106972_077    404 GGLAVLYGNLAERGCIVKTAGVDESIHVFKGKARIYESQDAAVKGILSDE    453
                     |.||:|.||||..|.:.|..||  .:....|.||::||::..:..||:|:
DSM106972_030    378 GHLAILKGNLATEGAVAKITGV--KLPQITGPARVFESEEECLDAILADK    425

DSM106972_077    454 VEPGDVVIIRYEGPRGGPGMQEMLYPTSYIKSKGLGKVCALLTDGRFSGG    503
                     ::|||:::||||||:|||||:|||.|||.|...|||....|:||||||||
DSM106972_030    426 IKPGDILVIRYEGPKGGPGMREMLAPTSAIIGAGLGDSVGLITDGRFSGG    475

DSM106972_077    504 TSGLSIGHASPEAAAGGNIALVRDGDLILIDIPNRSINVDISTEELSNRR    553
                     |.|:.:||.:||||.||.||||::||.|.||.|.|.:.::||.|||::||
DSM106972_030    476 TYGMVVGHVAPEAAVGGTIALVQEGDSITIDSPARLLQLNISEEELADRR    525

DSM106972_077    554 AAMEAKGKDAWKPEQKRQRRVTAALKAYALLATSADQGAVRNLEMLE    600
                     |        .|||:..|..:.|.|  .||.|..|:..|||.:|.:.|
DSM106972_030    526 A--------NWKPKPPRYTKGTLA--KYAKLVASSSLGAVTDLNLFE    562


#---------------------------------------
#---------------------------------------
```
