# Supplementary material for: Entner-Doudoroff pathway in Synechocystis PCC 6803: Proposed regulatory roles and enzyme multifunctionalities
Source: Front Microbiol. 2022 Aug 16;13:967545. doi: 10.3389/fmicb.2022.967545 (PMC9424857; doi:10.3389/fmicb.2022.967545)
Supplement: Supplementary file 4 (Data sheet 2) — Amino acid sequences for native EDD (6-phosphogluconate dehydratase) found among cyanobacteria. [file Data_Sheet_2.pdf]

Entner-Doudoroff pathway in *Synechocystis* PCC 6803: proposed regulatory roles and enzyme multifunctionalities

Amino acid sequences for native EDD (6-phosphogluconate dehydratase) found among cyanobacteria

>**Leptolyngbya valderiana_edd**

MHSTIQAVTDRIRERSGPTRSDYLDRLDAAVESGPSRARISCGNLAHAFAASGDDKPVLK

ALKAPNLGIVTAYNDMLSAHQPLEKFPALIKFAAQSRGCTAQVAGGVPAMCDGVTQGQPG

MELSLFSRDVIAQATAVALSHHTFDAALLLGVCDKIVPGLLIGALAFGHLPAILVPAGPM

PSGLPNKEKARVRKAHAAGEASREELLEAESRSYHSPGTCTFYGTANSNQMMMEVMGLHL

PGTAFVNPNTPLRERLTLAAADCACEITAQGDTFRPLGRTVDEKAIVNAMVGLAATGGST

NHAIHLVAIARAAGILINWDDLDEVSRATPLLTRIYPNGEADVNHFHEAGGMAFVIRELL

DAGRLHPDIATVHGGDLYAQAQDPFLDGDRLDWRPAIQSSRDTTILRPASDPFDPEGGIR

LLEGNLGRAIVKISAVDPAHRRIEARARVFDSQDELLEAFKAGELEGDLVAVIRGQGPHA

NGMPELHKLTPTLQVLQDRGQRVALLTDGRMSGASGSVLAAIHVVPEAARDGLIGRLRDG

DPIAIDAESGRFDCLVEATEMEAREAEPLVLDRYRFGVGRELFGGMREQVGDAESGASVL

FPDR

>**Nostoc sp._3335mG_edd**

MTVRQSIRDVTDRIAARSRDSRRDYLKRIEAAREAGVYRSSLSCGNLAHGFAACTPAEKA

ALAGNKTLNLGIVTSYNDMLSAHQPYQFYPDIIKEAAREMGATAQVAGGVPAMCDGVTQG

QPGMDLSLFSRDVIAMATAIALSHNMFDAAVYLGICDKIVPGLVIGALTFGHLPAVFIPA

GPMPSGIPNDEKSKVRQLYAEGKVGRAELLEAESKSYHSAGTCTFYGTANSNQMLMEIMG

LHLPGASFVNPGTPLRDALTREATKRALSLSAQGNDYTPIGHIIDEKAIVNGLVGLHATG

GSTNHTMHLIAMAAAAGLQVTWDDMSDLSDATPLLARVYPNGVADVNHFHAAGGMGFLIR

ELLDEGYLHEDVKTVWGAGLHNYTVEAKLIEDKLAFEPAPAESALPKVLTSARTPFQQTG

GLKLLTGNLGRSVIKVSAVKPEHRVVEAPARVFHGQDGLIAAFKAGELTGDVIAVVRFSG

PKAIGMPELHKLTPPLGILQDRGFKVALLTDGRMSGASGKVPAAIHMTPEAIDGGPISKI

RNGDLIRLDANEGTLTFLGDEKEFFSRTPAIEDLRPQQFGMGRELFAGFRNLVGVADRGA

SVFG

>**Nostoc sp. 3335mG_edd**

MTSLHPAVHAVTQRIVERSKPARERYLDLIERQRDAGVDRSRLGCANLAHGFAAAGEDKA
TIRAGAAMNIGIVTAYNDMLSAHQPYYRYPEQMKLWAREVGATAQVAGGVPAMCDGVTQG
YAGMELSLFSRDTIALSTAIGLSHGMFEGAAMLGICDKIVPGLLIGALRFGHLPMLLIPA
GPMPSGLANKEKQRIRQLYAEGKCSRDELLESEAASYHGAGTCTFYGTANSNQMMMEMMG
LHVPQSAFVNPGTKLRQALTRAAVHRVAEIGWDGDDYRPLGKVVDEKAVVNAAIGLLATG
GSTNHAIHLPAIARAAGIVIDWEDIDRLSSIVPLLARVYPNGSGDVNHFQAAGGLAYITK
TLLEHGLLHGDILTIAKGGFADYTADPRLDGEDLVWEPAPAEPLDASMVRPASDPFQADG
GMRLVKGNLGRAIFKTSAVDPSRWTIEAPARVFSDQLQVLEAFKAGELDRDVIVVVRFQG
PRANGMPELHKLTPPLGVLQDKGFKVALVTDGRMSGASGKVPAAIHVSPEVLGGGPLGKL
RDGDMVRLSAEEGSLEALVDFDEWEARQIADAPPPALGTGRELFAFMRNGADDAEKGGSA
MLAAMDAIV

>**Nostoc sp. 3335mG_edd**

MALNATIAAVTDRIIEASRTKRAAYLALIDRERESGVDRPMLGCANLAHAYAGTDEDRDA
MRPANRMNIGIVTAYNDMLSAHATYYRYPEQMKVWAREAGVTAQVAGGVPAMCDGVTQGF
PGMELSLFSRDTIALSTAVALSHGTFEGAALLGICDKIVPGLLMGALRFGHLPMVLIPGG
PMRSGIPNKQKAAVREAYAEGKVGREELLDAEIAAYHGKGTCTFYGTANSNQMMMEAMGL
HVPDAAFANPGTKLRQELTRHAVHRLAEIGWDGDDYRPIGRVVDEKAIVNAAIVLLATGG
STNHLIHLPAIARSAGIIIDWEDFNQLSQVVPLLTRVYPNGSADVNGFEDAGGPSFVIRE
LLAGGLMHGDVLTIARDGMAAYGRKSTIENDQLVWKEHGAVSGDDTIVRTVADPFNDEGG
FRILQGNIGRACIKVSAVDRDRWVIEAPCRVFSDQASVQAAFKAGELDRDVVVVVRHQGP
RANGMPELHKLTPPLGVLQNRGYRVALVTDGRMSGASGKVPAAIHLSPEALGGGPIGKLR
DGDIVRLDADANTLEALVPADEWAAREHAPVPPEADGTGREIFAMMRQGASEAEAGGSAM
LAAAGL

>**Moorea producens_edd**

MTLKPSGGKYQSLCDLKPSGRYVTVNLHRAGGIPQVMKMLLVHDLLHGDALTISGQTIAE
VLEEVPDQPPADQDVIRPWDNPVYAQGHLAILKGNLATEGSVAKITGVKNPQITGPARVF
ESEEACLDAILAGKINSGDVIVVRYEGPKGGPGMREMLAPTSAIIGAGLGDSVGLITDGR
FSGGTYGMVVGHVAPEAAVGGAIALVQEGDMITIDAHQRYLQLNVSDQELEQRRASWQPP
KPRYTTGVLAKYATLVSSSSVGAVTDLDLD

>**Moorea producens_edd**

MPENLRSQVVTQGVQRAPNRAMLRAVGFTDDDFTKPIVGLANGYSTITPCNLGLNDLAKR

AEAGLTTAGAMAQMFGTITISDGISMGTEGMKYSLVSREVIADSIETACNGQSMDGVLAI

GGCDKNMPGAMIAIARMNIPAIFVYGGTIKPGHLDGQDLTVVSAFEAVGQHSAGKIDEAK

LKAVELNACPGAGSCGGMFTANTMSSAFEAMGMSLPYSSTMAAEDIEKADSAEKSAFVLV

EAIRQQILPRAILTRKAFENAIAVIMAVGGSTNSVLHLLGIAHAAGVALSIDDFETIRGK

VPVPV

>**Chroococcidiopsis_Chr6712_edd**

MTLQNLQKQALQLSSSDRWQLVQVLLESLKQEAHLKLKRGNLLQLRGIAKTSTATEEIDV

KEDYVNYLTEKYQ
