# Supplementary material for: Entner-Doudoroff pathway in Synechocystis PCC 6803: Proposed regulatory roles and enzyme multifunctionalities
Source: Front Microbiol. 2022 Aug 16;13:967545. doi: 10.3389/fmicb.2022.967545 (PMC9424857; doi:10.3389/fmicb.2022.967545)
Supplement: Supplementary file 5 (Data sheet 3) — Kinetic model of central carbon metabolism of Synechocystis sp. PCC 6803 under autotrophic conditions. Model description can be found in the section “General information about the model”. [file Data_Sheet_3.pdf]

How to use: copy the code below without this text into .txt file and after saving, change the extension to .xml, then open it in MATLAB SimBiology


```
<?xml version="1.0" encoding="UTF-8"?>
<sbml xmlns="http://www.sbml.org/sbml/level2" xmlns:html="http://www.w3.org/1999/xhtml"
level="2" version="1">
  <annotation>
Created by The MathWorks, Inc. SimBiology tool, Version 3.3
</annotation>
  <model id="mw7798a38b_e877_43f5_9e96_34cfdf09edd2" name="initial">
    <notes>
      <body xmlns="http://www.w3.org/1999/xhtml"></body>
    </notes>
    <annotation>
      <COPASI xmlns="http://www.copasi.org/static/sbml">
        <rdf:RDF xmlns:dcterms="http://purl.org/dc/terms/"
xmlns:rdf="http://www.w3.org/1999/02/22-rdf-syntax-ns#">
          <rdf:Description rdf:about="#COPASI1">
            <dcterms:created>
              <rdf:Description>
                <dcterms:W3CDTF>2010-04-14T14:47:02Z</dcterms:W3CDTF>
              </rdf:Description>
            </dcterms:created>
          </rdf:Description>
        </rdf:RDF>
      </COPASI>
    </annotation>
    <listOfCompartments>
      <compartment id="mw52bf8910_f4a9_4914_a2fc_a38d17f3b84f" name="Stroma" size="1">
        <annotation>
          <COPASI xmlns="http://www.copasi.org/static/sbml">
```

```
        <rdf:RDF xmlns:dcterms="http://purl.org/dc/terms/" xmlns:rdf="http://www.w3.org/1999/02/22-rdf-syntax-ns#">
          <rdf:Description rdf:about="#COPASI2">
            <dcterms:created>
              <rdf:Description>
                <dcterms:W3CDTF>2010-04-14T14:56:43Z</dcterms:W3CDTF>
              </rdf:Description>
            </dcterms:created>
          </rdf:Description>
        </rdf:RDF>
      </COPASI>
    </annotation>
  </compartment>
  <compartment id="mw079afbc6_67d9_4a50_9b45_518eeb242fcc" name="external" size="1"/>
</listOfCompartments>
<listOfSpecies>
  <species id="mw5b3b2d76_cb36_4bfa_8e00_2fa00ad3e7c6" name="CO2" compartment="mw52bf8910_f4a9_4914_a2fc_a38d17f3b84f" initialAmount="0.02">
    <annotation>
      <COPASI xmlns="http://www.copasi.org/static/sbml">
        <rdf:RDF xmlns:dcterms="http://purl.org/dc/terms/" xmlns:rdf="http://www.w3.org/1999/02/22-rdf-syntax-ns#">
          <rdf:Description rdf:about="#COPASI3">
            <dcterms:created>
              <rdf:Description>
                <dcterms:W3CDTF>2010-04-14T15:46:19Z</dcterms:W3CDTF>
              </rdf:Description>
            </dcterms:created>
          </rdf:Description>
        </rdf:RDF>
      </COPASI>
    </annotation>
```

```
    </species>
    <species id="mw1e1e1695_f297_4f58_906b_5f76ada38e61" name="PGA" compartment="mw52bf8910_f4a9_4914_a2fc_a38d17f3b84f" initialAmount="4.7">
      <annotation>
        <COPASI xmlns="http://www.copasi.org/static/sbml">
          <rdf:RDF xmlns:dcterms="http://purl.org/dc/terms/" xmlns:rdf="http://www.w3.org/1999/02/22-rdf-syntax-ns#">
            <rdf:Description rdf:about="#COPASI4">
              <dcterms:created>
                <rdf:Description>
                  <dcterms:W3CDTF>2010-04-14T15:47:21Z</dcterms:W3CDTF>
                </rdf:Description>
              </dcterms:created>
            </rdf:Description>
          </rdf:RDF>
        </COPASI>
      </annotation>
    </species>
    <species id="mw2603ec03_ce86_4597_b49c_ba6ac6d68e0b" name="RuBP" compartment="mw52bf8910_f4a9_4914_a2fc_a38d17f3b84f" initialAmount="1">
      <annotation>
        <COPASI xmlns="http://www.copasi.org/static/sbml">
          <rdf:RDF xmlns:dcterms="http://purl.org/dc/terms/" xmlns:rdf="http://www.w3.org/1999/02/22-rdf-syntax-ns#">
            <rdf:Description rdf:about="#COPASI5">
              <dcterms:created>
                <rdf:Description>
                  <dcterms:W3CDTF>2010-04-14T15:47:38Z</dcterms:W3CDTF>
                </rdf:Description>
              </dcterms:created>
            </rdf:Description>
          </rdf:RDF>
```

</COPASI>

</annotation>

</species>

<species id="mw4e68b2da_34ac_4484_9cf5_03f0f6d9abe9" name="NADPH" compartment="mw52bf8910_f4a9_4914_a2fc_a38d17f3b84f" initialAmount="0.148995513447536">

<annotation>

<COPASI xmlns="http://www.copasi.org/static/sbml">

<rdf:RDF xmlns:dcterms="http://purl.org/dc/terms/" xmlns:rdf="http://www.w3.org/1999/02/22-rdf-syntax-ns#">

<rdf:Description rdf:about="#COPASI6">

<dcterms:created>

<rdf:Description>

<dcterms:W3CDTF>2010-04-14T15:47:02Z</dcterms:W3CDTF>

</rdf:Description>

</dcterms:created>

</rdf:Description>

</rdf:RDF>

</COPASI>

</annotation>

</species>

<species id="mwb534b1f7_d8d3_4922_9a23_493784f48e7d" name="O2" compartment="mw52bf8910_f4a9_4914_a2fc_a38d17f3b84f" initialAmount="0.0259999987479538" boundaryCondition="true" constant="true">

<annotation>

<COPASI xmlns="http://www.copasi.org/static/sbml">

<rdf:RDF xmlns:dcterms="http://purl.org/dc/terms/" xmlns:rdf="http://www.w3.org/1999/02/22-rdf-syntax-ns#">

<rdf:Description rdf:about="#COPASI7">

<dcterms:created>

<rdf:Description>

<dcterms:W3CDTF>2010-04-14T15:47:13Z</dcterms:W3CDTF>

</rdf:Description>

</dcterms:created>

</rdf:Description>

</rdf:RDF>

</COPASI>

</annotation>

</species>

<species id="mw264fafe4_178f_491d_8866_ba394e95a71b" name="SBP" compartment="mw52bf8910_f4a9_4914_a2fc_a38d17f3b84f" initialAmount="1.36261887752912">

<annotation>

<COPASI xmlns="http://www.copasi.org/static/sbml">

<rdf:RDF xmlns:dcterms="http://purl.org/dc/terms/" xmlns:rdf="http://www.w3.org/1999/02/22-rdf-syntax-ns#">

<rdf:Description rdf:about="#COPASI8">

<dcterms:created>

<rdf:Description>

<dcterms:W3CDTF>2010-04-14T15:47:55Z</dcterms:W3CDTF>

</rdf:Description>

</dcterms:created>

</rdf:Description>

</rdf:RDF>

</COPASI>

</annotation>

</species>

<species id="mw4b092c2e_9b30_46e1_85f7_9bb39d9b10aa" name="ADP" compartment="mw52bf8910_f4a9_4914_a2fc_a38d17f3b84f" initialAmount="0.344798607704336">

<annotation>

<COPASI xmlns="http://www.copasi.org/static/sbml">

<rdf:RDF xmlns:dcterms="http://purl.org/dc/terms/" xmlns:rdf="http://www.w3.org/1999/02/22-rdf-syntax-ns#">

<rdf:Description rdf:about="#COPASI9">

<dcterms:created>

<rdf:Description>

```
              <dcterms:W3CDTF>2010-04-14T16:01:25Z</dcterms:W3CDTF>
            </rdf:Description>
          </dcterms:created>
        </rdf:Description>
      </rdf:RDF>
    </COPASI>
  </annotation>
</species>
<species id="mwa083eb39_db7c_4dc7_a1b7_5d06c9120757" name="ATP"
compartment="mw52bf8910_f4a9_4914_a2fc_a38d17f3b84f" initialAmount="1.15520132006222">
  <annotation>
    <COPASI xmlns="http://www.copasi.org/static/sbml">
      <rdf:RDF xmlns:dcterms="http://purl.org/dc/terms/"
xmlns:rdf="http://www.w3.org/1999/02/22-rdf-syntax-ns#">
        <rdf:Description rdf:about="#COPASI10">
          <dcterms:created>
            <rdf:Description>
              <dcterms:W3CDTF>2010-04-14T16:01:14Z</dcterms:W3CDTF>
            </rdf:Description>
          </dcterms:created>
        </rdf:Description>
      </rdf:RDF>
    </COPASI>
  </annotation>
</species>
<species id="mw0c5a9637_94b1_47f0_87f9_8729e75ae8de" name="BPGA"
compartment="mw52bf8910_f4a9_4914_a2fc_a38d17f3b84f"
initialAmount="0.00748248557057663">
  <annotation>
    <COPASI xmlns="http://www.copasi.org/static/sbml">
      <rdf:RDF xmlns:dcterms="http://purl.org/dc/terms/"
xmlns:rdf="http://www.w3.org/1999/02/22-rdf-syntax-ns#">
        <rdf:Description rdf:about="#COPASI11">
```

<dcterms:created>
<rdf:Description>
<dcterms:W3CDTF>2010-04-14T16:01:08Z</dcterms:W3CDTF>
</rdf:Description>
</dcterms:created>
</rdf:Description>
</rdf:RDF>
</COPASI>
</annotation>
</species>
<species id="mw98624f9d_a464_456c_9a02_026d96612aff" name="GAP" compartment="mw52bf8910_f4a9_4914_a2fc_a38d17f3b84f" initialAmount="0.368362636458716">
<annotation>
<COPASI xmlns="http://www.copasi.org/static/sbml">
<rdf:RDF xmlns:dcterms="http://purl.org/dc/terms/" xmlns:rdf="http://www.w3.org/1999/02/22-rdf-syntax-ns#">
<rdf:Description rdf:about="#COPASI12">
<dcterms:created>
<rdf:Description>
<dcterms:W3CDTF>2010-06-08T23:46:46Z</dcterms:W3CDTF>
</rdf:Description>
</dcterms:created>
</rdf:Description>
</rdf:RDF>
</COPASI>
</annotation>
</species>
<species id="mwf5dc5c05_4d36_4ea4_8ee0_6a1cfd2ae49b" name="NADPp" compartment="mw52bf8910_f4a9_4914_a2fc_a38d17f3b84f" initialAmount="0.271004466327097">
<annotation>
<COPASI xmlns="http://www.copasi.org/static/sbml">

```
          <rdf:RDF xmlns:dcterms="http://purl.org/dc/terms/"
xmlns:rdf="http://www.w3.org/1999/02/22-rdf-syntax-ns#">
            <rdf:Description rdf:about="#COPASI13">
              <dcterms:created>
                <rdf:Description>
                  <dcterms:W3CDTF>2010-06-08T23:43:15Z</dcterms:W3CDTF>
                </rdf:Description>
              </dcterms:created>
            </rdf:Description>
          </rdf:RDF>
        </COPASI>
      </annotation>
    </species>
    <species id="mw34d9dd05_1772_4f59_ae50_4d6ef1f63225" name="Pi"
compartment="mw52bf8910_f4a9_4914_a2fc_a38d17f3b84f" initialAmount="4.99999975922188"
boundaryCondition="true" constant="true">
      <annotation>
        <COPASI xmlns="http://www.copasi.org/static/sbml">
          <rdf:RDF xmlns:dcterms="http://purl.org/dc/terms/"
xmlns:rdf="http://www.w3.org/1999/02/22-rdf-syntax-ns#">
            <rdf:Description rdf:about="#COPASI14">
              <dcterms:created>
                <rdf:Description>
                  <dcterms:W3CDTF>2010-05-18T18:10:26Z</dcterms:W3CDTF>
                </rdf:Description>
              </dcterms:created>
            </rdf:Description>
          </rdf:RDF>
        </COPASI>
      </annotation>
    </species>
    <species id="mw8bc33181_fd96_4636_93eb_674dfce67260" name="DHAP"
compartment="mw52bf8910_f4a9_4914_a2fc_a38d17f3b84f" initialAmount="0.012">
```

```
        <annotation>
          <COPASI xmlns="http://www.copasi.org/static/sbml">
            <rdf:RDF xmlns:dcterms="http://purl.org/dc/terms/"
xmlns:rdf="http://www.w3.org/1999/02/22-rdf-syntax-ns#">
              <rdf:Description rdf:about="#COPASI15">
                <dcterms:created>
                  <rdf:Description>
                    <dcterms:W3CDTF>2010-06-08T23:43:34Z</dcterms:W3CDTF>
                  </rdf:Description>
                </dcterms:created>
              </rdf:Description>
            </rdf:RDF>
          </COPASI>
        </annotation>
      </species>
      <species id="mw001a645f_0641_44d7_9a48_6a15ea7e4a51" name="FBP"
compartment="mw52bf8910_f4a9_4914_a2fc_a38d17f3b84f" initialAmount="0.02">
        <annotation>
          <COPASI xmlns="http://www.copasi.org/static/sbml">
            <rdf:RDF xmlns:dcterms="http://purl.org/dc/terms/"
xmlns:rdf="http://www.w3.org/1999/02/22-rdf-syntax-ns#">
              <rdf:Description rdf:about="#COPASI16">
                <dcterms:created>
                  <rdf:Description>
                    <dcterms:W3CDTF>2010-06-08T23:44:30Z</dcterms:W3CDTF>
                  </rdf:Description>
                </dcterms:created>
              </rdf:Description>
            </rdf:RDF>
          </COPASI>
        </annotation>
      </species>
```

<species id="mwf1237a2f_b6c2_4f60_aec2_f24eaff9e01a" name="F6P" compartment="mw52bf8910_f4a9_4914_a2fc_a38d17f3b84f" initialAmount="2">

<annotation>

<COPASI xmlns="http://www.copasi.org/static/sbml">

<rdf:RDF xmlns:dcterms="http://purl.org/dc/terms/" xmlns:rdf="http://www.w3.org/1999/02/22-rdf-syntax-ns#">

<rdf:Description rdf:about="#COPASI17">

<dcterms:created>

<rdf:Description>

<dcterms:W3CDTF>2010-06-08T23:44:21Z</dcterms:W3CDTF>

</rdf:Description>

</dcterms:created>

</rdf:Description>

</rdf:RDF>

</COPASI>

</annotation>

</species>

<species id="mw6c1ca071_aba1_4113_ace9_3d429d27bc60" name="E4P" compartment="mw52bf8910_f4a9_4914_a2fc_a38d17f3b84f" initialAmount="0.228820226685508">

<annotation>

<COPASI xmlns="http://www.copasi.org/static/sbml">

<rdf:RDF xmlns:dcterms="http://purl.org/dc/terms/" xmlns:rdf="http://www.w3.org/1999/02/22-rdf-syntax-ns#">

<rdf:Description rdf:about="#COPASI18">

<dcterms:created>

<rdf:Description>

<dcterms:W3CDTF>2010-06-08T23:43:55Z</dcterms:W3CDTF>

</rdf:Description>

</dcterms:created>

</rdf:Description>

</rdf:RDF>

</COPASI>

</annotation>

</species>

<species id="mwb2da70f3_0970_4374_bdf2_4a3914afa142" name="Xu5P" compartment="mw52bf8910_f4a9_4914_a2fc_a38d17f3b84f" initialAmount="0.0558077870361159">

<annotation>

<COPASI xmlns="http://www.copasi.org/static/sbml">

<rdf:RDF xmlns:dcterms="http://purl.org/dc/terms/" xmlns:rdf="http://www.w3.org/1999/02/22-rdf-syntax-ns#">

<rdf:Description rdf:about="#COPASI19">

<dcterms:created>

<rdf:Description>

<dcterms:W3CDTF>2010-06-08T23:41:49Z</dcterms:W3CDTF>

</rdf:Description>

</dcterms:created>

</rdf:Description>

</rdf:RDF>

</COPASI>

</annotation>

</species>

<species id="mw7b7d97f6_2db4_4473_80be_5e4d69e05b59" name="Ri5P" compartment="mw52bf8910_f4a9_4914_a2fc_a38d17f3b84f" initialAmount="0.0744045363011372">

<annotation>

<COPASI xmlns="http://www.copasi.org/static/sbml">

<rdf:RDF xmlns:dcterms="http://purl.org/dc/terms/" xmlns:rdf="http://www.w3.org/1999/02/22-rdf-syntax-ns#">

<rdf:Description rdf:about="#COPASI20">

<dcterms:created>

<rdf:Description>

<dcterms:W3CDTF>2010-06-08T23:41:08Z</dcterms:W3CDTF>

</rdf:Description>

</dcterms:created>

```
            </rdf:Description>
          </rdf:RDF>
        </COPASI>
      </annotation>
    </species>
    <species id="mwa5f72579_82b4_4f63_802b_ae5d613cef27" name="S7P"
compartment="mw52bf8910_f4a9_4914_a2fc_a38d17f3b84f"
initialAmount="0.511319423085088">
      <annotation>
        <COPASI xmlns="http://www.copasi.org/static/sbml">
          <rdf:RDF xmlns:dcterms="http://purl.org/dc/terms/"
xmlns:rdf="http://www.w3.org/1999/02/22-rdf-syntax-ns#">
            <rdf:Description rdf:about="#COPASI21">
              <dcterms:created>
                <rdf:Description>
                  <dcterms:W3CDTF>2010-06-08T23:47:56Z</dcterms:W3CDTF>
                </rdf:Description>
              </dcterms:created>
            </rdf:Description>
          </rdf:RDF>
        </COPASI>
      </annotation>
    </species>
    <species id="mw580d5da5_ec62_4ea2_8f87_a5d617982c02" name="Ru5P"
compartment="mw52bf8910_f4a9_4914_a2fc_a38d17f3b84f"
initialAmount="0.026898222838205">
      <annotation>
        <COPASI xmlns="http://www.copasi.org/static/sbml">
          <rdf:RDF xmlns:dcterms="http://purl.org/dc/terms/"
xmlns:rdf="http://www.w3.org/1999/02/22-rdf-syntax-ns#">
            <rdf:Description rdf:about="#COPASI22">
              <dcterms:created>
                <rdf:Description>
```

<dcterms:W3CDTF>2010-06-08T23:41:17Z</dcterms:W3CDTF>
</rdf:Description>
</dcterms:created>
</rdf:Description>
</rdf:RDF>
</COPASI>
</annotation>
</species>
<species id="mwc5d2bfb5_b299_4e35_b8b7_6f7ff0ce2a5a" name="G6P" compartment="mw52bf8910_f4a9_4914_a2fc_a38d17f3b84f" initialAmount="1.94057630985951">
<annotation>
<COPASI xmlns="http://www.copasi.org/static/sbml">
<rdf:RDF xmlns:dcterms="http://purl.org/dc/terms/" xmlns:rdf="http://www.w3.org/1999/02/22-rdf-syntax-ns#">
<rdf:Description rdf:about="#COPASI23">
<dcterms:created>
<rdf:Description>
<dcterms:W3CDTF>2010-06-08T23:46:39Z</dcterms:W3CDTF>
</rdf:Description>
</dcterms:created>
</rdf:Description>
</rdf:RDF>
</COPASI>
</annotation>
</species>
<species id="mwc1711fc7_ff82_413c_8a9c_248c948df9bc" name="Sink1" compartment="mw52bf8910_f4a9_4914_a2fc_a38d17f3b84f" initialAmount="0">
<annotation>
<COPASI xmlns="http://www.copasi.org/static/sbml">
<rdf:RDF xmlns:dcterms="http://purl.org/dc/terms/" xmlns:rdf="http://www.w3.org/1999/02/22-rdf-syntax-ns#">
<rdf:Description rdf:about="#COPASI25">

```
            <dcterms:created>
              <rdf:Description>
                <dcterms:W3CDTF>2011-05-10T00:14:21Z</dcterms:W3CDTF>
              </rdf:Description>
            </dcterms:created>
          </rdf:Description>
        </rdf:RDF>
      </COPASI>
    </annotation>
  </species>
  <species id="mw3bf50b2f_519c_495b_b72a_b0753138f38d" name="Sink2"
compartment="mw52bf8910_f4a9_4914_a2fc_a38d17f3b84f" initialAmount="0">
    <annotation>
      <COPASI xmlns="http://www.copasi.org/static/sbml">
        <rdf:RDF xmlns:dcterms="http://purl.org/dc/terms/"
xmlns:rdf="http://www.w3.org/1999/02/22-rdf-syntax-ns#">
          <rdf:Description rdf:about="#COPASI26">
            <dcterms:created>
              <rdf:Description>
                <dcterms:W3CDTF>2011-04-26T00:48:45Z</dcterms:W3CDTF>
              </rdf:Description>
            </dcterms:created>
          </rdf:Description>
        </rdf:RDF>
      </COPASI>
    </annotation>
  </species>
  <species id="mw69137d0b_f529_4e9a_b7ef_33dffd020665" name="PGCA"
compartment="mw52bf8910_f4a9_4914_a2fc_a38d17f3b84f" initialAmount="0.01">
    <annotation>
      <COPASI xmlns="http://www.copasi.org/static/sbml">
```

<rdf:RDF xmlns:dcterms="http://purl.org/dc/terms/" xmlns:rdf="http://www.w3.org/1999/02/22-rdf-syntax-ns#">

<rdf:Description rdf:about="#COPASI27">

<dcterms:created>

<rdf:Description>

<dcterms:W3CDTF>2011-06-15T16:28:14Z</dcterms:W3CDTF>

</rdf:Description>

</dcterms:created>

</rdf:Description>

</rdf:RDF>

</COPASI>

</annotation>

</species>

<species id="mw62b862f7_f0c7_48df_895b_319e5b84b577" name="GCA" compartment="mw52bf8910_f4a9_4914_a2fc_a38d17f3b84f" initialAmount="0.0123593835433703">

<annotation>

<COPASI xmlns="http://www.copasi.org/static/sbml">

<rdf:RDF xmlns:dcterms="http://purl.org/dc/terms/" xmlns:rdf="http://www.w3.org/1999/02/22-rdf-syntax-ns#">

<rdf:Description rdf:about="#COPASI28">

<dcterms:created>

<rdf:Description>

<dcterms:W3CDTF>2011-06-15T16:28:15Z</dcterms:W3CDTF>

</rdf:Description>

</dcterms:created>

</rdf:Description>

</rdf:RDF>

</COPASI>

</annotation>

</species>

<species id="mwa60ca2e1_0142_4294_8726_6b633c50a6bf" name="GOA" compartment="mw52bf8910_f4a9_4914_a2fc_a38d17f3b84f" initialAmount="0.0327363830028097">

<annotation>

<COPASI xmlns="http://www.copasi.org/static/sbml">

<rdf:RDF xmlns:dcterms="http://purl.org/dc/terms/" xmlns:rdf="http://www.w3.org/1999/02/22-rdf-syntax-ns#">

<rdf:Description rdf:about="#COPASI29">

<dcterms:created>

<rdf:Description>

<dcterms:W3CDTF>2011-06-15T16:28:23Z</dcterms:W3CDTF>

</rdf:Description>

</dcterms:created>

</rdf:Description>

</rdf:RDF>

</COPASI>

</annotation>

</species>

<species id="mw7ee6ca0b_cc39_45ef_a044_36cfeb983c0b" name="GLY" compartment="mw52bf8910_f4a9_4914_a2fc_a38d17f3b84f" initialAmount="0.8">

<annotation>

<COPASI xmlns="http://www.copasi.org/static/sbml">

<rdf:RDF xmlns:dcterms="http://purl.org/dc/terms/" xmlns:rdf="http://www.w3.org/1999/02/22-rdf-syntax-ns#">

<rdf:Description rdf:about="#COPASI30">

<dcterms:created>

<rdf:Description>

<dcterms:W3CDTF>2011-06-15T16:27:59Z</dcterms:W3CDTF>

</rdf:Description>

</dcterms:created>

</rdf:Description>

</rdf:RDF>

</COPASI>

</annotation>

</species>

<species id="mw4bff44c2_7520_41bc_80fb_bd781f8b6503" name="SER" compartment="mw52bf8910_f4a9_4914_a2fc_a38d17f3b84f" initialAmount="0.5">

<annotation>

<COPASI xmlns="http://www.copasi.org/static/sbml">

<rdf:RDF xmlns:dcterms="http://purl.org/dc/terms/" xmlns:rdf="http://www.w3.org/1999/02/22-rdf-syntax-ns#">

<rdf:Description rdf:about="#COPASI31">

<dcterms:created>

<rdf:Description>

<dcterms:W3CDTF>2011-06-15T16:28:35Z</dcterms:W3CDTF>

</rdf:Description>

</dcterms:created>

</rdf:Description>

</rdf:RDF>

</COPASI>

</annotation>

</species>

<species id="mw881e6414_8bc5_4885_b415_3467a94c3d32" name="HPR" compartment="mw52bf8910_f4a9_4914_a2fc_a38d17f3b84f" initialAmount="0.1">

<annotation>

<COPASI xmlns="http://www.copasi.org/static/sbml">

<rdf:RDF xmlns:dcterms="http://purl.org/dc/terms/" xmlns:rdf="http://www.w3.org/1999/02/22-rdf-syntax-ns#">

<rdf:Description rdf:about="#COPASI32">

<dcterms:created>

<rdf:Description>

<dcterms:W3CDTF>2011-06-15T16:38:48Z</dcterms:W3CDTF>

</rdf:Description>

</dcterms:created>

</rdf:Description>

```
        </rdf:RDF>
      </COPASI>
    </annotation>
  </species>
  <species id="mw924156fa_35b2_403e_891e_fbc962e777d9" name="GCEA"
compartment="mw52bf8910_f4a9_4914_a2fc_a38d17f3b84f"
initialAmount="0.00400097153227282">
    <annotation>
      <COPASI xmlns="http://www.copasi.org/static/sbml">
        <rdf:RDF xmlns:dcterms="http://purl.org/dc/terms/"
xmlns:rdf="http://www.w3.org/1999/02/22-rdf-syntax-ns#">
          <rdf:Description rdf:about="#COPASI33">
            <dcterms:created>
              <rdf:Description>
                <dcterms:W3CDTF>2011-06-15T16:30:28Z</dcterms:W3CDTF>
              </rdf:Description>
            </dcterms:created>
          </rdf:Description>
        </rdf:RDF>
      </COPASI>
    </annotation>
  </species>
  <species id="mwc3b39320_8ede_4d49_ba08_c569fbe1474d" name="PEP"
compartment="mw52bf8910_f4a9_4914_a2fc_a38d17f3b84f" initialAmount="4.19091918967107">
    <annotation>
      <COPASI xmlns="http://www.copasi.org/static/sbml">
        <rdf:RDF xmlns:dcterms="http://purl.org/dc/terms/"
xmlns:rdf="http://www.w3.org/1999/02/22-rdf-syntax-ns#">
          <rdf:Description rdf:about="#COPASI34">
            <dcterms:created>
              <rdf:Description>
                <dcterms:W3CDTF>2011-05-10T16:27:35Z</dcterms:W3CDTF>
              </rdf:Description>
```

```
        </dcterms:created>
      </rdf:Description>
    </rdf:RDF>
  </COPASI>
</annotation>
</species>
<species id="mw1b5d434c_13de_4b56_bd7d_627742a6ceae" name="Sink3"
compartment="mw52bf8910_f4a9_4914_a2fc_a38d17f3b84f" initialAmount="0">
<annotation>
  <COPASI xmlns="http://www.copasi.org/static/sbml">
    <rdf:RDF xmlns:dcterms="http://purl.org/dc/terms/"
xmlns:rdf="http://www.w3.org/1999/02/22-rdf-syntax-ns#">
      <rdf:Description rdf:about="#COPASI35">
        <dcterms:created>
          <rdf:Description>
            <dcterms:W3CDTF>2011-05-10T16:30:50Z</dcterms:W3CDTF>
          </rdf:Description>
        </dcterms:created>
      </rdf:Description>
    </rdf:RDF>
  </COPASI>
</annotation>
</species>
<species id="mw6a9b239b_a9cc_43c8_b083_35f106e0485d" name="Sink4"
compartment="mw52bf8910_f4a9_4914_a2fc_a38d17f3b84f" initialAmount="0">
<annotation>
  <COPASI xmlns="http://www.copasi.org/static/sbml">
    <rdf:RDF xmlns:dcterms="http://purl.org/dc/terms/"
xmlns:rdf="http://www.w3.org/1999/02/22-rdf-syntax-ns#">
      <rdf:Description rdf:about="#COPASI36">
        <dcterms:created>
          <rdf:Description>
```

<dcterms:W3CDTF>2011-06-15T16:28:43Z</dcterms:W3CDTF>
</rdf:Description>
</dcterms:created>
</rdf:Description>
</rdf:RDF>
</COPASI>
</annotation>
</species>
<species id="mwcd2b43d1_7092_45b9_af63_c926207ad771" name="ppGA" compartment="mw52bf8910_f4a9_4914_a2fc_a38d17f3b84f" initialAmount="4.19091917967427">
<annotation>
<COPASI xmlns="http://www.copasi.org/static/sbml">
<rdf:RDF xmlns:dcterms="http://purl.org/dc/terms/" xmlns:rdf="http://www.w3.org/1999/02/22-rdf-syntax-ns#">
<rdf:Description rdf:about="#COPASI37">
<dcterms:created>
<rdf:Description>
<dcterms:W3CDTF>2011-06-15T16:30:09Z</dcterms:W3CDTF>
</rdf:Description>
</dcterms:created>
</rdf:Description>
</rdf:RDF>
</COPASI>
</annotation>
</species>
<species id="mw142bb566_7067_4fc2_b957_dc196c94324a" name="TSA" compartment="mw52bf8910_f4a9_4914_a2fc_a38d17f3b84f" initialAmount="0.0324331443243821">
<annotation>
<COPASI xmlns="http://www.copasi.org/static/sbml">
<rdf:RDF xmlns:dcterms="http://purl.org/dc/terms/" xmlns:rdf="http://www.w3.org/1999/02/22-rdf-syntax-ns#">
<rdf:Description rdf:about="#COPASI38">

```
<dcterms:created>
<rdf:Description>
<dcterms:W3CDTF>2011-06-15T16:42:10Z</dcterms:W3CDTF>
</rdf:Description>
</dcterms:created>
</rdf:Description>
</rdf:RDF>
</COPASI>
</annotation>
</species>
<species id="mwa86b050a_b141_4f76_9597_674909286fc9" name="OXA"
compartment="mw52bf8910_f4a9_4914_a2fc_a38d17f3b84f" initialAmount="0.1">
<annotation>
<COPASI xmlns="http://www.copasi.org/static/sbml">
<rdf:RDF xmlns:dcterms="http://purl.org/dc/terms/"
xmlns:rdf="http://www.w3.org/1999/02/22-rdf-syntax-ns#">
<rdf:Description rdf:about="#COPASI39">
<dcterms:created>
<rdf:Description>
<dcterms:W3CDTF>2011-06-28T01:15:08Z</dcterms:W3CDTF>
</rdf:Description>
</dcterms:created>
</rdf:Description>
</rdf:RDF>
</COPASI>
</annotation>
</species>
<species id="mw72c1af91_f65b_4bf6_bad0_b78d4b2e6890" name="ATPr"
compartment="mw52bf8910_f4a9_4914_a2fc_a38d17f3b84f" initialAmount="0"/>
<species id="mw121ef383_adea_44fd_af43_a9fef1ca3455" name="Mass"
compartment="mw52bf8910_f4a9_4914_a2fc_a38d17f3b84f" initialAmount="0"/>
<species id="mwacd9e1e6_f4cc_4f7e_9a51_0a8732e6ddde" name="Sink_DHAP"
compartment="mw52bf8910_f4a9_4914_a2fc_a38d17f3b84f" initialAmount="0"/>
```

<species id="mw2ac0cdfb_0f6b_484c_b7b5_41cfbdace9b8" name="Sink_E4P" compartment="mw52bf8910_f4a9_4914_a2fc_a38d17f3b84f" initialAmount="0"/>

<species id="mw6c2cc5c8_f456_40b7_929f_e3d9e37f99b2" name="Sink_Ri5P" compartment="mw52bf8910_f4a9_4914_a2fc_a38d17f3b84f" initialAmount="0"/>

<species id="mwdc722523_d3fc_41a7_99f4_eea898e1ed4f" name="Sink_PEP" compartment="mw52bf8910_f4a9_4914_a2fc_a38d17f3b84f" initialAmount="0"/>

<species id="mw975386b7_cfea_40d4_849f_bf8f1b96464a" name="Sink_GAP" compartment="mw52bf8910_f4a9_4914_a2fc_a38d17f3b84f" initialAmount="0"/>

<species id="mw4cf40e73_d04d_403a_8a00_f19be586107d" name="NADPHr" compartment="mw52bf8910_f4a9_4914_a2fc_a38d17f3b84f" initialAmount="0"/>

<species id="mwdd5682ca_fe92_487e_857d_3f58bed7a828" name="rate_CC_1" compartment="mw52bf8910_f4a9_4914_a2fc_a38d17f3b84f" initialAmount="0"/>

<species id="mw2f3e9def_c2a4_4cfa_8c12_ee1aac19b5aa" name="rate_CC_2" compartment="mw52bf8910_f4a9_4914_a2fc_a38d17f3b84f" initialAmount="0"/>

<species id="mwb1050bab_824b_40ea_9230_6623b1126122" name="rate_CC_3" compartment="mw52bf8910_f4a9_4914_a2fc_a38d17f3b84f" initialAmount="0"/>

<species id="mwd959437e_e966_44f4_a058_a4d962b5fb20" name="rate_CC_4" compartment="mw52bf8910_f4a9_4914_a2fc_a38d17f3b84f" initialAmount="0"/>

<species id="mwc965bd74_dc1e_428c_b262_e88fb94a03c6" name="rate_CC_5" compartment="mw52bf8910_f4a9_4914_a2fc_a38d17f3b84f" initialAmount="0"/>

<species id="mw763d2415_d551_49b6_9ea8_6765ded6374a" name="rate_CC_6" compartment="mw52bf8910_f4a9_4914_a2fc_a38d17f3b84f" initialAmount="0"/>

<species id="mw51d0f400_64de_478b_9486_a4d146737c8a" name="rate_CC_7" compartment="mw52bf8910_f4a9_4914_a2fc_a38d17f3b84f" initialAmount="0"/>

<species id="mw6e5d7f26_34e6_45a3_9e3b_b8384ba79cd6" name="rate_CC_8" compartment="mw52bf8910_f4a9_4914_a2fc_a38d17f3b84f" initialAmount="0"/>

<species id="mw20e1f4be_43c3_4abc_a168_ee18dd77b40e" name="rate_CC_9" compartment="mw52bf8910_f4a9_4914_a2fc_a38d17f3b84f" initialAmount="0"/>

<species id="mw6d4e3500_0ac5_4333_8ca6_ee6f58266982" name="rate_CC_10" compartment="mw52bf8910_f4a9_4914_a2fc_a38d17f3b84f" initialAmount="0"/>

<species id="mwf14fae22_a889_4245_b539_05c724efdd58" name="rate_CC_11" compartment="mw52bf8910_f4a9_4914_a2fc_a38d17f3b84f" initialAmount="0"/>

<species id="mwf26c9e95_3acc_4dab_a40f_48aa0b8d9442" name="rate_CC_12" compartment="mw52bf8910_f4a9_4914_a2fc_a38d17f3b84f" initialAmount="0"/>

<species id="mw7509fc1d_6172_41cf_9e31_08c39accd539" name="rate_CC_13" compartment="mw52bf8910_f4a9_4914_a2fc_a38d17f3b84f" initialAmount="0"/>

<species id="mwe64ace10_5b0b_414d_9f57_9fd87b03174e" name="rate_PFK" compartment="mw52bf8910_f4a9_4914_a2fc_a38d17f3b84f" initialAmount="0"/>

<species id="mw31b2d210_181c_4d37_8e6f_dea686ada228" name="rate_PGM" compartment="mw52bf8910_f4a9_4914_a2fc_a38d17f3b84f" initialAmount="0"/>

<species id="mwfb0df553_4b52_4b95_9154_bb66087e5593" name="rate_ENO" compartment="mw52bf8910_f4a9_4914_a2fc_a38d17f3b84f" initialAmount="0"/>

<species id="mw80bf3a05_9be7_4dfa_baec_a5142f07eb3b" name="rate_glyc_GAP" compartment="mw52bf8910_f4a9_4914_a2fc_a38d17f3b84f" initialAmount="0"/>

<species id="mw04299979_e245_4206_9f43_d70b28685aeb" name="rate_PEPsink" compartment="mw52bf8910_f4a9_4914_a2fc_a38d17f3b84f" initialAmount="0"/>

<species id="mw179364ce_63fb_4c6a_a11b_75a2ce49ae4d" name="Sink_G6P" compartment="mw52bf8910_f4a9_4914_a2fc_a38d17f3b84f" initialAmount="0"/>

<species id="mw260a4232_836b_47e0_9e60_c60b15f0ecd5" name="rate_G6Psink" compartment="mw52bf8910_f4a9_4914_a2fc_a38d17f3b84f" initialAmount="0"/>

<species id="mw34c708ab_f8fd_4daf_8e29_2096cb7e1563" name="rate_GAPsink" compartment="mw52bf8910_f4a9_4914_a2fc_a38d17f3b84f" initialAmount="0"/>

<species id="mwf634a4ad_468b_4f81_a966_d081677b2bd6" name="rate_E4Psink" compartment="mw52bf8910_f4a9_4914_a2fc_a38d17f3b84f" initialAmount="0"/>

<species id="mwa912d5b8_71c9_4d1b_ae93_7fe4563570ea" name="rate_Ri5Psink" compartment="mw52bf8910_f4a9_4914_a2fc_a38d17f3b84f" initialAmount="0"/>

<species id="mwe1915c23_bc54_4444_a6b6_afb9f835d8cf" name="AceP" compartment="mw52bf8910_f4a9_4914_a2fc_a38d17f3b84f" initialAmount="0"/>

<species id="mwa94479e8_4e60_4fc5_9681_8c8e54d33e74" name="Sink_AceP" compartment="mw52bf8910_f4a9_4914_a2fc_a38d17f3b84f" initialAmount="0"/>

<species id="mw630d7a9f_d907_496c_b750_6c23afbb4658" name="P6G" compartment="mw52bf8910_f4a9_4914_a2fc_a38d17f3b84f" initialAmount="0"/>

<species id="mwce034c6e_3ba3_4a8c_bd2f_94b469c74eeb" name="OPP_rate" compartment="mw52bf8910_f4a9_4914_a2fc_a38d17f3b84f" initialAmount="0"/>

<species id="mw76a409bd_32d8_438e_8ec2_80106d26e956" name="rate_GSM1" compartment="mw52bf8910_f4a9_4914_a2fc_a38d17f3b84f" initialAmount="0"/>

<species id="mw22183e88_4ec6_4ae3_9e1d_0e66b0c75c7d" name="netC6_PFK" compartment="mw52bf8910_f4a9_4914_a2fc_a38d17f3b84f" initialAmount="0"/>

<species id="mwc153f931_c15d_4f00_915f_d828490f3e0c" name="rate_PKET1" compartment="mw52bf8910_f4a9_4914_a2fc_a38d17f3b84f" initialAmount="0"/>

<species id="mw6679025e_faa6_4ddb_9090_6b6b0c957d8c" name="rate_PKET2" compartment="mw52bf8910_f4a9_4914_a2fc_a38d17f3b84f" initialAmount="0"/>

<species id="mwd81831e8_a743_4783_ae7e_bc4e8ecef290" name="Pyr" compartment="mw52bf8910_f4a9_4914_a2fc_a38d17f3b84f" initialAmount="0"/>

<species id="mwee5b965c_bc88_4930_9408_a56b03f8777d" name="rate_EDglyc" compartment="mw52bf8910_f4a9_4914_a2fc_a38d17f3b84f" initialAmount="0"/>

<species id="mw4237c9a8_dcd7_4b30_8ef0_8cecd69c9694" name="rate_GPI" compartment="mw52bf8910_f4a9_4914_a2fc_a38d17f3b84f" initialAmount="0"/>

<species id="mw5682e6fc_33b0_4c3f_83a6_0027e7f9efcc" name="rate_glucose" compartment="mw52bf8910_f4a9_4914_a2fc_a38d17f3b84f" initialAmount="0"/>

<species id="mwb4d18f64_6a6f_4ee2_9e60_e95453fd193a" name="rate_PFK1" compartment="mw52bf8910_f4a9_4914_a2fc_a38d17f3b84f" initialAmount="0"/>

<species id="mw5d306209_83b3_4fd3_b2d3_3d0821016185" name="rate_PFK2" compartment="mw52bf8910_f4a9_4914_a2fc_a38d17f3b84f" initialAmount="0"/>

<species id="mw9c6265f4_0d84_4317_85e3_1397b925dd6b" name="rate_ALDO1_CC_5" compartment="mw52bf8910_f4a9_4914_a2fc_a38d17f3b84f" initialAmount="0"/>

<species id="mwd2ecc838_3e25_4d27_a551_24c2d4281c13" name="rate_ALDO2_CC_5" compartment="mw52bf8910_f4a9_4914_a2fc_a38d17f3b84f" initialAmount="0"/>

<species id="mw39355e27_2c4e_4381_9725_324749e9dfd0" name="rate_PGM1" compartment="mw52bf8910_f4a9_4914_a2fc_a38d17f3b84f" initialAmount="0"/>

<species id="mwc0bac73f_72bd_4dfc_8db5_38f7dbadfa15" name="rate_PGM2" compartment="mw52bf8910_f4a9_4914_a2fc_a38d17f3b84f" initialAmount="0"/>

<species id="mwb358c90c_fcdf_46a5_a8c4_d6c214ad7409" name="rate_PGM3" compartment="mw52bf8910_f4a9_4914_a2fc_a38d17f3b84f" initialAmount="0"/>

<species id="mwbf496d1e_e804_42a8_a991_17b4cf79d939" name="PhotoRes" compartment="mw52bf8910_f4a9_4914_a2fc_a38d17f3b84f" initialAmount="0"/>

<species id="mwcd4b57d6_55a3_45b7_a37e_2d3d83b4bec8" name="net_CC_3" compartment="mw52bf8910_f4a9_4914_a2fc_a38d17f3b84f" initialAmount="0"/>

<species id="mw67386f72_70ed_4213_b83a_51849feda20a" name="OPP2_rate" compartment="mw52bf8910_f4a9_4914_a2fc_a38d17f3b84f" initialAmount="0"/>

<species id="mw41061232_3fb7_429f_920c_084b45725d79" name="rate_PKET1a" compartment="mw52bf8910_f4a9_4914_a2fc_a38d17f3b84f" initialAmount="0"/>

<species id="mwaafe132e_e071_4509_96cb_b4a56a9eee89" name="rate_PKET1b" compartment="mw52bf8910_f4a9_4914_a2fc_a38d17f3b84f" initialAmount="0"/>

<species id="mwb5e1728d_1e5f_4709_b9f3_36151c3f7fe1" name="rate_PKET2a" compartment="mw52bf8910_f4a9_4914_a2fc_a38d17f3b84f" initialAmount="0"/>

<species id="mw2a2436c2_8c5c_4876_bdb1_1941dfad77e4" name="rate_PKET2b" compartment="mw52bf8910_f4a9_4914_a2fc_a38d17f3b84f" initialAmount="0"/>

<species id="mw01be9f1c_a2a5_492c_bcd3_f7f50fab164f" name="KDPG" compartment="mw52bf8910_f4a9_4914_a2fc_a38d17f3b84f" initialAmount="0"/>

<species id="mw4fc9d1b8_3a21_4910_bf03_3e90cc702de3" name="CO2" compartment="mw079afbc6_67d9_4a50_9b45_518eeb242fcc" initialAmount="0.1" boundaryCondition="true" constant="true"/>

```
      <species id="mw5c9d3afc_2037_42d9_a8c6_e341b406cf37" name="G6P" compartment="mw079afbc6_67d9_4a50_9b45_518eeb242fcc" initialAmount="1" boundaryCondition="true" constant="true"/>
    </listOfSpecies>
    <listOfParameters>
      <parameter id="mwf2a81dda_e6ba_4ef2_82e7_ac9c7c029cd8" name="kf_ATP" value="0.04">
        <annotation>
          <COPASI xmlns="http://www.copasi.org/static/sbml">
            <rdf:RDF xmlns:dcterms="http://purl.org/dc/terms/" xmlns:rdf="http://www.w3.org/1999/02/22-rdf-syntax-ns#">
              <rdf:Description rdf:about="#COPASI47">
                <dcterms:created>
                  <rdf:Description>
                    <dcterms:W3CDTF>2010-04-14T15:40:36Z</dcterms:W3CDTF>
                  </rdf:Description>
                </dcterms:created>
              </rdf:Description>
            </rdf:RDF>
          </COPASI>
        </annotation>
      </parameter>
      <parameter id="mw942ee080_9495_43cb_8637_43348cad5712" name="kf_NADPH" value="0.1218">
        <annotation>
          <COPASI xmlns="http://www.copasi.org/static/sbml">
            <rdf:RDF xmlns:dcterms="http://purl.org/dc/terms/" xmlns:rdf="http://www.w3.org/1999/02/22-rdf-syntax-ns#">
              <rdf:Description rdf:about="#COPASI48">
                <dcterms:created>
                  <rdf:Description>
                    <dcterms:W3CDTF>2010-07-25T02:11:54Z</dcterms:W3CDTF>
                  </rdf:Description>
                </dcterms:created>
```

```xml
</rdf:Description>

</rdf:RDF>

</COPASI>

</annotation>

</parameter>

<parameter id="mwe1a7456e_ee15_425a_a731_b7dd46838115" name="V_PP1" value="0.048"/>

<parameter id="mw3eb7fb4b_04fb_4fb5_a285_b18042ae9a2a" name="V1" value="0.952"/>

<parameter id="mw5b9d10ab_7daf_4921_a8cb_86028e0d31e6" name="V5" value="2.6928"/>

<parameter id="mwec7d9352_a492_407e_b7e6_2b403e29e97c" name="V2" value="1.7243"/>

<parameter id="mw33659ae3_6e62_49ef_a1c2_a33adc049723" name="V3" value="0.42344"/>

<parameter id="mw696548be_270b_4aa4_a957_53f3540f254b" name="V4" value="0.0784792"/>

<parameter id="mw48d7d26d_fe30_4b1e_a4ee_a4eef1cfcb6b" name="V6" value="1.7255"/>

<parameter id="mwc5b9dbbd_d536_4b37_9dd4_99d0328d3414" name="V7" value="1.75584"/>

<parameter id="mwc4332dd6_0358_4c1a_ae97_1131d7ea48c2" name="V8" value="2.3664"/>

<parameter id="mw9c962784_a662_43bc_816d_58889af49cc2" name="V9" value="1.8186875"/>

<parameter id="mw520d09f0_2f53_4caa_8bd1_f1066e47098f" name="V10" value="0.55097"/>

<parameter id="mw9ac027ed_faba_463f_8575_f0e6d69200bd" name="V11" value="0.96"/>

<parameter id="mw634909f8_5cc7_478f_b6b4_3d713718216b" name="V12" value="0.46904"/>

<parameter id="mw442d2399_d826_45dc_96d1_1d9a3af70032" name="V13" value="0.4257"/>

<parameter id="mw1dc7ae44_7d88_420e_8084_bc013cd8b1dc" name="V_SS1" value="0.6237"/>

<parameter id="mw6d3e7fb2_fbcb_4028_ae3c_377e5ec0c707" name="KE_SS1" value="3"/>

<parameter id="mw94c1a2ea_8406_444a_beaf_3ae6b855d7b5" name="V_Sink_E4P" value="0.00012"/>

<parameter id="mwcb26c99a_ae43_4c60_ae4d_a62a4a7bf1d1" name="V_Sink_Ri5P" value="0.005"/>

<parameter id="mwac20e884_51a5_40a9_b093_21b8def92417" name="V_PP2a" value="0.000588"/>

<parameter id="mw0a05a9a7_f8e0_4216_9a36_0a20398d0fa7" name="V_PP3" value="0.0042"/>
```

```xml
<parameter id="mw928bed3b_b2b3_42b5_89f3_a5090846accf" name="V_PP4" value="0.1806"/>
<parameter id="mw02354494_0fe5_4a28_b05b_89e41b0d76e7" name="V_PP5" value="0.002"/>
<parameter id="mwcc8ed7db_a4f4_435d_9ed4_449f30e69058" name="V_PP6" value="0.004"/>
<parameter id="mw557b4a1b_a1b0_4840_a606_ec763580e90c" name="V_PP7" value="0.013"/>
<parameter id="mw10b1b1bc_dce8_475d_b4fa_c06a66547e4e" name="V_Sink_PEP" value="0.0010584"/>
<parameter id="mwd12025b7_9ac7_4a08_ae36_005fa0a2ad82" name="V_synth_SER" value="0.00024"/>
<parameter id="mw94b56681_0cac_45dc_b68e_1fda032f53b9" name="V_TSA1" value="0.01"/>
<parameter id="mwba16aae1_effd_497c_bbdc_ecb17a65553e" name="V_TSA2" value="0.023"/>
<parameter id="mwc67e78e3_63c8_4314_ae67_03f4ba59bc51" name="V_TSA3" value="0.1"/>
<parameter id="mw6870894e_d5a3_4bb2_8096_8de1303cdbb9" name="V_OXA1" value="0.096"/>
<parameter id="mwa9080cfb_3bd3_4be1_9e3d_57f259d6e494" name="V_OXA2" value="0.000268"/>
<parameter id="mw587a43b1_6567_47d8_ab28_db86856d6189" name="KE4" value="0.59835"/>
<parameter id="mwc97c27ed_cba8_4dda_8745_bc8e8da8a6b2" name="KE7" value="0.509"/>
<parameter id="mwe11dcb8e_d7c1_423b_bbe4_5da671bd64ca" name="V_PFK" value="1.91139"/>
<parameter id="mw70e0ec5e_4fd7_4384_ace0_bb4f0fe59eb5" name="Km112b" value="0.1"/>
<parameter id="mw9aac7a54_37ae_4bc1_abc6_f66f5ce326f8" name="V_PP2b" value="0.00039"/>
<parameter id="mwbd86004a_c6e8_4b82_b6bc_a1fc8148a985" name="Km112a" value="3"/>
<parameter id="mw48edc399_e14c_4e4e_8fd5_27aa7a43e91a" name="Kms_enol" value="0.525"/>
<parameter id="mw28cc925f_1ec1_40e8_a64d_bacb34b7b472" name="Kmp_enol" value="0.279"/>
<parameter id="mw75e9f4a7_4ba0_493e_b8ce_844e0daf2309" name="K_Sink_PEP" value="0.674"/>
<parameter id="mwa7315a55_d6f0_400f_8bd5_a60441cade2d" name="V_Sink_GLY" value="0.00044"/>
```

```
<parameter id="mw02fc51b4_e03b_401c_bcf1_da0d8fd704ab" name="V_Sink_SER" value="0.000679"/>

<parameter id="mwa540938d_712a_46c8_b3d5_bff2c38919f8" name="KE5" value="3.5829"/>

<parameter id="mwfa3dac90_b557_4ea0_950c_b2e6cea1f624" name="Keq_PGM" value="0.71"/>

<parameter id="mw2091e1f5_0cd3_4d23_9dcf_9f54ae671686" name="Kmp_PGM_alpha" value="0.74"/>

<parameter id="mw407bee91_c458_4b42_ad72_7dea8c0f84b8" name="Kms_PGM_alpha" value="0.189"/>

<parameter id="mw4354fd87_eec3_45ff_ba09_8e66f6cec0d4" name="Vf_PGM_alpha" value="0.2688"/>

<parameter id="mw51232471_96c5_44a5_9906_91a4af9717da" name="Kmp_PGM_beta" value="0.005"/>

<parameter id="mw099e3275_3aed_4b7c_abd7_1d978c4681d1" name="Kms_PGM_beta" value="0.277"/>

<parameter id="mw9e3b0877_c7fe_4a60_886f_4c0b97f63ef9" name="Vf_PGM_beta" value="0.5538"/>

<parameter id="mw93985682_472b_4e3b_895b_705c1b0929b3" name="Kmp_PGM_gama" value="0.6"/>

<parameter id="mwefaeb6d1_a2ae_41cb_b478_f28cb1be6a7c" name="Kms_PGM_gama" value="2.58"/>

<parameter id="mwa466c9c9_3297_437c_baed_0dce17c19743" name="Vf_PGM_gama" value="0.0115092"/>

<parameter id="mw60d16eba_0bba_4387_94f0_ddda983743a2" name="Keq_enol" value="0.866"/>

<parameter id="mw1560b05b_911d_4737_8105_b669df533ad3" name="Vf_enol" value="0.826292"/>

<parameter id="mw9aa3463d_ca8c_4f73_a1b9_b2cd84be87b9" name="kf_CO2" value="0.26931"/>

<parameter id="mw7815c29b_f589_487d_8abf_8c69866a16bf" name="V_Sink_GAP" value="9e-005"/>

<parameter id="mw0e500944_5cfc_4224_907b_dd0573a17387" name="K_Sink_GAP" value="0.1"/>

<parameter id="mw2b15c7a2_60bd_4539_bbca_ea2887c83c7e" name="Kgap" value="1"/>

<parameter id="mw42d232ce_870c_4c6c_80e1_b80db63c24dc" name="Knadpp" value="2"/>

<parameter id="mw66c149b7_107b_4f1e_8ba3_e998a2eb4341" name="Kpga" value="0.357"/>
```

```xml
<parameter id="mw30cb04f1_447a_49ab_9566_af33f6c68f02" name="Vgap_dehyd" value="1.07756"/>

<parameter id="mw3f587528_6a1e_45cf_85a1_c83170fa3db2" name="knadph" value="0.927"/>

<parameter id="mw86a32edf_a962_44c9_adfe_8059b0c656c9" name="KM61" value="7.82"/>

<parameter id="mw08f10a43_f575_462a_b1d4_5b4943f2aecf" name="KE8" value="1.75"/>

<parameter id="mwdab67499_dc15_4f7a_b42a_b553d395bdeb" name="KE10" value="0.576"/>

<parameter id="mw3143b349_becb_4982_a83f_441e274ee00c" name="KE11" value="2.4"/>

<parameter id="mw9973b455_04bb_4ef2_8bad_e5d9038f2afa" name="KE12" value="0.477"/>

<parameter id="mw0d24671c_626c_4751_a2f5_52d895c46b12" name="KM132" value="0.05"/>

<parameter id="mw561b9d1e_a922_4d5d_a419_705bdcdc1479" name="K_GOA" value="0.1"/>

<parameter id="mw71ca7b9e_7a15_48c2_af0a_3b49048dc22c" name="V_GLY_syn" value="0.1"/>

<parameter id="mw52f105dd_e643_48db_81f5_b4895cb10150" name="KM11" value="0.0115"/>

<parameter id="mw506e2da2_c32d_4748_a086_6dc0aaae9d55" name="KM12" value="0.222"/>

<parameter id="mwe54dda3a_7a37_48ba_bfe5_43bf6fd9d3b9" name="KI11" value="0.84"/>

<parameter id="mwf40efeb1_e89c_424e_8006_a9095546bee5" name="KI14" value="0.9"/>

<parameter id="mw7c678412_1b31_4b4a_a7d6_fbbc27d86166" name="KM13" value="0.02"/>

<parameter id="mw753db9ce_1d29_4e20_a0f6_0e6e9639bd53" name="KI12" value="0.04"/>

<parameter id="mw52296718_7c93_45fd_9b1e_c13a865f9e2b" name="KI13" value="0.075"/>

<parameter id="mwb6a831eb_5143_4bc3_9230_f7756d19cbb8" name="KI15" value="0.07"/>

<parameter id="mw9d2a6be6_f7f4_4871_82c1_5a6d5cf40bbc" name="KE2" value="0.000616"/>

<parameter id="mw82fe7cf0_c731_49ce_9621_2c502956630b" name="KM31" value="0.004"/>

<parameter id="mw0f926ea1_951f_45a7_b0e8_afa94ba266f9" name="KM32" value="0.1"/>

<parameter id="mw5d188609_730f_4a50_84a3_654056528e7a" name="KI62" value="12"/>

<parameter id="mwae1595b9_af51_4b6b_b328_ae530ded7fe0" name="KI61" value="0.7"/>

<parameter id="mw353036af_5e46_4c9c_92b2_191591d2c857" name="KI135" value="0.4"/>

<parameter id="mweb5b46a0_a515_418a_a2c7_5986240a61ff" name="KI131" value="2"/>

<parameter id="mw55d72d42_b4f1_4c2d_8b39_594a18219b62" name="KI134" value="2.5"/>

<parameter id="mwdacf95a3_1ffa_43b0_8a8b_04a0c41ea664" name="KI133" value="4"/>

<parameter id="mw62747a3d_d67f_443e_bf91_3c16927d07c9" name="KI132" value="0.7"/>

<parameter id="mw51f910d1_180e_4a92_aa3e_17f247a0f4fe" name="KM131" value="0.05"/>
```

```xml
<parameter id="mw88fca50d_8086_41ed_9f8b_bfce18be9463" name="K_Sink_G6P" value="2"/>
<parameter id="mw2f7b3fed_abd8_460f_aa2b_6034d9dc3ffc" name="V_Sink_G6P" value="0.0035"/>
<parameter id="mw8638e2a1_77bb_4b82_9b9b_0be22fe4d7e4" name="K_Sink_AceP" value="1"/>
<parameter id="mw4ffd888c_f8cc_4980_b408_b8c7d289c5aa" name="V_Sink_AceP" value="1"/>
<parameter id="mw663cb8cd_1897_4185_98e4_23ce87526c47" name="K_OXA1" value="2"/>
<parameter id="mw2c0ecf40_827b_4e66_af45_4bbcef400f33" name="K_OXA2" value="5"/>
<parameter id="mw4c067e5e_f048_4dc4_bc7d_8e95972e89b1" name="V_PFK_beta" value="0.19647"/>
<parameter id="mw361ddcb4_26fd_439c_b85e_c582a34f84b1" name="Kmp_PPP1" value="0.1"/>
<parameter id="mw2db94c60_e05f_4408_bc54_54fb9f9f7d42" name="Kms_PPP1" value="2"/>
<parameter id="mw9726ca8c_0c0e_4d16_bae1_3d731a5408aa" name="V_PPP1" value="0.54"/>
<parameter id="mw47eee472_fb8a_4673_8f9b_3bd3ced88c79" name="Kms_nadpp_ppp2" value="1"/>
<parameter id="mw4b31666e_aef2_4756_8e67_3da9f3276f7e" name="Kms_p6g_ppp2" value="1.2"/>
<parameter id="mw6d34a400_fde1_4a71_81d0_322fd5573e89" name="V_PPP2" value="0.3"/>
<parameter id="mwd6c6584f_18c7_4c2e_928b_bc7cebf26f01" name="K_Sink_E4P" value="0.1"/>
<parameter id="mw73931537_4826_46e8_9efc_5a58ea02c555" name="K_Sink_Ri5P" value="0.1"/>
<parameter id="mw55bd15f3_cb3c_4fda_b599_ac7410bffac2" name="KI1121" value="94"/>
<parameter id="mwaea44262_a9e9_4810_9d88_b4d97c605854" name="KI1122" value="2.55"/>
<parameter id="mw356de434_dd62_4e14_b35b_d2e87ddcc2d8" name="Km112c" value="0.5"/>
<parameter id="mw95d51cdd_5101_48f8_ad60_b5c37d37c5de" name="V_PP2c" value="0.001003"/>
<parameter id="mwd9ae7c23_c98b_4e49_8f0c_1c722eaa18d6" name="V8b" value="0.972"/>
<parameter id="mwad7edeb0_57d8_44dc_af42_e15e631d77bf" name="K_TSA1" value="0.1"/>
<parameter id="mwfb4dc7ff_b572_42e3_8611_ff50379e3543" name="K_TSA2" value="0.4"/>
<parameter id="mwdc293431_8b07_49fe_8303_4cb1a50e1b25" name="K_TSA3" value="0.1"/>
<parameter id="mw5395f222_63ed_4a50_b204_433a6b1f6cbd" name="K_Sink_SER" value="1"/>
```

```xml
<parameter id="mw09424d24_2a0c_4c48_99fd_174b17fae4c6" name="K_synth_SER" value="2"/>

<parameter id="mw6f910a3d_c12b_4c9c_8602_d4487e9568ac" name="K_Sink_GLY" value="1"/>

<parameter id="mw97764192_3b51_4a4b_b6af_86b67f201f3d" name="Km121" value="0.1"/>

<parameter id="mw56c6a02a_7634_401b_b487_a5ee2fdaa884" name="Km1231" value="0.09"/>

<parameter id="mw5bf928d1_2a99_48f9_ac13_ecba1a3e04ad" name="Km1131" value="0.21"/>

<parameter id="mwa21e7932_75e2_471d_9fb1_bcb876fbbd76" name="Km1132" value="0.25"/>

<parameter id="mwfe4b8452_607e_443f_9448_4fe832048e5d" name="Km1241" value="0.15"/>

<parameter id="mwffd5861f_1df3_4e1b_b6f8_ca0b6da59319" name="Km1242" value="1.7"/>

<parameter id="mw3554f65b_6b0b_4025_be9b_44db5077d8e3" name="KI124" value="2"/>

<parameter id="mw4149ab27_a9d5_449b_9996_8c4f87c94e9f" name="KE113" value="300"/>

<parameter id="mw2ad9e2a3_9d3b_4185_8007_b877627c8032" name="KI113" value="0.36"/>

<parameter id="mw1270f895_d29a_44e6_82fb_b7e4a5b90172" name="KE124" value="607"/>

<parameter id="mwcbeed6ea_4576_4b4a_950a_7b8ba162bb5b" name="KE123" value="250000"/>

<parameter id="mw34409210_66ed_415b_a829_f6dbf66524f4" name="KI123" value="12"/>

<parameter id="mw9acd4aeb_fdb0_4a5e_833f_e3946722d810" name="KM_ATP_PFK" value="15.87"/>

<parameter id="mw921c626f_edc9_441b_8547_3c7edacfc4e3" name="KM_F6P_PFK" value="11.84"/>

<parameter id="mw4f29d2d9_0857_4db2_880a_535c67780929" name="KM_ATP_PFK_beta" value="9.38"/>

<parameter id="mw5d3b7d4f_cb37_4189_808f_9b316593f3fd" name="KM_F6P_PFK_beta" value="6.43"/>

<parameter id="mw60c4515d_f1f6_46fd_9cb5_2c58ee609270" name="KM2_ADP" value="0.2458"/>

<parameter id="mw8a532dd1_c07c_4f64_89c8_0e880b187648" name="KM2_ATP" value="1.99"/>

<parameter id="mwe85d1b9f_58eb_48ae_8033_765e75b5bc89" name="KM2_BPGA" value="0.107"/>

<parameter id="mwdaffc0cc_4d0d_49e1_ac01_aaad36724d06" name="KM2_PGA" value="1.2126"/>
```

```xml
<parameter id="mw6e36548d_68ee_4f99_bdf4_bc0081dec083" name="KM4_DHAP" value="0.76"/>
<parameter id="mw18156bae_84ae_479f_a552_4b556b807e65" name="KM4_GAP" value="0.3708"/>
<parameter id="mw2664b98d_62f2_4597_97e0_ac8bf9badc1c" name="KM5_8_DHAP" value="0.1212"/>
<parameter id="mw6f8f2f0d_dfd6_4df4_b62b_5cc576a99618" name="KM5_8_E4P" value="0.5718"/>
<parameter id="mw7d7ad1c0_08d2_429b_89a5_44adb5fc08f2" name="KM5_8_FBP" value="1.38"/>
<parameter id="mw1ef19eb9_d783_49e0_ab98_1512e9923aff" name="KM5_8_GAP" value="1.794"/>
<parameter id="mwed39790d_132b_4762_acf2_55e9d5d068be" name="KM5_8_SBP" value="2.03"/>
<parameter id="mwe3eb2841_b1eb_4dc4_9c4c_a5d8c5cc75cd" name="KM5b_8b_DHAP" value="0.6438"/>
<parameter id="mw2881ad2b_2458_44be_919a_8a2cddc2a765" name="KM5b_8b_E4P" value="0.1581"/>
<parameter id="mw343b1085_b0e0_4e60_8805_60c5ae4ec26b" name="KM5b_8b_FBP" value="1.772"/>
<parameter id="mwc2e367c9_e93b_406b_a5db_e77794a92b22" name="KM5b_8b_GAP" value="2.3765"/>
<parameter id="mw301fbb5f_9697_492b_8f32_015458d521c5" name="KM5b_8b_SBP" value="1.73"/>
<parameter id="mw16c6ff04_2e36_4d15_bccb_59d7661e10cb" name="V5b" value="1.6225"/>
<parameter id="mweb78553f_492c_44fc_b049_30f4089f5e52" name="KM9_FBP" value="1.71"/>
<parameter id="mw65b811e9_89c2_407c_a470_8f071a6422d5" name="KM9_SBP" value="0.11"/>
<parameter id="mw2df7359c_5ca8_46cf_830c_64f2aef66d6a" name="KM7_10_E4P" value="1.3675"/>
<parameter id="mwf76b7498_870d_4272_aed2_2e9cdad4acd9" name="KM7_10_F6P" value="1.233"/>
<parameter id="mw1c53df26_ee90_4319_898d_ab1565c18d13" name="KM7_10_GAP" value="1.054"/>
<parameter id="mw319586e5_21bf_4aef_ac3a_736836d7e448" name="KM7_10_Ri5P" value="0.5363"/>
```

```
<parameter id="mw5d1be663_9187_43e2_95e7_5852c1c840a8" name="KM7_10_S7P" value="2.006"/>
<parameter id="mw5f7db393_8e3c_4a77_9cfc_395acd74d218" name="KM7_10_Xu5P" value="0.0685"/>
<parameter id="mw576b0b82_5947_49ce_8f54_e086cc631504" name="KM11_Ri5P" value="0.1"/>
<parameter id="mw53176689_3f53_433a_a683_9edf99a5993d" name="KM11_Ru5P" value="1.1"/>
<parameter id="mw916809f8_ec94_4f28_aaaa_56521dfe234b" name="KM12_Ru5P" value="0.439"/>
<parameter id="mw770450fd_fa82_4c62_8132_cbc289403f10" name="KM12_Xu5P" value="0.6"/>
<parameter id="mw3bfe76ae_7a7c_4327_8477_c7664ca39e64" name="KM_Sink_Pyr" value="0.021"/>
<parameter id="mw13783949_2260_41c5_a254_46b337a4ec55" name="V_Sink_Pyr" value="0.6"/>
<parameter id="mwc6aa212e_7e00_4697_af9f_140f4ef235ea" name="kf_G6P" value="0"/>
<parameter id="mw38214f65_8acd_4a84_b1d6_59c785833902" name="KM_F6P_SS1" value="1"/>
<parameter id="mw7a8cfa64_22c5_4f72_b851_6fa78ce1d00a" name="KM_G6P_SS1" value="1"/>
<parameter id="mw29daf52a_3537_43f2_b889_86b8d087fab9" name="KM_PKET1a_AceP" value="1.765818327"/>
<parameter id="mw35d79455_d586_4f7e_b53d_b9b05403c18f" name="KM_PKET1a_E4P" value="0.708995427"/>
<parameter id="mw95025ff8_a096_4b4e_b30a_c86490520d3c" name="KM_PKET1a_F6P" value="0.195859927"/>
<parameter id="mw4231ba04_0406_4368_925a_599001509b3f" name="KM_PKET1a_GAP" value="0.001101781"/>
<parameter id="mwcc132bf2_7c6b_4cbb_b3e3_caddab999b30" name="KM_PKET1a_Xu5P" value="1.255418228"/>
<parameter id="mwac1b2180_f1af_4819_9233_94af7fae5fe8" name="Keq_PKETa" value="0.2896651"/>
<parameter id="mw14672e46_52f5_4ab7_bff1_4b749b587f1d" name="V_PKET1a" value="0.87544734676"/>
<parameter id="mw07fd01bf_c788_41e8_ad93_f9663c71e731" name="KM_PKET1b_AceP" value="0.674920019"/>
```

```xml
<parameter id="mwf1cc2080_281e_420a_bea7_9f1ace706f8b" name="KM_PKET1b_E4P" value="0.973496654"/>
<parameter id="mw08464d63_135a_4871_9108_9a430e7da3c9" name="KM_PKET1b_F6P" value="0.073704394"/>
<parameter id="mw4925fe87_c38b_4c63_b257_76e6a00ba813" name="KM_PKET1b_GAP" value="0.126696262"/>
<parameter id="mw94c699e4_fed2_455f_9f56_63394e10e6aa" name="KM_PKET1b_Xu5P" value="0.315391885"/>
<parameter id="mwb043257b_34eb_45c4_b4bd_7fadfb6e19c8" name="Keq_PKETb" value="0.471060325"/>
<parameter id="mwe6cc0f80_5d7e_478d_8df3_785b611461a8" name="V_PKET1b" value="0.03457700124"/>
<parameter id="mw48d5b78e_f2bf_4cae_92f9_9b4b7e011e9a" name="KM_PKET2b_AceP" value="0.708589676"/>
<parameter id="mw36675533_f3f1_443f_a00a_e9f3ab7b4e43" name="KM_PKET2b_E4P" value="0.001478515"/>
<parameter id="mw04c1dd77_705d_42f9_a0ad_169227a654aa" name="KM_PKET2b_F6P" value="0.71897234"/>
<parameter id="mwc941b16b_73fe_48ea_828c_7d4127c52bdc" name="KM_PKET2b_GAP" value="0.051244493"/>
<parameter id="mw6aa54137_d905_4ffa_a29c_b192c4901c8f" name="KM_PKET2b_Xu5P" value="0.79285068"/>
<parameter id="mwbfebafa8_c3ee_48cc_843e_9a4bad33bfb3" name="V_PKET2b" value="0.0535589002"/>
<parameter id="mwffdd6cd4_59fc_4211_a076_bf7bccbb2861" name="KM_PKET2a_AceP" value="0.347537253"/>
<parameter id="mw1b9a6d90_0834_45b2_80c3_894c8f3806df" name="KM_PKET2a_E4P" value="0.564845446"/>
<parameter id="mw235e9584_299a_49b0_917e_b62cb998dc8e" name="KM_PKET2a_F6P" value="0.21030757"/>
<parameter id="mwee148dba_6936_4ff5_98e9_3fafc08bf46c" name="KM_PKET2a_GAP" value="1.219287928"/>
<parameter id="mwe2c4c957_b4a0_41f4_aacc_9a73304177bf" name="KM_PKET2a_Xu5P" value="0.669382423"/>
<parameter id="mwb9289074_0186_4371_aed9_7470cba640dc" name="V_PKET2a" value="6.995326e-005"/>
<parameter id="mw3cd3e9a1_dbe4_41c7_870c_fb4e65d18298" name="KI_KDPG" value="1.5"/>
```

```xml
<parameter id="mw68578e28_5e7b_43b7_a251_4585ec996637" name="Km_edd_P6G" value="1"/>

<parameter id="mwc689e60c_5ebe_4df0_9311_a4fa4858f41d" name="V_edd" value="0.005925"/>

<parameter id="mwe9068a7b_a4f6_4f0d_b901_8440765efbaf" name="Km_eda_KDPG" value="1"/>

<parameter id="mw7d24069f_e15e_473e_99dc_4e20bd4238c9" name="V_eda" value="0.1"/>

</listOfParameters>

<listOfRules>

<assignmentRule metaid="repeatedAssignment_mw3605ba42_5588_4342_a33e_1fb6d9661e63" variable="mw72c1af91_f65b_4bf6_bad0_b78d4b2e6890">

<math xmlns="http://www.w3.org/1998/Math/MathML">

<apply>

<divide/>

<ci> mwa083eb39_db7c_4dc7_a1b7_5d06c9120757 </ci>

<apply>

<plus/>

<ci> mwa083eb39_db7c_4dc7_a1b7_5d06c9120757 </ci>

<ci> mw4b092c2e_9b30_46e1_85f7_9bb39d9b10aa </ci>

</apply>

</apply>

</math>

</assignmentRule>

<assignmentRule metaid="repeatedAssignment_mwb069880c_8805_48c3_a372_8790a9bbfe65" variable="mw4cf40e73_d04d_403a_8a00_f19be586107d">

<math xmlns="http://www.w3.org/1998/Math/MathML">

<apply>

<divide/>

<ci> mw4e68b2da_34ac_4484_9cf5_03f0f6d9abe9 </ci>

<apply>

<plus/>

<ci> mw4e68b2da_34ac_4484_9cf5_03f0f6d9abe9 </ci>

<ci> mwf5dc5c05_4d36_4ea4_8ee0_6a1cfd2ae49b </ci>
```

```
        </apply>
      </apply>
    </math>
  </assignmentRule>
  <assignmentRule metaid="repeatedAssignment_mwfdc74a3c_2f3b_45c4_af86_10669b6ff8e1" variable="mwdd5682ca_fe92_487e_857d_3f58bed7a828">
    <math xmlns="http://www.w3.org/1998/Math/MathML">
      <apply>
        <divide/>
        <apply>
          <times/>
          <ci> mw2603ec03_ce86_4597_b49c_ba6ac6d68e0b </ci>
          <apply>
            <divide/>
            <apply>
              <times/>
              <ci> mw3eb7fb4b_04fb_4fb5_a285_b18042ae9a2a </ci>
              <ci> mw5b3b2d76_cb36_4bfa_8e00_2fa00ad3e7c6 </ci>
            </apply>
            <apply>
              <plus/>
              <ci> mw5b3b2d76_cb36_4bfa_8e00_2fa00ad3e7c6 </ci>
              <apply>
                <times/>
                <ci> mw52f105dd_e643_48db_81f5_b4895cb10150 </ci>
                <apply>
                  <plus/>
                  <cn type="integer"> 1 </cn>
                  <apply>
                    <divide/>
                    <ci> mwb534b1f7_d8d3_4922_9a23_493784f48e7d </ci>
```

```
          <ci> mw506e2da2_c32d_4748_a086_6dc0aaae9d55 </ci>
         </apply>
        </apply>
       </apply>
      </apply>
     </apply>
    </apply>
    <apply>
     <plus/>
     <ci> mw2603ec03_ce86_4597_b49c_ba6ac6d68e0b </ci>
     <apply>
      <times/>
      <ci> mw7c678412_1b31_4b4a_a7d6_fbbc27d86166 </ci>
      <apply>
       <plus/>
       <cn type="integer"> 1 </cn>
       <apply>
        <divide/>
        <ci> mw1e1e1695_f297_4f58_906b_5f76ada38e61 </ci>
        <ci> mwe54dda3a_7a37_48ba_bfe5_43bf6fd9d3b9 </ci>
       </apply>
       <apply>
        <divide/>
        <ci> mw001a645f_0641_44d7_9a48_6a15ea7e4a51 </ci>
        <ci> mw753db9ce_1d29_4e20_a0f6_0e6e9639bd53 </ci>
       </apply>
       <apply>
        <divide/>
        <ci> mw264fafe4_178f_491d_8866_ba394e95a71b </ci>
        <ci> mw52296718_7c93_45fd_9b1e_c13a865f9e2b </ci>
       </apply>
```

```
            <apply>
              <divide/>
              <ci> mw34d9dd05_1772_4f59_ae50_4d6ef1f63225 </ci>
              <ci> mwf40efeb1_e89c_424e_8006_a9095546bee5 </ci>
            </apply>
            <apply>
              <divide/>
              <ci> mw4e68b2da_34ac_4484_9cf5_03f0f6d9abe9 </ci>
              <ci> mwb6a831eb_5143_4bc3_9230_f7756d19cbb8 </ci>
            </apply>
          </apply>
        </apply>
      </apply>
    </apply>
  </math>
</assignmentRule>
<assignmentRule metaid="repeatedAssignment_mwe5e62827_34a1_46ad_9fe7_85d590f0f61f" variable="mw2f3e9def_c2a4_4cfa_8c12_ee1aac19b5aa">
  <math xmlns="http://www.w3.org/1998/Math/MathML">
    <apply>
      <divide/>
      <apply>
        <divide/>
        <apply>
          <times/>
          <ci> mwec7d9352_a492_407e_b7e6_2b403e29e97c </ci>
          <apply>
            <minus/>
            <apply>
              <times/>
              <ci> mw1e1e1695_f297_4f58_906b_5f76ada38e61 </ci>
```

```
        <ci> mwa083eb39_db7c_4dc7_a1b7_5d06c9120757 </ci>
      </apply>
      <apply>
        <divide/>
        <apply>
          <times/>
          <ci> mw0c5a9637_94b1_47f0_87f9_8729e75ae8de </ci>
          <ci> mw4b092c2e_9b30_46e1_85f7_9bb39d9b10aa </ci>
        </apply>
        <ci> mw9d2a6be6_f7f4_4871_82c1_5a6d5cf40bbc </ci>
      </apply>
    </apply>
  </apply>
  <apply>
    <times/>
    <ci> mwdaffc0cc_4d0d_49e1_ac01_aaad36724d06 </ci>
    <ci> mw8a532dd1_c07c_4f64_89c8_0e880b187648 </ci>
  </apply>
</apply>
<apply>
  <times/>
  <apply>
    <plus/>
    <cn type="integer"> 1 </cn>
    <apply>
      <divide/>
      <ci> mw1e1e1695_f297_4f58_906b_5f76ada38e61 </ci>
      <ci> mwdaffc0cc_4d0d_49e1_ac01_aaad36724d06 </ci>
    </apply>
    <apply>
      <divide/>
```

```
          <ci> mw0c5a9637_94b1_47f0_87f9_8729e75ae8de </ci>
          <ci> mwe85d1b9f_58eb_48ae_8033_765e75b5bc89 </ci>
        </apply>
      </apply>
      <apply>
        <plus/>
        <cn type="integer"> 1 </cn>
        <apply>
          <divide/>
          <ci> mwa083eb39_db7c_4dc7_a1b7_5d06c9120757 </ci>
          <ci> mw8a532dd1_c07c_4f64_89c8_0e880b187648 </ci>
        </apply>
        <apply>
          <divide/>
          <ci> mw4b092c2e_9b30_46e1_85f7_9bb39d9b10aa </ci>
          <ci> mw60c4515d_f1f6_46fd_9cb5_2c58ee609270 </ci>
        </apply>
      </apply>
    </apply>
  </apply>
</math>
</assignmentRule>
<assignmentRule metaid="repeatedAssignment_mw4f880f25_ab7b_4271_8575_d3cabb1bdcc5"
variable="mwb1050bab_824b_40ea_9230_6623b1126122">
  <math xmlns="http://www.w3.org/1998/Math/MathML">
    <apply>
      <divide/>
      <apply>
        <times/>
        <ci> mw33659ae3_6e62_49ef_a1c2_a33adc049723 </ci>
        <ci> mw0c5a9637_94b1_47f0_87f9_8729e75ae8de </ci>
```

<ci> mw4e68b2da_34ac_4484_9cf5_03f0f6d9abe9 </ci>

</apply>

<apply>

<times/>

<apply>

<plus/>

<ci> mw0c5a9637_94b1_47f0_87f9_8729e75ae8de </ci>

<ci> mw82fe7cf0_c731_49ce_9621_2c502956630b </ci>

</apply>

<apply>

<plus/>

<ci> mw4e68b2da_34ac_4484_9cf5_03f0f6d9abe9 </ci>

<ci> mw0f926ea1_951f_45a7_b0e8_afa94ba266f9 </ci>

</apply>

</apply>

</apply>

</math>

</assignmentRule>

<assignmentRule metaid="repeatedAssignment_mw1a688a3f_337e_4eb8_8285_1755c35d325d" variable="mwd959437e_e966_44f4_a058_a4d962b5fb20">

<math xmlns="http://www.w3.org/1998/Math/MathML">

<apply>

<divide/>

<apply>

<divide/>

<apply>

<times/>

<ci> mw696548be_270b_4aa4_a957_53f3540f254b </ci>

<apply>

<minus/>

<ci> mw98624f9d_a464_456c_9a02_026d96612aff </ci>

```
            <apply>
              <divide/>
              <ci> mw8bc33181_fd96_4636_93eb_674dfce67260 </ci>
              <ci> mw587a43b1_6567_47d8_ab28_db86856d6189 </ci>
            </apply>
          </apply>
        </apply>
        <ci> mw18156bae_84ae_479f_a552_4b556b807e65 </ci>
      </apply>
      <apply>
        <plus/>
        <cn type="integer"> 1 </cn>
        <apply>
          <divide/>
          <ci> mw98624f9d_a464_456c_9a02_026d96612aff </ci>
          <ci> mw18156bae_84ae_479f_a552_4b556b807e65 </ci>
        </apply>
        <apply>
          <divide/>
          <ci> mw8bc33181_fd96_4636_93eb_674dfce67260 </ci>
          <ci> mw6e36548d_68ee_4f99_bdf4_bc0081dec083 </ci>
        </apply>
      </apply>
    </apply>
  </math>
</assignmentRule>
<assignmentRule metaid="repeatedAssignment_mweb33c99f_f573_43c0_90f3_38c78d9b7177" variable="mwc965bd74_dc1e_428c_b262_e88fb94a03c6">
  <math xmlns="http://www.w3.org/1998/Math/MathML">
    <apply>
      <plus/>
```

<apply>
<divide/>
<apply>
<divide/>
<apply>
<times/>
<ci> mw5b9d10ab_7daf_4921_a8cb_86028e0d31e6 </ci>
<apply>
<minus/>
<apply>
<times/>
<ci> mw98624f9d_a464_456c_9a02_026d96612aff </ci>
<ci> mw8bc33181_fd96_4636_93eb_674dfce67260 </ci>
</apply>
<apply>
<divide/>
<ci> mw001a645f_0641_44d7_9a48_6a15ea7e4a51 </ci>
<ci> mwa540938d_712a_46c8_b3d5_bff2c38919f8 </ci>
</apply>
</apply>
</apply>
<apply>
<times/>
<ci> mw2664b98d_62f2_4597_97e0_ac8bf9badc1c </ci>
<ci> mw1ef19eb9_d783_49e0_ab98_1512e9923aff </ci>
</apply>
</apply>
<apply>
<minus/>
<apply>
<plus/>

<apply>
<times/>
<apply>
<plus/>
<cn type="integer"> 1 </cn>
<apply>
<divide/>
<ci> mw001a645f_0641_44d7_9a48_6a15ea7e4a51 </ci>
<ci> mw7d7ad1c0_08d2_429b_89a5_44adb5fc08f2 </ci>
</apply>
</apply>
<apply>
<plus/>
<cn type="integer"> 1 </cn>
<apply>
<divide/>
<ci> mw264fafe4_178f_491d_8866_ba394e95a71b </ci>
<ci> mwed39790d_132b_4762_acf2_55e9d5d068be </ci>
</apply>
</apply>
</apply>
<apply>
<times/>
<apply>
<plus/>
<cn type="integer"> 1 </cn>
<apply>
<divide/>
<ci> mw8bc33181_fd96_4636_93eb_674dfce67260 </ci>
<ci> mw2664b98d_62f2_4597_97e0_ac8bf9badc1c </ci>
</apply>

```
    </apply>
    <apply>
     <plus/>
     <cn type="integer"> 1 </cn>
     <apply>
      <divide/>
      <ci> mw6c1ca071_aba1_4113_ace9_3d429d27bc60 </ci>
      <ci> mw6f8f2f0d_dfd6_4df4_b62b_5cc576a99618 </ci>
     </apply>
    </apply>
    <apply>
     <plus/>
     <cn type="integer"> 1 </cn>
     <apply>
      <divide/>
      <ci> mw98624f9d_a464_456c_9a02_026d96612aff </ci>
      <ci> mw1ef19eb9_d783_49e0_ab98_1512e9923aff </ci>
     </apply>
    </apply>
   </apply>
  </apply>
  <cn type="integer"> 1 </cn>
 </apply>
</apply>
<apply>
 <divide/>
 <apply>
  <divide/>
  <apply>
   <times/>
   <ci> mw16c6ff04_2e36_4d15_bccb_59d7661e10cb </ci>
```

```
    <apply>
      <minus/>
      <apply>
        <times/>
        <ci> mw98624f9d_a464_456c_9a02_026d96612aff </ci>
        <ci> mw8bc33181_fd96_4636_93eb_674dfce67260 </ci>
      </apply>
      <apply>
        <divide/>
        <ci> mw001a645f_0641_44d7_9a48_6a15ea7e4a51 </ci>
        <ci> mwa540938d_712a_46c8_b3d5_bff2c38919f8 </ci>
      </apply>
    </apply>
  </apply>
  <apply>
    <times/>
    <ci> mwe3eb2841_b1eb_4dc4_9c4c_a5d8c5cc75cd </ci>
    <ci> mwc2e367c9_e93b_406b_a5db_e77794a92b22 </ci>
  </apply>
</apply>
<apply>
  <minus/>
  <apply>
    <plus/>
    <apply>
      <times/>
      <apply>
        <plus/>
        <cn type="integer"> 1 </cn>
        <apply>
          <divide/>
```

<ci> mw001a645f_0641_44d7_9a48_6a15ea7e4a51 </ci>
<ci> mw343b1085_b0e0_4e60_8805_60c5ae4ec26b </ci>
</apply>
</apply>
<apply>
<plus/>
<cn type="integer"> 1 </cn>
<apply>
<divide/>
<ci> mw264fafe4_178f_491d_8866_ba394e95a71b </ci>
<ci> mw301fbb5f_9697_492b_8f32_015458d521c5 </ci>
</apply>
</apply>
</apply>
<apply>
<times/>
<apply>
<plus/>
<cn type="integer"> 1 </cn>
<apply>
<divide/>
<ci> mw8bc33181_fd96_4636_93eb_674dfce67260 </ci>
<ci> mwe3eb2841_b1eb_4dc4_9c4c_a5d8c5cc75cd </ci>
</apply>
</apply>
<apply>
<plus/>
<cn type="integer"> 1 </cn>
<apply>
<divide/>
<ci> mw6c1ca071_aba1_4113_ace9_3d429d27bc60 </ci>

```
              <ci> mw2881ad2b_2458_44be_919a_8a2cddc2a765 </ci>
             </apply>
            </apply>
            <apply>
             <plus/>
             <cn type="integer"> 1 </cn>
             <apply>
              <divide/>
              <ci> mw98624f9d_a464_456c_9a02_026d96612aff </ci>
              <ci> mwc2e367c9_e93b_406b_a5db_e77794a92b22 </ci>
             </apply>
            </apply>
           </apply>
          </apply>
          <cn type="integer"> 1 </cn>
         </apply>
        </apply>
       </apply>
      </math>
     </assignmentRule>
     <assignmentRule metaid="repeatedAssignment_mw72bbc029_c078_41cd_b487_9018034e5cee"
variable="mw763d2415_d551_49b6_9ea8_6765ded6374a">
      <math xmlns="http://www.w3.org/1998/Math/MathML">
       <apply>
        <divide/>
        <apply>
         <times/>
         <ci> mw48d7d26d_fe30_4b1e_a4ee_a4eef1cfcb6b </ci>
         <ci> mw001a645f_0641_44d7_9a48_6a15ea7e4a51 </ci>
        </apply>
        <apply>
```

```
            <plus/>
            <ci> mw001a645f_0641_44d7_9a48_6a15ea7e4a51 </ci>
            <apply>
              <times/>
              <ci> mw86a32edf_a962_44c9_adfe_8059b0c656c9 </ci>
              <apply>
                <plus/>
                <cn type="integer"> 1 </cn>
                <apply>
                  <divide/>
                  <ci> mwf1237a2f_b6c2_4f60_aec2_f24eaff9e01a </ci>
                  <ci> mwae1595b9_af51_4b6b_b328_ae530ded7fe0 </ci>
                </apply>
                <apply>
                  <divide/>
                  <ci> mw34d9dd05_1772_4f59_ae50_4d6ef1f63225 </ci>
                  <ci> mw5d188609_730f_4a50_84a3_654056528e7a </ci>
                </apply>
              </apply>
            </apply>
          </apply>
        </apply>
      </math>
    </assignmentRule>
    <assignmentRule metaid="repeatedAssignment_mwf71cb7f1_bbeb_4fa6_b346_cd624d428ad4"
variable="mw51d0f400_64de_478b_9486_a4d146737c8a">
      <math xmlns="http://www.w3.org/1998/Math/MathML">
        <apply>
          <divide/>
          <apply>
            <divide/>
```

```
  <apply>
   <times/>
   <ci> mwc5b9dbbd_d536_4b37_9dd4_99d0328d3414 </ci>
   <apply>
    <minus/>
    <apply>
     <times/>
     <ci> mwf1237a2f_b6c2_4f60_aec2_f24eaff9e01a </ci>
     <ci> mw98624f9d_a464_456c_9a02_026d96612aff </ci>
    </apply>
    <apply>
     <divide/>
     <apply>
      <times/>
      <ci> mwb2da70f3_0970_4374_bdf2_4a3914afa142 </ci>
      <ci> mw6c1ca071_aba1_4113_ace9_3d429d27bc60 </ci>
     </apply>
     <ci> mwc97c27ed_cba8_4dda_8745_bc8e8da8a6b2 </ci>
    </apply>
   </apply>
  </apply>
  <apply>
   <times/>
   <ci> mwf76b7498_870d_4272_aed2_2e9cdad4acd9 </ci>
   <ci> mw1c53df26_ee90_4319_898d_ab1565c18d13 </ci>
  </apply>
 </apply>
 <apply>
  <times/>
  <apply>
   <plus/>
```

```
  <cn type="integer"> 1 </cn>
  <apply>
   <divide/>
   <ci> mwf1237a2f_b6c2_4f60_aec2_f24eaff9e01a </ci>
   <ci> mwf76b7498_870d_4272_aed2_2e9cdad4acd9 </ci>
  </apply>
  <apply>
   <divide/>
   <ci> mw6c1ca071_aba1_4113_ace9_3d429d27bc60 </ci>
   <ci> mw2df7359c_5ca8_46cf_830c_64f2aef66d6a </ci>
  </apply>
 </apply>
 <apply>
  <plus/>
  <cn type="integer"> 1 </cn>
  <apply>
   <divide/>
   <ci> mw98624f9d_a464_456c_9a02_026d96612aff </ci>
   <ci> mw1c53df26_ee90_4319_898d_ab1565c18d13 </ci>
  </apply>
  <apply>
   <divide/>
   <ci> mwb2da70f3_0970_4374_bdf2_4a3914afa142 </ci>
   <ci> mw5f7db393_8e3c_4a77_9cfc_395acd74d218 </ci>
  </apply>
 </apply>
 <apply>
  <plus/>
  <cn type="integer"> 1 </cn>
  <apply>
   <divide/>
```

```
        <ci> mwa5f72579_82b4_4f63_802b_ae5d613cef27 </ci>
        <ci> mw5d1be663_9187_43e2_95e7_5852c1c840a8 </ci>
       </apply>
       <apply>
        <divide/>
        <ci> mw7b7d97f6_2db4_4473_80be_5e4d69e05b59 </ci>
        <ci> mw319586e5_21bf_4aef_ac3a_736836d7e448 </ci>
       </apply>
      </apply>
     </apply>
    </apply>
   </math>
  </assignmentRule>
  <assignmentRule metaid="repeatedAssignment_mw7996cfdf_1bb4_4508_8e52_1f9a13d73690"
variable="mw6e5d7f26_34e6_45a3_9e3b_b8384ba79cd6">
   <math xmlns="http://www.w3.org/1998/Math/MathML">
    <apply>
     <plus/>
     <apply>
      <divide/>
      <apply>
       <divide/>
       <apply>
        <times/>
        <ci> mwc4332dd6_0358_4c1a_ae97_1131d7ea48c2 </ci>
        <apply>
         <minus/>
         <apply>
          <times/>
          <ci> mw8bc33181_fd96_4636_93eb_674dfce67260 </ci>
          <ci> mw6c1ca071_aba1_4113_ace9_3d429d27bc60 </ci>
```

</apply>
<apply>
<divide/>
<ci> mw264fafe4_178f_491d_8866_ba394e95a71b </ci>
<ci> mw08f10a43_f575_462a_b1d4_5b4943f2aecf </ci>
</apply>
</apply>
</apply>
<apply>
<times/>
<ci> mw2664b98d_62f2_4597_97e0_ac8bf9badc1c </ci>
<ci> mw6f8f2f0d_dfd6_4df4_b62b_5cc576a99618 </ci>
</apply>
</apply>
<apply>
<minus/>
<apply>
<plus/>
<apply>
<times/>
<apply>
<plus/>
<cn type="integer"> 1 </cn>
<apply>
<divide/>
<ci> mw001a645f_0641_44d7_9a48_6a15ea7e4a51 </ci>
<ci> mw7d7ad1c0_08d2_429b_89a5_44adb5fc08f2 </ci>
</apply>
</apply>
<apply>
<plus/>

```
    <cn type="integer"> 1 </cn>
    <apply>
      <divide/>
      <ci> mw264fafe4_178f_491d_8866_ba394e95a71b </ci>
      <ci> mwed39790d_132b_4762_acf2_55e9d5d068be </ci>
    </apply>
  </apply>
</apply>
<apply>
  <times/>
  <apply>
    <plus/>
    <cn type="integer"> 1 </cn>
    <apply>
      <divide/>
      <ci> mw8bc33181_fd96_4636_93eb_674dfce67260 </ci>
      <ci> mw2664b98d_62f2_4597_97e0_ac8bf9badc1c </ci>
    </apply>
  </apply>
  <apply>
    <plus/>
    <cn type="integer"> 1 </cn>
    <apply>
      <divide/>
      <ci> mw6c1ca071_aba1_4113_ace9_3d429d27bc60 </ci>
      <ci> mw6f8f2f0d_dfd6_4df4_b62b_5cc576a99618 </ci>
    </apply>
  </apply>
  <apply>
    <plus/>
    <cn type="integer"> 1 </cn>
```

```
          <apply>
            <divide/>
            <ci> mw98624f9d_a464_456c_9a02_026d96612aff </ci>
            <ci> mw1ef19eb9_d783_49e0_ab98_1512e9923aff </ci>
          </apply>
        </apply>
      </apply>
    </apply>
    <cn type="integer"> 1 </cn>
  </apply>
</apply>
<apply>
  <divide/>
  <apply>
    <divide/>
    <apply>
      <times/>
      <ci> mwd9ae7c23_c98b_4e49_8f0c_1c722eaa18d6 </ci>
      <apply>
        <minus/>
        <apply>
          <times/>
          <ci> mw8bc33181_fd96_4636_93eb_674dfce67260 </ci>
          <ci> mw6c1ca071_aba1_4113_ace9_3d429d27bc60 </ci>
        </apply>
        <apply>
          <divide/>
          <ci> mw264fafe4_178f_491d_8866_ba394e95a71b </ci>
          <ci> mw08f10a43_f575_462a_b1d4_5b4943f2aecf </ci>
        </apply>
      </apply>
```

```
  </apply>
  <apply>
   <times/>
   <ci> mwe3eb2841_b1eb_4dc4_9c4c_a5d8c5cc75cd </ci>
   <ci> mw2881ad2b_2458_44be_919a_8a2cddc2a765 </ci>
  </apply>
 </apply>
 <apply>
  <minus/>
  <apply>
   <plus/>
   <apply>
    <times/>
    <apply>
     <plus/>
     <cn type="integer"> 1 </cn>
     <apply>
      <divide/>
      <ci> mw001a645f_0641_44d7_9a48_6a15ea7e4a51 </ci>
      <ci> mw343b1085_b0e0_4e60_8805_60c5ae4ec26b </ci>
     </apply>
    </apply>
    <apply>
     <plus/>
     <cn type="integer"> 1 </cn>
     <apply>
      <divide/>
      <ci> mw264fafe4_178f_491d_8866_ba394e95a71b </ci>
      <ci> mw301fbb5f_9697_492b_8f32_015458d521c5 </ci>
     </apply>
    </apply>
```

```
</apply>
<apply>
 <times/>
 <apply>
  <plus/>
  <cn type="integer"> 1 </cn>
  <apply>
   <divide/>
   <ci> mw8bc33181_fd96_4636_93eb_674dfce67260 </ci>
   <ci> mwe3eb2841_b1eb_4dc4_9c4c_a5d8c5cc75cd </ci>
  </apply>
 </apply>
 <apply>
  <plus/>
  <cn type="integer"> 1 </cn>
  <apply>
   <divide/>
   <ci> mw6c1ca071_aba1_4113_ace9_3d429d27bc60 </ci>
   <ci> mw2881ad2b_2458_44be_919a_8a2cddc2a765 </ci>
  </apply>
 </apply>
 <apply>
  <plus/>
  <cn type="integer"> 1 </cn>
  <apply>
   <divide/>
   <ci> mw98624f9d_a464_456c_9a02_026d96612aff </ci>
   <ci> mwc2e367c9_e93b_406b_a5db_e77794a92b22 </ci>
  </apply>
 </apply>
</apply>
```

```
          </apply>
          <cn type="integer"> 1 </cn>
        </apply>
      </apply>
    </apply>
  </math>
</assignmentRule>
<assignmentRule metaid="repeatedAssignment_mwc36f6b62_9b61_454d_8666_45a43981a902"
variable="mw20e1f4be_43c3_4abc_a168_ee18dd77b40e">
  <math xmlns="http://www.w3.org/1998/Math/MathML">
    <apply>
      <divide/>
      <apply>
        <times/>
        <ci> mw9c962784_a662_43bc_816d_58889af49cc2 </ci>
        <ci> mw264fafe4_178f_491d_8866_ba394e95a71b </ci>
      </apply>
      <apply>
        <plus/>
        <ci> mw264fafe4_178f_491d_8866_ba394e95a71b </ci>
        <apply>
          <times/>
          <ci> mw65b811e9_89c2_407c_a470_8f071a6422d5 </ci>
          <apply>
            <plus/>
            <cn type="integer"> 1 </cn>
            <apply>
              <divide/>
              <ci> mw001a645f_0641_44d7_9a48_6a15ea7e4a51 </ci>
              <ci> mweb78553f_492c_44fc_b049_30f4089f5e52 </ci>
            </apply>
```

</apply>
</apply>
</apply>
</apply>
</math>
</assignmentRule>
<assignmentRule metaid="repeatedAssignment_mw95fba5a4_4ba3_4d29_877e_5e9e32d3856c" variable="mw6d4e3500_0ac5_4333_8ca6_ee6f58266982">
<math xmlns="http://www.w3.org/1998/Math/MathML">
<apply>
<divide/>
<apply>
<divide/>
<apply>
<times/>
<ci> mw520d09f0_2f53_4caa_8bd1_f1066e47098f </ci>
<apply>
<minus/>
<apply>
<times/>
<ci> mw98624f9d_a464_456c_9a02_026d96612aff </ci>
<ci> mwa5f72579_82b4_4f63_802b_ae5d613cef27 </ci>
</apply>
<apply>
<divide/>
<apply>
<times/>
<ci> mw7b7d97f6_2db4_4473_80be_5e4d69e05b59 </ci>
<ci> mwb2da70f3_0970_4374_bdf2_4a3914afa142 </ci>
</apply>
<ci> mwdab67499_dc15_4f7a_b42a_b553d395bdeb </ci>

```
      </apply>
    </apply>
  </apply>
  <apply>
    <times/>
    <ci> mw5d1be663_9187_43e2_95e7_5852c1c840a8 </ci>
    <ci> mw1c53df26_ee90_4319_898d_ab1565c18d13 </ci>
  </apply>
</apply>
<apply>
  <times/>
  <apply>
    <plus/>
    <cn type="integer"> 1 </cn>
    <apply>
      <divide/>
      <ci> mwf1237a2f_b6c2_4f60_aec2_f24eaff9e01a </ci>
      <ci> mwf76b7498_870d_4272_aed2_2e9cdad4acd9 </ci>
    </apply>
    <apply>
      <divide/>
      <ci> mw6c1ca071_aba1_4113_ace9_3d429d27bc60 </ci>
      <ci> mw2df7359c_5ca8_46cf_830c_64f2aef66d6a </ci>
    </apply>
  </apply>
  <apply>
    <plus/>
    <cn type="integer"> 1 </cn>
    <apply>
      <divide/>
      <ci> mw98624f9d_a464_456c_9a02_026d96612aff </ci>
```

```
          <ci> mw1c53df26_ee90_4319_898d_ab1565c18d13 </ci>
         </apply>
         <apply>
          <divide/>
          <ci> mwb2da70f3_0970_4374_bdf2_4a3914afa142 </ci>
          <ci> mw5f7db393_8e3c_4a77_9cfc_395acd74d218 </ci>
         </apply>
        </apply>
        <apply>
         <plus/>
         <cn type="integer"> 1 </cn>
         <apply>
          <divide/>
          <ci> mwa5f72579_82b4_4f63_802b_ae5d613cef27 </ci>
          <ci> mw5d1be663_9187_43e2_95e7_5852c1c840a8 </ci>
         </apply>
         <apply>
          <divide/>
          <ci> mw7b7d97f6_2db4_4473_80be_5e4d69e05b59 </ci>
          <ci> mw319586e5_21bf_4aef_ac3a_736836d7e448 </ci>
         </apply>
        </apply>
       </apply>
      </apply>
     </math>
    </assignmentRule>
    <assignmentRule metaid="repeatedAssignment_mw91fd07a1_e0b0_4504_bccf_23248777036c"
variable="mwf14fae22_a889_4245_b539_05c724efdd58">
     <math xmlns="http://www.w3.org/1998/Math/MathML">
      <apply>
       <divide/>
```

```
<apply>
  <divide/>
  <apply>
    <times/>
    <ci> mw9ac027ed_faba_463f_8575_f0e6d69200bd </ci>
    <apply>
      <minus/>
      <ci> mw7b7d97f6_2db4_4473_80be_5e4d69e05b59 </ci>
      <apply>
        <divide/>
        <ci> mw580d5da5_ec62_4ea2_8f87_a5d617982c02 </ci>
        <ci> mw3143b349_becb_4982_a83f_441e274ee00c </ci>
      </apply>
    </apply>
  </apply>
  <ci> mw576b0b82_5947_49ce_8f54_e086cc631504 </ci>
</apply>
<apply>
  <plus/>
  <cn type="integer"> 1 </cn>
  <apply>
    <divide/>
    <ci> mw580d5da5_ec62_4ea2_8f87_a5d617982c02 </ci>
    <ci> mw53176689_3f53_433a_a683_9edf99a5993d </ci>
  </apply>
  <apply>
    <divide/>
    <ci> mw7b7d97f6_2db4_4473_80be_5e4d69e05b59 </ci>
    <ci> mw576b0b82_5947_49ce_8f54_e086cc631504 </ci>
  </apply>
</apply>
```

</apply>
</math>
</assignmentRule>
<assignmentRule metaid="repeatedAssignment_mw6931f682_c09d_4b91_b309_cc3d78db884f" variable="mwf26c9e95_3acc_4dab_a40f_48aa0b8d9442">
<math xmlns="http://www.w3.org/1998/Math/MathML">
<apply>
<divide/>
<apply>
<divide/>
<apply>
<times/>
<ci> mw634909f8_5cc7_478f_b6b4_3d713718216b </ci>
<apply>
<minus/>
<ci> mwb2da70f3_0970_4374_bdf2_4a3914afa142 </ci>
<apply>
<divide/>
<ci> mw580d5da5_ec62_4ea2_8f87_a5d617982c02 </ci>
<ci> mw9973b455_04bb_4ef2_8bad_e5d9038f2afa </ci>
</apply>
</apply>
</apply>
<ci> mw770450fd_fa82_4c62_8132_cbc289403f10 </ci>
</apply>
<apply>
<plus/>
<cn type="integer"> 1 </cn>
<apply>
<divide/>
<ci> mw580d5da5_ec62_4ea2_8f87_a5d617982c02 </ci>

<ci> mw916809f8_ec94_4f28_aaaa_56521dfe234b </ci>
</apply>
<apply>
<divide/>
<ci> mwb2da70f3_0970_4374_bdf2_4a3914afa142 </ci>
<ci> mw770450fd_fa82_4c62_8132_cbc289403f10 </ci>
</apply>
</apply>
</apply>
</math>
</assignmentRule>
<assignmentRule metaid="repeatedAssignment_mw06794107_3d7b_424b_851a_35600c00e52e" variable="mw7509fc1d_6172_41cf_9e31_08c39accd539">
<math xmlns="http://www.w3.org/1998/Math/MathML">
<apply>
<divide/>
<apply>
<times/>
<ci> mw442d2399_d826_45dc_96d1_1d9a3af70032 </ci>
<ci> mwa083eb39_db7c_4dc7_a1b7_5d06c9120757 </ci>
<ci> mw580d5da5_ec62_4ea2_8f87_a5d617982c02 </ci>
</apply>
<apply>
<times/>
<apply>
<plus/>
<apply>
<times/>
<ci> mwa083eb39_db7c_4dc7_a1b7_5d06c9120757 </ci>
<apply>
<plus/>

<cn type="integer"> 1 </cn>
<apply>
<divide/>
<ci> mw4b092c2e_9b30_46e1_85f7_9bb39d9b10aa </ci>
<ci> mw55d72d42_b4f1_4c2d_8b39_594a18219b62 </ci>
</apply>
</apply>
</apply>
<apply>
<times/>
<ci> mw0d24671c_626c_4751_a2f5_52d895c46b12 </ci>
<apply>
<plus/>
<cn type="integer"> 1 </cn>
<apply>
<divide/>
<ci> mw4b092c2e_9b30_46e1_85f7_9bb39d9b10aa </ci>
<ci> mw353036af_5e46_4c9c_92b2_191591d2c857 </ci>
</apply>
</apply>
</apply>
</apply>
<apply>
<plus/>
<ci> mw580d5da5_ec62_4ea2_8f87_a5d617982c02 </ci>
<apply>
<times/>
<ci> mw51f910d1_180e_4a92_aa3e_17f247a0f4fe </ci>
<apply>
<plus/>
<cn type="integer"> 1 </cn>

```
              <apply>
                <divide/>
                <ci> mw1e1e1695_f297_4f58_906b_5f76ada38e61 </ci>
                <ci> mweb5b46a0_a515_418a_a2c7_5986240a61ff </ci>
              </apply>
              <apply>
                <divide/>
                <ci> mw2603ec03_ce86_4597_b49c_ba6ac6d68e0b </ci>
                <ci> mw62747a3d_d67f_443e_bf91_3c16927d07c9 </ci>
              </apply>
              <apply>
                <divide/>
                <ci> mw34d9dd05_1772_4f59_ae50_4d6ef1f63225 </ci>
                <ci> mwdacf95a3_1ffa_43b0_8a8b_04a0c41ea664 </ci>
              </apply>
            </apply>
          </apply>
        </apply>
      </apply>
    </apply>
  </math>
</assignmentRule>
<assignmentRule
metaid="repeatedAssignment_mwd0374196_2197_4703_b742_b72e9ac130b8"
variable="mwe64ace10_5b0b_414d_9f57_9fd87b03174e">
  <math xmlns="http://www.w3.org/1998/Math/MathML">
    <apply>
      <plus/>
      <apply>
        <divide/>
        <apply>
          <divide/>
```

```
    <apply>
      <times/>
      <ci> mwe11dcb8e_d7c1_423b_bbe4_5da671bd64ca </ci>
      <ci> mwf1237a2f_b6c2_4f60_aec2_f24eaff9e01a </ci>
      <ci> mwa083eb39_db7c_4dc7_a1b7_5d06c9120757 </ci>
    </apply>
    <apply>
      <times/>
      <ci> mw921c626f_edc9_441b_8547_3c7edacfc4e3 </ci>
      <ci> mw9acd4aeb_fdb0_4a5e_833f_e3946722d810 </ci>
    </apply>
  </apply>
  <apply>
    <plus/>
    <cn type="integer"> 1 </cn>
    <apply>
      <times/>
      <apply>
        <divide/>
        <ci> mwa083eb39_db7c_4dc7_a1b7_5d06c9120757 </ci>
        <ci> mw9acd4aeb_fdb0_4a5e_833f_e3946722d810 </ci>
      </apply>
      <apply>
        <divide/>
        <ci> mwf1237a2f_b6c2_4f60_aec2_f24eaff9e01a </ci>
        <ci> mw921c626f_edc9_441b_8547_3c7edacfc4e3 </ci>
      </apply>
    </apply>
  </apply>
</apply>
<apply>
```

```
<divide/>
<apply>
 <divide/>
 <apply>
  <times/>
  <ci> mw4c067e5e_f048_4dc4_bc7d_8e95972e89b1 </ci>
  <ci> mwf1237a2f_b6c2_4f60_aec2_f24eaff9e01a </ci>
  <ci> mwa083eb39_db7c_4dc7_a1b7_5d06c9120757 </ci>
 </apply>
 <apply>
  <times/>
  <ci> mw5d3b7d4f_cb37_4189_808f_9b316593f3fd </ci>
  <ci> mw4f29d2d9_0857_4db2_880a_535c67780929 </ci>
 </apply>
</apply>
<apply>
 <plus/>
 <cn type="integer"> 1 </cn>
 <apply>
  <times/>
  <apply>
   <divide/>
   <ci> mwa083eb39_db7c_4dc7_a1b7_5d06c9120757 </ci>
   <ci> mw4f29d2d9_0857_4db2_880a_535c67780929 </ci>
  </apply>
  <apply>
   <divide/>
   <ci> mwf1237a2f_b6c2_4f60_aec2_f24eaff9e01a </ci>
   <ci> mw5d3b7d4f_cb37_4189_808f_9b316593f3fd </ci>
  </apply>
 </apply>
```

```
      </apply>
     </apply>
    </apply>
   </math>
  </assignmentRule>
  <assignmentRule metaid="repeatedAssignment_mw30c9e18e_e3b9_410e_9175_93f5822e5df8" variable="mw31b2d210_181c_4d37_8e6f_dea686ada228">
   <math xmlns="http://www.w3.org/1998/Math/MathML">
    <apply>
     <plus/>
     <apply>
      <divide/>
      <apply>
       <times/>
       <ci> mw4354fd87_eec3_45ff_ba09_8e66f6cec0d4 </ci>
       <apply>
        <minus/>
        <ci> mw1e1e1695_f297_4f58_906b_5f76ada38e61 </ci>
        <apply>
         <divide/>
         <ci> mwcd2b43d1_7092_45b9_af63_c926207ad771 </ci>
         <ci> mwfa3dac90_b557_4ea0_950c_b2e6cea1f624 </ci>
        </apply>
       </apply>
      </apply>
      <apply>
       <times/>
       <ci> mw407bee91_c458_4b42_ad72_7dea8c0f84b8 </ci>
       <apply>
        <plus/>
        <cn type="integer"> 1 </cn>
```

```
   <apply>
    <divide/>
    <ci> mw1e1e1695_f297_4f58_906b_5f76ada38e61 </ci>
    <ci> mw407bee91_c458_4b42_ad72_7dea8c0f84b8 </ci>
   </apply>
   <apply>
    <divide/>
    <ci> mwcd2b43d1_7092_45b9_af63_c926207ad771 </ci>
    <ci> mw2091e1f5_0cd3_4d23_9dcf_9f54ae671686 </ci>
   </apply>
  </apply>
 </apply>
</apply>
<apply>
 <divide/>
 <apply>
  <times/>
  <ci> mw9e3b0877_c7fe_4a60_886f_4c0b97f63ef9 </ci>
  <apply>
   <minus/>
   <ci> mw1e1e1695_f297_4f58_906b_5f76ada38e61 </ci>
   <apply>
    <divide/>
    <ci> mwcd2b43d1_7092_45b9_af63_c926207ad771 </ci>
    <ci> mwfa3dac90_b557_4ea0_950c_b2e6cea1f624 </ci>
   </apply>
  </apply>
 </apply>
 <apply>
  <times/>
  <ci> mw099e3275_3aed_4b7c_abd7_1d978c4681d1 </ci>
```

```
    <apply>
      <plus/>
      <cn type="integer"> 1 </cn>
      <apply>
        <divide/>
        <ci> mw1e1e1695_f297_4f58_906b_5f76ada38e61 </ci>
        <ci> mw099e3275_3aed_4b7c_abd7_1d978c4681d1 </ci>
      </apply>
      <apply>
        <divide/>
        <ci> mwcd2b43d1_7092_45b9_af63_c926207ad771 </ci>
        <ci> mw51232471_96c5_44a5_9906_91a4af9717da </ci>
      </apply>
    </apply>
  </apply>
</apply>
<apply>
  <divide/>
  <apply>
    <times/>
    <ci> mwa466c9c9_3297_437c_baed_0dce17c19743 </ci>
    <apply>
      <minus/>
      <ci> mw1e1e1695_f297_4f58_906b_5f76ada38e61 </ci>
      <apply>
        <divide/>
        <ci> mwcd2b43d1_7092_45b9_af63_c926207ad771 </ci>
        <ci> mwfa3dac90_b557_4ea0_950c_b2e6cea1f624 </ci>
      </apply>
    </apply>
  </apply>
```

```
          <apply>
            <times/>
            <ci> mwefaeb6d1_a2ae_41cb_b478_f28cb1be6a7c </ci>
            <apply>
              <plus/>
              <cn type="integer"> 1 </cn>
              <apply>
                <divide/>
                <ci> mw1e1e1695_f297_4f58_906b_5f76ada38e61 </ci>
                <ci> mwefaeb6d1_a2ae_41cb_b478_f28cb1be6a7c </ci>
              </apply>
              <apply>
                <divide/>
                <ci> mwcd2b43d1_7092_45b9_af63_c926207ad771 </ci>
                <ci> mw93985682_472b_4e3b_895b_705c1b0929b3 </ci>
              </apply>
            </apply>
          </apply>
        </apply>
      </apply>
    </math>
  </assignmentRule>
  <assignmentRule metaid="repeatedAssignment_mw00b47794_57d7_44c4_a0f8_f9d60c938d25"
variable="mwfb0df553_4b52_4b95_9154_bb66087e5593">
    <math xmlns="http://www.w3.org/1998/Math/MathML">
      <apply>
        <divide/>
        <apply>
          <times/>
          <ci> mw1560b05b_911d_4737_8105_b669df533ad3 </ci>
          <apply>
```

```
        <minus/>
        <ci> mwcd2b43d1_7092_45b9_af63_c926207ad771 </ci>
        <apply>
          <divide/>
          <ci> mwc3b39320_8ede_4d49_ba08_c569fbe1474d </ci>
          <ci> mw60d16eba_0bba_4387_94f0_ddda983743a2 </ci>
        </apply>
      </apply>
    </apply>
    <apply>
      <times/>
      <ci> mw48edc399_e14c_4e4e_8fd5_27aa7a43e91a </ci>
      <apply>
        <plus/>
        <cn type="integer"> 1 </cn>
        <apply>
          <divide/>
          <ci> mwcd2b43d1_7092_45b9_af63_c926207ad771 </ci>
          <ci> mw48edc399_e14c_4e4e_8fd5_27aa7a43e91a </ci>
        </apply>
        <apply>
          <divide/>
          <ci> mwc3b39320_8ede_4d49_ba08_c569fbe1474d </ci>
          <ci> mw28cc925f_1ec1_40e8_a64d_bacb34b7b472 </ci>
        </apply>
      </apply>
    </apply>
  </apply>
</math>
</assignmentRule>
```

```
<assignmentRule metaid="repeatedAssignment_mwb7307a8c_5fe2_426f_9a7e_6e8383a28f04" variable="mw80bf3a05_9be7_4dfa_baec_a5142f07eb3b">
  <math xmlns="http://www.w3.org/1998/Math/MathML">
    <apply>
      <divide/>
      <apply>
        <times/>
        <ci> mw30cb04f1_447a_49ab_9566_af33f6c68f02 </ci>
        <ci> mw98624f9d_a464_456c_9a02_026d96612aff </ci>
        <ci> mwf5dc5c05_4d36_4ea4_8ee0_6a1cfd2ae49b </ci>
      </apply>
      <apply>
        <times/>
        <apply>
          <plus/>
          <cn type="integer"> 1 </cn>
          <apply>
            <divide/>
            <ci> mw98624f9d_a464_456c_9a02_026d96612aff </ci>
            <ci> mw2b15c7a2_60bd_4539_bbca_ea2887c83c7e </ci>
          </apply>
          <apply>
            <divide/>
            <ci> mw1e1e1695_f297_4f58_906b_5f76ada38e61 </ci>
            <ci> mw66c149b7_107b_4f1e_8ba3_e998a2eb4341 </ci>
          </apply>
        </apply>
        <apply>
          <plus/>
          <cn type="integer"> 1 </cn>
          <apply>
```

<divide/>
<ci> mwf5dc5c05_4d36_4ea4_8ee0_6a1cfd2ae49b </ci>
<ci> mw42d232ce_870c_4c6c_80e1_b80db63c24dc </ci>
</apply>
<apply>
<divide/>
<ci> mw4e68b2da_34ac_4484_9cf5_03f0f6d9abe9 </ci>
<ci> mw3f587528_6a1e_45cf_85a1_c83170fa3db2 </ci>
</apply>
</apply>
</apply>
</apply>
</math>
</assignmentRule>
<assignmentRule metaid="repeatedAssignment_mwdbf0afc4_646d_4ec5_8657_03c2e797160b" variable="mw04299979_e245_4206_9f43_d70b28685aeb">
<math xmlns="http://www.w3.org/1998/Math/MathML">
<apply>
<divide/>
<apply>
<times/>
<ci> mw10b1b1bc_dce8_475d_b4fa_c06a66547e4e </ci>
<ci> mwc3b39320_8ede_4d49_ba08_c569fbe1474d </ci>
</apply>
<apply>
<plus/>
<ci> mw75e9f4a7_4ba0_493e_b8ce_844e0daf2309 </ci>
<ci> mwc3b39320_8ede_4d49_ba08_c569fbe1474d </ci>
</apply>
</apply>
</math>

```
      </assignmentRule>
      <assignmentRule metaid="repeatedAssignment_mwefacd0a3_90b7_4c05_990c_887e4e612033" variable="mw260a4232_836b_47e0_9e60_c60b15f0ecd5">
        <math xmlns="http://www.w3.org/1998/Math/MathML">
          <apply>
            <divide/>
            <apply>
              <times/>
              <ci> mw2f7b3fed_abd8_460f_aa2b_6034d9dc3ffc </ci>
              <ci> mwc5d2bfb5_b299_4e35_b8b7_6f7ff0ce2a5a </ci>
            </apply>
            <apply>
              <plus/>
              <ci> mw88fca50d_8086_41ed_9f8b_bfce18be9463 </ci>
              <ci> mwc5d2bfb5_b299_4e35_b8b7_6f7ff0ce2a5a </ci>
            </apply>
          </apply>
        </math>
      </assignmentRule>
      <assignmentRule metaid="repeatedAssignment_mwf6b57a79_64dd_437a_b8af_ef25b06e8db5" variable="mw34c708ab_f8fd_4daf_8e29_2096cb7e1563">
        <math xmlns="http://www.w3.org/1998/Math/MathML">
          <apply>
            <divide/>
            <apply>
              <times/>
              <ci> mw7815c29b_f589_487d_8abf_8c69866a16bf </ci>
              <ci> mw98624f9d_a464_456c_9a02_026d96612aff </ci>
            </apply>
            <apply>
              <plus/>
              <ci> mw0e500944_5cfc_4224_907b_dd0573a17387 </ci>
```

```
<ci> mw98624f9d_a464_456c_9a02_026d96612aff </ci>
</apply>
</apply>
</math>
</assignmentRule>
<assignmentRule metaid="repeatedAssignment_mwe7f1786f_5186_4b39_9fbc_b9b9c6656b7c" variable="mwf634a4ad_468b_4f81_a966_d081677b2bd6">
<math xmlns="http://www.w3.org/1998/Math/MathML">
<apply>
<divide/>
<apply>
<times/>
<ci> mw94c1a2ea_8406_444a_beaf_3ae6b855d7b5 </ci>
<ci> mw6c1ca071_aba1_4113_ace9_3d429d27bc60 </ci>
</apply>
<apply>
<plus/>
<ci> mwd6c6584f_18c7_4c2e_928b_bc7cebf26f01 </ci>
<ci> mw6c1ca071_aba1_4113_ace9_3d429d27bc60 </ci>
</apply>
</apply>
</math>
</assignmentRule>
<assignmentRule metaid="repeatedAssignment_mwc2fba0a6_09ce_400c_9d69_7d485dc38c78" variable="mwa912d5b8_71c9_4d1b_ae93_7fe4563570ea">
<math xmlns="http://www.w3.org/1998/Math/MathML">
<apply>
<divide/>
<apply>
<times/>
<ci> mwcb26c99a_ae43_4c60_ae4d_a62a4a7bf1d1 </ci>
<ci> mw7b7d97f6_2db4_4473_80be_5e4d69e05b59 </ci>
```

```
      </apply>
      <apply>
        <plus/>
        <ci> mw73931537_4826_46e8_9efc_5a58ea02c555 </ci>
        <ci> mw7b7d97f6_2db4_4473_80be_5e4d69e05b59 </ci>
      </apply>
    </apply>
  </math>
</assignmentRule>
<assignmentRule metaid="repeatedAssignment_mwd056abdf_7591_40bc_95ed_7aed4facf379"
variable="mwc153f931_c15d_4f00_915f_d828490f3e0c">
  <math xmlns="http://www.w3.org/1998/Math/MathML">
    <apply>
      <plus/>
      <apply>
        <divide/>
        <apply>
          <divide/>
          <apply>
            <times/>
            <ci> mw14672e46_52f5_4ab7_bff1_4b749b587f1d </ci>
            <apply>
              <minus/>
              <ci> mwf1237a2f_b6c2_4f60_aec2_f24eaff9e01a </ci>
              <apply>
                <divide/>
                <apply>
                  <times/>
                  <ci> mwe1915c23_bc54_4444_a6b6_afb9f835d8cf </ci>
                  <ci> mw6c1ca071_aba1_4113_ace9_3d429d27bc60 </ci>
                </apply>
```

```
    <ci> mwac1b2180_f1af_4819_9233_94af7fae5fe8 </ci>
   </apply>
  </apply>
 </apply>
 <ci> mw95025ff8_a096_4b4e_b30a_c86490520d3c </ci>
</apply>
<apply>
 <times/>
 <apply>
  <plus/>
  <cn type="integer"> 1 </cn>
  <apply>
   <divide/>
   <ci> mwf1237a2f_b6c2_4f60_aec2_f24eaff9e01a </ci>
   <ci> mw95025ff8_a096_4b4e_b30a_c86490520d3c </ci>
  </apply>
  <apply>
   <divide/>
   <ci> mw6c1ca071_aba1_4113_ace9_3d429d27bc60 </ci>
   <ci> mw35d79455_d586_4f7e_b53d_b9b05403c18f </ci>
  </apply>
 </apply>
 <apply>
  <plus/>
  <cn type="integer"> 1 </cn>
  <apply>
   <divide/>
   <ci> mwe1915c23_bc54_4444_a6b6_afb9f835d8cf </ci>
   <ci> mw29daf52a_3537_43f2_b889_86b8d087fab9 </ci>
  </apply>
 </apply>
```

<apply>
<plus/>
<cn type="integer"> 1 </cn>
<apply>
<divide/>
<ci> mwb2da70f3_0970_4374_bdf2_4a3914afa142 </ci>
<ci> mwcc132bf2_7c6b_4cbb_b3e3_caddab999b30 </ci>
</apply>
<apply>
<divide/>
<ci> mw98624f9d_a464_456c_9a02_026d96612aff </ci>
<ci> mw4231ba04_0406_4368_925a_599001509b3f </ci>
</apply>
</apply>
</apply>
</apply>
<apply>
<divide/>
<apply>
<divide/>
<apply>
<times/>
<ci> mwe6cc0f80_5d7e_478d_8df3_785b611461a8 </ci>
<apply>
<minus/>
<ci> mwb2da70f3_0970_4374_bdf2_4a3914afa142 </ci>
<apply>
<divide/>
<apply>
<times/>
<ci> mwe1915c23_bc54_4444_a6b6_afb9f835d8cf </ci>

<ci> mw98624f9d_a464_456c_9a02_026d96612aff </ci>
</apply>
<ci> mwb043257b_34eb_45c4_b4bd_7fadfb6e19c8 </ci>
</apply>
</apply>
</apply>
<ci> mw94c699e4_fed2_455f_9f56_63394e10e6aa </ci>
</apply>
<apply>
<times/>
<apply>
<plus/>
<cn type="integer"> 1 </cn>
<apply>
<divide/>
<ci> mwf1237a2f_b6c2_4f60_aec2_f24eaff9e01a </ci>
<ci> mw08464d63_135a_4871_9108_9a430e7da3c9 </ci>
</apply>
<apply>
<divide/>
<ci> mw6c1ca071_aba1_4113_ace9_3d429d27bc60 </ci>
<ci> mwf1cc2080_281e_420a_bea7_9f1ace706f8b </ci>
</apply>
</apply>
<apply>
<plus/>
<cn type="integer"> 1 </cn>
<apply>
<divide/>
<ci> mwe1915c23_bc54_4444_a6b6_afb9f835d8cf </ci>
<ci> mw07fd01bf_c788_41e8_ad93_f9663c71e731 </ci>

</apply>
</apply>
<apply>
<plus/>
<cn type="integer"> 1 </cn>
<apply>
<divide/>
<ci> mwb2da70f3_0970_4374_bdf2_4a3914afa142 </ci>
<ci> mw94c699e4_fed2_455f_9f56_63394e10e6aa </ci>
</apply>
<apply>
<divide/>
<ci> mw98624f9d_a464_456c_9a02_026d96612aff </ci>
<ci> mw4925fe87_c38b_4c63_b257_76e6a00ba813 </ci>
</apply>
</apply>
</apply>
</apply>
</apply>
</math>
</assignmentRule>
<assignmentRule metaid="repeatedAssignment_mw692b2dca_4533_40b2_afff_7080a8c72368" variable="mw6679025e_faa6_4ddb_9090_6b6b0c957d8c">
<math xmlns="http://www.w3.org/1998/Math/MathML">
<apply>
<plus/>
<apply>
<divide/>
<apply>
<divide/>
<apply>

```
    <times/>
    <ci> mwb9289074_0186_4371_aed9_7470cba640dc </ci>
    <apply>
      <minus/>
      <ci> mwf1237a2f_b6c2_4f60_aec2_f24eaff9e01a </ci>
      <apply>
        <divide/>
        <apply>
          <times/>
          <ci> mwe1915c23_bc54_4444_a6b6_afb9f835d8cf </ci>
          <ci> mw6c1ca071_aba1_4113_ace9_3d429d27bc60 </ci>
        </apply>
        <ci> mwac1b2180_f1af_4819_9233_94af7fae5fe8 </ci>
      </apply>
    </apply>
  </apply>
  <ci> mw235e9584_299a_49b0_917e_b62cb998dc8e </ci>
</apply>
<apply>
  <times/>
  <apply>
    <plus/>
    <cn type="integer"> 1 </cn>
    <apply>
      <divide/>
      <ci> mwf1237a2f_b6c2_4f60_aec2_f24eaff9e01a </ci>
      <ci> mw235e9584_299a_49b0_917e_b62cb998dc8e </ci>
    </apply>
    <apply>
      <divide/>
      <ci> mw6c1ca071_aba1_4113_ace9_3d429d27bc60 </ci>
```

```
        <ci> mw1b9a6d90_0834_45b2_80c3_894c8f3806df </ci>
      </apply>
    </apply>
    <apply>
      <plus/>
      <cn type="integer"> 1 </cn>
      <apply>
        <divide/>
        <ci> mwe1915c23_bc54_4444_a6b6_afb9f835d8cf </ci>
        <ci> mwffdd6cd4_59fc_4211_a076_bf7bccbb2861 </ci>
      </apply>
    </apply>
    <apply>
      <plus/>
      <cn type="integer"> 1 </cn>
      <apply>
        <divide/>
        <ci> mwb2da70f3_0970_4374_bdf2_4a3914afa142 </ci>
        <ci> mwe2c4c957_b4a0_41f4_aacc_9a73304177bf </ci>
      </apply>
      <apply>
        <divide/>
        <ci> mw98624f9d_a464_456c_9a02_026d96612aff </ci>
        <ci> mwee148dba_6936_4ff5_98e9_3fafc08bf46c </ci>
      </apply>
    </apply>
  </apply>
</apply>
<apply>
  <divide/>
  <apply>
```

<divide/>
<apply>
<times/>
<ci> mwbfebafa8_c3ee_48cc_843e_9a4bad33bfb3 </ci>
<apply>
<minus/>
<ci> mwb2da70f3_0970_4374_bdf2_4a3914afa142 </ci>
<apply>
<divide/>
<apply>
<times/>
<ci> mwe1915c23_bc54_4444_a6b6_afb9f835d8cf </ci>
<ci> mw98624f9d_a464_456c_9a02_026d96612aff </ci>
</apply>
<ci> mwb043257b_34eb_45c4_b4bd_7fadfb6e19c8 </ci>
</apply>
</apply>
</apply>
<ci> mw6aa54137_d905_4ffa_a29c_b192c4901c8f </ci>
</apply>
<apply>
<times/>
<apply>
<plus/>
<cn type="integer"> 1 </cn>
<apply>
<divide/>
<ci> mwf1237a2f_b6c2_4f60_aec2_f24eaff9e01a </ci>
<ci> mw04c1dd77_705d_42f9_a0ad_169227a654aa </ci>
</apply>
<apply>

<divide/>
<ci> mw6c1ca071_aba1_4113_ace9_3d429d27bc60 </ci>
<ci> mw36675533_f3f1_443f_a00a_e9f3ab7b4e43 </ci>
</apply>
</apply>
<apply>
<plus/>
<cn type="integer"> 1 </cn>
<apply>
<divide/>
<ci> mwe1915c23_bc54_4444_a6b6_afb9f835d8cf </ci>
<ci> mw48d5b78e_f2bf_4cae_92f9_9b4b7e011e9a </ci>
</apply>
</apply>
<apply>
<plus/>
<cn type="integer"> 1 </cn>
<apply>
<divide/>
<ci> mwb2da70f3_0970_4374_bdf2_4a3914afa142 </ci>
<ci> mw6aa54137_d905_4ffa_a29c_b192c4901c8f </ci>
</apply>
<apply>
<divide/>
<ci> mw98624f9d_a464_456c_9a02_026d96612aff </ci>
<ci> mwc941b16b_73fe_48ea_828c_7d4127c52bdc </ci>
</apply>
</apply>
</apply>
</apply>
</apply>

</math>

</assignmentRule>

<assignmentRule metaid="repeatedAssignment_mw3f3013df_5b11_4aae_87a1_bfd8ac4ae3cd" variable="mwce034c6e_3ba3_4a8c_bd2f_94b469c74eeb">

<math xmlns="http://www.w3.org/1998/Math/MathML">

<apply>

<divide/>

<apply>

<times/>

<ci> mw9726ca8c_0c0e_4d16_bae1_3d731a5408aa </ci>

<ci> mwc5d2bfb5_b299_4e35_b8b7_6f7ff0ce2a5a </ci>

</apply>

<apply>

<times/>

<ci> mw2db94c60_e05f_4408_bc54_54fb9f9f7d42 </ci>

<apply>

<plus/>

<cn type="integer"> 1 </cn>

<apply>

<divide/>

<ci> mwc5d2bfb5_b299_4e35_b8b7_6f7ff0ce2a5a </ci>

<ci> mw2db94c60_e05f_4408_bc54_54fb9f9f7d42 </ci>

</apply>

<apply>

<divide/>

<ci> mw630d7a9f_d907_496c_b750_6c23afbb4658 </ci>

<ci> mw361ddcb4_26fd_439c_b85e_c582a34f84b1 </ci>

</apply>

</apply>

</apply>

</apply>

</math>

</assignmentRule>

<assignmentRule metaid="repeatedAssignment_mwc8822c75_d225_475c_a5c4_0fd6d85c3110" variable="mw121ef383_adea_44fd_af43_a9fef1ca3455">

<math xmlns="http://www.w3.org/1998/Math/MathML">

<apply>

<plus/>

<ci> mwdc722523_d3fc_41a7_99f4_eea898e1ed4f </ci>

<ci> mwacd9e1e6_f4cc_4f7e_9a51_0a8732e6ddde </ci>

<ci> mw2ac0cdfb_0f6b_484c_b7b5_41cfbdace9b8 </ci>

<ci> mw6c2cc5c8_f456_40b7_929f_e3d9e37f99b2 </ci>

<ci> mwa94479e8_4e60_4fc5_9681_8c8e54d33e74 </ci>

<ci> mwc1711fc7_ff82_413c_8a9c_248c948df9bc </ci>

<ci> mw3bf50b2f_519c_495b_b72a_b0753138f38d </ci>

<ci> mw1b5d434c_13de_4b56_bd7d_627742a6ceae </ci>

<ci> mw6a9b239b_a9cc_43c8_b083_35f106e0485d </ci>

</apply>

</math>

</assignmentRule>

<assignmentRule metaid="repeatedAssignment_mw7e9a160a_9921_40c1_af91_72bd4bf2165e" variable="mw76a409bd_32d8_438e_8ec2_80106d26e956">

<math xmlns="http://www.w3.org/1998/Math/MathML">

<apply>

<divide/>

<apply>

<times/>

<ci> mwd12025b7_9ac7_4a08_ae36_005fa0a2ad82 </ci>

<ci> mw1e1e1695_f297_4f58_906b_5f76ada38e61 </ci>

</apply>

<apply>

<plus/>

<ci> mw09424d24_2a0c_4c48_99fd_174b17fae4c6 </ci>

<ci> mw1e1e1695_f297_4f58_906b_5f76ada38e61 </ci>

</apply>

</apply>

</math>

</assignmentRule>

<assignmentRule metaid="repeatedAssignment_mw874f83eb_4685_4d33_a36c_8fb5549b054b" variable="mw22183e88_4ec6_4ae3_9e1d_0e66b0c75c7d">

<math xmlns="http://www.w3.org/1998/Math/MathML">

<apply>

<minus/>

<ci> mw763d2415_d551_49b6_9ea8_6765ded6374a </ci>

<ci> mwe64ace10_5b0b_414d_9f57_9fd87b03174e </ci>

</apply>

</math>

</assignmentRule>

<assignmentRule metaid="repeatedAssignment_mw619a035c_0c60_40c4_8f4d_ec2224b02eb1" variable="mwee5b965c_bc88_4930_9408_a56b03f8777d">

<math xmlns="http://www.w3.org/1998/Math/MathML">

<apply>

<divide/>

<apply>

<divide/>

<apply>

<times/>

<ci> mwc689e60c_5ebe_4df0_9311_a4fa4858f41d </ci>

<ci> mw630d7a9f_d907_496c_b750_6c23afbb4658 </ci>

</apply>

<apply>

<plus/>

<ci> mw630d7a9f_d907_496c_b750_6c23afbb4658 </ci>

<ci> mw68578e28_5e7b_43b7_a251_4585ec996637 </ci>

</apply>

```
          </apply>
          <apply>
            <plus/>
            <cn type="integer"> 1 </cn>
            <apply>
              <divide/>
              <ci> mw01be9f1c_a2a5_492c_bcd3_f7f50fab164f </ci>
              <ci> mw3cd3e9a1_dbe4_41c7_870c_fb4e65d18298 </ci>
            </apply>
          </apply>
        </apply>
      </math>
    </assignmentRule>
    <assignmentRule metaid="repeatedAssignment_mwee2b4d08_d834_4669_a629_9fb45c78adc7" variable="mw4237c9a8_dcd7_4b30_8ef0_8cecd69c9694">
      <math xmlns="http://www.w3.org/1998/Math/MathML">
        <apply>
          <divide/>
          <apply>
            <times/>
            <ci> mw1dc7ae44_7d88_420e_8084_bc013cd8b1dc </ci>
            <apply>
              <minus/>
              <apply>
                <times/>
                <ci> mw6d3e7fb2_fbcb_4028_ae3c_377e5ec0c707 </ci>
                <ci> mwf1237a2f_b6c2_4f60_aec2_f24eaff9e01a </ci>
              </apply>
              <ci> mwc5d2bfb5_b299_4e35_b8b7_6f7ff0ce2a5a </ci>
            </apply>
          </apply>
```

```
          <apply>
            <plus/>
            <ci> mwf1237a2f_b6c2_4f60_aec2_f24eaff9e01a </ci>
            <ci> mwc5d2bfb5_b299_4e35_b8b7_6f7ff0ce2a5a </ci>
          </apply>
        </apply>
      </math>
    </assignmentRule>
    <assignmentRule metaid="repeatedAssignment_mw751cb985_7c4e_4b22_a2a8_421a224e9410"
variable="mw5682e6fc_33b0_4c3f_83a6_0027e7f9efcc">
      <math xmlns="http://www.w3.org/1998/Math/MathML">
        <apply>
          <times/>
          <ci> mwc6aa212e_7e00_4697_af9f_140f4ef235ea </ci>
          <ci> mw5c9d3afc_2037_42d9_a8c6_e341b406cf37 </ci>
        </apply>
      </math>
    </assignmentRule>
    <assignmentRule metaid="repeatedAssignment_mw055343c2_5c27_40fa_8cfc_cafe4ccd8dda"
variable="mwb4d18f64_6a6f_4ee2_9e60_e95453fd193a">
      <math xmlns="http://www.w3.org/1998/Math/MathML">
        <apply>
          <divide/>
          <apply>
            <divide/>
            <apply>
              <times/>
              <ci> mwe11dcb8e_d7c1_423b_bbe4_5da671bd64ca </ci>
              <ci> mwf1237a2f_b6c2_4f60_aec2_f24eaff9e01a </ci>
              <ci> mwa083eb39_db7c_4dc7_a1b7_5d06c9120757 </ci>
            </apply>
            <apply>
```

```
          <times/>
          <ci> mw921c626f_edc9_441b_8547_3c7edacfc4e3 </ci>
          <ci> mw9acd4aeb_fdb0_4a5e_833f_e3946722d810 </ci>
        </apply>
      </apply>
      <apply>
        <plus/>
        <cn type="integer"> 1 </cn>
        <apply>
          <times/>
          <apply>
            <divide/>
            <ci> mwa083eb39_db7c_4dc7_a1b7_5d06c9120757 </ci>
            <ci> mw9acd4aeb_fdb0_4a5e_833f_e3946722d810 </ci>
          </apply>
          <apply>
            <divide/>
            <ci> mwf1237a2f_b6c2_4f60_aec2_f24eaff9e01a </ci>
            <ci> mw921c626f_edc9_441b_8547_3c7edacfc4e3 </ci>
          </apply>
        </apply>
      </apply>
    </apply>
  </math>
</assignmentRule>
<assignmentRule metaid="repeatedAssignment_mwe819d945_b3f7_44be_82d5_815656304c46"
variable="mw5d306209_83b3_4fd3_b2d3_3d0821016185">
  <math xmlns="http://www.w3.org/1998/Math/MathML">
    <apply>
      <divide/>
      <apply>
```

<divide/>
<apply>
<times/>
<ci> mw4c067e5e_f048_4dc4_bc7d_8e95972e89b1 </ci>
<ci> mwf1237a2f_b6c2_4f60_aec2_f24eaff9e01a </ci>
<ci> mwa083eb39_db7c_4dc7_a1b7_5d06c9120757 </ci>
</apply>
<apply>
<times/>
<ci> mw5d3b7d4f_cb37_4189_808f_9b316593f3fd </ci>
<ci> mw4f29d2d9_0857_4db2_880a_535c67780929 </ci>
</apply>
</apply>
<apply>
<plus/>
<cn type="integer"> 1 </cn>
<apply>
<times/>
<apply>
<divide/>
<ci> mwa083eb39_db7c_4dc7_a1b7_5d06c9120757 </ci>
<ci> mw4f29d2d9_0857_4db2_880a_535c67780929 </ci>
</apply>
<apply>
<divide/>
<ci> mwf1237a2f_b6c2_4f60_aec2_f24eaff9e01a </ci>
<ci> mw5d3b7d4f_cb37_4189_808f_9b316593f3fd </ci>
</apply>
</apply>
</apply>
</apply>

</math>

</assignmentRule>

<assignmentRule metaid="repeatedAssignment_mwa64829f2_9e3b_4286_8ca9_fb2a2542b067" variable="mw9c6265f4_0d84_4317_85e3_1397b925dd6b">

<math xmlns="http://www.w3.org/1998/Math/MathML">

<apply>

<divide/>

<apply>

<divide/>

<apply>

<times/>

<ci> mw5b9d10ab_7daf_4921_a8cb_86028e0d31e6 </ci>

<apply>

<minus/>

<apply>

<times/>

<ci> mw98624f9d_a464_456c_9a02_026d96612aff </ci>

<ci> mw8bc33181_fd96_4636_93eb_674dfce67260 </ci>

</apply>

<apply>

<divide/>

<ci> mw001a645f_0641_44d7_9a48_6a15ea7e4a51 </ci>

<ci> mwa540938d_712a_46c8_b3d5_bff2c38919f8 </ci>

</apply>

</apply>

</apply>

<apply>

<times/>

<ci> mw2664b98d_62f2_4597_97e0_ac8bf9badc1c </ci>

<ci> mw1ef19eb9_d783_49e0_ab98_1512e9923aff </ci>

</apply>

```
</apply>
<apply>
 <minus/>
 <apply>
  <plus/>
  <apply>
   <times/>
   <apply>
    <plus/>
    <cn type="integer"> 1 </cn>
    <apply>
     <divide/>
     <ci> mw001a645f_0641_44d7_9a48_6a15ea7e4a51 </ci>
     <ci> mw7d7ad1c0_08d2_429b_89a5_44adb5fc08f2 </ci>
    </apply>
   </apply>
   <apply>
    <plus/>
    <cn type="integer"> 1 </cn>
    <apply>
     <divide/>
     <ci> mw264fafe4_178f_491d_8866_ba394e95a71b </ci>
     <ci> mwed39790d_132b_4762_acf2_55e9d5d068be </ci>
    </apply>
   </apply>
  </apply>
  <apply>
   <times/>
   <apply>
    <plus/>
    <cn type="integer"> 1 </cn>
```

```
          <apply>
            <divide/>
            <ci> mw8bc33181_fd96_4636_93eb_674dfce67260 </ci>
            <ci> mw2664b98d_62f2_4597_97e0_ac8bf9badc1c </ci>
          </apply>
        </apply>
        <apply>
          <plus/>
          <cn type="integer"> 1 </cn>
          <apply>
            <divide/>
            <ci> mw6c1ca071_aba1_4113_ace9_3d429d27bc60 </ci>
            <ci> mw6f8f2f0d_dfd6_4df4_b62b_5cc576a99618 </ci>
          </apply>
        </apply>
        <apply>
          <plus/>
          <cn type="integer"> 1 </cn>
          <apply>
            <divide/>
            <ci> mw98624f9d_a464_456c_9a02_026d96612aff </ci>
            <ci> mw1ef19eb9_d783_49e0_ab98_1512e9923aff </ci>
          </apply>
        </apply>
      </apply>
    </apply>
    <cn type="integer"> 1 </cn>
  </apply>
 </apply>
</math>
</assignmentRule>
```

```
<assignmentRule metaid="repeatedAssignment_mwcd5efd74_003e_4bc3_9d8e_3cab1dcc227f" variable="mwd2ecc838_3e25_4d27_a551_24c2d4281c13">
  <math xmlns="http://www.w3.org/1998/Math/MathML">
    <apply>
      <divide/>
      <apply>
        <divide/>
        <apply>
          <times/>
          <ci> mw16c6ff04_2e36_4d15_bccb_59d7661e10cb </ci>
          <apply>
            <minus/>
            <apply>
              <times/>
              <ci> mw98624f9d_a464_456c_9a02_026d96612aff </ci>
              <ci> mw8bc33181_fd96_4636_93eb_674dfce67260 </ci>
            </apply>
            <apply>
              <divide/>
              <ci> mw001a645f_0641_44d7_9a48_6a15ea7e4a51 </ci>
              <ci> mwa540938d_712a_46c8_b3d5_bff2c38919f8 </ci>
            </apply>
          </apply>
        </apply>
        <apply>
          <times/>
          <ci> mwe3eb2841_b1eb_4dc4_9c4c_a5d8c5cc75cd </ci>
          <ci> mwc2e367c9_e93b_406b_a5db_e77794a92b22 </ci>
        </apply>
      </apply>
      <apply>
```

<minus/>
<apply>
<plus/>
<apply>
<times/>
<apply>
<plus/>
<cn type="integer"> 1 </cn>
<apply>
<divide/>
<ci> mw001a645f_0641_44d7_9a48_6a15ea7e4a51 </ci>
<ci> mw343b1085_b0e0_4e60_8805_60c5ae4ec26b </ci>
</apply>
</apply>
<apply>
<plus/>
<cn type="integer"> 1 </cn>
<apply>
<divide/>
<ci> mw264fafe4_178f_491d_8866_ba394e95a71b </ci>
<ci> mw301fbb5f_9697_492b_8f32_015458d521c5 </ci>
</apply>
</apply>
</apply>
<apply>
<times/>
<apply>
<plus/>
<cn type="integer"> 1 </cn>
<apply>
<divide/>

```
            <ci> mw8bc33181_fd96_4636_93eb_674dfce67260 </ci>
            <ci> mwe3eb2841_b1eb_4dc4_9c4c_a5d8c5cc75cd </ci>
          </apply>
        </apply>
        <apply>
          <plus/>
          <cn type="integer"> 1 </cn>
          <apply>
            <divide/>
            <ci> mw6c1ca071_aba1_4113_ace9_3d429d27bc60 </ci>
            <ci> mw2881ad2b_2458_44be_919a_8a2cddc2a765 </ci>
          </apply>
        </apply>
        <apply>
          <plus/>
          <cn type="integer"> 1 </cn>
          <apply>
            <divide/>
            <ci> mw98624f9d_a464_456c_9a02_026d96612aff </ci>
            <ci> mwc2e367c9_e93b_406b_a5db_e77794a92b22 </ci>
          </apply>
        </apply>
      </apply>
    </apply>
    <cn type="integer"> 1 </cn>
  </apply>
 </apply>
</math>
</assignmentRule>
<assignmentRule metaid="repeatedAssignment_mw4b3b8f35_cc5d_494e_9540_533f443554b1"
variable="mw39355e27_2c4e_4381_9725_324749e9dfd0">
```

```
<math xmlns="http://www.w3.org/1998/Math/MathML">
 <apply>
  <divide/>
  <apply>
   <times/>
   <ci> mw4354fd87_eec3_45ff_ba09_8e66f6cec0d4 </ci>
   <apply>
    <minus/>
    <ci> mw1e1e1695_f297_4f58_906b_5f76ada38e61 </ci>
    <apply>
     <divide/>
     <ci> mwcd2b43d1_7092_45b9_af63_c926207ad771 </ci>
     <ci> mwfa3dac90_b557_4ea0_950c_b2e6cea1f624 </ci>
    </apply>
   </apply>
  </apply>
  <apply>
   <times/>
   <ci> mw407bee91_c458_4b42_ad72_7dea8c0f84b8 </ci>
   <apply>
    <plus/>
    <cn type="integer"> 1 </cn>
    <apply>
     <divide/>
     <ci> mw1e1e1695_f297_4f58_906b_5f76ada38e61 </ci>
     <ci> mw407bee91_c458_4b42_ad72_7dea8c0f84b8 </ci>
    </apply>
    <apply>
     <divide/>
     <ci> mwcd2b43d1_7092_45b9_af63_c926207ad771 </ci>
     <ci> mw2091e1f5_0cd3_4d23_9dcf_9f54ae671686 </ci>
```

```
          </apply>
        </apply>
      </apply>
    </apply>
  </math>
</assignmentRule>
<assignmentRule metaid="repeatedAssignment_mwb18aca6c_3d98_4338_9184_bfe0b0d18347"
variable="mwc0bac73f_72bd_4dfc_8db5_38f7dbadfa15">
  <math xmlns="http://www.w3.org/1998/Math/MathML">
    <apply>
      <divide/>
      <apply>
        <times/>
        <ci> mw9e3b0877_c7fe_4a60_886f_4c0b97f63ef9 </ci>
        <apply>
          <minus/>
          <ci> mw1e1e1695_f297_4f58_906b_5f76ada38e61 </ci>
          <apply>
            <divide/>
            <ci> mwcd2b43d1_7092_45b9_af63_c926207ad771 </ci>
            <ci> mwfa3dac90_b557_4ea0_950c_b2e6cea1f624 </ci>
          </apply>
        </apply>
      </apply>
      <apply>
        <times/>
        <ci> mw099e3275_3aed_4b7c_abd7_1d978c4681d1 </ci>
        <apply>
          <plus/>
          <cn type="integer"> 1 </cn>
          <apply>
```

<divide/>

<ci> mw1e1e1695_f297_4f58_906b_5f76ada38e61 </ci>

<ci> mw099e3275_3aed_4b7c_abd7_1d978c4681d1 </ci>

</apply>

<apply>

<divide/>

<ci> mwcd2b43d1_7092_45b9_af63_c926207ad771 </ci>

<ci> mw51232471_96c5_44a5_9906_91a4af9717da </ci>

</apply>

</apply>

</apply>

</apply>

</math>

</assignmentRule>

<assignmentRule metaid="repeatedAssignment_mw724b1ac8_35a9_43aa_a493_3c6568dd6d6b" variable="mwb358c90c_fcdf_46a5_a8c4_d6c214ad7409">

<math xmlns="http://www.w3.org/1998/Math/MathML">

<apply>

<divide/>

<apply>

<times/>

<ci> mwa466c9c9_3297_437c_baed_0dce17c19743 </ci>

<apply>

<minus/>

<ci> mw1e1e1695_f297_4f58_906b_5f76ada38e61 </ci>

<apply>

<divide/>

<ci> mwcd2b43d1_7092_45b9_af63_c926207ad771 </ci>

<ci> mwfa3dac90_b557_4ea0_950c_b2e6cea1f624 </ci>

</apply>

</apply>

</apply>

<apply>

<times/>

<ci> mwefaeb6d1_a2ae_41cb_b478_f28cb1be6a7c </ci>

<apply>

<plus/>

<cn type="integer"> 1 </cn>

<apply>

<divide/>

<ci> mw1e1e1695_f297_4f58_906b_5f76ada38e61 </ci>

<ci> mwefaeb6d1_a2ae_41cb_b478_f28cb1be6a7c </ci>

</apply>

<apply>

<divide/>

<ci> mwcd2b43d1_7092_45b9_af63_c926207ad771 </ci>

<ci> mw93985682_472b_4e3b_895b_705c1b0929b3 </ci>

</apply>

</apply>

</apply>

</apply>

</math>

</assignmentRule>

<assignmentRule metaid="repeatedAssignment_mw2009abb1_15da_4b48_b70b_15315bdeaf63" variable="mwbf496d1e_e804_42a8_a991_17b4cf79d939">

<math xmlns="http://www.w3.org/1998/Math/MathML">

<apply>

<divide/>

<apply>

<times/>

<ci> mw2603ec03_ce86_4597_b49c_ba6ac6d68e0b </ci>

<apply>

```
    <divide/>
    <apply>
      <times/>
      <ci> mwe1a7456e_ee15_425a_a731_b7dd46838115 </ci>
      <ci> mw5b3b2d76_cb36_4bfa_8e00_2fa00ad3e7c6 </ci>
    </apply>
    <apply>
      <plus/>
      <ci> mw5b3b2d76_cb36_4bfa_8e00_2fa00ad3e7c6 </ci>
      <apply>
        <times/>
        <ci> mw52f105dd_e643_48db_81f5_b4895cb10150 </ci>
        <apply>
          <plus/>
          <cn type="integer"> 1 </cn>
          <apply>
            <divide/>
            <ci> mwb534b1f7_d8d3_4922_9a23_493784f48e7d </ci>
            <ci> mw506e2da2_c32d_4748_a086_6dc0aaae9d55 </ci>
          </apply>
        </apply>
      </apply>
    </apply>
  </apply>
</apply>
<apply>
  <plus/>
  <ci> mw2603ec03_ce86_4597_b49c_ba6ac6d68e0b </ci>
  <apply>
    <times/>
    <ci> mw7c678412_1b31_4b4a_a7d6_fbbc27d86166 </ci>
```

```
    <apply>
      <plus/>
      <cn type="integer"> 1 </cn>
      <apply>
        <divide/>
        <ci> mw1e1e1695_f297_4f58_906b_5f76ada38e61 </ci>
        <ci> mwe54dda3a_7a37_48ba_bfe5_43bf6fd9d3b9 </ci>
      </apply>
      <apply>
        <divide/>
        <ci> mw001a645f_0641_44d7_9a48_6a15ea7e4a51 </ci>
        <ci> mw753db9ce_1d29_4e20_a0f6_0e6e9639bd53 </ci>
      </apply>
      <apply>
        <divide/>
        <ci> mw264fafe4_178f_491d_8866_ba394e95a71b </ci>
        <ci> mw52296718_7c93_45fd_9b1e_c13a865f9e2b </ci>
      </apply>
      <apply>
        <divide/>
        <ci> mw34d9dd05_1772_4f59_ae50_4d6ef1f63225 </ci>
        <ci> mwf40efeb1_e89c_424e_8006_a9095546bee5 </ci>
      </apply>
      <apply>
        <divide/>
        <ci> mw4e68b2da_34ac_4484_9cf5_03f0f6d9abe9 </ci>
        <ci> mwb6a831eb_5143_4bc3_9230_f7756d19cbb8 </ci>
      </apply>
    </apply>
  </apply>
</apply>
```

</apply>

</math>

</assignmentRule>

<assignmentRule metaid="repeatedAssignment_mwa91c2af6_af97_4100_840a_d0614917b12c" variable="mwcd4b57d6_55a3_45b7_a37e_2d3d83b4bec8">

<math xmlns="http://www.w3.org/1998/Math/MathML">

<apply>

<minus/>

<apply>

<divide/>

<apply>

<times/>

<ci> mw33659ae3_6e62_49ef_a1c2_a33adc049723 </ci>

<ci> mw0c5a9637_94b1_47f0_87f9_8729e75ae8de </ci>

<ci> mw4e68b2da_34ac_4484_9cf5_03f0f6d9abe9 </ci>

</apply>

<apply>

<times/>

<apply>

<plus/>

<ci> mw0c5a9637_94b1_47f0_87f9_8729e75ae8de </ci>

<ci> mw82fe7cf0_c731_49ce_9621_2c502956630b </ci>

</apply>

<apply>

<plus/>

<ci> mw4e68b2da_34ac_4484_9cf5_03f0f6d9abe9 </ci>

<ci> mw0f926ea1_951f_45a7_b0e8_afa94ba266f9 </ci>

</apply>

</apply>

</apply>

<apply>

```
<divide/>
<apply>
  <times/>
  <ci> mw30cb04f1_447a_49ab_9566_af33f6c68f02 </ci>
  <ci> mw98624f9d_a464_456c_9a02_026d96612aff </ci>
  <ci> mwf5dc5c05_4d36_4ea4_8ee0_6a1cfd2ae49b </ci>
</apply>
<apply>
  <times/>
  <apply>
    <plus/>
    <cn type="integer"> 1 </cn>
    <apply>
      <divide/>
      <ci> mw98624f9d_a464_456c_9a02_026d96612aff </ci>
      <ci> mw2b15c7a2_60bd_4539_bbca_ea2887c83c7e </ci>
    </apply>
    <apply>
      <divide/>
      <ci> mw1e1e1695_f297_4f58_906b_5f76ada38e61 </ci>
      <ci> mw66c149b7_107b_4f1e_8ba3_e998a2eb4341 </ci>
    </apply>
  </apply>
  <apply>
    <plus/>
    <cn type="integer"> 1 </cn>
    <apply>
      <divide/>
      <ci> mwf5dc5c05_4d36_4ea4_8ee0_6a1cfd2ae49b </ci>
      <ci> mw42d232ce_870c_4c6c_80e1_b80db63c24dc </ci>
    </apply>
```

```
              <apply>
                <divide/>
                <ci> mw4e68b2da_34ac_4484_9cf5_03f0f6d9abe9 </ci>
                <ci> mw3f587528_6a1e_45cf_85a1_c83170fa3db2 </ci>
              </apply>
            </apply>
          </apply>
        </apply>
      </apply>
    </math>
  </assignmentRule>
  <assignmentRule metaid="repeatedAssignment_mw3648581a_76a5_4e31_85f0_248ab7cfc8c2" variable="mw67386f72_70ed_4213_b83a_51849feda20a">
    <math xmlns="http://www.w3.org/1998/Math/MathML">
      <apply>
        <divide/>
        <apply>
          <times/>
          <ci> mw6d34a400_fde1_4a71_81d0_322fd5573e89 </ci>
          <ci> mw630d7a9f_d907_496c_b750_6c23afbb4658 </ci>
          <ci> mwf5dc5c05_4d36_4ea4_8ee0_6a1cfd2ae49b </ci>
        </apply>
        <apply>
          <times/>
          <apply>
            <plus/>
            <ci> mw630d7a9f_d907_496c_b750_6c23afbb4658 </ci>
            <ci> mw4b31666e_aef2_4756_8e67_3da9f3276f7e </ci>
          </apply>
          <apply>
            <plus/>
```

<ci> mwf5dc5c05_4d36_4ea4_8ee0_6a1cfd2ae49b </ci>

<ci> mw47eee472_fb8a_4673_8f9b_3bd3ced88c79 </ci>

</apply>

</apply>

</apply>

</math>

</assignmentRule>

<assignmentRule metaid="repeatedAssignment_mwcbe5f1c1_8508_4f54_910d_7c9f26b6fea4" variable="mw41061232_3fb7_429f_920c_084b45725d79">

<math xmlns="http://www.w3.org/1998/Math/MathML">

<apply>

<divide/>

<apply>

<divide/>

<apply>

<times/>

<ci> mw14672e46_52f5_4ab7_bff1_4b749b587f1d </ci>

<apply>

<minus/>

<ci> mwf1237a2f_b6c2_4f60_aec2_f24eaff9e01a </ci>

<apply>

<divide/>

<apply>

<times/>

<ci> mwe1915c23_bc54_4444_a6b6_afb9f835d8cf </ci>

<ci> mw6c1ca071_aba1_4113_ace9_3d429d27bc60 </ci>

</apply>

<ci> mwac1b2180_f1af_4819_9233_94af7fae5fe8 </ci>

</apply>

</apply>

</apply>

```
  <ci> mw95025ff8_a096_4b4e_b30a_c86490520d3c </ci>
</apply>
<apply>
  <times/>
  <apply>
    <plus/>
    <cn type="integer"> 1 </cn>
    <apply>
      <divide/>
      <ci> mwf1237a2f_b6c2_4f60_aec2_f24eaff9e01a </ci>
      <ci> mw95025ff8_a096_4b4e_b30a_c86490520d3c </ci>
    </apply>
    <apply>
      <divide/>
      <ci> mw6c1ca071_aba1_4113_ace9_3d429d27bc60 </ci>
      <ci> mw35d79455_d586_4f7e_b53d_b9b05403c18f </ci>
    </apply>
  </apply>
  <apply>
    <plus/>
    <cn type="integer"> 1 </cn>
    <apply>
      <divide/>
      <ci> mwe1915c23_bc54_4444_a6b6_afb9f835d8cf </ci>
      <ci> mw29daf52a_3537_43f2_b889_86b8d087fab9 </ci>
    </apply>
  </apply>
  <apply>
    <plus/>
    <cn type="integer"> 1 </cn>
    <apply>
```

<divide/>

<ci> mwb2da70f3_0970_4374_bdf2_4a3914afa142 </ci>

<ci> mwcc132bf2_7c6b_4cbb_b3e3_caddab999b30 </ci>

</apply>

<apply>

<divide/>

<ci> mw98624f9d_a464_456c_9a02_026d96612aff </ci>

<ci> mw4231ba04_0406_4368_925a_599001509b3f </ci>

</apply>

</apply>

</apply>

</apply>

</math>

</assignmentRule>

<assignmentRule metaid="repeatedAssignment_mw29e18370_5b3a_45cd_b0c8_24fe465db8d5" variable="mwaafe132e_e071_4509_96cb_b4a56a9eee89">

<math xmlns="http://www.w3.org/1998/Math/MathML">

<apply>

<divide/>

<apply>

<divide/>

<apply>

<times/>

<ci> mwe6cc0f80_5d7e_478d_8df3_785b611461a8 </ci>

<apply>

<minus/>

<ci> mwb2da70f3_0970_4374_bdf2_4a3914afa142 </ci>

<apply>

<divide/>

<apply>

<times/>

```
     <ci> mwe1915c23_bc54_4444_a6b6_afb9f835d8cf </ci>
     <ci> mw98624f9d_a464_456c_9a02_026d96612aff </ci>
    </apply>
    <ci> mwb043257b_34eb_45c4_b4bd_7fadfb6e19c8 </ci>
   </apply>
  </apply>
 </apply>
 <ci> mw94c699e4_fed2_455f_9f56_63394e10e6aa </ci>
</apply>
<apply>
 <times/>
 <apply>
  <plus/>
  <cn type="integer"> 1 </cn>
  <apply>
   <divide/>
   <ci> mwf1237a2f_b6c2_4f60_aec2_f24eaff9e01a </ci>
   <ci> mw08464d63_135a_4871_9108_9a430e7da3c9 </ci>
  </apply>
  <apply>
   <divide/>
   <ci> mw6c1ca071_aba1_4113_ace9_3d429d27bc60 </ci>
   <ci> mwf1cc2080_281e_420a_bea7_9f1ace706f8b </ci>
  </apply>
 </apply>
 <apply>
  <plus/>
  <cn type="integer"> 1 </cn>
  <apply>
   <divide/>
   <ci> mwe1915c23_bc54_4444_a6b6_afb9f835d8cf </ci>
```

```
          <ci> mw07fd01bf_c788_41e8_ad93_f9663c71e731 </ci>
         </apply>
        </apply>
        <apply>
         <plus/>
         <cn type="integer"> 1 </cn>
         <apply>
          <divide/>
          <ci> mwb2da70f3_0970_4374_bdf2_4a3914afa142 </ci>
          <ci> mw94c699e4_fed2_455f_9f56_63394e10e6aa </ci>
         </apply>
         <apply>
          <divide/>
          <ci> mw98624f9d_a464_456c_9a02_026d96612aff </ci>
          <ci> mw4925fe87_c38b_4c63_b257_76e6a00ba813 </ci>
         </apply>
        </apply>
       </apply>
      </apply>
    </math>
   </assignmentRule>
   <assignmentRule metaid="repeatedAssignment_mw7b8b8e17_d709_4f81_99be_69f94fb54cbd"
variable="mwb5e1728d_1e5f_4709_b9f3_36151c3f7fe1">
    <math xmlns="http://www.w3.org/1998/Math/MathML">
      <apply>
       <divide/>
       <apply>
        <divide/>
        <apply>
         <times/>
         <ci> mwb9289074_0186_4371_aed9_7470cba640dc </ci>
```

```
    <apply>
      <minus/>
      <ci> mwf1237a2f_b6c2_4f60_aec2_f24eaff9e01a </ci>
      <apply>
        <divide/>
        <apply>
          <times/>
          <ci> mwe1915c23_bc54_4444_a6b6_afb9f835d8cf </ci>
          <ci> mw6c1ca071_aba1_4113_ace9_3d429d27bc60 </ci>
        </apply>
        <ci> mwac1b2180_f1af_4819_9233_94af7fae5fe8 </ci>
      </apply>
    </apply>
  </apply>
  <ci> mw235e9584_299a_49b0_917e_b62cb998dc8e </ci>
</apply>
<apply>
  <times/>
  <apply>
    <plus/>
    <cn type="integer"> 1 </cn>
    <apply>
      <divide/>
      <ci> mwf1237a2f_b6c2_4f60_aec2_f24eaff9e01a </ci>
      <ci> mw235e9584_299a_49b0_917e_b62cb998dc8e </ci>
    </apply>
    <apply>
      <divide/>
      <ci> mw6c1ca071_aba1_4113_ace9_3d429d27bc60 </ci>
      <ci> mw1b9a6d90_0834_45b2_80c3_894c8f3806df </ci>
    </apply>
```

```
        </apply>
        <apply>
          <plus/>
          <cn type="integer"> 1 </cn>
          <apply>
            <divide/>
            <ci> mwe1915c23_bc54_4444_a6b6_afb9f835d8cf </ci>
            <ci> mwffdd6cd4_59fc_4211_a076_bf7bccbb2861 </ci>
          </apply>
        </apply>
        <apply>
          <plus/>
          <cn type="integer"> 1 </cn>
          <apply>
            <divide/>
            <ci> mwb2da70f3_0970_4374_bdf2_4a3914afa142 </ci>
            <ci> mwe2c4c957_b4a0_41f4_aacc_9a73304177bf </ci>
          </apply>
          <apply>
            <divide/>
            <ci> mw98624f9d_a464_456c_9a02_026d96612aff </ci>
            <ci> mwee148dba_6936_4ff5_98e9_3fafc08bf46c </ci>
          </apply>
        </apply>
      </apply>
    </apply>
  </math>
</assignmentRule>
<assignmentRule metaid="repeatedAssignment_mw96fb08a6_d5be_44db_9b05_c64494fc31ac" variable="mw2a2436c2_8c5c_4876_bdb1_1941dfad77e4">
  <math xmlns="http://www.w3.org/1998/Math/MathML">
```

```
<apply>
 <divide/>
 <apply>
  <divide/>
  <apply>
   <times/>
   <ci> mwbfebafa8_c3ee_48cc_843e_9a4bad33bfb3 </ci>
   <apply>
    <minus/>
    <ci> mwb2da70f3_0970_4374_bdf2_4a3914afa142 </ci>
    <apply>
     <divide/>
     <apply>
      <times/>
      <ci> mwe1915c23_bc54_4444_a6b6_afb9f835d8cf </ci>
      <ci> mw98624f9d_a464_456c_9a02_026d96612aff </ci>
     </apply>
     <ci> mwb043257b_34eb_45c4_b4bd_7fadfb6e19c8 </ci>
    </apply>
   </apply>
  </apply>
  <ci> mw6aa54137_d905_4ffa_a29c_b192c4901c8f </ci>
 </apply>
 <apply>
  <times/>
  <apply>
   <plus/>
   <cn type="integer"> 1 </cn>
   <apply>
    <divide/>
    <ci> mwf1237a2f_b6c2_4f60_aec2_f24eaff9e01a </ci>
```

```
    <ci> mw04c1dd77_705d_42f9_a0ad_169227a654aa </ci>
  </apply>
  <apply>
    <divide/>
    <ci> mw6c1ca071_aba1_4113_ace9_3d429d27bc60 </ci>
    <ci> mw36675533_f3f1_443f_a00a_e9f3ab7b4e43 </ci>
  </apply>
</apply>
<apply>
  <plus/>
  <cn type="integer"> 1 </cn>
  <apply>
    <divide/>
    <ci> mwe1915c23_bc54_4444_a6b6_afb9f835d8cf </ci>
    <ci> mw48d5b78e_f2bf_4cae_92f9_9b4b7e011e9a </ci>
  </apply>
</apply>
<apply>
  <plus/>
  <cn type="integer"> 1 </cn>
  <apply>
    <divide/>
    <ci> mwb2da70f3_0970_4374_bdf2_4a3914afa142 </ci>
    <ci> mw6aa54137_d905_4ffa_a29c_b192c4901c8f </ci>
  </apply>
  <apply>
    <divide/>
    <ci> mw98624f9d_a464_456c_9a02_026d96612aff </ci>
    <ci> mwc941b16b_73fe_48ea_828c_7d4127c52bdc </ci>
  </apply>
</apply>
```

```
          </apply>
        </apply>
      </math>
    </assignmentRule>
  </listOfRules>
  <listOfReactions>
    <reaction id="mw4754cad7_9090_4d76_810b_5fb4f524c792" name="CO2 import"
reversible="false" fast="false">
      <listOfReactants>
        <speciesReference species="mw4fc9d1b8_3a21_4910_bf03_3e90cc702de3"/>
      </listOfReactants>
      <listOfProducts>
        <speciesReference species="mw5b3b2d76_cb36_4bfa_8e00_2fa00ad3e7c6"/>
      </listOfProducts>
      <kineticLaw>
        <math xmlns="http://www.w3.org/1998/Math/MathML">
          <apply>
            <times/>
            <ci> mw9aa3463d_ca8c_4f73_a1b9_b2cd84be87b9 </ci>
            <ci> mw4fc9d1b8_3a21_4910_bf03_3e90cc702de3 </ci>
          </apply>
        </math>
      </kineticLaw>
    </reaction>
    <reaction id="mw778f1882_19c7_429f_96c7_3566f2362e38" name="LIGHT_1 (ATP synthase)"
reversible="false" fast="false">
      <annotation>
        <COPASI xmlns="http://www.copasi.org/static/sbml">
          <rdf:RDF xmlns:dcterms="http://purl.org/dc/terms/"
xmlns:rdf="http://www.w3.org/1999/02/22-rdf-syntax-ns#">
            <rdf:Description rdf:about="#COPASI65">
              <dcterms:created>
```

```
        <rdf:Description>
          <dcterms:W3CDTF>2010-05-24T02:11:27Z</dcterms:W3CDTF>
        </rdf:Description>
      </dcterms:created>
    </rdf:Description>
  </rdf:RDF>
 </COPASI>
</annotation>
<listOfReactants>
 <speciesReference species="mw34d9dd05_1772_4f59_ae50_4d6ef1f63225"/>
 <speciesReference species="mw4b092c2e_9b30_46e1_85f7_9bb39d9b10aa"/>
</listOfReactants>
<listOfProducts>
 <speciesReference species="mwa083eb39_db7c_4dc7_a1b7_5d06c9120757"/>
</listOfProducts>
<kineticLaw>
 <math xmlns="http://www.w3.org/1998/Math/MathML">
  <apply>
   <times/>
   <ci> mwf2a81dda_e6ba_4ef2_82e7_ac9c7c029cd8 </ci>
   <ci> mw34d9dd05_1772_4f59_ae50_4d6ef1f63225 </ci>
   <ci> mw4b092c2e_9b30_46e1_85f7_9bb39d9b10aa </ci>
  </apply>
 </math>
</kineticLaw>
</reaction>
<reaction id="mwad717e32_e6d2_44b2_9aca_1bf71acc947a" name="LIGHT_2"
reversible="false" fast="false">
<annotation>
 <COPASI xmlns="http://www.copasi.org/static/sbml">
  <rdf:RDF xmlns:dcterms="http://purl.org/dc/terms/"
xmlns:rdf="http://www.w3.org/1999/02/22-rdf-syntax-ns#">
```

<rdf:Description rdf:about="#COPASI66">
<dcterms:created>
<rdf:Description>
<dcterms:W3CDTF>2010-06-09T00:18:40Z</dcterms:W3CDTF>
</rdf:Description>
</dcterms:created>
</rdf:Description>
</rdf:RDF>
</COPASI>
</annotation>
<listOfReactants>
<speciesReference species="mwf5dc5c05_4d36_4ea4_8ee0_6a1cfd2ae49b"/>
</listOfReactants>
<listOfProducts>
<speciesReference species="mw4e68b2da_34ac_4484_9cf5_03f0f6d9abe9"/>
</listOfProducts>
<kineticLaw>
<math xmlns="http://www.w3.org/1998/Math/MathML">
<apply>
<times/>
<ci> mw942ee080_9495_43cb_8637_43348cad5712 </ci>
<ci> mwf5dc5c05_4d36_4ea4_8ee0_6a1cfd2ae49b </ci>
</apply>
</math>
</kineticLaw>
</reaction>
<reaction id="mwc52d157f_7cd4_4c6a_aa11_30ea0e1a7bb4" name="CC_1 (RuBisCO)" reversible="false" fast="false">
<annotation>
<COPASI xmlns="http://www.copasi.org/static/sbml">
<rdf:RDF xmlns:dcterms="http://purl.org/dc/terms/" xmlns:rdf="http://www.w3.org/1999/02/22-rdf-syntax-ns#">

```
        <rdf:Description rdf:about="#COPASI49">
          <dcterms:created>
            <rdf:Description>
              <dcterms:W3CDTF>2010-04-14T14:47:32Z</dcterms:W3CDTF>
            </rdf:Description>
          </dcterms:created>
        </rdf:Description>
      </rdf:RDF>
    </COPASI>
  </annotation>
  <listOfReactants>
    <speciesReference species="mw2603ec03_ce86_4597_b49c_ba6ac6d68e0b"/>
    <speciesReference species="mw5b3b2d76_cb36_4bfa_8e00_2fa00ad3e7c6"/>
    <speciesReference species="mwb534b1f7_d8d3_4922_9a23_493784f48e7d"/>
    <speciesReference species="mw001a645f_0641_44d7_9a48_6a15ea7e4a51"/>
    <speciesReference species="mw264fafe4_178f_491d_8866_ba394e95a71b"/>
    <speciesReference species="mw34d9dd05_1772_4f59_ae50_4d6ef1f63225"/>
    <speciesReference species="mw4e68b2da_34ac_4484_9cf5_03f0f6d9abe9"/>
  </listOfReactants>
  <listOfProducts>
    <speciesReference species="mw1e1e1695_f297_4f58_906b_5f76ada38e61"
stoichiometry="2"/>
    <speciesReference species="mwb534b1f7_d8d3_4922_9a23_493784f48e7d"/>
    <speciesReference species="mw001a645f_0641_44d7_9a48_6a15ea7e4a51"/>
    <speciesReference species="mw264fafe4_178f_491d_8866_ba394e95a71b"/>
    <speciesReference species="mw34d9dd05_1772_4f59_ae50_4d6ef1f63225"/>
    <speciesReference species="mw4e68b2da_34ac_4484_9cf5_03f0f6d9abe9"/>
  </listOfProducts>
  <kineticLaw>
    <math xmlns="http://www.w3.org/1998/Math/MathML">
      <apply>
```

```
<divide/>
<apply>
 <times/>
 <ci> mw2603ec03_ce86_4597_b49c_ba6ac6d68e0b </ci>
 <apply>
  <divide/>
  <apply>
   <times/>
   <ci> mw3eb7fb4b_04fb_4fb5_a285_b18042ae9a2a </ci>
   <ci> mw5b3b2d76_cb36_4bfa_8e00_2fa00ad3e7c6 </ci>
  </apply>
  <apply>
   <plus/>
   <ci> mw5b3b2d76_cb36_4bfa_8e00_2fa00ad3e7c6 </ci>
   <apply>
    <times/>
    <ci> mw52f105dd_e643_48db_81f5_b4895cb10150 </ci>
    <apply>
     <plus/>
     <cn type="integer"> 1 </cn>
     <apply>
      <divide/>
      <ci> mwb534b1f7_d8d3_4922_9a23_493784f48e7d </ci>
      <ci> mw506e2da2_c32d_4748_a086_6dc0aaae9d55 </ci>
     </apply>
    </apply>
   </apply>
  </apply>
 </apply>
</apply>
<apply>
```

<plus/>

<ci> mw2603ec03_ce86_4597_b49c_ba6ac6d68e0b </ci>

<apply>

<times/>

<ci> mw7c678412_1b31_4b4a_a7d6_fbbc27d86166 </ci>

<apply>

<plus/>

<cn type="integer"> 1 </cn>

<apply>

<divide/>

<ci> mw1e1e1695_f297_4f58_906b_5f76ada38e61 </ci>

<ci> mwe54dda3a_7a37_48ba_bfe5_43bf6fd9d3b9 </ci>

</apply>

<apply>

<divide/>

<ci> mw001a645f_0641_44d7_9a48_6a15ea7e4a51 </ci>

<ci> mw753db9ce_1d29_4e20_a0f6_0e6e9639bd53 </ci>

</apply>

<apply>

<divide/>

<ci> mw264fafe4_178f_491d_8866_ba394e95a71b </ci>

<ci> mw52296718_7c93_45fd_9b1e_c13a865f9e2b </ci>

</apply>

<apply>

<divide/>

<ci> mw34d9dd05_1772_4f59_ae50_4d6ef1f63225 </ci>

<ci> mwf40efeb1_e89c_424e_8006_a9095546bee5 </ci>

</apply>

<apply>

<divide/>

<ci> mw4e68b2da_34ac_4484_9cf5_03f0f6d9abe9 </ci>

```
                <ci> mwb6a831eb_5143_4bc3_9230_f7756d19cbb8 </ci>
               </apply>
              </apply>
             </apply>
            </apply>
           </apply>
          </math>
        </kineticLaw>
      </reaction>
      <reaction id="mwd5c93146_b0d3_42e9_94a2_e441bd3b6372" name="CC_2 (phosphoglycerate
kinase)" fast="false">
        <notes>
          <body xmlns="http://www.w3.org/1999/xhtml">
            <pre>Vmax sn&#xeD;&#x17e;eno z 10.3 na 2</pre>
          </body>
        </notes>
        <annotation>
          <COPASI xmlns="http://www.copasi.org/static/sbml">
            <rdf:RDF xmlns:dcterms="http://purl.org/dc/terms/"
xmlns:rdf="http://www.w3.org/1999/02/22-rdf-syntax-ns#">
              <rdf:Description rdf:about="#COPASI50">
                <dcterms:created>
                  <rdf:Description>
                    <dcterms:W3CDTF>2010-04-14T15:57:26Z</dcterms:W3CDTF>
                  </rdf:Description>
                </dcterms:created>
              </rdf:Description>
            </rdf:RDF>
          </COPASI>
        </annotation>
        <listOfReactants>
          <speciesReference species="mw1e1e1695_f297_4f58_906b_5f76ada38e61"/>
```

```
 <speciesReference species="mwa083eb39_db7c_4dc7_a1b7_5d06c9120757"/>
</listOfReactants>
<listOfProducts>
 <speciesReference species="mw0c5a9637_94b1_47f0_87f9_8729e75ae8de"/>
 <speciesReference species="mw4b092c2e_9b30_46e1_85f7_9bb39d9b10aa"/>
</listOfProducts>
<kineticLaw>
 <math xmlns="http://www.w3.org/1998/Math/MathML">
  <apply>
   <divide/>
   <apply>
    <divide/>
    <apply>
     <times/>
     <ci> mwec7d9352_a492_407e_b7e6_2b403e29e97c </ci>
     <apply>
      <minus/>
      <apply>
       <times/>
       <ci> mw1e1e1695_f297_4f58_906b_5f76ada38e61 </ci>
       <ci> mwa083eb39_db7c_4dc7_a1b7_5d06c9120757 </ci>
      </apply>
      <apply>
       <divide/>
       <apply>
        <times/>
        <ci> mw0c5a9637_94b1_47f0_87f9_8729e75ae8de </ci>
        <ci> mw4b092c2e_9b30_46e1_85f7_9bb39d9b10aa </ci>
       </apply>
       <ci> mw9d2a6be6_f7f4_4871_82c1_5a6d5cf40bbc </ci>
      </apply>
```

```
    </apply>
  </apply>
  <apply>
    <times/>
    <ci> mwdaffc0cc_4d0d_49e1_ac01_aaad36724d06 </ci>
    <ci> mw8a532dd1_c07c_4f64_89c8_0e880b187648 </ci>
  </apply>
</apply>
<apply>
  <times/>
  <apply>
    <plus/>
    <cn type="integer"> 1 </cn>
    <apply>
      <divide/>
      <ci> mw1e1e1695_f297_4f58_906b_5f76ada38e61 </ci>
      <ci> mwdaffc0cc_4d0d_49e1_ac01_aaad36724d06 </ci>
    </apply>
    <apply>
      <divide/>
      <ci> mw0c5a9637_94b1_47f0_87f9_8729e75ae8de </ci>
      <ci> mwe85d1b9f_58eb_48ae_8033_765e75b5bc89 </ci>
    </apply>
  </apply>
  <apply>
    <plus/>
    <cn type="integer"> 1 </cn>
    <apply>
      <divide/>
      <ci> mwa083eb39_db7c_4dc7_a1b7_5d06c9120757 </ci>
      <ci> mw8a532dd1_c07c_4f64_89c8_0e880b187648 </ci>
```

</apply>
<apply>
<divide/>
<ci> mw4b092c2e_9b30_46e1_85f7_9bb39d9b10aa </ci>
<ci> mw60c4515d_f1f6_46fd_9cb5_2c58ee609270 </ci>
</apply>
</apply>
</apply>
</apply>
</math>
</kineticLaw>
</reaction>
<reaction id="mw69dc3872_9137_4aa1_8c45_9eb30ac46150" name="CC_3 (glyceraldehyde 3-phosphate dehydrogenase)" reversible="false" fast="false">
<notes>
cannot fit the parameters, even irreversible, model is super sensitive
</notes>
<annotation>
<COPASI xmlns="http://www.copasi.org/static/sbml">
<rdf:RDF xmlns:dcterms="http://purl.org/dc/terms/" xmlns:rdf="http://www.w3.org/1999/02/22-rdf-syntax-ns#">
<rdf:Description rdf:about="#COPASI51">
<dcterms:created>
<rdf:Description>
<dcterms:W3CDTF>2010-04-14T16:10:42Z</dcterms:W3CDTF>
</rdf:Description>
</dcterms:created>
</rdf:Description>
</rdf:RDF>
</COPASI>
</annotation>
<listOfReactants>

```
  <speciesReference species="mw0c5a9637_94b1_47f0_87f9_8729e75ae8de"/>
  <speciesReference species="mw4e68b2da_34ac_4484_9cf5_03f0f6d9abe9"/>
</listOfReactants>
<listOfProducts>
  <speciesReference species="mw98624f9d_a464_456c_9a02_026d96612aff"/>
  <speciesReference species="mwf5dc5c05_4d36_4ea4_8ee0_6a1cfd2ae49b"/>
</listOfProducts>
<kineticLaw>
  <math xmlns="http://www.w3.org/1998/Math/MathML">
    <apply>
      <divide/>
      <apply>
        <times/>
        <ci> mw33659ae3_6e62_49ef_a1c2_a33adc049723 </ci>
        <ci> mw0c5a9637_94b1_47f0_87f9_8729e75ae8de </ci>
        <ci> mw4e68b2da_34ac_4484_9cf5_03f0f6d9abe9 </ci>
      </apply>
      <apply>
        <times/>
        <apply>
          <plus/>
          <ci> mw0c5a9637_94b1_47f0_87f9_8729e75ae8de </ci>
          <ci> mw82fe7cf0_c731_49ce_9621_2c502956630b </ci>
        </apply>
        <apply>
          <plus/>
          <ci> mw4e68b2da_34ac_4484_9cf5_03f0f6d9abe9 </ci>
          <ci> mw0f926ea1_951f_45a7_b0e8_afa94ba266f9 </ci>
        </apply>
      </apply>
    </apply>
```

```
      </math>
     </kineticLaw>
    </reaction>
    <reaction id="mw217cf156_db05_4272_a1c7_dc6abf6e656a" name="CC_4 (triose phosphate
isomerase)" fast="false">
     <annotation>
      <COPASI xmlns="http://www.copasi.org/static/sbml">
       <rdf:RDF xmlns:dcterms="http://purl.org/dc/terms/"
xmlns:rdf="http://www.w3.org/1999/02/22-rdf-syntax-ns#">
        <rdf:Description rdf:about="#COPASI52">
         <dcterms:created>
          <rdf:Description>
           <dcterms:W3CDTF>2010-04-14T16:18:37Z</dcterms:W3CDTF>
          </rdf:Description>
         </dcterms:created>
        </rdf:Description>
       </rdf:RDF>
      </COPASI>
     </annotation>
     <listOfReactants>
      <speciesReference species="mw98624f9d_a464_456c_9a02_026d96612aff"/>
     </listOfReactants>
     <listOfProducts>
      <speciesReference species="mw8bc33181_fd96_4636_93eb_674dfce67260"/>
     </listOfProducts>
     <kineticLaw>
      <math xmlns="http://www.w3.org/1998/Math/MathML">
       <apply>
        <divide/>
        <apply>
         <divide/>
         <apply>
```

```
        <times/>
        <ci> mw696548be_270b_4aa4_a957_53f3540f254b </ci>
        <apply>
          <minus/>
          <ci> mw98624f9d_a464_456c_9a02_026d96612aff </ci>
          <apply>
            <divide/>
            <ci> mw8bc33181_fd96_4636_93eb_674dfce67260 </ci>
            <ci> mw587a43b1_6567_47d8_ab28_db86856d6189 </ci>
          </apply>
        </apply>
      </apply>
      <ci> mw18156bae_84ae_479f_a552_4b556b807e65 </ci>
    </apply>
    <apply>
      <plus/>
      <cn type="integer"> 1 </cn>
      <apply>
        <divide/>
        <ci> mw98624f9d_a464_456c_9a02_026d96612aff </ci>
        <ci> mw18156bae_84ae_479f_a552_4b556b807e65 </ci>
      </apply>
      <apply>
        <divide/>
        <ci> mw8bc33181_fd96_4636_93eb_674dfce67260 </ci>
        <ci> mw6e36548d_68ee_4f99_bdf4_bc0081dec083 </ci>
      </apply>
    </apply>
  </apply>
 </math>
</kineticLaw>
```

```xml
      </reaction>
      <reaction id="mw439b8aa6_a964_4797_96b3_87bffcb644f1" name="CC_5 (aldolase) alpha"
fast="false">
        <annotation>
          <COPASI xmlns="http://www.copasi.org/static/sbml">
            <rdf:RDF xmlns:dcterms="http://purl.org/dc/terms/"
xmlns:rdf="http://www.w3.org/1999/02/22-rdf-syntax-ns#">
              <rdf:Description rdf:about="#COPASI53">
                <dcterms:created>
                  <rdf:Description>
                    <dcterms:W3CDTF>2010-04-14T16:46:06Z</dcterms:W3CDTF>
                  </rdf:Description>
                </dcterms:created>
              </rdf:Description>
            </rdf:RDF>
          </COPASI>
        </annotation>
        <listOfReactants>
          <speciesReference species="mw98624f9d_a464_456c_9a02_026d96612aff"/>
          <speciesReference species="mw8bc33181_fd96_4636_93eb_674dfce67260"/>
          <speciesReference species="mw264fafe4_178f_491d_8866_ba394e95a71b"/>
          <speciesReference species="mw6c1ca071_aba1_4113_ace9_3d429d27bc60"/>
        </listOfReactants>
        <listOfProducts>
          <speciesReference species="mw001a645f_0641_44d7_9a48_6a15ea7e4a51"/>
          <speciesReference species="mw264fafe4_178f_491d_8866_ba394e95a71b"/>
          <speciesReference species="mw6c1ca071_aba1_4113_ace9_3d429d27bc60"/>
        </listOfProducts>
        <kineticLaw>
          <math xmlns="http://www.w3.org/1998/Math/MathML">
            <apply>
              <divide/>
```

<apply>
<divide/>
<apply>
<times/>
<ci> mw5b9d10ab_7daf_4921_a8cb_86028e0d31e6 </ci>
<apply>
<minus/>
<apply>
<times/>
<ci> mw98624f9d_a464_456c_9a02_026d96612aff </ci>
<ci> mw8bc33181_fd96_4636_93eb_674dfce67260 </ci>
</apply>
<apply>
<divide/>
<ci> mw001a645f_0641_44d7_9a48_6a15ea7e4a51 </ci>
<ci> mwa540938d_712a_46c8_b3d5_bff2c38919f8 </ci>
</apply>
</apply>
</apply>
<apply>
<times/>
<ci> mw2664b98d_62f2_4597_97e0_ac8bf9badc1c </ci>
<ci> mw1ef19eb9_d783_49e0_ab98_1512e9923aff </ci>
</apply>
</apply>
<apply>
<minus/>
<apply>
<plus/>
<apply>
<times/>

```
   <apply>
    <plus/>
    <cn type="integer"> 1 </cn>
    <apply>
     <divide/>
     <ci> mw001a645f_0641_44d7_9a48_6a15ea7e4a51 </ci>
     <ci> mw7d7ad1c0_08d2_429b_89a5_44adb5fc08f2 </ci>
    </apply>
   </apply>
   <apply>
    <plus/>
    <cn type="integer"> 1 </cn>
    <apply>
     <divide/>
     <ci> mw264fafe4_178f_491d_8866_ba394e95a71b </ci>
     <ci> mwed39790d_132b_4762_acf2_55e9d5d068be </ci>
    </apply>
   </apply>
  </apply>
  <apply>
   <times/>
   <apply>
    <plus/>
    <cn type="integer"> 1 </cn>
    <apply>
     <divide/>
     <ci> mw8bc33181_fd96_4636_93eb_674dfce67260 </ci>
     <ci> mw2664b98d_62f2_4597_97e0_ac8bf9badc1c </ci>
    </apply>
   </apply>
   <apply>
```

```
          <plus/>
          <cn type="integer"> 1 </cn>
          <apply>
            <divide/>
            <ci> mw6c1ca071_aba1_4113_ace9_3d429d27bc60 </ci>
            <ci> mw6f8f2f0d_dfd6_4df4_b62b_5cc576a99618 </ci>
          </apply>
        </apply>
        <apply>
          <plus/>
          <cn type="integer"> 1 </cn>
          <apply>
            <divide/>
            <ci> mw98624f9d_a464_456c_9a02_026d96612aff </ci>
            <ci> mw1ef19eb9_d783_49e0_ab98_1512e9923aff </ci>
          </apply>
        </apply>
      </apply>
    </apply>
    <cn type="integer"> 1 </cn>
   </apply>
  </apply>
 </math>
</kineticLaw>
</reaction>
<reaction id="mw8014eb5c_a580_4062_942b_d449ad4755f4" name="CC_5 (aldolase) beta"
fast="false">
 <listOfReactants>
  <speciesReference species="mw98624f9d_a464_456c_9a02_026d96612aff"/>
  <speciesReference species="mw8bc33181_fd96_4636_93eb_674dfce67260"/>
  <speciesReference species="mw264fafe4_178f_491d_8866_ba394e95a71b"/>
```

```
<speciesReference species="mw6c1ca071_aba1_4113_ace9_3d429d27bc60"/>
</listOfReactants>
<listOfProducts>
  <speciesReference species="mw001a645f_0641_44d7_9a48_6a15ea7e4a51"/>
  <speciesReference species="mw264fafe4_178f_491d_8866_ba394e95a71b"/>
  <speciesReference species="mw6c1ca071_aba1_4113_ace9_3d429d27bc60"/>
</listOfProducts>
<kineticLaw>
  <math xmlns="http://www.w3.org/1998/Math/MathML">
    <apply>
      <divide/>
      <apply>
        <divide/>
        <apply>
          <times/>
          <ci> mw16c6ff04_2e36_4d15_bccb_59d7661e10cb </ci>
          <apply>
            <minus/>
            <apply>
              <times/>
              <ci> mw98624f9d_a464_456c_9a02_026d96612aff </ci>
              <ci> mw8bc33181_fd96_4636_93eb_674dfce67260 </ci>
            </apply>
            <apply>
              <divide/>
              <ci> mw001a645f_0641_44d7_9a48_6a15ea7e4a51 </ci>
              <ci> mwa540938d_712a_46c8_b3d5_bff2c38919f8 </ci>
            </apply>
          </apply>
        </apply>
        <apply>
```

```
    <times/>
    <ci> mwe3eb2841_b1eb_4dc4_9c4c_a5d8c5cc75cd </ci>
    <ci> mwc2e367c9_e93b_406b_a5db_e77794a92b22 </ci>
  </apply>
</apply>
<apply>
  <minus/>
  <apply>
    <plus/>
    <apply>
      <times/>
      <apply>
        <plus/>
        <cn type="integer"> 1 </cn>
        <apply>
          <divide/>
          <ci> mw001a645f_0641_44d7_9a48_6a15ea7e4a51 </ci>
          <ci> mw343b1085_b0e0_4e60_8805_60c5ae4ec26b </ci>
        </apply>
      </apply>
      <apply>
        <plus/>
        <cn type="integer"> 1 </cn>
        <apply>
          <divide/>
          <ci> mw264fafe4_178f_491d_8866_ba394e95a71b </ci>
          <ci> mw301fbb5f_9697_492b_8f32_015458d521c5 </ci>
        </apply>
      </apply>
    </apply>
    <apply>
```

<times/>
<apply>
<plus/>
<cn type="integer"> 1 </cn>
<apply>
<divide/>
<ci> mw8bc33181_fd96_4636_93eb_674dfce67260 </ci>
<ci> mwe3eb2841_b1eb_4dc4_9c4c_a5d8c5cc75cd </ci>
</apply>
</apply>
<apply>
<plus/>
<cn type="integer"> 1 </cn>
<apply>
<divide/>
<ci> mw6c1ca071_aba1_4113_ace9_3d429d27bc60 </ci>
<ci> mw2881ad2b_2458_44be_919a_8a2cddc2a765 </ci>
</apply>
</apply>
<apply>
<plus/>
<cn type="integer"> 1 </cn>
<apply>
<divide/>
<ci> mw98624f9d_a464_456c_9a02_026d96612aff </ci>
<ci> mwc2e367c9_e93b_406b_a5db_e77794a92b22 </ci>
</apply>
</apply>
</apply>
</apply>
<cn type="integer"> 1 </cn>

```
        </apply>
      </apply>
    </math>
  </kineticLaw>
</reaction>
<reaction id="mw29cb94a3_c753_4556_a5fc_1e9b8517df02" name="CC_6 alpha (fructose-1,6-
bisphosphatase)" reversible="false" fast="false">
  <annotation>
    <COPASI xmlns="http://www.copasi.org/static/sbml">
      <rdf:RDF xmlns:dcterms="http://purl.org/dc/terms/"
xmlns:rdf="http://www.w3.org/1999/02/22-rdf-syntax-ns#">
        <rdf:Description rdf:about="#COPASI54">
          <dcterms:created>
            <rdf:Description>
              <dcterms:W3CDTF>2010-04-14T16:52:19Z</dcterms:W3CDTF>
            </rdf:Description>
          </dcterms:created>
        </rdf:Description>
      </rdf:RDF>
    </COPASI>
  </annotation>
  <listOfReactants>
    <speciesReference species="mw001a645f_0641_44d7_9a48_6a15ea7e4a51"/>
  </listOfReactants>
  <listOfProducts>
    <speciesReference species="mwf1237a2f_b6c2_4f60_aec2_f24eaff9e01a"/>
    <speciesReference species="mw34d9dd05_1772_4f59_ae50_4d6ef1f63225"/>
  </listOfProducts>
  <kineticLaw>
    <math xmlns="http://www.w3.org/1998/Math/MathML">
      <apply>
        <divide/>
```

```xml
        <apply>
          <times/>
          <ci> mw48d7d26d_fe30_4b1e_a4ee_a4eef1cfcb6b </ci>
          <ci> mw001a645f_0641_44d7_9a48_6a15ea7e4a51 </ci>
        </apply>
        <apply>
          <plus/>
          <ci> mw001a645f_0641_44d7_9a48_6a15ea7e4a51 </ci>
          <apply>
            <times/>
            <ci> mw86a32edf_a962_44c9_adfe_8059b0c656c9 </ci>
            <apply>
              <plus/>
              <cn type="integer"> 1 </cn>
              <apply>
                <divide/>
                <ci> mwf1237a2f_b6c2_4f60_aec2_f24eaff9e01a </ci>
                <ci> mwae1595b9_af51_4b6b_b328_ae530ded7fe0 </ci>
              </apply>
              <apply>
                <divide/>
                <ci> mw34d9dd05_1772_4f59_ae50_4d6ef1f63225 </ci>
                <ci> mw5d188609_730f_4a50_84a3_654056528e7a </ci>
              </apply>
            </apply>
          </apply>
        </apply>
      </apply>
    </math>
  </kineticLaw>
</reaction>
```

```
<reaction id="mwbc15fc9a_0af4_4748_b362_1a717a74e73c" name="CC_7 (transketolase)"
fast="false">
  <annotation>
    <COPASI xmlns="http://www.copasi.org/static/sbml">
      <rdf:RDF xmlns:dcterms="http://purl.org/dc/terms/"
xmlns:rdf="http://www.w3.org/1999/02/22-rdf-syntax-ns#">
        <rdf:Description rdf:about="#COPASI55">
          <dcterms:created>
            <rdf:Description>
              <dcterms:W3CDTF>2010-04-15T18:06:55Z</dcterms:W3CDTF>
            </rdf:Description>
          </dcterms:created>
        </rdf:Description>
      </rdf:RDF>
    </COPASI>
  </annotation>
  <listOfReactants>
    <speciesReference species="mwf1237a2f_b6c2_4f60_aec2_f24eaff9e01a"/>
    <speciesReference species="mw98624f9d_a464_456c_9a02_026d96612aff"/>
    <speciesReference species="mwa5f72579_82b4_4f63_802b_ae5d613cef27"/>
    <speciesReference species="mw7b7d97f6_2db4_4473_80be_5e4d69e05b59"/>
  </listOfReactants>
  <listOfProducts>
    <speciesReference species="mw6c1ca071_aba1_4113_ace9_3d429d27bc60"/>
    <speciesReference species="mwb2da70f3_0970_4374_bdf2_4a3914afa142"/>
    <speciesReference species="mwa5f72579_82b4_4f63_802b_ae5d613cef27"/>
    <speciesReference species="mw7b7d97f6_2db4_4473_80be_5e4d69e05b59"/>
  </listOfProducts>
  <kineticLaw>
    <math xmlns="http://www.w3.org/1998/Math/MathML">
      <apply>
        <divide/>
```

<apply>
<divide/>
<apply>
<times/>
<ci> mwc5b9dbbd_d536_4b37_9dd4_99d0328d3414 </ci>
<apply>
<minus/>
<apply>
<times/>
<ci> mwf1237a2f_b6c2_4f60_aec2_f24eaff9e01a </ci>
<ci> mw98624f9d_a464_456c_9a02_026d96612aff </ci>
</apply>
<apply>
<divide/>
<apply>
<times/>
<ci> mwb2da70f3_0970_4374_bdf2_4a3914afa142 </ci>
<ci> mw6c1ca071_aba1_4113_ace9_3d429d27bc60 </ci>
</apply>
<ci> mwc97c27ed_cba8_4dda_8745_bc8e8da8a6b2 </ci>
</apply>
</apply>
</apply>
<apply>
<times/>
<ci> mwf76b7498_870d_4272_aed2_2e9cdad4acd9 </ci>
<ci> mw1c53df26_ee90_4319_898d_ab1565c18d13 </ci>
</apply>
</apply>
<apply>
<times/>

```
<apply>
  <plus/>
  <cn type="integer"> 1 </cn>
  <apply>
    <divide/>
    <ci> mwf1237a2f_b6c2_4f60_aec2_f24eaff9e01a </ci>
    <ci> mwf76b7498_870d_4272_aed2_2e9cdad4acd9 </ci>
  </apply>
  <apply>
    <divide/>
    <ci> mw6c1ca071_aba1_4113_ace9_3d429d27bc60 </ci>
    <ci> mw2df7359c_5ca8_46cf_830c_64f2aef66d6a </ci>
  </apply>
</apply>
<apply>
  <plus/>
  <cn type="integer"> 1 </cn>
  <apply>
    <divide/>
    <ci> mw98624f9d_a464_456c_9a02_026d96612aff </ci>
    <ci> mw1c53df26_ee90_4319_898d_ab1565c18d13 </ci>
  </apply>
  <apply>
    <divide/>
    <ci> mwb2da70f3_0970_4374_bdf2_4a3914afa142 </ci>
    <ci> mw5f7db393_8e3c_4a77_9cfc_395acd74d218 </ci>
  </apply>
</apply>
<apply>
  <plus/>
  <cn type="integer"> 1 </cn>
```

```
            <apply>
              <divide/>
              <ci> mwa5f72579_82b4_4f63_802b_ae5d613cef27 </ci>
              <ci> mw5d1be663_9187_43e2_95e7_5852c1c840a8 </ci>
            </apply>
            <apply>
              <divide/>
              <ci> mw7b7d97f6_2db4_4473_80be_5e4d69e05b59 </ci>
              <ci> mw319586e5_21bf_4aef_ac3a_736836d7e448 </ci>
            </apply>
          </apply>
        </apply>
      </apply>
    </math>
  </kineticLaw>
</reaction>
<reaction id="mwad76d387_f3d0_456b_8863_d454954a15c5" name="CC_8 (aldolase)"
fast="false">
  <annotation>
    <COPASI xmlns="http://www.copasi.org/static/sbml">
      <rdf:RDF xmlns:dcterms="http://purl.org/dc/terms/"
xmlns:rdf="http://www.w3.org/1999/02/22-rdf-syntax-ns#">
        <rdf:Description rdf:about="#COPASI56">
          <dcterms:created>
            <rdf:Description>
              <dcterms:W3CDTF>2010-04-20T11:20:56Z</dcterms:W3CDTF>
            </rdf:Description>
          </dcterms:created>
        </rdf:Description>
      </rdf:RDF>
    </COPASI>
  </annotation>
```

```
<listOfReactants>
  <speciesReference species="mw8bc33181_fd96_4636_93eb_674dfce67260"/>
  <speciesReference species="mw6c1ca071_aba1_4113_ace9_3d429d27bc60"/>
  <speciesReference species="mw001a645f_0641_44d7_9a48_6a15ea7e4a51"/>
  <speciesReference species="mw98624f9d_a464_456c_9a02_026d96612aff"/>
</listOfReactants>
<listOfProducts>
  <speciesReference species="mw264fafe4_178f_491d_8866_ba394e95a71b"/>
  <speciesReference species="mw001a645f_0641_44d7_9a48_6a15ea7e4a51"/>
  <speciesReference species="mw98624f9d_a464_456c_9a02_026d96612aff"/>
</listOfProducts>
<kineticLaw>
  <math xmlns="http://www.w3.org/1998/Math/MathML">
    <apply>
      <divide/>
      <apply>
        <divide/>
        <apply>
          <times/>
          <ci> mwc4332dd6_0358_4c1a_ae97_1131d7ea48c2 </ci>
          <apply>
            <minus/>
            <apply>
              <times/>
              <ci> mw8bc33181_fd96_4636_93eb_674dfce67260 </ci>
              <ci> mw6c1ca071_aba1_4113_ace9_3d429d27bc60 </ci>
            </apply>
            <apply>
              <divide/>
              <ci> mw264fafe4_178f_491d_8866_ba394e95a71b </ci>
              <ci> mw08f10a43_f575_462a_b1d4_5b4943f2aecf </ci>
```

```
      </apply>
    </apply>
  </apply>
  <apply>
    <times/>
    <ci> mw2664b98d_62f2_4597_97e0_ac8bf9badc1c </ci>
    <ci> mw6f8f2f0d_dfd6_4df4_b62b_5cc576a99618 </ci>
  </apply>
</apply>
<apply>
  <minus/>
  <apply>
    <plus/>
    <apply>
      <times/>
      <apply>
        <plus/>
        <cn type="integer"> 1 </cn>
        <apply>
          <divide/>
          <ci> mw001a645f_0641_44d7_9a48_6a15ea7e4a51 </ci>
          <ci> mw7d7ad1c0_08d2_429b_89a5_44adb5fc08f2 </ci>
        </apply>
      </apply>
      <apply>
        <plus/>
        <cn type="integer"> 1 </cn>
        <apply>
          <divide/>
          <ci> mw264fafe4_178f_491d_8866_ba394e95a71b </ci>
          <ci> mwed39790d_132b_4762_acf2_55e9d5d068be </ci>
```

```
    </apply>
  </apply>
</apply>
<apply>
  <times/>
  <apply>
    <plus/>
    <cn type="integer"> 1 </cn>
    <apply>
      <divide/>
      <ci> mw8bc33181_fd96_4636_93eb_674dfce67260 </ci>
      <ci> mw2664b98d_62f2_4597_97e0_ac8bf9badc1c </ci>
    </apply>
  </apply>
  <apply>
    <plus/>
    <cn type="integer"> 1 </cn>
    <apply>
      <divide/>
      <ci> mw6c1ca071_aba1_4113_ace9_3d429d27bc60 </ci>
      <ci> mw6f8f2f0d_dfd6_4df4_b62b_5cc576a99618 </ci>
    </apply>
  </apply>
  <apply>
    <plus/>
    <cn type="integer"> 1 </cn>
    <apply>
      <divide/>
      <ci> mw98624f9d_a464_456c_9a02_026d96612aff </ci>
      <ci> mw1ef19eb9_d783_49e0_ab98_1512e9923aff </ci>
    </apply>
```

```
              </apply>
            </apply>
          </apply>
          <cn type="integer"> 1 </cn>
        </apply>
      </apply>
    </math>
  </kineticLaw>
</reaction>
<reaction id="mw949fb1b8_61c1_4eba_9798_2c6d82fc9588" name="CC_8 (aldolase) beta"
fast="false">
  <listOfReactants>
    <speciesReference species="mw8bc33181_fd96_4636_93eb_674dfce67260"/>
    <speciesReference species="mw6c1ca071_aba1_4113_ace9_3d429d27bc60"/>
    <speciesReference species="mw001a645f_0641_44d7_9a48_6a15ea7e4a51"/>
    <speciesReference species="mw98624f9d_a464_456c_9a02_026d96612aff"/>
  </listOfReactants>
  <listOfProducts>
    <speciesReference species="mw264fafe4_178f_491d_8866_ba394e95a71b"/>
    <speciesReference species="mw001a645f_0641_44d7_9a48_6a15ea7e4a51"/>
    <speciesReference species="mw98624f9d_a464_456c_9a02_026d96612aff"/>
  </listOfProducts>
  <kineticLaw>
    <math xmlns="http://www.w3.org/1998/Math/MathML">
      <apply>
        <divide/>
        <apply>
          <divide/>
          <apply>
            <times/>
            <ci> mwd9ae7c23_c98b_4e49_8f0c_1c722eaa18d6 </ci>
```

```
    <apply>
      <minus/>
      <apply>
        <times/>
        <ci> mw8bc33181_fd96_4636_93eb_674dfce67260 </ci>
        <ci> mw6c1ca071_aba1_4113_ace9_3d429d27bc60 </ci>
      </apply>
      <apply>
        <divide/>
        <ci> mw264fafe4_178f_491d_8866_ba394e95a71b </ci>
        <ci> mw08f10a43_f575_462a_b1d4_5b4943f2aecf </ci>
      </apply>
    </apply>
  </apply>
  <apply>
    <times/>
    <ci> mwe3eb2841_b1eb_4dc4_9c4c_a5d8c5cc75cd </ci>
    <ci> mw2881ad2b_2458_44be_919a_8a2cddc2a765 </ci>
  </apply>
</apply>
<apply>
  <minus/>
  <apply>
    <plus/>
    <apply>
      <times/>
      <apply>
        <plus/>
        <cn type="integer"> 1 </cn>
        <apply>
          <divide/>
```

```
      <ci> mw001a645f_0641_44d7_9a48_6a15ea7e4a51 </ci>
      <ci> mw343b1085_b0e0_4e60_8805_60c5ae4ec26b </ci>
    </apply>
  </apply>
  <apply>
    <plus/>
    <cn type="integer"> 1 </cn>
    <apply>
      <divide/>
      <ci> mw264fafe4_178f_491d_8866_ba394e95a71b </ci>
      <ci> mw301fbb5f_9697_492b_8f32_015458d521c5 </ci>
    </apply>
  </apply>
</apply>
<apply>
  <times/>
  <apply>
    <plus/>
    <cn type="integer"> 1 </cn>
    <apply>
      <divide/>
      <ci> mw8bc33181_fd96_4636_93eb_674dfce67260 </ci>
      <ci> mwe3eb2841_b1eb_4dc4_9c4c_a5d8c5cc75cd </ci>
    </apply>
  </apply>
  <apply>
    <plus/>
    <cn type="integer"> 1 </cn>
    <apply>
      <divide/>
      <ci> mw6c1ca071_aba1_4113_ace9_3d429d27bc60 </ci>
```

<ci> mw2881ad2b_2458_44be_919a_8a2cddc2a765 </ci>

</apply>

</apply>

<apply>

<plus/>

<cn type="integer"> 1 </cn>

<apply>

<divide/>

<ci> mw98624f9d_a464_456c_9a02_026d96612aff </ci>

<ci> mwc2e367c9_e93b_406b_a5db_e77794a92b22 </ci>

</apply>

</apply>

</apply>

</apply>

<cn type="integer"> 1 </cn>

</apply>

</apply>

</math>

</kineticLaw>

</reaction>

<reaction id="mw1be9e309_73ee_4116_ae9b_764ead1a785f" name="CC_9 (sedoheptulose-1,7 bisphosphatase)" reversible="false" fast="false">

<annotation>

<COPASI xmlns="http://www.copasi.org/static/sbml">

<rdf:RDF xmlns:dcterms="http://purl.org/dc/terms/" xmlns:rdf="http://www.w3.org/1999/02/22-rdf-syntax-ns#">

<rdf:Description rdf:about="#COPASI57">

<dcterms:created>

<rdf:Description>

<dcterms:W3CDTF>2010-04-21T02:31:25Z</dcterms:W3CDTF>

</rdf:Description>

</dcterms:created>

```
      </rdf:Description>
    </rdf:RDF>
  </COPASI>
</annotation>
<listOfReactants>
  <speciesReference species="mw264fafe4_178f_491d_8866_ba394e95a71b"/>
  <speciesReference species="mw001a645f_0641_44d7_9a48_6a15ea7e4a51"/>
</listOfReactants>
<listOfProducts>
  <speciesReference species="mwa5f72579_82b4_4f63_802b_ae5d613cef27"/>
  <speciesReference species="mw001a645f_0641_44d7_9a48_6a15ea7e4a51"/>
</listOfProducts>
<kineticLaw>
  <math xmlns="http://www.w3.org/1998/Math/MathML">
    <apply>
      <divide/>
      <apply>
        <times/>
        <ci> mw9c962784_a662_43bc_816d_58889af49cc2 </ci>
        <ci> mw264fafe4_178f_491d_8866_ba394e95a71b </ci>
      </apply>
      <apply>
        <plus/>
        <ci> mw264fafe4_178f_491d_8866_ba394e95a71b </ci>
        <apply>
          <times/>
          <ci> mw65b811e9_89c2_407c_a470_8f071a6422d5 </ci>
          <apply>
            <plus/>
            <cn type="integer"> 1 </cn>
            <apply>
```

```
            <divide/>
            <ci> mw001a645f_0641_44d7_9a48_6a15ea7e4a51 </ci>
            <ci> mweb78553f_492c_44fc_b049_30f4089f5e52 </ci>
           </apply>
          </apply>
         </apply>
        </apply>
       </apply>
      </math>
     </kineticLaw>
    </reaction>
    <reaction id="mw18beaca2_3a63_40e9_bebd_033e3aa8251a" name="CC_10 (transketolase)"
fast="false">
     <annotation>
      <COPASI xmlns="http://www.copasi.org/static/sbml">
       <rdf:RDF xmlns:dcterms="http://purl.org/dc/terms/"
xmlns:rdf="http://www.w3.org/1999/02/22-rdf-syntax-ns#">
        <rdf:Description rdf:about="#COPASI58">
         <dcterms:created>
          <rdf:Description>
           <dcterms:W3CDTF>2010-04-21T03:46:16Z</dcterms:W3CDTF>
          </rdf:Description>
         </dcterms:created>
        </rdf:Description>
       </rdf:RDF>
      </COPASI>
     </annotation>
     <listOfReactants>
      <speciesReference species="mwa5f72579_82b4_4f63_802b_ae5d613cef27"/>
      <speciesReference species="mw98624f9d_a464_456c_9a02_026d96612aff"/>
      <speciesReference species="mwf1237a2f_b6c2_4f60_aec2_f24eaff9e01a"/>
      <speciesReference species="mw6c1ca071_aba1_4113_ace9_3d429d27bc60"/>
```

```
</listOfReactants>
<listOfProducts>
 <speciesReference species="mw7b7d97f6_2db4_4473_80be_5e4d69e05b59"/>
 <speciesReference species="mwb2da70f3_0970_4374_bdf2_4a3914afa142"/>
 <speciesReference species="mwf1237a2f_b6c2_4f60_aec2_f24eaff9e01a"/>
 <speciesReference species="mw6c1ca071_aba1_4113_ace9_3d429d27bc60"/>
</listOfProducts>
<kineticLaw>
 <math xmlns="http://www.w3.org/1998/Math/MathML">
  <apply>
   <divide/>
   <apply>
    <divide/>
    <apply>
     <times/>
     <ci> mw520d09f0_2f53_4caa_8bd1_f1066e47098f </ci>
     <apply>
      <minus/>
      <apply>
       <times/>
       <ci> mw98624f9d_a464_456c_9a02_026d96612aff </ci>
       <ci> mwa5f72579_82b4_4f63_802b_ae5d613cef27 </ci>
      </apply>
      <apply>
       <divide/>
       <apply>
        <times/>
        <ci> mw7b7d97f6_2db4_4473_80be_5e4d69e05b59 </ci>
        <ci> mwb2da70f3_0970_4374_bdf2_4a3914afa142 </ci>
       </apply>
       <ci> mwdab67499_dc15_4f7a_b42a_b553d395bdeb </ci>
```

</apply>
</apply>
</apply>
<apply>
<times/>
<ci> mw5d1be663_9187_43e2_95e7_5852c1c840a8 </ci>
<ci> mw1c53df26_ee90_4319_898d_ab1565c18d13 </ci>
</apply>
</apply>
<apply>
<times/>
<apply>
<plus/>
<cn type="integer"> 1 </cn>
<apply>
<divide/>
<ci> mwf1237a2f_b6c2_4f60_aec2_f24eaff9e01a </ci>
<ci> mwf76b7498_870d_4272_aed2_2e9cdad4acd9 </ci>
</apply>
<apply>
<divide/>
<ci> mw6c1ca071_aba1_4113_ace9_3d429d27bc60 </ci>
<ci> mw2df7359c_5ca8_46cf_830c_64f2aef66d6a </ci>
</apply>
</apply>
<apply>
<plus/>
<cn type="integer"> 1 </cn>
<apply>
<divide/>
<ci> mw98624f9d_a464_456c_9a02_026d96612aff </ci>

```
          <ci> mw1c53df26_ee90_4319_898d_ab1565c18d13 </ci>
         </apply>
         <apply>
          <divide/>
          <ci> mwb2da70f3_0970_4374_bdf2_4a3914afa142 </ci>
          <ci> mw5f7db393_8e3c_4a77_9cfc_395acd74d218 </ci>
         </apply>
        </apply>
        <apply>
         <plus/>
         <cn type="integer"> 1 </cn>
         <apply>
          <divide/>
          <ci> mwa5f72579_82b4_4f63_802b_ae5d613cef27 </ci>
          <ci> mw5d1be663_9187_43e2_95e7_5852c1c840a8 </ci>
         </apply>
         <apply>
          <divide/>
          <ci> mw7b7d97f6_2db4_4473_80be_5e4d69e05b59 </ci>
          <ci> mw319586e5_21bf_4aef_ac3a_736836d7e448 </ci>
         </apply>
        </apply>
       </apply>
      </apply>
     </math>
    </kineticLaw>
   </reaction>
   <reaction id="mwafafbc4e_bb7d_44dc_89e5_9bc05611e759" name="C_11 (phosphopentose
isomerase)" fast="false">
    <annotation>
     <COPASI xmlns="http://www.copasi.org/static/sbml">
```

```
          <rdf:RDF xmlns:dcterms="http://purl.org/dc/terms/"
xmlns:rdf="http://www.w3.org/1999/02/22-rdf-syntax-ns#">
            <rdf:Description rdf:about="#COPASI59">
              <dcterms:created>
                <rdf:Description>
                  <dcterms:W3CDTF>2010-04-23T03:43:39Z</dcterms:W3CDTF>
                </rdf:Description>
              </dcterms:created>
            </rdf:Description>
          </rdf:RDF>
        </COPASI>
      </annotation>
      <listOfReactants>
        <speciesReference species="mw7b7d97f6_2db4_4473_80be_5e4d69e05b59"/>
      </listOfReactants>
      <listOfProducts>
        <speciesReference species="mw580d5da5_ec62_4ea2_8f87_a5d617982c02"/>
      </listOfProducts>
      <kineticLaw>
        <math xmlns="http://www.w3.org/1998/Math/MathML">
          <apply>
            <divide/>
            <apply>
              <divide/>
              <apply>
                <times/>
                <ci> mw9ac027ed_faba_463f_8575_f0e6d69200bd </ci>
                <apply>
                  <minus/>
                  <ci> mw7b7d97f6_2db4_4473_80be_5e4d69e05b59 </ci>
                  <apply>
```

<divide/>
<ci> mw580d5da5_ec62_4ea2_8f87_a5d617982c02 </ci>
<ci> mw3143b349_becb_4982_a83f_441e274ee00c </ci>
</apply>
</apply>
</apply>
<ci> mw576b0b82_5947_49ce_8f54_e086cc631504 </ci>
</apply>
<apply>
<plus/>
<cn type="integer"> 1 </cn>
<apply>
<divide/>
<ci> mw580d5da5_ec62_4ea2_8f87_a5d617982c02 </ci>
<ci> mw53176689_3f53_433a_a683_9edf99a5993d </ci>
</apply>
<apply>
<divide/>
<ci> mw7b7d97f6_2db4_4473_80be_5e4d69e05b59 </ci>
<ci> mw576b0b82_5947_49ce_8f54_e086cc631504 </ci>
</apply>
</apply>
</apply>
</math>
</kineticLaw>
</reaction>
<reaction id="mw7b5e6900_c1d6_4fc7_bad6_1c3cd125c6a6" name="C_12 (phosphopentose epimerase)" fast="false">
<annotation>
<COPASI xmlns="http://www.copasi.org/static/sbml">
<rdf:RDF xmlns:dcterms="http://purl.org/dc/terms/" xmlns:rdf="http://www.w3.org/1999/02/22-rdf-syntax-ns#">

```
      <rdf:Description rdf:about="#COPASI60">
        <dcterms:created>
          <rdf:Description>
            <dcterms:W3CDTF>2010-04-23T03:43:48Z</dcterms:W3CDTF>
          </rdf:Description>
        </dcterms:created>
      </rdf:Description>
    </rdf:RDF>
  </COPASI>
</annotation>
<listOfReactants>
  <speciesReference species="mwb2da70f3_0970_4374_bdf2_4a3914afa142"/>
</listOfReactants>
<listOfProducts>
  <speciesReference species="mw580d5da5_ec62_4ea2_8f87_a5d617982c02"/>
</listOfProducts>
<kineticLaw>
  <math xmlns="http://www.w3.org/1998/Math/MathML">
    <apply>
      <divide/>
      <apply>
        <divide/>
        <apply>
          <times/>
          <ci> mw634909f8_5cc7_478f_b6b4_3d713718216b </ci>
          <apply>
            <minus/>
            <ci> mwb2da70f3_0970_4374_bdf2_4a3914afa142 </ci>
            <apply>
              <divide/>
              <ci> mw580d5da5_ec62_4ea2_8f87_a5d617982c02 </ci>
```

```
                <ci> mw9973b455_04bb_4ef2_8bad_e5d9038f2afa </ci>
              </apply>
            </apply>
          </apply>
          <ci> mw770450fd_fa82_4c62_8132_cbc289403f10 </ci>
        </apply>
        <apply>
          <plus/>
          <cn type="integer"> 1 </cn>
          <apply>
            <divide/>
            <ci> mw580d5da5_ec62_4ea2_8f87_a5d617982c02 </ci>
            <ci> mw916809f8_ec94_4f28_aaaa_56521dfe234b </ci>
          </apply>
          <apply>
            <divide/>
            <ci> mwb2da70f3_0970_4374_bdf2_4a3914afa142 </ci>
            <ci> mw770450fd_fa82_4c62_8132_cbc289403f10 </ci>
          </apply>
        </apply>
      </apply>
    </math>
  </kineticLaw>
</reaction>
<reaction id="mwbbecb8c2_c87a_4a29_a0fe_c64fca1701df" name="C_13
(phosphoribulokinase)" reversible="false" fast="false">
  <annotation>
    <COPASI xmlns="http://www.copasi.org/static/sbml">
      <rdf:RDF xmlns:dcterms="http://purl.org/dc/terms/"
xmlns:rdf="http://www.w3.org/1999/02/22-rdf-syntax-ns#">
        <rdf:Description rdf:about="#COPASI61">
          <dcterms:created>
```

```
        <rdf:Description>
          <dcterms:W3CDTF>2010-04-23T03:48:04Z</dcterms:W3CDTF>
        </rdf:Description>
      </dcterms:created>
    </rdf:Description>
  </rdf:RDF>
</COPASI>
</annotation>
<listOfReactants>
  <speciesReference species="mw580d5da5_ec62_4ea2_8f87_a5d617982c02"/>
  <speciesReference species="mwa083eb39_db7c_4dc7_a1b7_5d06c9120757"/>
  <speciesReference species="mw34d9dd05_1772_4f59_ae50_4d6ef1f63225"/>
  <speciesReference species="mw1e1e1695_f297_4f58_906b_5f76ada38e61"/>
</listOfReactants>
<listOfProducts>
  <speciesReference species="mw2603ec03_ce86_4597_b49c_ba6ac6d68e0b"/>
  <speciesReference species="mw4b092c2e_9b30_46e1_85f7_9bb39d9b10aa"/>
  <speciesReference species="mw34d9dd05_1772_4f59_ae50_4d6ef1f63225"/>
  <speciesReference species="mw1e1e1695_f297_4f58_906b_5f76ada38e61"/>
</listOfProducts>
<kineticLaw>
  <math xmlns="http://www.w3.org/1998/Math/MathML">
    <apply>
      <divide/>
      <apply>
        <times/>
        <ci> mw442d2399_d826_45dc_96d1_1d9a3af70032 </ci>
        <ci> mwa083eb39_db7c_4dc7_a1b7_5d06c9120757 </ci>
        <ci> mw580d5da5_ec62_4ea2_8f87_a5d617982c02 </ci>
      </apply>
      <apply>
```

```
<times/>
<apply>
  <plus/>
  <apply>
    <times/>
    <ci> mwa083eb39_db7c_4dc7_a1b7_5d06c9120757 </ci>
    <apply>
      <plus/>
      <cn type="integer"> 1 </cn>
      <apply>
        <divide/>
        <ci> mw4b092c2e_9b30_46e1_85f7_9bb39d9b10aa </ci>
        <ci> mw55d72d42_b4f1_4c2d_8b39_594a18219b62 </ci>
      </apply>
    </apply>
  </apply>
  <apply>
    <times/>
    <ci> mw0d24671c_626c_4751_a2f5_52d895c46b12 </ci>
    <apply>
      <plus/>
      <cn type="integer"> 1 </cn>
      <apply>
        <divide/>
        <ci> mw4b092c2e_9b30_46e1_85f7_9bb39d9b10aa </ci>
        <ci> mw353036af_5e46_4c9c_92b2_191591d2c857 </ci>
      </apply>
    </apply>
  </apply>
</apply>
<apply>
```

```
        <plus/>
        <ci> mw580d5da5_ec62_4ea2_8f87_a5d617982c02 </ci>
        <apply>
          <times/>
          <ci> mw51f910d1_180e_4a92_aa3e_17f247a0f4fe </ci>
          <apply>
            <plus/>
            <cn type="integer"> 1 </cn>
            <apply>
              <divide/>
              <ci> mw1e1e1695_f297_4f58_906b_5f76ada38e61 </ci>
              <ci> mweb5b46a0_a515_418a_a2c7_5986240a61ff </ci>
            </apply>
            <apply>
              <divide/>
              <ci> mw2603ec03_ce86_4597_b49c_ba6ac6d68e0b </ci>
              <ci> mw62747a3d_d67f_443e_bf91_3c16927d07c9 </ci>
            </apply>
            <apply>
              <divide/>
              <ci> mw34d9dd05_1772_4f59_ae50_4d6ef1f63225 </ci>
              <ci> mwdacf95a3_1ffa_43b0_8a8b_04a0c41ea664 </ci>
            </apply>
          </apply>
        </apply>
      </apply>
    </apply>
  </apply>
 </math>
 </kineticLaw>
</reaction>
```

```
<reaction id="mw77395e11_016b_42cb_b0ce_fa41174e38d7" name="GPI (glucosephosphate isomerase)" fast="false">
  <annotation>
    <COPASI xmlns="http://www.copasi.org/static/sbml">
      <rdf:RDF xmlns:dcterms="http://purl.org/dc/terms/" xmlns:rdf="http://www.w3.org/1999/02/22-rdf-syntax-ns#">
        <rdf:Description rdf:about="#COPASI62">
          <dcterms:created>
            <rdf:Description>
              <dcterms:W3CDTF>2010-04-29T13:08:18Z</dcterms:W3CDTF>
            </rdf:Description>
          </dcterms:created>
        </rdf:Description>
      </rdf:RDF>
    </COPASI>
  </annotation>
  <listOfReactants>
    <speciesReference species="mwf1237a2f_b6c2_4f60_aec2_f24eaff9e01a"/>
  </listOfReactants>
  <listOfProducts>
    <speciesReference species="mwc5d2bfb5_b299_4e35_b8b7_6f7ff0ce2a5a"/>
  </listOfProducts>
  <kineticLaw>
    <math xmlns="http://www.w3.org/1998/Math/MathML">
      <apply>
        <divide/>
        <apply>
          <divide/>
          <apply>
            <times/>
            <ci> mw1dc7ae44_7d88_420e_8084_bc013cd8b1dc </ci>
            <apply>
```

```
          <minus/>
          <ci> mwf1237a2f_b6c2_4f60_aec2_f24eaff9e01a </ci>
          <apply>
            <divide/>
            <ci> mwc5d2bfb5_b299_4e35_b8b7_6f7ff0ce2a5a </ci>
            <ci> mw6d3e7fb2_fbcb_4028_ae3c_377e5ec0c707 </ci>
          </apply>
        </apply>
      </apply>
      <ci> mw38214f65_8acd_4a84_b1d6_59c785833902 </ci>
    </apply>
    <apply>
      <plus/>
      <apply>
        <divide/>
        <apply>
          <times/>
          <cn type="integer"> 1 </cn>
          <ci> mwf1237a2f_b6c2_4f60_aec2_f24eaff9e01a </ci>
        </apply>
        <ci> mw38214f65_8acd_4a84_b1d6_59c785833902 </ci>
      </apply>
      <apply>
        <divide/>
        <ci> mwc5d2bfb5_b299_4e35_b8b7_6f7ff0ce2a5a </ci>
        <ci> mw7a8cfa64_22c5_4f72_b851_6fa78ce1d00a </ci>
      </apply>
    </apply>
  </apply>
</math>
</kineticLaw>
```

```
  </reaction>
  <reaction id="mw88a08588_3eb3_435e_a36f_7a24e86f067d" name="OPP_1 (glucose-6-phosphate dehydrogenase)" reversible="false" fast="false">
   <listOfReactants>
    <speciesReference species="mwc5d2bfb5_b299_4e35_b8b7_6f7ff0ce2a5a"/>
   </listOfReactants>
   <listOfProducts>
    <speciesReference species="mw630d7a9f_d907_496c_b750_6c23afbb4658"/>
   </listOfProducts>
   <kineticLaw>
    <math xmlns="http://www.w3.org/1998/Math/MathML">
     <apply>
      <divide/>
      <apply>
       <times/>
       <ci> mw9726ca8c_0c0e_4d16_bae1_3d731a5408aa </ci>
       <ci> mwc5d2bfb5_b299_4e35_b8b7_6f7ff0ce2a5a </ci>
      </apply>
      <apply>
       <times/>
       <ci> mw2db94c60_e05f_4408_bc54_54fb9f9f7d42 </ci>
       <apply>
        <plus/>
        <cn type="integer"> 1 </cn>
        <apply>
         <divide/>
         <ci> mwc5d2bfb5_b299_4e35_b8b7_6f7ff0ce2a5a </ci>
         <ci> mw2db94c60_e05f_4408_bc54_54fb9f9f7d42 </ci>
        </apply>
        <apply>
         <divide/>
```

```
        <ci> mw630d7a9f_d907_496c_b750_6c23afbb4658 </ci>
        <ci> mw361ddcb4_26fd_439c_b85e_c582a34f84b1 </ci>
       </apply>
      </apply>
     </apply>
    </apply>
   </math>
  </kineticLaw>
 </reaction>
 <reaction id="mw5c036684_f866_4c62_920d_38bf0ad5aab9" name="OPP_2 (phosphogluconate
dehydrogenase)" reversible="false" fast="false">
  <listOfReactants>
   <speciesReference species="mw630d7a9f_d907_496c_b750_6c23afbb4658"/>
   <speciesReference species="mwf5dc5c05_4d36_4ea4_8ee0_6a1cfd2ae49b"/>
  </listOfReactants>
  <listOfProducts>
   <speciesReference species="mw580d5da5_ec62_4ea2_8f87_a5d617982c02"/>
   <speciesReference species="mw4e68b2da_34ac_4484_9cf5_03f0f6d9abe9"/>
  </listOfProducts>
  <kineticLaw>
   <math xmlns="http://www.w3.org/1998/Math/MathML">
    <apply>
     <divide/>
     <apply>
      <times/>
      <ci> mw6d34a400_fde1_4a71_81d0_322fd5573e89 </ci>
      <ci> mw630d7a9f_d907_496c_b750_6c23afbb4658 </ci>
      <ci> mwf5dc5c05_4d36_4ea4_8ee0_6a1cfd2ae49b </ci>
     </apply>
     <apply>
      <times/>
```

<apply>

<plus/>

<ci> mw630d7a9f_d907_496c_b750_6c23afbb4658 </ci>

<ci> mw4b31666e_aef2_4756_8e67_3da9f3276f7e </ci>

</apply>

<apply>

<plus/>

<ci> mwf5dc5c05_4d36_4ea4_8ee0_6a1cfd2ae49b </ci>

<ci> mw47eee472_fb8a_4673_8f9b_3bd3ced88c79 </ci>

</apply>

</apply>

</apply>

</math>

</kineticLaw>

</reaction>

<reaction id="mw1c94eec9_9746_462d_be1c_a788c3b879cc" name="GSM_1 (simplified phosphoserine transaminase)" reversible="false" fast="false">

<annotation>

<COPASI xmlns="http://www.copasi.org/static/sbml">

<rdf:RDF xmlns:dcterms="http://purl.org/dc/terms/" xmlns:rdf="http://www.w3.org/1999/02/22-rdf-syntax-ns#">

<rdf:Description rdf:about="#COPASI81">

<dcterms:created>

<rdf:Description>

<dcterms:W3CDTF>2011-06-15T16:11:22Z</dcterms:W3CDTF>

</rdf:Description>

</dcterms:created>

</rdf:Description>

</rdf:RDF>

</COPASI>

</annotation>

<listOfReactants>

```
      <speciesReference species="mw1e1e1695_f297_4f58_906b_5f76ada38e61"/>
    </listOfReactants>
    <listOfProducts>
      <speciesReference species="mw4bff44c2_7520_41bc_80fb_bd781f8b6503"/>
    </listOfProducts>
    <kineticLaw>
      <math xmlns="http://www.w3.org/1998/Math/MathML">
        <apply>
          <divide/>
          <apply>
            <times/>
            <ci> mwd12025b7_9ac7_4a08_ae36_005fa0a2ad82 </ci>
            <ci> mw1e1e1695_f297_4f58_906b_5f76ada38e61 </ci>
          </apply>
          <apply>
            <plus/>
            <ci> mw09424d24_2a0c_4c48_99fd_174b17fae4c6 </ci>
            <ci> mw1e1e1695_f297_4f58_906b_5f76ada38e61 </ci>
          </apply>
        </apply>
      </math>
    </kineticLaw>
  </reaction>
  <reaction id="mwfa68b360_5777_4d6a_9b2a_46cdfcd0096b" name="GSM_2 (glycine
transaminase)" reversible="false" fast="false">
    <listOfReactants>
      <speciesReference species="mwa60ca2e1_0142_4294_8726_6b633c50a6bf"/>
    </listOfReactants>
    <listOfProducts>
      <speciesReference species="mw7ee6ca0b_cc39_45ef_a044_36cfeb983c0b"/>
    </listOfProducts>
```

```
    <kineticLaw>
      <math xmlns="http://www.w3.org/1998/Math/MathML">
        <apply>
          <divide/>
          <apply>
            <times/>
            <ci> mw71ca7b9e_7a15_48c2_af0a_3b49048dc22c </ci>
            <ci> mwa60ca2e1_0142_4294_8726_6b633c50a6bf </ci>
          </apply>
          <apply>
            <plus/>
            <ci> mw561b9d1e_a922_4d5d_a419_705bdcdc1479 </ci>
            <ci> mwa60ca2e1_0142_4294_8726_6b633c50a6bf </ci>
          </apply>
        </apply>
      </math>
    </kineticLaw>
  </reaction>
  <reaction id="mwbfec15b6_38b0_48b8_a741_df9125b7c386" name="PP_1 (RuBisCO)"
reversible="false" fast="false">
    <listOfReactants>
      <speciesReference species="mw2603ec03_ce86_4597_b49c_ba6ac6d68e0b"/>
      <speciesReference species="mw5b3b2d76_cb36_4bfa_8e00_2fa00ad3e7c6"/>
      <speciesReference species="mwb534b1f7_d8d3_4922_9a23_493784f48e7d"/>
      <speciesReference species="mw1e1e1695_f297_4f58_906b_5f76ada38e61"/>
      <speciesReference species="mw001a645f_0641_44d7_9a48_6a15ea7e4a51"/>
      <speciesReference species="mw264fafe4_178f_491d_8866_ba394e95a71b"/>
      <speciesReference species="mw34d9dd05_1772_4f59_ae50_4d6ef1f63225"/>
      <speciesReference species="mw4e68b2da_34ac_4484_9cf5_03f0f6d9abe9"/>
    </listOfReactants>
    <listOfProducts>
```

```
  <speciesReference species="mw69137d0b_f529_4e9a_b7ef_33dffd020665"/>
  <speciesReference species="mw1e1e1695_f297_4f58_906b_5f76ada38e61"/>
  <speciesReference species="mwb534b1f7_d8d3_4922_9a23_493784f48e7d"/>
  <speciesReference species="mw001a645f_0641_44d7_9a48_6a15ea7e4a51"/>
  <speciesReference species="mw264fafe4_178f_491d_8866_ba394e95a71b"/>
  <speciesReference species="mw34d9dd05_1772_4f59_ae50_4d6ef1f63225"/>
  <speciesReference species="mw4e68b2da_34ac_4484_9cf5_03f0f6d9abe9"/>
</listOfProducts>
<kineticLaw>
  <math xmlns="http://www.w3.org/1998/Math/MathML">
    <apply>
      <divide/>
      <apply>
        <times/>
        <ci> mw2603ec03_ce86_4597_b49c_ba6ac6d68e0b </ci>
        <apply>
          <divide/>
          <apply>
            <times/>
            <ci> mwe1a7456e_ee15_425a_a731_b7dd46838115 </ci>
            <ci> mw5b3b2d76_cb36_4bfa_8e00_2fa00ad3e7c6 </ci>
          </apply>
          <apply>
            <plus/>
            <ci> mw5b3b2d76_cb36_4bfa_8e00_2fa00ad3e7c6 </ci>
            <apply>
              <times/>
              <ci> mw52f105dd_e643_48db_81f5_b4895cb10150 </ci>
              <apply>
                <plus/>
                <cn type="integer"> 1 </cn>
```

<apply>
<divide/>
<ci> mwb534b1f7_d8d3_4922_9a23_493784f48e7d </ci>
<ci> mw506e2da2_c32d_4748_a086_6dc0aaae9d55 </ci>
</apply>
</apply>
</apply>
</apply>
</apply>
</apply>
<apply>
<plus/>
<ci> mw2603ec03_ce86_4597_b49c_ba6ac6d68e0b </ci>
<apply>
<times/>
<ci> mw7c678412_1b31_4b4a_a7d6_fbbc27d86166 </ci>
<apply>
<plus/>
<cn type="integer"> 1 </cn>
<apply>
<divide/>
<ci> mw1e1e1695_f297_4f58_906b_5f76ada38e61 </ci>
<ci> mwe54dda3a_7a37_48ba_bfe5_43bf6fd9d3b9 </ci>
</apply>
<apply>
<divide/>
<ci> mw001a645f_0641_44d7_9a48_6a15ea7e4a51 </ci>
<ci> mw753db9ce_1d29_4e20_a0f6_0e6e9639bd53 </ci>
</apply>
<apply>
<divide/>

<ci> mw264fafe4_178f_491d_8866_ba394e95a71b </ci>

<ci> mw52296718_7c93_45fd_9b1e_c13a865f9e2b </ci>

</apply>

<apply>

<divide/>

<ci> mw34d9dd05_1772_4f59_ae50_4d6ef1f63225 </ci>

<ci> mwf40efeb1_e89c_424e_8006_a9095546bee5 </ci>

</apply>

<apply>

<divide/>

<ci> mw4e68b2da_34ac_4484_9cf5_03f0f6d9abe9 </ci>

<ci> mwb6a831eb_5143_4bc3_9230_f7756d19cbb8 </ci>

</apply>

</apply>

</apply>

</apply>

</apply>

</math>

</kineticLaw>

</reaction>

<reaction id="mw33f027c9_9ebf_4d65_a74c_57c5bb2b18e6" name="PP_2a (phosphoglycolate phosphatase)" reversible="false" fast="false">

<annotation>

<COPASI xmlns="http://www.copasi.org/static/sbml">

<rdf:RDF xmlns:dcterms="http://purl.org/dc/terms/" xmlns:rdf="http://www.w3.org/1999/02/22-rdf-syntax-ns#">

<rdf:Description rdf:about="#COPASI70">

<dcterms:created>

<rdf:Description>

<dcterms:W3CDTF>2011-05-09T23:56:05Z</dcterms:W3CDTF>

</rdf:Description>

</dcterms:created>

```
      </rdf:Description>
    </rdf:RDF>
  </COPASI>
</annotation>
<listOfReactants>
  <speciesReference species="mw69137d0b_f529_4e9a_b7ef_33dffd020665"/>
</listOfReactants>
<listOfProducts>
  <speciesReference species="mw62b862f7_f0c7_48df_895b_319e5b84b577"/>
  <speciesReference species="mw34d9dd05_1772_4f59_ae50_4d6ef1f63225"/>
</listOfProducts>
<kineticLaw>
  <math xmlns="http://www.w3.org/1998/Math/MathML">
    <apply>
      <divide/>
      <apply>
        <times/>
        <ci> mwac20e884_51a5_40a9_b093_21b8def92417 </ci>
        <ci> mw69137d0b_f529_4e9a_b7ef_33dffd020665 </ci>
      </apply>
      <apply>
        <plus/>
        <ci> mw69137d0b_f529_4e9a_b7ef_33dffd020665 </ci>
        <apply>
          <times/>
          <ci> mwbd86004a_c6e8_4b82_b6bc_a1fc8148a985 </ci>
          <apply>
            <plus/>
            <cn type="integer"> 1 </cn>
            <apply>
              <divide/>
```

```
          <ci> mw62b862f7_f0c7_48df_895b_319e5b84b577 </ci>
          <ci> mw55bd15f3_cb3c_4fda_b599_ac7410bffac2 </ci>
         </apply>
        </apply>
        <apply>
         <plus/>
         <cn type="integer"> 1 </cn>
         <apply>
          <divide/>
          <ci> mw34d9dd05_1772_4f59_ae50_4d6ef1f63225 </ci>
          <ci> mwaea44262_a9e9_4810_9d88_b4d97c605854 </ci>
         </apply>
        </apply>
       </apply>
      </apply>
     </apply>
    </math>
   </kineticLaw>
  </reaction>
  <reaction id="mw7d55131e_3dcf_44a3_ad5a_e795fdce96c5" name="PP_2b (phosphoglycolate
phosphatase)" reversible="false" fast="false">
   <listOfReactants>
    <speciesReference species="mw69137d0b_f529_4e9a_b7ef_33dffd020665"/>
   </listOfReactants>
   <listOfProducts>
    <speciesReference species="mw62b862f7_f0c7_48df_895b_319e5b84b577"/>
    <speciesReference species="mw34d9dd05_1772_4f59_ae50_4d6ef1f63225"/>
   </listOfProducts>
   <kineticLaw>
    <math xmlns="http://www.w3.org/1998/Math/MathML">
     <apply>
```

<divide/>
<apply>
  <times/>
  <ci> mw9aac7a54_37ae_4bc1_abc6_f66f5ce326f8 </ci>
  <ci> mw69137d0b_f529_4e9a_b7ef_33dffd020665 </ci>
</apply>
<apply>
  <plus/>
  <ci> mw69137d0b_f529_4e9a_b7ef_33dffd020665 </ci>
  <apply>
    <times/>
    <ci> mw70e0ec5e_4fd7_4384_ace0_bb4f0fe59eb5 </ci>
    <apply>
      <plus/>
      <cn type="integer"> 1 </cn>
      <apply>
        <divide/>
        <ci> mw62b862f7_f0c7_48df_895b_319e5b84b577 </ci>
        <ci> mw55bd15f3_cb3c_4fda_b599_ac7410bffac2 </ci>
      </apply>
    </apply>
    <apply>
      <plus/>
      <cn type="integer"> 1 </cn>
      <apply>
        <divide/>
        <ci> mw34d9dd05_1772_4f59_ae50_4d6ef1f63225 </ci>
        <ci> mwaea44262_a9e9_4810_9d88_b4d97c605854 </ci>
      </apply>
    </apply>
  </apply>

```
          </apply>
        </apply>
      </math>
    </kineticLaw>
  </reaction>
  <reaction id="mwc12e40ca_3d7e_4a15_8dc2_c86cf23b62a8" name="PP_2c (phosphoglycolate phosphatase)" reversible="false" fast="false">
    <listOfReactants>
      <speciesReference species="mw69137d0b_f529_4e9a_b7ef_33dffd020665"/>
    </listOfReactants>
    <listOfProducts>
      <speciesReference species="mw62b862f7_f0c7_48df_895b_319e5b84b577"/>
      <speciesReference species="mw34d9dd05_1772_4f59_ae50_4d6ef1f63225"/>
    </listOfProducts>
    <kineticLaw>
      <math xmlns="http://www.w3.org/1998/Math/MathML">
        <apply>
          <divide/>
          <apply>
            <times/>
            <ci> mw95d51cdd_5101_48f8_ad60_b5c37d37c5de </ci>
            <ci> mw69137d0b_f529_4e9a_b7ef_33dffd020665 </ci>
          </apply>
          <apply>
            <plus/>
            <ci> mw69137d0b_f529_4e9a_b7ef_33dffd020665 </ci>
            <apply>
              <times/>
              <ci> mw356de434_dd62_4e14_b35b_d2e87ddcc2d8 </ci>
              <apply>
                <plus/>
```

```
              <cn type="integer"> 1 </cn>
              <apply>
                <divide/>
                <ci> mw62b862f7_f0c7_48df_895b_319e5b84b577 </ci>
                <ci> mw55bd15f3_cb3c_4fda_b599_ac7410bffac2 </ci>
              </apply>
            </apply>
            <apply>
              <plus/>
              <cn type="integer"> 1 </cn>
              <apply>
                <divide/>
                <ci> mw34d9dd05_1772_4f59_ae50_4d6ef1f63225 </ci>
                <ci> mwaea44262_a9e9_4810_9d88_b4d97c605854 </ci>
              </apply>
            </apply>
          </apply>
        </apply>
      </apply>
    </math>
  </kineticLaw>
</reaction>
<reaction id="mwab700478_106c_473c_b40b_4afc4d160764" name="PP_3 (glycolate oxidase)"
reversible="false" fast="false">
  <annotation>
    <COPASI xmlns="http://www.copasi.org/static/sbml">
      <rdf:RDF xmlns:dcterms="http://purl.org/dc/terms/"
xmlns:rdf="http://www.w3.org/1999/02/22-rdf-syntax-ns#">
        <rdf:Description rdf:about="#COPASI71">
          <dcterms:created>
            <rdf:Description>
              <dcterms:W3CDTF>2011-05-10T00:12:50Z</dcterms:W3CDTF>
```

```
          </rdf:Description>
        </dcterms:created>
      </rdf:Description>
    </rdf:RDF>
  </COPASI>
 </annotation>
 <listOfReactants>
  <speciesReference species="mw62b862f7_f0c7_48df_895b_319e5b84b577"/>
 </listOfReactants>
 <listOfProducts>
  <speciesReference species="mwa60ca2e1_0142_4294_8726_6b633c50a6bf"/>
 </listOfProducts>
 <kineticLaw>
  <math xmlns="http://www.w3.org/1998/Math/MathML">
   <apply>
    <divide/>
    <apply>
     <times/>
     <ci> mw0a05a9a7_f8e0_4216_9a36_0a20398d0fa7 </ci>
     <ci> mw62b862f7_f0c7_48df_895b_319e5b84b577 </ci>
    </apply>
    <apply>
     <plus/>
     <ci> mw62b862f7_f0c7_48df_895b_319e5b84b577 </ci>
     <ci> mw97764192_3b51_4a4b_b6af_86b67f201f3d </ci>
    </apply>
   </apply>
  </math>
 </kineticLaw>
</reaction>
```

<reaction id="mw8b4d06b9_c0d0_46fc_aae5_74c506bd0980" name="PP_4 (serineglyoxylate transaminase)" fast="false">

<annotation>

<COPASI xmlns="http://www.copasi.org/static/sbml">

<rdf:RDF xmlns:dcterms="http://purl.org/dc/terms/" xmlns:rdf="http://www.w3.org/1999/02/22-rdf-syntax-ns#">

<rdf:Description rdf:about="#COPASI72">

<dcterms:created>

<rdf:Description>

<dcterms:W3CDTF>2011-05-10T00:16:51Z</dcterms:W3CDTF>

</rdf:Description>

</dcterms:created>

</rdf:Description>

</rdf:RDF>

</COPASI>

</annotation>

<listOfReactants>

<speciesReference species="mwa60ca2e1_0142_4294_8726_6b633c50a6bf"/>

<speciesReference species="mw4bff44c2_7520_41bc_80fb_bd781f8b6503"/>

</listOfReactants>

<listOfProducts>

<speciesReference species="mw881e6414_8bc5_4885_b415_3467a94c3d32"/>

<speciesReference species="mw7ee6ca0b_cc39_45ef_a044_36cfeb983c0b"/>

</listOfProducts>

<kineticLaw>

<math xmlns="http://www.w3.org/1998/Math/MathML">

<apply>

<divide/>

<apply>

<times/>

<ci> mw928bed3b_b2b3_42b5_89f3_a5090846accf </ci>

<apply>

```
    <minus/>
    <apply>
      <times/>
      <ci> mwa60ca2e1_0142_4294_8726_6b633c50a6bf </ci>
      <ci> mw4bff44c2_7520_41bc_80fb_bd781f8b6503 </ci>
    </apply>
    <apply>
      <divide/>
      <apply>
        <times/>
        <ci> mw881e6414_8bc5_4885_b415_3467a94c3d32 </ci>
        <ci> mw7ee6ca0b_cc39_45ef_a044_36cfeb983c0b </ci>
      </apply>
      <ci> mw1270f895_d29a_44e6_82fb_b7e4a5b90172 </ci>
    </apply>
  </apply>
</apply>
<apply>
  <times/>
  <apply>
    <plus/>
    <ci> mwa60ca2e1_0142_4294_8726_6b633c50a6bf </ci>
    <ci> mwfe4b8452_607e_443f_9448_4fe832048e5d </ci>
  </apply>
  <apply>
    <plus/>
    <ci> mw4bff44c2_7520_41bc_80fb_bd781f8b6503 </ci>
    <apply>
      <times/>
      <ci> mwffd5861f_1df3_4e1b_b6f8_ca0b6da59319 </ci>
      <apply>
```

```
              <plus/>
              <cn type="integer"> 1 </cn>
              <apply>
                <divide/>
                <ci> mw7ee6ca0b_cc39_45ef_a044_36cfeb983c0b </ci>
                <ci> mw3554f65b_6b0b_4025_be9b_44db5077d8e3 </ci>
              </apply>
            </apply>
          </apply>
        </apply>
      </apply>
    </apply>
  </math>
 </kineticLaw>
</reaction>
<reaction id="mw9bb434c4_9aac_4358_b932_f36e436ceb51" name="PP_5 (serine
hydroxymethyltransferase)" reversible="false" fast="false">
 <annotation>
  <COPASI xmlns="http://www.copasi.org/static/sbml">
   <rdf:RDF xmlns:dcterms="http://purl.org/dc/terms/"
xmlns:rdf="http://www.w3.org/1999/02/22-rdf-syntax-ns#">
    <rdf:Description rdf:about="#COPASI73">
     <dcterms:created>
      <rdf:Description>
       <dcterms:W3CDTF>2011-05-10T00:22:17Z</dcterms:W3CDTF>
      </rdf:Description>
     </dcterms:created>
    </rdf:Description>
   </rdf:RDF>
  </COPASI>
 </annotation>
 <listOfReactants>
```

```
<speciesReference species="mw7ee6ca0b_cc39_45ef_a044_36cfeb983c0b"
stoichiometry="2"/>
</listOfReactants>
<listOfProducts>
<speciesReference species="mw4bff44c2_7520_41bc_80fb_bd781f8b6503"/>
</listOfProducts>
<kineticLaw>
<math xmlns="http://www.w3.org/1998/Math/MathML">
<apply>
<divide/>
<apply>
<times/>
<ci> mw02354494_0fe5_4a28_b05b_89e41b0d76e7 </ci>
<ci> mw7ee6ca0b_cc39_45ef_a044_36cfeb983c0b </ci>
</apply>
<apply>
<plus/>
<ci> mwa0637650_33dc_461f_9c46_982ca7f2e6dd </ci>
<ci> mw7ee6ca0b_cc39_45ef_a044_36cfeb983c0b </ci>
</apply>
</apply>
</math>
<listOfParameters>
<parameter id="mwa0637650_33dc_461f_9c46_982ca7f2e6dd" name="K1" value="6"/>
</listOfParameters>
</kineticLaw>
</reaction>
<reaction id="mw43d57a91_0ced_4dd8_ad61_f31545f964f8" name="PP_6 (hydroxypyruvate
reductase)" reversible="false" fast="false">
<annotation>
<COPASI xmlns="http://www.copasi.org/static/sbml">
```

```
<rdf:RDF xmlns:dcterms="http://purl.org/dc/terms/"
xmlns:rdf="http://www.w3.org/1999/02/22-rdf-syntax-ns#">
  <rdf:Description rdf:about="#COPASI74">
    <dcterms:created>
      <rdf:Description>
        <dcterms:W3CDTF>2011-05-10T01:04:31Z</dcterms:W3CDTF>
      </rdf:Description>
    </dcterms:created>
  </rdf:Description>
</rdf:RDF>
</COPASI>
</annotation>
<listOfReactants>
  <speciesReference species="mw881e6414_8bc5_4885_b415_3467a94c3d32"/>
</listOfReactants>
<listOfProducts>
  <speciesReference species="mw924156fa_35b2_403e_891e_fbc962e777d9"/>
</listOfProducts>
<kineticLaw>
  <math xmlns="http://www.w3.org/1998/Math/MathML">
    <apply>
      <divide/>
      <apply>
        <times/>
        <ci> mwcc8ed7db_a4f4_435d_9ed4_449f30e69058 </ci>
        <apply>
          <minus/>
          <ci> mw881e6414_8bc5_4885_b415_3467a94c3d32 </ci>
          <apply>
            <divide/>
            <ci> mw924156fa_35b2_403e_891e_fbc962e777d9 </ci>
```

<ci> mwcbeed6ea_4576_4b4a_950a_7b8ba162bb5b </ci>

</apply>

</apply>

</apply>

<apply>

<plus/>

<ci> mw881e6414_8bc5_4885_b415_3467a94c3d32 </ci>

<apply>

<times/>

<ci> mw56c6a02a_7634_401b_b487_a5ee2fdaa884 </ci>

<apply>

<plus/>

<cn type="integer"> 1 </cn>

<apply>

<divide/>

<ci> mw881e6414_8bc5_4885_b415_3467a94c3d32 </ci>

<ci> mw34409210_66ed_415b_a829_f6dbf66524f4 </ci>

</apply>

</apply>

</apply>

</apply>

</apply>

</math>

</kineticLaw>

</reaction>

<reaction id="mwc98b94b6_b43f_4529_ad71_cfc752845c2b" name="PP_7 (glycerate kinase)" reversible="false" fast="false">

<annotation>

<COPASI xmlns="http://www.copasi.org/static/sbml">

<rdf:RDF xmlns:dcterms="http://purl.org/dc/terms/" xmlns:rdf="http://www.w3.org/1999/02/22-rdf-syntax-ns#">

<rdf:Description rdf:about="#COPASI75">

```
        <dcterms:created>
          <rdf:Description>
            <dcterms:W3CDTF>2011-05-10T01:09:19Z</dcterms:W3CDTF>
          </rdf:Description>
        </dcterms:created>
      </rdf:Description>
    </rdf:RDF>
  </COPASI>
</annotation>
<listOfReactants>
  <speciesReference species="mw924156fa_35b2_403e_891e_fbc962e777d9"/>
  <speciesReference species="mwa083eb39_db7c_4dc7_a1b7_5d06c9120757"/>
</listOfReactants>
<listOfProducts>
  <speciesReference species="mw1e1e1695_f297_4f58_906b_5f76ada38e61"/>
  <speciesReference species="mw4b092c2e_9b30_46e1_85f7_9bb39d9b10aa"/>
</listOfProducts>
<kineticLaw>
  <math xmlns="http://www.w3.org/1998/Math/MathML">
    <apply>
      <divide/>
      <apply>
        <times/>
        <ci> mw557b4a1b_a1b0_4840_a606_ec763580e90c </ci>
        <apply>
          <minus/>
          <apply>
            <times/>
            <ci> mwa083eb39_db7c_4dc7_a1b7_5d06c9120757 </ci>
            <ci> mw924156fa_35b2_403e_891e_fbc962e777d9 </ci>
          </apply>
```

```
      <apply>
        <divide/>
        <apply>
          <times/>
          <ci> mw4b092c2e_9b30_46e1_85f7_9bb39d9b10aa </ci>
          <ci> mw1e1e1695_f297_4f58_906b_5f76ada38e61 </ci>
        </apply>
        <ci> mw4149ab27_a9d5_449b_9996_8c4f87c94e9f </ci>
      </apply>
    </apply>
  </apply>
  <apply>
    <times/>
    <apply>
      <plus/>
      <ci> mwa083eb39_db7c_4dc7_a1b7_5d06c9120757 </ci>
      <apply>
        <times/>
        <ci> mw5bf928d1_2a99_48f9_ac13_ecba1a3e04ad </ci>
        <apply>
          <plus/>
          <cn type="integer"> 1 </cn>
          <apply>
            <divide/>
            <ci> mw1e1e1695_f297_4f58_906b_5f76ada38e61 </ci>
            <ci> mw2ad9e2a3_9d3b_4185_8007_b877627c8032 </ci>
          </apply>
        </apply>
      </apply>
    </apply>
    <apply>
```

```
          <plus/>
          <ci> mw924156fa_35b2_403e_891e_fbc962e777d9 </ci>
          <ci> mwa21e7932_75e2_471d_9fb1_bcb876fbbd76 </ci>
        </apply>
      </apply>
    </apply>
  </math>
 </kineticLaw>
</reaction>
<reaction id="mwc13bcaab_9d56_4d41_b70f_1cf81de67123" name="GC_1 (tartronate
semialdehyde synthase)" reversible="false" fast="false">
 <annotation>
  <COPASI xmlns="http://www.copasi.org/static/sbml">
   <rdf:RDF xmlns:dcterms="http://purl.org/dc/terms/"
xmlns:rdf="http://www.w3.org/1999/02/22-rdf-syntax-ns#">
    <rdf:Description rdf:about="#COPASI82">
     <dcterms:created>
      <rdf:Description>
       <dcterms:W3CDTF>2011-06-15T16:39:15Z</dcterms:W3CDTF>
      </rdf:Description>
     </dcterms:created>
    </rdf:Description>
   </rdf:RDF>
  </COPASI>
 </annotation>
 <listOfReactants>
  <speciesReference species="mwa60ca2e1_0142_4294_8726_6b633c50a6bf"
stoichiometry="2"/>
 </listOfReactants>
 <listOfProducts>
  <speciesReference species="mw142bb566_7067_4fc2_b957_dc196c94324a"/>
 </listOfProducts>
```

```
<kineticLaw>
  <math xmlns="http://www.w3.org/1998/Math/MathML">
    <apply>
      <divide/>
      <apply>
        <times/>
        <ci> mw94b56681_0cac_45dc_b68e_1fda032f53b9 </ci>
        <ci> mwa60ca2e1_0142_4294_8726_6b633c50a6bf </ci>
      </apply>
      <apply>
        <plus/>
        <ci> mwad7edeb0_57d8_44dc_af42_e15e631d77bf </ci>
        <ci> mwa60ca2e1_0142_4294_8726_6b633c50a6bf </ci>
      </apply>
    </apply>
  </math>
</kineticLaw>
</reaction>
<reaction id="mwbbf30da0_64f4_4d31_b874_807862d3d277" name="GC_2 (tartronate semialdehyde reductase)" reversible="false" fast="false">
<annotation>
  <COPASI xmlns="http://www.copasi.org/static/sbml">
    <rdf:RDF xmlns:dcterms="http://purl.org/dc/terms/" xmlns:rdf="http://www.w3.org/1999/02/22-rdf-syntax-ns#">
      <rdf:Description rdf:about="#COPASI84">
        <dcterms:created>
          <rdf:Description>
            <dcterms:W3CDTF>2011-06-15T16:40:54Z</dcterms:W3CDTF>
          </rdf:Description>
        </dcterms:created>
      </rdf:Description>
    </rdf:RDF>
```

```
      </COPASI>
     </annotation>
     <listOfReactants>
      <speciesReference species="mw142bb566_7067_4fc2_b957_dc196c94324a"/>
     </listOfReactants>
     <listOfProducts>
      <speciesReference species="mw924156fa_35b2_403e_891e_fbc962e777d9"/>
     </listOfProducts>
     <kineticLaw>
      <math xmlns="http://www.w3.org/1998/Math/MathML">
       <apply>
        <divide/>
        <apply>
         <times/>
         <ci> mwc67e78e3_63c8_4314_ae67_03f4ba59bc51 </ci>
         <ci> mw142bb566_7067_4fc2_b957_dc196c94324a </ci>
        </apply>
        <apply>
         <plus/>
         <ci> mwdc293431_8b07_49fe_8303_4cb1a50e1b25 </ci>
         <ci> mw142bb566_7067_4fc2_b957_dc196c94324a </ci>
        </apply>
       </apply>
      </math>
     </kineticLaw>
    </reaction>
    <reaction id="mwce94c3a1_be92_48d8_b17c_ae005035f92c" name="OX_1 (glyoxylate oxidase)"
reversible="false" fast="false">
     <annotation>
      <COPASI xmlns="http://www.copasi.org/static/sbml">
       <rdf:RDF xmlns:dcterms="http://purl.org/dc/terms/"
xmlns:rdf="http://www.w3.org/1999/02/22-rdf-syntax-ns#">
```

```
      <rdf:Description rdf:about="#COPASI85">
        <dcterms:created>
          <rdf:Description>
            <dcterms:W3CDTF>2011-06-28T01:12:01Z</dcterms:W3CDTF>
          </rdf:Description>
        </dcterms:created>
      </rdf:Description>
    </rdf:RDF>
  </COPASI>
</annotation>
<listOfReactants>
  <speciesReference species="mwa60ca2e1_0142_4294_8726_6b633c50a6bf"/>
</listOfReactants>
<listOfProducts>
  <speciesReference species="mwa86b050a_b141_4f76_9597_674909286fc9"/>
</listOfProducts>
<kineticLaw>
  <math xmlns="http://www.w3.org/1998/Math/MathML">
    <apply>
      <divide/>
      <apply>
        <times/>
        <ci> mw6870894e_d5a3_4bb2_8096_8de1303cdbb9 </ci>
        <ci> mwa60ca2e1_0142_4294_8726_6b633c50a6bf </ci>
      </apply>
      <apply>
        <plus/>
        <ci> mw663cb8cd_1897_4185_98e4_23ce87526c47 </ci>
        <ci> mwa60ca2e1_0142_4294_8726_6b633c50a6bf </ci>
      </apply>
    </apply>
```

```
          </math>
        </kineticLaw>
      </reaction>
      <reaction id="mw10426607_7ee9_4230_9c12_143f8ddb7a29" name="GLY_1 alpha
(phosphofructokinase)" reversible="false" fast="false">
        <listOfReactants>
          <speciesReference species="mwf1237a2f_b6c2_4f60_aec2_f24eaff9e01a"/>
          <speciesReference species="mwa083eb39_db7c_4dc7_a1b7_5d06c9120757"/>
        </listOfReactants>
        <listOfProducts>
          <speciesReference species="mw001a645f_0641_44d7_9a48_6a15ea7e4a51"/>
          <speciesReference species="mw4b092c2e_9b30_46e1_85f7_9bb39d9b10aa"/>
        </listOfProducts>
        <kineticLaw>
          <math xmlns="http://www.w3.org/1998/Math/MathML">
            <apply>
              <divide/>
              <apply>
                <divide/>
                <apply>
                  <times/>
                  <ci> mwe11dcb8e_d7c1_423b_bbe4_5da671bd64ca </ci>
                  <ci> mwf1237a2f_b6c2_4f60_aec2_f24eaff9e01a </ci>
                  <ci> mwa083eb39_db7c_4dc7_a1b7_5d06c9120757 </ci>
                </apply>
                <apply>
                  <times/>
                  <ci> mw921c626f_edc9_441b_8547_3c7edacfc4e3 </ci>
                  <ci> mw9acd4aeb_fdb0_4a5e_833f_e3946722d810 </ci>
                </apply>
              </apply>
```

```
            <apply>
              <plus/>
              <cn type="integer"> 1 </cn>
              <apply>
                <times/>
                <apply>
                  <divide/>
                  <ci> mwa083eb39_db7c_4dc7_a1b7_5d06c9120757 </ci>
                  <ci> mw9acd4aeb_fdb0_4a5e_833f_e3946722d810 </ci>
                </apply>
                <apply>
                  <divide/>
                  <ci> mwf1237a2f_b6c2_4f60_aec2_f24eaff9e01a </ci>
                  <ci> mw921c626f_edc9_441b_8547_3c7edacfc4e3 </ci>
                </apply>
              </apply>
            </apply>
          </apply>
        </math>
      </kineticLaw>
    </reaction>
    <reaction id="mwc244d55e_17dd_40d7_935e_84e10aed5480" name="GLY_1 beta
(phosphofructokinase)" reversible="false" fast="false">
      <listOfReactants>
        <speciesReference species="mwf1237a2f_b6c2_4f60_aec2_f24eaff9e01a"/>
        <speciesReference species="mwa083eb39_db7c_4dc7_a1b7_5d06c9120757"/>
      </listOfReactants>
      <listOfProducts>
        <speciesReference species="mw001a645f_0641_44d7_9a48_6a15ea7e4a51"/>
        <speciesReference species="mw4b092c2e_9b30_46e1_85f7_9bb39d9b10aa"/>
      </listOfProducts>
```

```
<kineticLaw>
  <math xmlns="http://www.w3.org/1998/Math/MathML">
    <apply>
      <divide/>
      <apply>
        <divide/>
        <apply>
          <times/>
          <ci> mw4c067e5e_f048_4dc4_bc7d_8e95972e89b1 </ci>
          <ci> mwf1237a2f_b6c2_4f60_aec2_f24eaff9e01a </ci>
          <ci> mwa083eb39_db7c_4dc7_a1b7_5d06c9120757 </ci>
        </apply>
        <apply>
          <times/>
          <ci> mw5d3b7d4f_cb37_4189_808f_9b316593f3fd </ci>
          <ci> mw4f29d2d9_0857_4db2_880a_535c67780929 </ci>
        </apply>
      </apply>
      <apply>
        <plus/>
        <cn type="integer"> 1 </cn>
        <apply>
          <times/>
          <apply>
            <divide/>
            <ci> mwa083eb39_db7c_4dc7_a1b7_5d06c9120757 </ci>
            <ci> mw4f29d2d9_0857_4db2_880a_535c67780929 </ci>
          </apply>
          <apply>
            <divide/>
            <ci> mwf1237a2f_b6c2_4f60_aec2_f24eaff9e01a </ci>
```

<ci> mw5d3b7d4f_cb37_4189_808f_9b316593f3fd </ci>
</apply>
</apply>
</apply>
</apply>
</math>
</kineticLaw>
</reaction>
<reaction id="mw35210c27_18e7_4e96_885c_5809ff149d96" name="GLY_2 (glyceraldehyde 3-phosphate dehydrogenase)" reversible="false" fast="false">
<listOfReactants>
<speciesReference species="mw98624f9d_a464_456c_9a02_026d96612aff"/>
<speciesReference species="mwf5dc5c05_4d36_4ea4_8ee0_6a1cfd2ae49b"/>
</listOfReactants>
<listOfProducts>
<speciesReference species="mw0c5a9637_94b1_47f0_87f9_8729e75ae8de"/>
<speciesReference species="mw4e68b2da_34ac_4484_9cf5_03f0f6d9abe9"/>
</listOfProducts>
<kineticLaw>
<math xmlns="http://www.w3.org/1998/Math/MathML">
<apply>
<divide/>
<apply>
<times/>
<ci> mw30cb04f1_447a_49ab_9566_af33f6c68f02 </ci>
<ci> mw98624f9d_a464_456c_9a02_026d96612aff </ci>
<ci> mwf5dc5c05_4d36_4ea4_8ee0_6a1cfd2ae49b </ci>
</apply>
<apply>
<times/>
<apply>

```
          <plus/>
          <cn type="integer"> 1 </cn>
          <apply>
            <divide/>
            <ci> mw98624f9d_a464_456c_9a02_026d96612aff </ci>
            <ci> mw2b15c7a2_60bd_4539_bbca_ea2887c83c7e </ci>
          </apply>
          <apply>
            <divide/>
            <ci> mw1e1e1695_f297_4f58_906b_5f76ada38e61 </ci>
            <ci> mw66c149b7_107b_4f1e_8ba3_e998a2eb4341 </ci>
          </apply>
        </apply>
        <apply>
          <plus/>
          <cn type="integer"> 1 </cn>
          <apply>
            <divide/>
            <ci> mwf5dc5c05_4d36_4ea4_8ee0_6a1cfd2ae49b </ci>
            <ci> mw42d232ce_870c_4c6c_80e1_b80db63c24dc </ci>
          </apply>
          <apply>
            <divide/>
            <ci> mw4e68b2da_34ac_4484_9cf5_03f0f6d9abe9 </ci>
            <ci> mw3f587528_6a1e_45cf_85a1_c83170fa3db2 </ci>
          </apply>
        </apply>
      </apply>
    </apply>
  </math>
</kineticLaw>
```

```
    </reaction>
    <reaction id="mw2eaba045_d759_4b18_8805_fc100736f8d3" name="GLY_3a (phosphoglycerate mutase)" fast="false">
      <listOfReactants>
        <speciesReference species="mw1e1e1695_f297_4f58_906b_5f76ada38e61"/>
      </listOfReactants>
      <listOfProducts>
        <speciesReference species="mwcd2b43d1_7092_45b9_af63_c926207ad771"/>
      </listOfProducts>
      <kineticLaw>
        <math xmlns="http://www.w3.org/1998/Math/MathML">
          <apply>
            <divide/>
            <apply>
              <times/>
              <ci> mw4354fd87_eec3_45ff_ba09_8e66f6cec0d4 </ci>
              <apply>
                <minus/>
                <ci> mw1e1e1695_f297_4f58_906b_5f76ada38e61 </ci>
                <apply>
                  <divide/>
                  <ci> mwcd2b43d1_7092_45b9_af63_c926207ad771 </ci>
                  <ci> mwfa3dac90_b557_4ea0_950c_b2e6cea1f624 </ci>
                </apply>
              </apply>
            </apply>
            <apply>
              <times/>
              <ci> mw407bee91_c458_4b42_ad72_7dea8c0f84b8 </ci>
              <apply>
                <plus/>
```

```
<cn type="integer"> 1 </cn>
<apply>
  <divide/>
  <ci> mw1e1e1695_f297_4f58_906b_5f76ada38e61 </ci>
  <ci> mw407bee91_c458_4b42_ad72_7dea8c0f84b8 </ci>
</apply>
<apply>
  <divide/>
  <ci> mwcd2b43d1_7092_45b9_af63_c926207ad771 </ci>
  <ci> mw2091e1f5_0cd3_4d23_9dcf_9f54ae671686 </ci>
</apply>
</apply>
</apply>
</apply>
</math>
</kineticLaw>
</reaction>
<reaction id="mwec1e61bf_385d_483c_87a8_4d049d98e5f5" name="GLY_3b (phosphoglycerate mutase)" fast="false">
<listOfReactants>
  <speciesReference species="mw1e1e1695_f297_4f58_906b_5f76ada38e61"/>
</listOfReactants>
<listOfProducts>
  <speciesReference species="mwcd2b43d1_7092_45b9_af63_c926207ad771"/>
</listOfProducts>
<kineticLaw>
  <math xmlns="http://www.w3.org/1998/Math/MathML">
    <apply>
      <divide/>
      <apply>
        <times/>
```

```
    &lt;ci&gt; mw9e3b0877_c7fe_4a60_886f_4c0b97f63ef9 &lt;/ci&gt;
    &lt;apply&gt;
      &lt;minus/&gt;
      &lt;ci&gt; mw1e1e1695_f297_4f58_906b_5f76ada38e61 &lt;/ci&gt;
      &lt;apply&gt;
        &lt;divide/&gt;
        &lt;ci&gt; mwcd2b43d1_7092_45b9_af63_c926207ad771 &lt;/ci&gt;
        &lt;ci&gt; mwfa3dac90_b557_4ea0_950c_b2e6cea1f624 &lt;/ci&gt;
      &lt;/apply&gt;
    &lt;/apply&gt;
  &lt;/apply&gt;
  &lt;apply&gt;
    &lt;times/&gt;
    &lt;ci&gt; mw099e3275_3aed_4b7c_abd7_1d978c4681d1 &lt;/ci&gt;
    &lt;apply&gt;
      &lt;plus/&gt;
      &lt;cn type="integer"&gt; 1 &lt;/cn&gt;
      &lt;apply&gt;
        &lt;divide/&gt;
        &lt;ci&gt; mw1e1e1695_f297_4f58_906b_5f76ada38e61 &lt;/ci&gt;
        &lt;ci&gt; mw099e3275_3aed_4b7c_abd7_1d978c4681d1 &lt;/ci&gt;
      &lt;/apply&gt;
      &lt;apply&gt;
        &lt;divide/&gt;
        &lt;ci&gt; mwcd2b43d1_7092_45b9_af63_c926207ad771 &lt;/ci&gt;
        &lt;ci&gt; mw51232471_96c5_44a5_9906_91a4af9717da &lt;/ci&gt;
      &lt;/apply&gt;
    &lt;/apply&gt;
  &lt;/apply&gt;
&lt;/apply&gt;
&lt;/math&gt;
```

```xml
    </kineticLaw>
   </reaction>
   <reaction id="mwcf3943b7_1f74_4109_9555_eaa961abb641" name="GLY_3c (phosphoglycerate mutase)" fast="false">
    <listOfReactants>
     <speciesReference species="mw1e1e1695_f297_4f58_906b_5f76ada38e61"/>
    </listOfReactants>
    <listOfProducts>
     <speciesReference species="mwcd2b43d1_7092_45b9_af63_c926207ad771"/>
    </listOfProducts>
    <kineticLaw>
     <math xmlns="http://www.w3.org/1998/Math/MathML">
      <apply>
       <divide/>
       <apply>
        <times/>
        <ci> mwa466c9c9_3297_437c_baed_0dce17c19743 </ci>
        <apply>
         <minus/>
         <ci> mw1e1e1695_f297_4f58_906b_5f76ada38e61 </ci>
         <apply>
          <divide/>
          <ci> mwcd2b43d1_7092_45b9_af63_c926207ad771 </ci>
          <ci> mwfa3dac90_b557_4ea0_950c_b2e6cea1f624 </ci>
         </apply>
        </apply>
       </apply>
       <apply>
        <times/>
        <ci> mwefaeb6d1_a2ae_41cb_b478_f28cb1be6a7c </ci>
        <apply>
```

```
            <plus/>
            <cn type="integer"> 1 </cn>
            <apply>
              <divide/>
              <ci> mw1e1e1695_f297_4f58_906b_5f76ada38e61 </ci>
              <ci> mwefaeb6d1_a2ae_41cb_b478_f28cb1be6a7c </ci>
            </apply>
            <apply>
              <divide/>
              <ci> mwcd2b43d1_7092_45b9_af63_c926207ad771 </ci>
              <ci> mw93985682_472b_4e3b_895b_705c1b0929b3 </ci>
            </apply>
          </apply>
        </apply>
      </apply>
    </math>
  </kineticLaw>
</reaction>
<reaction id="mw73add333_bc63_4059_9453_bf39fa97a4ea" name="GLY_4 (enolase)"
fast="false">
  <annotation>
    <COPASI xmlns="http://www.copasi.org/static/sbml">
      <rdf:RDF xmlns:dcterms="http://purl.org/dc/terms/"
xmlns:rdf="http://www.w3.org/1999/02/22-rdf-syntax-ns#">
        <rdf:Description rdf:about="#COPASI78">
          <dcterms:created>
            <rdf:Description>
              <dcterms:W3CDTF>2011-05-10T15:12:27Z</dcterms:W3CDTF>
            </rdf:Description>
          </dcterms:created>
        </rdf:Description>
      </rdf:RDF>
```

```
 </COPASI>
</annotation>
<listOfReactants>
 <speciesReference species="mwcd2b43d1_7092_45b9_af63_c926207ad771"/>
</listOfReactants>
<listOfProducts>
 <speciesReference species="mwc3b39320_8ede_4d49_ba08_c569fbe1474d"/>
</listOfProducts>
<kineticLaw>
 <math xmlns="http://www.w3.org/1998/Math/MathML">
  <apply>
   <divide/>
   <apply>
    <times/>
    <ci> mw1560b05b_911d_4737_8105_b669df533ad3 </ci>
    <apply>
     <minus/>
     <ci> mwcd2b43d1_7092_45b9_af63_c926207ad771 </ci>
     <apply>
      <divide/>
      <ci> mwc3b39320_8ede_4d49_ba08_c569fbe1474d </ci>
      <ci> mw60d16eba_0bba_4387_94f0_ddda983743a2 </ci>
     </apply>
    </apply>
   </apply>
   <apply>
    <times/>
    <ci> mw48edc399_e14c_4e4e_8fd5_27aa7a43e91a </ci>
    <apply>
     <plus/>
     <cn type="integer"> 1 </cn>
```

```
          <apply>
            <divide/>
            <ci> mwcd2b43d1_7092_45b9_af63_c926207ad771 </ci>
            <ci> mw48edc399_e14c_4e4e_8fd5_27aa7a43e91a </ci>
          </apply>
          <apply>
            <divide/>
            <ci> mwc3b39320_8ede_4d49_ba08_c569fbe1474d </ci>
            <ci> mw28cc925f_1ec1_40e8_a64d_bacb34b7b472 </ci>
          </apply>
         </apply>
        </apply>
       </apply>
      </math>
     </kineticLaw>
    </reaction>
    <reaction id="mw53e07cb5_b951_4776_bc61_2db95b9f767f" name="PKET1a (phosphoketolase)" fast="false">
     <listOfReactants>
      <speciesReference species="mwf1237a2f_b6c2_4f60_aec2_f24eaff9e01a"/>
      <speciesReference species="mw98624f9d_a464_456c_9a02_026d96612aff"/>
      <speciesReference species="mwb2da70f3_0970_4374_bdf2_4a3914afa142"/>
     </listOfReactants>
     <listOfProducts>
      <speciesReference species="mwe1915c23_bc54_4444_a6b6_afb9f835d8cf"/>
      <speciesReference species="mw6c1ca071_aba1_4113_ace9_3d429d27bc60"/>
      <speciesReference species="mw98624f9d_a464_456c_9a02_026d96612aff"/>
      <speciesReference species="mwb2da70f3_0970_4374_bdf2_4a3914afa142"/>
     </listOfProducts>
     <kineticLaw>
      <math xmlns="http://www.w3.org/1998/Math/MathML">
```

```
<apply>
 <divide/>
 <apply>
  <divide/>
  <apply>
   <times/>
   <ci> mw14672e46_52f5_4ab7_bff1_4b749b587f1d </ci>
   <apply>
    <minus/>
    <ci> mwf1237a2f_b6c2_4f60_aec2_f24eaff9e01a </ci>
    <apply>
     <divide/>
     <apply>
      <times/>
      <ci> mwe1915c23_bc54_4444_a6b6_afb9f835d8cf </ci>
      <ci> mw6c1ca071_aba1_4113_ace9_3d429d27bc60 </ci>
     </apply>
     <ci> mwac1b2180_f1af_4819_9233_94af7fae5fe8 </ci>
    </apply>
   </apply>
  </apply>
  <ci> mw95025ff8_a096_4b4e_b30a_c86490520d3c </ci>
 </apply>
 <apply>
  <times/>
  <apply>
   <plus/>
   <cn type="integer"> 1 </cn>
   <apply>
    <divide/>
    <ci> mwf1237a2f_b6c2_4f60_aec2_f24eaff9e01a </ci>
```

```
    <ci> mw95025ff8_a096_4b4e_b30a_c86490520d3c </ci>
  </apply>
  <apply>
    <divide/>
    <ci> mw6c1ca071_aba1_4113_ace9_3d429d27bc60 </ci>
    <ci> mw35d79455_d586_4f7e_b53d_b9b05403c18f </ci>
  </apply>
</apply>
<apply>
  <plus/>
  <cn type="integer"> 1 </cn>
  <apply>
    <divide/>
    <ci> mwe1915c23_bc54_4444_a6b6_afb9f835d8cf </ci>
    <ci> mw29daf52a_3537_43f2_b889_86b8d087fab9 </ci>
  </apply>
</apply>
<apply>
  <plus/>
  <cn type="integer"> 1 </cn>
  <apply>
    <divide/>
    <ci> mwb2da70f3_0970_4374_bdf2_4a3914afa142 </ci>
    <ci> mwcc132bf2_7c6b_4cbb_b3e3_caddab999b30 </ci>
  </apply>
  <apply>
    <divide/>
    <ci> mw98624f9d_a464_456c_9a02_026d96612aff </ci>
    <ci> mw4231ba04_0406_4368_925a_599001509b3f </ci>
  </apply>
</apply>
```

```
      </apply>
     </apply>
    </math>
   </kineticLaw>
  </reaction>
  <reaction id="mw876830e7_f9b8_4da1_9aef_47183980cc63" name="PKET1b" fast="false">
   <listOfReactants>
    <speciesReference species="mwb2da70f3_0970_4374_bdf2_4a3914afa142"/>
    <speciesReference species="mwf1237a2f_b6c2_4f60_aec2_f24eaff9e01a"/>
    <speciesReference species="mw6c1ca071_aba1_4113_ace9_3d429d27bc60"/>
   </listOfReactants>
   <listOfProducts>
    <speciesReference species="mw98624f9d_a464_456c_9a02_026d96612aff"/>
    <speciesReference species="mwe1915c23_bc54_4444_a6b6_afb9f835d8cf"/>
    <speciesReference species="mw6c1ca071_aba1_4113_ace9_3d429d27bc60"/>
    <speciesReference species="mwf1237a2f_b6c2_4f60_aec2_f24eaff9e01a"/>
   </listOfProducts>
   <kineticLaw>
    <math xmlns="http://www.w3.org/1998/Math/MathML">
     <apply>
      <divide/>
      <apply>
       <divide/>
       <apply>
        <times/>
        <ci> mwe6cc0f80_5d7e_478d_8df3_785b611461a8 </ci>
        <apply>
         <minus/>
         <ci> mwb2da70f3_0970_4374_bdf2_4a3914afa142 </ci>
         <apply>
          <divide/>
```

```
        <apply>
          <times/>
          <ci> mwe1915c23_bc54_4444_a6b6_afb9f835d8cf </ci>
          <ci> mw98624f9d_a464_456c_9a02_026d96612aff </ci>
        </apply>
        <ci> mwb043257b_34eb_45c4_b4bd_7fadfb6e19c8 </ci>
      </apply>
    </apply>
  </apply>
  <ci> mw94c699e4_fed2_455f_9f56_63394e10e6aa </ci>
</apply>
<apply>
  <times/>
  <apply>
    <plus/>
    <cn type="integer"> 1 </cn>
    <apply>
      <divide/>
      <ci> mwf1237a2f_b6c2_4f60_aec2_f24eaff9e01a </ci>
      <ci> mw08464d63_135a_4871_9108_9a430e7da3c9 </ci>
    </apply>
    <apply>
      <divide/>
      <ci> mw6c1ca071_aba1_4113_ace9_3d429d27bc60 </ci>
      <ci> mwf1cc2080_281e_420a_bea7_9f1ace706f8b </ci>
    </apply>
  </apply>
  <apply>
    <plus/>
    <cn type="integer"> 1 </cn>
    <apply>
```

```
            <divide/>
            <ci> mwe1915c23_bc54_4444_a6b6_afb9f835d8cf </ci>
            <ci> mw07fd01bf_c788_41e8_ad93_f9663c71e731 </ci>
          </apply>
        </apply>
        <apply>
          <plus/>
          <cn type="integer"> 1 </cn>
          <apply>
            <divide/>
            <ci> mwb2da70f3_0970_4374_bdf2_4a3914afa142 </ci>
            <ci> mw94c699e4_fed2_455f_9f56_63394e10e6aa </ci>
          </apply>
          <apply>
            <divide/>
            <ci> mw98624f9d_a464_456c_9a02_026d96612aff </ci>
            <ci> mw4925fe87_c38b_4c63_b257_76e6a00ba813 </ci>
          </apply>
        </apply>
      </apply>
    </apply>
   </math>
  </kineticLaw>
 </reaction>
 <reaction id="mw34869260_574b_422c_8eeb_d748d83020ba" name="PKET2a
(phosphoketolase)" fast="false">
  <listOfReactants>
   <speciesReference species="mwf1237a2f_b6c2_4f60_aec2_f24eaff9e01a"/>
   <speciesReference species="mw98624f9d_a464_456c_9a02_026d96612aff"/>
   <speciesReference species="mwb2da70f3_0970_4374_bdf2_4a3914afa142"/>
  </listOfReactants>
```

```
<listOfProducts>
  <speciesReference species="mwe1915c23_bc54_4444_a6b6_afb9f835d8cf"/>
  <speciesReference species="mw6c1ca071_aba1_4113_ace9_3d429d27bc60"/>
  <speciesReference species="mw98624f9d_a464_456c_9a02_026d96612aff"/>
  <speciesReference species="mwb2da70f3_0970_4374_bdf2_4a3914afa142"/>
</listOfProducts>
<kineticLaw>
  <math xmlns="http://www.w3.org/1998/Math/MathML">
    <apply>
      <divide/>
      <apply>
        <divide/>
        <apply>
          <times/>
          <ci> mwb9289074_0186_4371_aed9_7470cba640dc </ci>
          <apply>
            <minus/>
            <ci> mwf1237a2f_b6c2_4f60_aec2_f24eaff9e01a </ci>
            <apply>
              <divide/>
              <apply>
                <times/>
                <ci> mwe1915c23_bc54_4444_a6b6_afb9f835d8cf </ci>
                <ci> mw6c1ca071_aba1_4113_ace9_3d429d27bc60 </ci>
              </apply>
              <ci> mwac1b2180_f1af_4819_9233_94af7fae5fe8 </ci>
            </apply>
          </apply>
        </apply>
        <ci> mw235e9584_299a_49b0_917e_b62cb998dc8e </ci>
      </apply>
```

```
&lt;apply&gt;
 &lt;times/&gt;
 &lt;apply&gt;
  &lt;plus/&gt;
  &lt;cn type="integer"&gt; 1 &lt;/cn&gt;
  &lt;apply&gt;
   &lt;divide/&gt;
   &lt;ci&gt; mwf1237a2f_b6c2_4f60_aec2_f24eaff9e01a &lt;/ci&gt;
   &lt;ci&gt; mw235e9584_299a_49b0_917e_b62cb998dc8e &lt;/ci&gt;
  &lt;/apply&gt;
  &lt;apply&gt;
   &lt;divide/&gt;
   &lt;ci&gt; mw6c1ca071_aba1_4113_ace9_3d429d27bc60 &lt;/ci&gt;
   &lt;ci&gt; mw1b9a6d90_0834_45b2_80c3_894c8f3806df &lt;/ci&gt;
  &lt;/apply&gt;
 &lt;/apply&gt;
 &lt;apply&gt;
  &lt;plus/&gt;
  &lt;cn type="integer"&gt; 1 &lt;/cn&gt;
  &lt;apply&gt;
   &lt;divide/&gt;
   &lt;ci&gt; mwe1915c23_bc54_4444_a6b6_afb9f835d8cf &lt;/ci&gt;
   &lt;ci&gt; mwffdd6cd4_59fc_4211_a076_bf7bccbb2861 &lt;/ci&gt;
  &lt;/apply&gt;
 &lt;/apply&gt;
 &lt;apply&gt;
  &lt;plus/&gt;
  &lt;cn type="integer"&gt; 1 &lt;/cn&gt;
  &lt;apply&gt;
   &lt;divide/&gt;
   &lt;ci&gt; mwb2da70f3_0970_4374_bdf2_4a3914afa142 &lt;/ci&gt;
```

```
<ci> mwe2c4c957_b4a0_41f4_aacc_9a73304177bf </ci>
</apply>
<apply>
<divide/>
<ci> mw98624f9d_a464_456c_9a02_026d96612aff </ci>
<ci> mwee148dba_6936_4ff5_98e9_3fafc08bf46c </ci>
</apply>
</apply>
</apply>
</apply>
</math>
</kineticLaw>
</reaction>
<reaction id="mwd966360f_cbab_4110_a696_d9871c46d68f" name="PKET2b" fast="false">
<listOfReactants>
<speciesReference species="mwb2da70f3_0970_4374_bdf2_4a3914afa142"/>
<speciesReference species="mwf1237a2f_b6c2_4f60_aec2_f24eaff9e01a"/>
<speciesReference species="mw6c1ca071_aba1_4113_ace9_3d429d27bc60"/>
</listOfReactants>
<listOfProducts>
<speciesReference species="mw98624f9d_a464_456c_9a02_026d96612aff"/>
<speciesReference species="mwe1915c23_bc54_4444_a6b6_afb9f835d8cf"/>
<speciesReference species="mw6c1ca071_aba1_4113_ace9_3d429d27bc60"/>
<speciesReference species="mwf1237a2f_b6c2_4f60_aec2_f24eaff9e01a"/>
</listOfProducts>
<kineticLaw>
<math xmlns="http://www.w3.org/1998/Math/MathML">
<apply>
<divide/>
<apply>
<divide/>
```

<apply>
<times/>
<ci> mwbfebafa8_c3ee_48cc_843e_9a4bad33bfb3 </ci>
<apply>
<minus/>
<ci> mwb2da70f3_0970_4374_bdf2_4a3914afa142 </ci>
<apply>
<divide/>
<apply>
<times/>
<ci> mwe1915c23_bc54_4444_a6b6_afb9f835d8cf </ci>
<ci> mw98624f9d_a464_456c_9a02_026d96612aff </ci>
</apply>
<ci> mwb043257b_34eb_45c4_b4bd_7fadfb6e19c8 </ci>
</apply>
</apply>
</apply>
<ci> mw6aa54137_d905_4ffa_a29c_b192c4901c8f </ci>
</apply>
<apply>
<times/>
<apply>
<plus/>
<cn type="integer"> 1 </cn>
<apply>
<divide/>
<ci> mwf1237a2f_b6c2_4f60_aec2_f24eaff9e01a </ci>
<ci> mw04c1dd77_705d_42f9_a0ad_169227a654aa </ci>
</apply>
<apply>
<divide/>

<ci> mw6c1ca071_aba1_4113_ace9_3d429d27bc60 </ci>
<ci> mw36675533_f3f1_443f_a00a_e9f3ab7b4e43 </ci>
</apply>
</apply>
<apply>
<plus/>
<cn type="integer"> 1 </cn>
<apply>
<divide/>
<ci> mwe1915c23_bc54_4444_a6b6_afb9f835d8cf </ci>
<ci> mw48d5b78e_f2bf_4cae_92f9_9b4b7e011e9a </ci>
</apply>
</apply>
<apply>
<plus/>
<cn type="integer"> 1 </cn>
<apply>
<divide/>
<ci> mwb2da70f3_0970_4374_bdf2_4a3914afa142 </ci>
<ci> mw6aa54137_d905_4ffa_a29c_b192c4901c8f </ci>
</apply>
<apply>
<divide/>
<ci> mw98624f9d_a464_456c_9a02_026d96612aff </ci>
<ci> mwc941b16b_73fe_48ea_828c_7d4127c52bdc </ci>
</apply>
</apply>
</apply>
</apply>
</math>
</kineticLaw>

```
</reaction>
<reaction id="mwe305d8c3_4e77_4614_ae39_cc32f90ecf24" name="Sink GAP"
reversible="false" fast="false">
<annotation>
<COPASI xmlns="http://www.copasi.org/static/sbml">
<rdf:RDF xmlns:dcterms="http://purl.org/dc/terms/"
xmlns:rdf="http://www.w3.org/1999/02/22-rdf-syntax-ns#">
<rdf:Description rdf:about="#COPASI67">
<dcterms:created>
<rdf:Description>
<dcterms:W3CDTF>2011-04-26T00:16:45Z</dcterms:W3CDTF>
</rdf:Description>
</dcterms:created>
</rdf:Description>
</rdf:RDF>
</COPASI>
</annotation>
<listOfReactants>
<speciesReference species="mw98624f9d_a464_456c_9a02_026d96612aff"/>
</listOfReactants>
<listOfProducts>
<speciesReference species="mw975386b7_cfea_40d4_849f_bf8f1b96464a"
stoichiometry="3"/>
</listOfProducts>
<kineticLaw>
<math xmlns="http://www.w3.org/1998/Math/MathML">
<apply>
<divide/>
<apply>
<times/>
<ci> mw7815c29b_f589_487d_8abf_8c69866a16bf </ci>
<ci> mw98624f9d_a464_456c_9a02_026d96612aff </ci>
```

</apply>
<apply>
<plus/>
<ci> mw0e500944_5cfc_4224_907b_dd0573a17387 </ci>
<ci> mw98624f9d_a464_456c_9a02_026d96612aff </ci>
</apply>
</apply>
</math>
</kineticLaw>
</reaction>
<reaction id="mw6847ac89_b6a4_447f_966b_ddfae4c6bdc4" name="Sink E4P" reversible="false" fast="false">
<annotation>
<COPASI xmlns="http://www.copasi.org/static/sbml">
<rdf:RDF xmlns:dcterms="http://purl.org/dc/terms/" xmlns:rdf="http://www.w3.org/1999/02/22-rdf-syntax-ns#">
<rdf:Description rdf:about="#COPASI68">
<dcterms:created>
<rdf:Description>
<dcterms:W3CDTF>2011-04-26T00:17:24Z</dcterms:W3CDTF>
</rdf:Description>
</dcterms:created>
</rdf:Description>
</rdf:RDF>
</COPASI>
</annotation>
<listOfReactants>
<speciesReference species="mw6c1ca071_aba1_4113_ace9_3d429d27bc60"/>
</listOfReactants>
<listOfProducts>
<speciesReference species="mw2ac0cdfb_0f6b_484c_b7b5_41cfbdace9b8" stoichiometry="4"/>

```
        </listOfProducts>
        <kineticLaw>
          <math xmlns="http://www.w3.org/1998/Math/MathML">
            <apply>
              <divide/>
              <apply>
                <times/>
                <ci> mw94c1a2ea_8406_444a_beaf_3ae6b855d7b5 </ci>
                <ci> mw6c1ca071_aba1_4113_ace9_3d429d27bc60 </ci>
              </apply>
              <apply>
                <plus/>
                <ci> mwd6c6584f_18c7_4c2e_928b_bc7cebf26f01 </ci>
                <ci> mw6c1ca071_aba1_4113_ace9_3d429d27bc60 </ci>
              </apply>
            </apply>
          </math>
        </kineticLaw>
      </reaction>
      <reaction id="mw2110b7cd_2ef5_489b_a88b_2f04bce8790e" name="Sink Ri5P"
reversible="false" fast="false">
        <annotation>
          <COPASI xmlns="http://www.copasi.org/static/sbml">
            <rdf:RDF xmlns:dcterms="http://purl.org/dc/terms/"
xmlns:rdf="http://www.w3.org/1999/02/22-rdf-syntax-ns#">
              <rdf:Description rdf:about="#COPASI69">
                <dcterms:created>
                  <rdf:Description>
                    <dcterms:W3CDTF>2011-04-26T00:17:46Z</dcterms:W3CDTF>
                  </rdf:Description>
                </dcterms:created>
              </rdf:Description>
```

```
          </rdf:RDF>
        </COPASI>
      </annotation>
      <listOfReactants>
        <speciesReference species="mw7b7d97f6_2db4_4473_80be_5e4d69e05b59"/>
      </listOfReactants>
      <listOfProducts>
        <speciesReference species="mw6c2cc5c8_f456_40b7_929f_e3d9e37f99b2"
stoichiometry="5"/>
      </listOfProducts>
      <kineticLaw>
        <math xmlns="http://www.w3.org/1998/Math/MathML">
          <apply>
            <divide/>
            <apply>
              <times/>
              <ci> mwcb26c99a_ae43_4c60_ae4d_a62a4a7bf1d1 </ci>
              <ci> mw7b7d97f6_2db4_4473_80be_5e4d69e05b59 </ci>
            </apply>
            <apply>
              <plus/>
              <ci> mw73931537_4826_46e8_9efc_5a58ea02c555 </ci>
              <ci> mw7b7d97f6_2db4_4473_80be_5e4d69e05b59 </ci>
            </apply>
          </apply>
        </math>
      </kineticLaw>
    </reaction>
    <reaction id="mwfce5177b_a5cf_474a_a467_799ac786beb0" name="Sink TSA" reversible="false"
fast="false">
      <annotation>
        <COPASI xmlns="http://www.copasi.org/static/sbml">
```

```
<rdf:RDF xmlns:dcterms="http://purl.org/dc/terms/"
xmlns:rdf="http://www.w3.org/1999/02/22-rdf-syntax-ns#">
 <rdf:Description rdf:about="#COPASI83">
  <dcterms:created>
   <rdf:Description>
    <dcterms:W3CDTF>2011-06-15T16:40:30Z</dcterms:W3CDTF>
   </rdf:Description>
  </dcterms:created>
 </rdf:Description>
</rdf:RDF>
</COPASI>
</annotation>
<listOfReactants>
 <speciesReference species="mw142bb566_7067_4fc2_b957_dc196c94324a"/>
</listOfReactants>
<listOfProducts>
 <speciesReference species="mw1b5d434c_13de_4b56_bd7d_627742a6ceae"/>
</listOfProducts>
<kineticLaw>
 <math xmlns="http://www.w3.org/1998/Math/MathML">
  <apply>
   <divide/>
   <apply>
    <times/>
    <ci> mwba16aae1_effd_497c_bbdc_ecb17a65553e </ci>
    <ci> mw142bb566_7067_4fc2_b957_dc196c94324a </ci>
   </apply>
   <apply>
    <plus/>
    <ci> mwfb4dc7ff_b572_42e3_8611_ff50379e3543 </ci>
    <ci> mw142bb566_7067_4fc2_b957_dc196c94324a </ci>
```

</apply>
</apply>
</math>
</kineticLaw>
</reaction>
<reaction id="mw42ef88f4_c1fe_4682_b0a8_e3a2635ef888" name="Sink OXA" reversible="false" fast="false">
<annotation>
<COPASI xmlns="http://www.copasi.org/static/sbml">
<rdf:RDF xmlns:dcterms="http://purl.org/dc/terms/" xmlns:rdf="http://www.w3.org/1999/02/22-rdf-syntax-ns#">
<rdf:Description rdf:about="#COPASI86">
<dcterms:created>
<rdf:Description>
<dcterms:W3CDTF>2011-06-28T01:12:33Z</dcterms:W3CDTF>
</rdf:Description>
</dcterms:created>
</rdf:Description>
</rdf:RDF>
</COPASI>
</annotation>
<listOfReactants>
<speciesReference species="mwa86b050a_b141_4f76_9597_674909286fc9"/>
</listOfReactants>
<listOfProducts>
<speciesReference species="mw6a9b239b_a9cc_43c8_b083_35f106e0485d"/>
</listOfProducts>
<kineticLaw>
<math xmlns="http://www.w3.org/1998/Math/MathML">
<apply>
<divide/>
<apply>

<times/>

<ci> mwa9080cfb_3bd3_4be1_9e3d_57f259d6e494 </ci>

<ci> mwa86b050a_b141_4f76_9597_674909286fc9 </ci>

</apply>

<apply>

<plus/>

<ci> mw2c0ecf40_827b_4e66_af45_4bbcef400f33 </ci>

<ci> mwa86b050a_b141_4f76_9597_674909286fc9 </ci>

</apply>

</apply>

</math>

</kineticLaw>

</reaction>

<reaction id="mwb225f6b3_58d7_42a6_8fba_fc94728cc21b" name="Sink PEP" reversible="false" fast="false">

<annotation>

<COPASI xmlns="http://www.copasi.org/static/sbml">

<rdf:RDF xmlns:dcterms="http://purl.org/dc/terms/" xmlns:rdf="http://www.w3.org/1999/02/22-rdf-syntax-ns#">

<rdf:Description rdf:about="#COPASI79">

<dcterms:created>

<rdf:Description>

<dcterms:W3CDTF>2011-05-10T16:28:58Z</dcterms:W3CDTF>

</rdf:Description>

</dcterms:created>

</rdf:Description>

</rdf:RDF>

</COPASI>

</annotation>

<listOfReactants>

<speciesReference species="mwc3b39320_8ede_4d49_ba08_c569fbe1474d"/>

</listOfReactants>

```
        <listOfProducts>
          <speciesReference species="mwdc722523_d3fc_41a7_99f4_eea898e1ed4f"
stoichiometry="3"/>
        </listOfProducts>
        <kineticLaw>
          <math xmlns="http://www.w3.org/1998/Math/MathML">
            <apply>
              <divide/>
              <apply>
                <times/>
                <ci> mw10b1b1bc_dce8_475d_b4fa_c06a66547e4e </ci>
                <ci> mwc3b39320_8ede_4d49_ba08_c569fbe1474d </ci>
              </apply>
              <apply>
                <plus/>
                <ci> mw75e9f4a7_4ba0_493e_b8ce_844e0daf2309 </ci>
                <ci> mwc3b39320_8ede_4d49_ba08_c569fbe1474d </ci>
              </apply>
            </apply>
          </math>
        </kineticLaw>
      </reaction>
      <reaction id="mwff9061f2_1cb4_428f_a269_a48c273b5431" name="Sink Pyruvate"
reversible="false" fast="false">
        <listOfReactants>
          <speciesReference species="mwd81831e8_a743_4783_ae7e_bc4e8ecef290"/>
        </listOfReactants>
        <listOfProducts>
          <speciesReference species="mwdc722523_d3fc_41a7_99f4_eea898e1ed4f"
stoichiometry="3"/>
        </listOfProducts>
        <kineticLaw>
```

```xml
          <math xmlns="http://www.w3.org/1998/Math/MathML">
            <apply>
              <divide/>
              <apply>
                <times/>
                <ci> mw13783949_2260_41c5_a254_46b337a4ec55 </ci>
                <ci> mwd81831e8_a743_4783_ae7e_bc4e8ecef290 </ci>
              </apply>
              <apply>
                <plus/>
                <ci> mw3bfe76ae_7a7c_4327_8477_c7664ca39e64 </ci>
                <ci> mwd81831e8_a743_4783_ae7e_bc4e8ecef290 </ci>
              </apply>
            </apply>
          </math>
        </kineticLaw>
      </reaction>
      <reaction id="mw7547ce91_25b4_4239_bdca_f6a05830850e" name="Sink GLY" reversible="false" fast="false">
        <listOfReactants>
          <speciesReference species="mw7ee6ca0b_cc39_45ef_a044_36cfeb983c0b"/>
        </listOfReactants>
        <listOfProducts>
          <speciesReference species="mw3bf50b2f_519c_495b_b72a_b0753138f38d"/>
        </listOfProducts>
        <kineticLaw>
          <math xmlns="http://www.w3.org/1998/Math/MathML">
            <apply>
              <divide/>
              <apply>
                <times/>
```

```
            <ci> mwa7315a55_d6f0_400f_8bd5_a60441cade2d </ci>
            <ci> mw7ee6ca0b_cc39_45ef_a044_36cfeb983c0b </ci>
          </apply>
          <apply>
            <plus/>
            <ci> mw6f910a3d_c12b_4c9c_8602_d4487e9568ac </ci>
            <ci> mw7ee6ca0b_cc39_45ef_a044_36cfeb983c0b </ci>
          </apply>
        </apply>
      </math>
    </kineticLaw>
  </reaction>
  <reaction id="mw35aef0fc_9cb2_410b_9975_841b8ccef03d" name="Sink SER" reversible="false"
fast="false">
    <listOfReactants>
      <speciesReference species="mw4bff44c2_7520_41bc_80fb_bd781f8b6503"/>
    </listOfReactants>
    <listOfProducts>
      <speciesReference species="mw3bf50b2f_519c_495b_b72a_b0753138f38d"/>
    </listOfProducts>
    <kineticLaw>
      <math xmlns="http://www.w3.org/1998/Math/MathML">
        <apply>
          <divide/>
          <apply>
            <times/>
            <ci> mw02fc51b4_e03b_401c_bcf1_da0d8fd704ab </ci>
            <ci> mw4bff44c2_7520_41bc_80fb_bd781f8b6503 </ci>
          </apply>
          <apply>
            <plus/>
```

```
            <ci> mw5395f222_63ed_4a50_b204_433a6b1f6cbd </ci>
            <ci> mw4bff44c2_7520_41bc_80fb_bd781f8b6503 </ci>
          </apply>
        </apply>
      </math>
    </kineticLaw>
  </reaction>
  <reaction id="mwe92a22b9_76ed_414e_8b95_760351461939" name="Sink G6P"
reversible="false" fast="false">
    <listOfReactants>
      <speciesReference species="mwc5d2bfb5_b299_4e35_b8b7_6f7ff0ce2a5a"/>
    </listOfReactants>
    <listOfProducts>
      <speciesReference species="mw179364ce_63fb_4c6a_a11b_75a2ce49ae4d"
stoichiometry="6"/>
    </listOfProducts>
    <kineticLaw>
      <math xmlns="http://www.w3.org/1998/Math/MathML">
        <apply>
          <divide/>
          <apply>
            <times/>
            <ci> mw2f7b3fed_abd8_460f_aa2b_6034d9dc3ffc </ci>
            <ci> mwc5d2bfb5_b299_4e35_b8b7_6f7ff0ce2a5a </ci>
          </apply>
          <apply>
            <plus/>
            <ci> mw88fca50d_8086_41ed_9f8b_bfce18be9463 </ci>
            <ci> mwc5d2bfb5_b299_4e35_b8b7_6f7ff0ce2a5a </ci>
          </apply>
        </apply>
      </math>
```

</kineticLaw>
</reaction>
<reaction id="mw7c3b3862_dcf8_49ae_85f1_42be7f0ac598" name="Sink AceP" reversible="false" fast="false">
<listOfReactants>
<speciesReference species="mwe1915c23_bc54_4444_a6b6_afb9f835d8cf"/>
</listOfReactants>
<listOfProducts>
<speciesReference species="mwa94479e8_4e60_4fc5_9681_8c8e54d33e74" stoichiometry="2"/>
</listOfProducts>
<kineticLaw>
<math xmlns="http://www.w3.org/1998/Math/MathML">
<apply>
<divide/>
<apply>
<times/>
<ci> mw4ffd888c_f8cc_4980_b408_b8c7d289c5aa </ci>
<ci> mwe1915c23_bc54_4444_a6b6_afb9f835d8cf </ci>
</apply>
<apply>
<plus/>
<ci> mw8638e2a1_77bb_4b82_9b9b_0be22fe4d7e4 </ci>
<ci> mwe1915c23_bc54_4444_a6b6_afb9f835d8cf </ci>
</apply>
</apply>
</math>
</kineticLaw>
</reaction>
<reaction id="mwcb52801e_5eab_40d7_ac32_21920a2122f4" name="Mixotrophic growth - G6P as a source" reversible="false" fast="false">
<listOfReactants>

```
      &lt;speciesReference species="mw5c9d3afc_2037_42d9_a8c6_e341b406cf37"/&gt;
     &lt;/listOfReactants&gt;
     &lt;listOfProducts&gt;
      &lt;speciesReference species="mwc5d2bfb5_b299_4e35_b8b7_6f7ff0ce2a5a"/&gt;
     &lt;/listOfProducts&gt;
     &lt;kineticLaw&gt;
      &lt;math xmlns="http://www.w3.org/1998/Math/MathML"&gt;
       &lt;apply&gt;
        &lt;times/&gt;
        &lt;ci&gt; mwc6aa212e_7e00_4697_af9f_140f4ef235ea &lt;/ci&gt;
        &lt;ci&gt; mw5c9d3afc_2037_42d9_a8c6_e341b406cf37 &lt;/ci&gt;
       &lt;/apply&gt;
      &lt;/math&gt;
     &lt;/kineticLaw&gt;
    &lt;/reaction&gt;
    <reaction id="mw23c0648f_bdc2_49ac_a9af_a3e6b12480ae" name="ED-P edd"
reversible="false" fast="false">
     &lt;listOfReactants&gt;
      &lt;speciesReference species="mw630d7a9f_d907_496c_b750_6c23afbb4658"/&gt;
     &lt;/listOfReactants&gt;
     &lt;listOfProducts&gt;
      &lt;speciesReference species="mw01be9f1c_a2a5_492c_bcd3_f7f50fab164f"/&gt;
     &lt;/listOfProducts&gt;
     &lt;kineticLaw&gt;
      &lt;math xmlns="http://www.w3.org/1998/Math/MathML"&gt;
       &lt;apply&gt;
        &lt;divide/&gt;
        &lt;apply&gt;
         &lt;divide/&gt;
         &lt;apply&gt;
          &lt;times/&gt;
```

```
          <ci> mwc689e60c_5ebe_4df0_9311_a4fa4858f41d </ci>
          <ci> mw630d7a9f_d907_496c_b750_6c23afbb4658 </ci>
        </apply>
        <apply>
          <plus/>
          <ci> mw630d7a9f_d907_496c_b750_6c23afbb4658 </ci>
          <ci> mw68578e28_5e7b_43b7_a251_4585ec996637 </ci>
        </apply>
      </apply>
      <apply>
        <plus/>
        <cn type="integer"> 1 </cn>
        <apply>
          <divide/>
          <ci> mw01be9f1c_a2a5_492c_bcd3_f7f50fab164f </ci>
          <ci> mw3cd3e9a1_dbe4_41c7_870c_fb4e65d18298 </ci>
        </apply>
      </apply>
    </apply>
  </math>
</kineticLaw>
</reaction>
<reaction id="mw88918651_12ab_4759_ac46_e60d6750dd0e" name="ED-P eda"
reversible="false" fast="false">
  <listOfReactants>
    <speciesReference species="mw01be9f1c_a2a5_492c_bcd3_f7f50fab164f"/>
  </listOfReactants>
  <listOfProducts>
    <speciesReference species="mw98624f9d_a464_456c_9a02_026d96612aff"/>
    <speciesReference species="mwd81831e8_a743_4783_ae7e_bc4e8ecef290"/>
  </listOfProducts>
```

```
        <kineticLaw>
          <math xmlns="http://www.w3.org/1998/Math/MathML">
            <apply>
              <divide/>
              <apply>
                <times/>
                <ci> mw7d24069f_e15e_473e_99dc_4e20bd4238c9 </ci>
                <ci> mw01be9f1c_a2a5_492c_bcd3_f7f50fab164f </ci>
              </apply>
              <apply>
                <plus/>
                <ci> mwe9068a7b_a4f6_4f0d_b901_8440765efbaf </ci>
                <ci> mw01be9f1c_a2a5_492c_bcd3_f7f50fab164f </ci>
              </apply>
            </apply>
          </math>
        </kineticLaw>
      </reaction>
    </listOfReactions>
  </model>
</sbml>
```
