# Supplementary material for: Entner-Doudoroff pathway in Synechocystis PCC 6803: Proposed regulatory roles and enzyme multifunctionalities
Source: Front Microbiol. 2022 Aug 16;13:967545. doi: 10.3389/fmicb.2022.967545 (PMC9424857; doi:10.3389/fmicb.2022.967545)
Supplement: Supplementary file 6 (Data sheet 4) — Kinetic model of central carbon metabolism of Synechocystis sp. PCC 6803 under mixotrophic conditions. Model description can be found in the section “General information about the model”. [file Data_Sheet_4.pdf]

How to use: copy the code below without this text into .txt file and after saving, change the extension to .xml, then open it in MATLAB SimBiology


```
<?xml version="1.0" encoding="UTF-8"?>
<sbml xmlns="http://www.sbml.org/sbml/level2" xmlns:html="http://www.w3.org/1999/xhtml"
level="2" version="1">
  <annotation>
Created by The MathWorks, Inc. SimBiology tool, Version 3.3
</annotation>
  <model id="mw7d0fff43_d9a4_4c82_95ee_f3d36ee8cba0" name="initial">
    <notes>
      <body xmlns="http://www.w3.org/1999/xhtml"></body>
    </notes>
    <annotation>
      <COPASI xmlns="http://www.copasi.org/static/sbml">
        <rdf:RDF xmlns:dcterms="http://purl.org/dc/terms/"
xmlns:rdf="http://www.w3.org/1999/02/22-rdf-syntax-ns#">
          <rdf:Description rdf:about="#COPASI1">
            <dcterms:created>
              <rdf:Description>
                <dcterms:W3CDTF>2010-04-14T14:47:02Z</dcterms:W3CDTF>
              </rdf:Description>
            </dcterms:created>
          </rdf:Description>
        </rdf:RDF>
      </COPASI>
    </annotation>
    <listOfCompartments>
      <compartment id="mw9adfae27_c966_47b7_a7b5_9970861ef456" name="Stroma" size="1">
        <annotation>
          <COPASI xmlns="http://www.copasi.org/static/sbml">
```

```
          <rdf:RDF xmlns:dcterms="http://purl.org/dc/terms/"
xmlns:rdf="http://www.w3.org/1999/02/22-rdf-syntax-ns#">
            <rdf:Description rdf:about="#COPASI2">
              <dcterms:created>
                <rdf:Description>
                  <dcterms:W3CDTF>2010-04-14T14:56:43Z</dcterms:W3CDTF>
                </rdf:Description>
              </dcterms:created>
            </rdf:Description>
          </rdf:RDF>
        </COPASI>
      </annotation>
    </compartment>
    <compartment id="mw27e3f884_16a3_4659_ac3d_178dbbae0ed4" name="external" size="1"/>
  </listOfCompartments>
  <listOfSpecies>
    <species id="mwf3d533fa_7176_4543_a1c4_f46f4dc4f8f6" name="CO2"
compartment="mw9adfae27_c966_47b7_a7b5_9970861ef456" initialAmount="0.02">
      <annotation>
        <COPASI xmlns="http://www.copasi.org/static/sbml">
          <rdf:RDF xmlns:dcterms="http://purl.org/dc/terms/"
xmlns:rdf="http://www.w3.org/1999/02/22-rdf-syntax-ns#">
            <rdf:Description rdf:about="#COPASI3">
              <dcterms:created>
                <rdf:Description>
                  <dcterms:W3CDTF>2010-04-14T15:46:19Z</dcterms:W3CDTF>
                </rdf:Description>
              </dcterms:created>
            </rdf:Description>
          </rdf:RDF>
        </COPASI>
      </annotation>
```

```
      </species>
      <species id="mw372b8247_b11e_4d9b_be9b_e742fd2d3853" name="PGA" compartment="mw9adfae27_c966_47b7_a7b5_9970861ef456" initialAmount="4.7">
        <annotation>
          <COPASI xmlns="http://www.copasi.org/static/sbml">
            <rdf:RDF xmlns:dcterms="http://purl.org/dc/terms/" xmlns:rdf="http://www.w3.org/1999/02/22-rdf-syntax-ns#">
              <rdf:Description rdf:about="#COPASI4">
                <dcterms:created>
                  <rdf:Description>
                    <dcterms:W3CDTF>2010-04-14T15:47:21Z</dcterms:W3CDTF>
                  </rdf:Description>
                </dcterms:created>
              </rdf:Description>
            </rdf:RDF>
          </COPASI>
        </annotation>
      </species>
      <species id="mw98fecd51_ba08_4727_98ec_04637a47c5ac" name="RuBP" compartment="mw9adfae27_c966_47b7_a7b5_9970861ef456" initialAmount="1">
        <annotation>
          <COPASI xmlns="http://www.copasi.org/static/sbml">
            <rdf:RDF xmlns:dcterms="http://purl.org/dc/terms/" xmlns:rdf="http://www.w3.org/1999/02/22-rdf-syntax-ns#">
              <rdf:Description rdf:about="#COPASI5">
                <dcterms:created>
                  <rdf:Description>
                    <dcterms:W3CDTF>2010-04-14T15:47:38Z</dcterms:W3CDTF>
                  </rdf:Description>
                </dcterms:created>
              </rdf:Description>
            </rdf:RDF>
```

```
</COPASI>
</annotation>
</species>
<species id="mwd2e6e004_23f5_4cd8_81e0_8385021ec904" name="NADPH"
compartment="mw9adfae27_c966_47b7_a7b5_9970861ef456"
initialAmount="0.148995513447536">
<annotation>
<COPASI xmlns="http://www.copasi.org/static/sbml">
<rdf:RDF xmlns:dcterms="http://purl.org/dc/terms/"
xmlns:rdf="http://www.w3.org/1999/02/22-rdf-syntax-ns#">
<rdf:Description rdf:about="#COPASI6">
<dcterms:created>
<rdf:Description>
<dcterms:W3CDTF>2010-04-14T15:47:02Z</dcterms:W3CDTF>
</rdf:Description>
</dcterms:created>
</rdf:Description>
</rdf:RDF>
</COPASI>
</annotation>
</species>
<species id="mw739c8f22_6a61_4dca_8cfe_3c962adb4128" name="O2"
compartment="mw9adfae27_c966_47b7_a7b5_9970861ef456"
initialAmount="0.0259999987479538" boundaryCondition="true" constant="true">
<annotation>
<COPASI xmlns="http://www.copasi.org/static/sbml">
<rdf:RDF xmlns:dcterms="http://purl.org/dc/terms/"
xmlns:rdf="http://www.w3.org/1999/02/22-rdf-syntax-ns#">
<rdf:Description rdf:about="#COPASI7">
<dcterms:created>
<rdf:Description>
<dcterms:W3CDTF>2010-04-14T15:47:13Z</dcterms:W3CDTF>
</rdf:Description>
```

</dcterms:created>

</rdf:Description>

</rdf:RDF>

</COPASI>

</annotation>

</species>

<species id="mwacf08e72_f001_4a26_962d_729f504b1ad0" name="SBP" compartment="mw9adfae27_c966_47b7_a7b5_9970861ef456" initialAmount="1.36261887752912">

<annotation>

<COPASI xmlns="http://www.copasi.org/static/sbml">

<rdf:RDF xmlns:dcterms="http://purl.org/dc/terms/" xmlns:rdf="http://www.w3.org/1999/02/22-rdf-syntax-ns#">

<rdf:Description rdf:about="#COPASI8">

<dcterms:created>

<rdf:Description>

<dcterms:W3CDTF>2010-04-14T15:47:55Z</dcterms:W3CDTF>

</rdf:Description>

</dcterms:created>

</rdf:Description>

</rdf:RDF>

</COPASI>

</annotation>

</species>

<species id="mwe8d1ae1f_35f9_45fd_bedf_7dbeef3972e2" name="ADP" compartment="mw9adfae27_c966_47b7_a7b5_9970861ef456" initialAmount="0.344798607704336">

<annotation>

<COPASI xmlns="http://www.copasi.org/static/sbml">

<rdf:RDF xmlns:dcterms="http://purl.org/dc/terms/" xmlns:rdf="http://www.w3.org/1999/02/22-rdf-syntax-ns#">

<rdf:Description rdf:about="#COPASI9">

<dcterms:created>

<rdf:Description>

<dcterms:W3CDTF>2010-04-14T16:01:25Z</dcterms:W3CDTF>

</rdf:Description>

</dcterms:created>

</rdf:Description>

</rdf:RDF>

</COPASI>

</annotation>

</species>

<species id="mw8e2cc29c_c73e_4258_b3ba_2356620bb64e" name="ATP" compartment="mw9adfae27_c966_47b7_a7b5_9970861ef456" initialAmount="1.15520132006222">

<annotation>

<COPASI xmlns="http://www.copasi.org/static/sbml">

<rdf:RDF xmlns:dcterms="http://purl.org/dc/terms/" xmlns:rdf="http://www.w3.org/1999/02/22-rdf-syntax-ns#">

<rdf:Description rdf:about="#COPASI10">

<dcterms:created>

<rdf:Description>

<dcterms:W3CDTF>2010-04-14T16:01:14Z</dcterms:W3CDTF>

</rdf:Description>

</dcterms:created>

</rdf:Description>

</rdf:RDF>

</COPASI>

</annotation>

</species>

<species id="mwc8b5cb3b_9f99_46df_a6e4_0126749144fc" name="BPGA" compartment="mw9adfae27_c966_47b7_a7b5_9970861ef456" initialAmount="0.00748248557057663">

<annotation>

<COPASI xmlns="http://www.copasi.org/static/sbml">

<rdf:RDF xmlns:dcterms="http://purl.org/dc/terms/" xmlns:rdf="http://www.w3.org/1999/02/22-rdf-syntax-ns#">

<rdf:Description rdf:about="#COPASI11">

<dcterms:created>

<rdf:Description>

<dcterms:W3CDTF>2010-04-14T16:01:08Z</dcterms:W3CDTF>

</rdf:Description>

</dcterms:created>

</rdf:Description>

</rdf:RDF>

</COPASI>

</annotation>

</species>

<species id="mw5b528d37_b514_46e5_a018_0173afdae714" name="GAP" compartment="mw9adfae27_c966_47b7_a7b5_9970861ef456" initialAmount="0.368362636458716">

<annotation>

<COPASI xmlns="http://www.copasi.org/static/sbml">

<rdf:RDF xmlns:dcterms="http://purl.org/dc/terms/" xmlns:rdf="http://www.w3.org/1999/02/22-rdf-syntax-ns#">

<rdf:Description rdf:about="#COPASI12">

<dcterms:created>

<rdf:Description>

<dcterms:W3CDTF>2010-06-08T23:46:46Z</dcterms:W3CDTF>

</rdf:Description>

</dcterms:created>

</rdf:Description>

</rdf:RDF>

</COPASI>

</annotation>

</species>

```
<species id="mw7c834bb1_1865_4950_aa20_f3eceb92b21d" name="NADPp" compartment="mw9adfae27_c966_47b7_a7b5_9970861ef456" initialAmount="0.271004466327097">
  <annotation>
    <COPASI xmlns="http://www.copasi.org/static/sbml">
      <rdf:RDF xmlns:dcterms="http://purl.org/dc/terms/" xmlns:rdf="http://www.w3.org/1999/02/22-rdf-syntax-ns#">
        <rdf:Description rdf:about="#COPASI13">
          <dcterms:created>
            <rdf:Description>
              <dcterms:W3CDTF>2010-06-08T23:43:15Z</dcterms:W3CDTF>
            </rdf:Description>
          </dcterms:created>
        </rdf:Description>
      </rdf:RDF>
    </COPASI>
  </annotation>
</species>
<species id="mw854ce178_2796_4118_8690_c13951a897c1" name="Pi" compartment="mw9adfae27_c966_47b7_a7b5_9970861ef456" initialAmount="4.99999975922188" boundaryCondition="true" constant="true">
  <annotation>
    <COPASI xmlns="http://www.copasi.org/static/sbml">
      <rdf:RDF xmlns:dcterms="http://purl.org/dc/terms/" xmlns:rdf="http://www.w3.org/1999/02/22-rdf-syntax-ns#">
        <rdf:Description rdf:about="#COPASI14">
          <dcterms:created>
            <rdf:Description>
              <dcterms:W3CDTF>2010-05-18T18:10:26Z</dcterms:W3CDTF>
            </rdf:Description>
          </dcterms:created>
        </rdf:Description>
      </rdf:RDF>
```

</COPASI>
</annotation>
</species>
<species id="mw8f67b710_8c1c_453f_a7ec_e9b6a55814fa" name="DHAP" compartment="mw9adfae27_c966_47b7_a7b5_9970861ef456" initialAmount="0.012">
<annotation>
<COPASI xmlns="http://www.copasi.org/static/sbml">
<rdf:RDF xmlns:dcterms="http://purl.org/dc/terms/" xmlns:rdf="http://www.w3.org/1999/02/22-rdf-syntax-ns#">
<rdf:Description rdf:about="#COPASI15">
<dcterms:created>
<rdf:Description>
<dcterms:W3CDTF>2010-06-08T23:43:34Z</dcterms:W3CDTF>
</rdf:Description>
</dcterms:created>
</rdf:Description>
</rdf:RDF>
</COPASI>
</annotation>
</species>
<species id="mw2a09f89f_5936_4376_a2ab_4ff1bbbd4153" name="FBP" compartment="mw9adfae27_c966_47b7_a7b5_9970861ef456" initialAmount="0.02">
<annotation>
<COPASI xmlns="http://www.copasi.org/static/sbml">
<rdf:RDF xmlns:dcterms="http://purl.org/dc/terms/" xmlns:rdf="http://www.w3.org/1999/02/22-rdf-syntax-ns#">
<rdf:Description rdf:about="#COPASI16">
<dcterms:created>
<rdf:Description>
<dcterms:W3CDTF>2010-06-08T23:44:30Z</dcterms:W3CDTF>
</rdf:Description>
</dcterms:created>

</rdf:Description>

</rdf:RDF>

</COPASI>

</annotation>

</species>

<species id="mwfcedd013_9e1c_4e21_9a5a_93ddde2ffc11" name="F6P" compartment="mw9adfae27_c966_47b7_a7b5_9970861ef456" initialAmount="2">

<annotation>

<COPASI xmlns="http://www.copasi.org/static/sbml">

<rdf:RDF xmlns:dcterms="http://purl.org/dc/terms/" xmlns:rdf="http://www.w3.org/1999/02/22-rdf-syntax-ns#">

<rdf:Description rdf:about="#COPASI17">

<dcterms:created>

<rdf:Description>

<dcterms:W3CDTF>2010-06-08T23:44:21Z</dcterms:W3CDTF>

</rdf:Description>

</dcterms:created>

</rdf:Description>

</rdf:RDF>

</COPASI>

</annotation>

</species>

<species id="mwaa8b93c8_308a_4d19_b732_22cb31f77620" name="E4P" compartment="mw9adfae27_c966_47b7_a7b5_9970861ef456" initialAmount="0.228820226685508">

<annotation>

<COPASI xmlns="http://www.copasi.org/static/sbml">

<rdf:RDF xmlns:dcterms="http://purl.org/dc/terms/" xmlns:rdf="http://www.w3.org/1999/02/22-rdf-syntax-ns#">

<rdf:Description rdf:about="#COPASI18">

<dcterms:created>

<rdf:Description>

<dcterms:W3CDTF>2010-06-08T23:43:55Z</dcterms:W3CDTF>

</rdf:Description>

</dcterms:created>

</rdf:Description>

</rdf:RDF>

</COPASI>

</annotation>

</species>

<species id="mw7c70a014_6b69_42b3_9fbd_1d63cb722ae5" name="Xu5P" compartment="mw9adfae27_c966_47b7_a7b5_9970861ef456" initialAmount="0.0558077870361159">

<annotation>

<COPASI xmlns="http://www.copasi.org/static/sbml">

<rdf:RDF xmlns:dcterms="http://purl.org/dc/terms/" xmlns:rdf="http://www.w3.org/1999/02/22-rdf-syntax-ns#">

<rdf:Description rdf:about="#COPASI19">

<dcterms:created>

<rdf:Description>

<dcterms:W3CDTF>2010-06-08T23:41:49Z</dcterms:W3CDTF>

</rdf:Description>

</dcterms:created>

</rdf:Description>

</rdf:RDF>

</COPASI>

</annotation>

</species>

<species id="mw26816e75_d092_459e_bb3d_70aafa096e4d" name="Ri5P" compartment="mw9adfae27_c966_47b7_a7b5_9970861ef456" initialAmount="0.0744045363011372">

<annotation>

<COPASI xmlns="http://www.copasi.org/static/sbml">

<rdf:RDF xmlns:dcterms="http://purl.org/dc/terms/" xmlns:rdf="http://www.w3.org/1999/02/22-rdf-syntax-ns#">

<rdf:Description rdf:about="#COPASI20">

<dcterms:created>

<rdf:Description>

<dcterms:W3CDTF>2010-06-08T23:41:08Z</dcterms:W3CDTF>

</rdf:Description>

</dcterms:created>

</rdf:Description>

</rdf:RDF>

</COPASI>

</annotation>

</species>

<species id="mweadaac0b_cbd7_4eb3_b892_fe6bf884cfc8" name="S7P" compartment="mw9adfae27_c966_47b7_a7b5_9970861ef456" initialAmount="0.511319423085088">

<annotation>

<COPASI xmlns="http://www.copasi.org/static/sbml">

<rdf:RDF xmlns:dcterms="http://purl.org/dc/terms/" xmlns:rdf="http://www.w3.org/1999/02/22-rdf-syntax-ns#">

<rdf:Description rdf:about="#COPASI21">

<dcterms:created>

<rdf:Description>

<dcterms:W3CDTF>2010-06-08T23:47:56Z</dcterms:W3CDTF>

</rdf:Description>

</dcterms:created>

</rdf:Description>

</rdf:RDF>

</COPASI>

</annotation>

</species>

<species id="mw1d7eb0d8_a048_4013_9bbd_c347637cc6b9" name="Ru5P" compartment="mw9adfae27_c966_47b7_a7b5_9970861ef456" initialAmount="0.026898222838205">

<annotation>

<COPASI xmlns="http://www.copasi.org/static/sbml">

<rdf:RDF xmlns:dcterms="http://purl.org/dc/terms/" xmlns:rdf="http://www.w3.org/1999/02/22-rdf-syntax-ns#">

<rdf:Description rdf:about="#COPASI22">

<dcterms:created>

<rdf:Description>

<dcterms:W3CDTF>2010-06-08T23:41:17Z</dcterms:W3CDTF>

</rdf:Description>

</dcterms:created>

</rdf:Description>

</rdf:RDF>

</COPASI>

</annotation>

</species>

<species id="mw3a6830cd_1b04_49df_b4fe_fc7a6362142a" name="G6P" compartment="mw9adfae27_c966_47b7_a7b5_9970861ef456" initialAmount="1.94057630985951">

<annotation>

<COPASI xmlns="http://www.copasi.org/static/sbml">

<rdf:RDF xmlns:dcterms="http://purl.org/dc/terms/" xmlns:rdf="http://www.w3.org/1999/02/22-rdf-syntax-ns#">

<rdf:Description rdf:about="#COPASI23">

<dcterms:created>

<rdf:Description>

<dcterms:W3CDTF>2010-06-08T23:46:39Z</dcterms:W3CDTF>

</rdf:Description>

</dcterms:created>

</rdf:Description>

</rdf:RDF>

</COPASI>

</annotation>

</species>

<species id="mw11f497f7_cda2_4e49_a7e3_f2afaa053b5d" name="Sink1" compartment="mw9adfae27_c966_47b7_a7b5_9970861ef456" initialAmount="0">

<annotation>
<COPASI xmlns="http://www.copasi.org/static/sbml">
<rdf:RDF xmlns:dcterms="http://purl.org/dc/terms/" xmlns:rdf="http://www.w3.org/1999/02/22-rdf-syntax-ns#">
<rdf:Description rdf:about="#COPASI25">
<dcterms:created>
<rdf:Description>
<dcterms:W3CDTF>2011-05-10T00:14:21Z</dcterms:W3CDTF>
</rdf:Description>
</dcterms:created>
</rdf:Description>
</rdf:RDF>
</COPASI>
</annotation>
</species>
<species id="mw99ae2c3c_4850_462a_8eb9_29ce642d9a9c" name="Sink2" compartment="mw9adfae27_c966_47b7_a7b5_9970861ef456" initialAmount="0">
<annotation>
<COPASI xmlns="http://www.copasi.org/static/sbml">
<rdf:RDF xmlns:dcterms="http://purl.org/dc/terms/" xmlns:rdf="http://www.w3.org/1999/02/22-rdf-syntax-ns#">
<rdf:Description rdf:about="#COPASI26">
<dcterms:created>
<rdf:Description>
<dcterms:W3CDTF>2011-04-26T00:48:45Z</dcterms:W3CDTF>
</rdf:Description>
</dcterms:created>
</rdf:Description>
</rdf:RDF>
</COPASI>
</annotation>
</species>

<species id="mw04176f80_f815_4c8f_a0d2_7625e7c0868b" name="PGCA" compartment="mw9adfae27_c966_47b7_a7b5_9970861ef456" initialAmount="0.01">

<annotation>

<COPASI xmlns="http://www.copasi.org/static/sbml">

<rdf:RDF xmlns:dcterms="http://purl.org/dc/terms/" xmlns:rdf="http://www.w3.org/1999/02/22-rdf-syntax-ns#">

<rdf:Description rdf:about="#COPASI27">

<dcterms:created>

<rdf:Description>

<dcterms:W3CDTF>2011-06-15T16:28:14Z</dcterms:W3CDTF>

</rdf:Description>

</dcterms:created>

</rdf:Description>

</rdf:RDF>

</COPASI>

</annotation>

</species>

<species id="mwd946c4b9_b1d9_4b6d_9616_2f03e37dc588" name="GCA" compartment="mw9adfae27_c966_47b7_a7b5_9970861ef456" initialAmount="0.0123593835433703">

<annotation>

<COPASI xmlns="http://www.copasi.org/static/sbml">

<rdf:RDF xmlns:dcterms="http://purl.org/dc/terms/" xmlns:rdf="http://www.w3.org/1999/02/22-rdf-syntax-ns#">

<rdf:Description rdf:about="#COPASI28">

<dcterms:created>

<rdf:Description>

<dcterms:W3CDTF>2011-06-15T16:28:15Z</dcterms:W3CDTF>

</rdf:Description>

</dcterms:created>

</rdf:Description>

</rdf:RDF>

</COPASI>

</annotation>

</species>

<species id="mwb970288f_4109_4c97_90b7_cc60512dc613" name="GOA" compartment="mw9adfae27_c966_47b7_a7b5_9970861ef456" initialAmount="0.0327363830028097">

<annotation>

<COPASI xmlns="http://www.copasi.org/static/sbml">

<rdf:RDF xmlns:dcterms="http://purl.org/dc/terms/" xmlns:rdf="http://www.w3.org/1999/02/22-rdf-syntax-ns#">

<rdf:Description rdf:about="#COPASI29">

<dcterms:created>

<rdf:Description>

<dcterms:W3CDTF>2011-06-15T16:28:23Z</dcterms:W3CDTF>

</rdf:Description>

</dcterms:created>

</rdf:Description>

</rdf:RDF>

</COPASI>

</annotation>

</species>

<species id="mw3d986236_5c95_4128_9ee4_95d616f64d6f" name="GLY" compartment="mw9adfae27_c966_47b7_a7b5_9970861ef456" initialAmount="0.8">

<annotation>

<COPASI xmlns="http://www.copasi.org/static/sbml">

<rdf:RDF xmlns:dcterms="http://purl.org/dc/terms/" xmlns:rdf="http://www.w3.org/1999/02/22-rdf-syntax-ns#">

<rdf:Description rdf:about="#COPASI30">

<dcterms:created>

<rdf:Description>

<dcterms:W3CDTF>2011-06-15T16:27:59Z</dcterms:W3CDTF>

</rdf:Description>

</dcterms:created>

</rdf:Description>

</rdf:RDF>

</COPASI>

</annotation>

</species>

<species id="mw7bb27e61_6440_4c3b_af0a_6c1ecd36ff29" name="SER" compartment="mw9adfae27_c966_47b7_a7b5_9970861ef456" initialAmount="0.5">

<annotation>

<COPASI xmlns="http://www.copasi.org/static/sbml">

<rdf:RDF xmlns:dcterms="http://purl.org/dc/terms/" xmlns:rdf="http://www.w3.org/1999/02/22-rdf-syntax-ns#">

<rdf:Description rdf:about="#COPASI31">

<dcterms:created>

<rdf:Description>

<dcterms:W3CDTF>2011-06-15T16:28:35Z</dcterms:W3CDTF>

</rdf:Description>

</dcterms:created>

</rdf:Description>

</rdf:RDF>

</COPASI>

</annotation>

</species>

<species id="mw21f7bed2_a8e0_4d16_a4a4_8718de3f1c6a" name="HPR" compartment="mw9adfae27_c966_47b7_a7b5_9970861ef456" initialAmount="0.1">

<annotation>

<COPASI xmlns="http://www.copasi.org/static/sbml">

<rdf:RDF xmlns:dcterms="http://purl.org/dc/terms/" xmlns:rdf="http://www.w3.org/1999/02/22-rdf-syntax-ns#">

<rdf:Description rdf:about="#COPASI32">

<dcterms:created>

<rdf:Description>

<dcterms:W3CDTF>2011-06-15T16:38:48Z</dcterms:W3CDTF>

</rdf:Description>

```
              </dcterms:created>
            </rdf:Description>
          </rdf:RDF>
        </COPASI>
      </annotation>
    </species>
    <species id="mw3a8a1813_5005_474a_8fd1_d573b1ec121f" name="GCEA"
compartment="mw9adfae27_c966_47b7_a7b5_9970861ef456"
initialAmount="0.00400097153227282">
      <annotation>
        <COPASI xmlns="http://www.copasi.org/static/sbml">
          <rdf:RDF xmlns:dcterms="http://purl.org/dc/terms/"
xmlns:rdf="http://www.w3.org/1999/02/22-rdf-syntax-ns#">
            <rdf:Description rdf:about="#COPASI33">
              <dcterms:created>
                <rdf:Description>
                  <dcterms:W3CDTF>2011-06-15T16:30:28Z</dcterms:W3CDTF>
                </rdf:Description>
              </dcterms:created>
            </rdf:Description>
          </rdf:RDF>
        </COPASI>
      </annotation>
    </species>
    <species id="mw6951dd88_19cf_4045_95c4_0e8e2bf976c1" name="PEP"
compartment="mw9adfae27_c966_47b7_a7b5_9970861ef456"
initialAmount="4.19091918967107">
      <annotation>
        <COPASI xmlns="http://www.copasi.org/static/sbml">
          <rdf:RDF xmlns:dcterms="http://purl.org/dc/terms/"
xmlns:rdf="http://www.w3.org/1999/02/22-rdf-syntax-ns#">
            <rdf:Description rdf:about="#COPASI34">
              <dcterms:created>
```

<rdf:Description>

<dcterms:W3CDTF>2011-05-10T16:27:35Z</dcterms:W3CDTF>

</rdf:Description>

</dcterms:created>

</rdf:Description>

</rdf:RDF>

</COPASI>

</annotation>

</species>

<species id="mw5ef07c48_166e_4959_9987_d5fc6d58add4" name="Sink3" compartment="mw9adfae27_c966_47b7_a7b5_9970861ef456" initialAmount="0">

<annotation>

<COPASI xmlns="http://www.copasi.org/static/sbml">

<rdf:RDF xmlns:dcterms="http://purl.org/dc/terms/" xmlns:rdf="http://www.w3.org/1999/02/22-rdf-syntax-ns#">

<rdf:Description rdf:about="#COPASI35">

<dcterms:created>

<rdf:Description>

<dcterms:W3CDTF>2011-05-10T16:30:50Z</dcterms:W3CDTF>

</rdf:Description>

</dcterms:created>

</rdf:Description>

</rdf:RDF>

</COPASI>

</annotation>

</species>

<species id="mw6b76fe71_4295_4311_999a_67179a3d5a74" name="Sink4" compartment="mw9adfae27_c966_47b7_a7b5_9970861ef456" initialAmount="0">

<annotation>

<COPASI xmlns="http://www.copasi.org/static/sbml">

<rdf:RDF xmlns:dcterms="http://purl.org/dc/terms/" xmlns:rdf="http://www.w3.org/1999/02/22-rdf-syntax-ns#">

<rdf:Description rdf:about="#COPASI36">

<dcterms:created>

<rdf:Description>

<dcterms:W3CDTF>2011-06-15T16:28:43Z</dcterms:W3CDTF>

</rdf:Description>

</dcterms:created>

</rdf:Description>

</rdf:RDF>

</COPASI>

</annotation>

</species>

<species id="mwc25bd841_1335_4e94_9ffa_621b205bc0da" name="ppGA" compartment="mw9adfae27_c966_47b7_a7b5_9970861ef456" initialAmount="4.19091917967427">

<annotation>

<COPASI xmlns="http://www.copasi.org/static/sbml">

<rdf:RDF xmlns:dcterms="http://purl.org/dc/terms/" xmlns:rdf="http://www.w3.org/1999/02/22-rdf-syntax-ns#">

<rdf:Description rdf:about="#COPASI37">

<dcterms:created>

<rdf:Description>

<dcterms:W3CDTF>2011-06-15T16:30:09Z</dcterms:W3CDTF>

</rdf:Description>

</dcterms:created>

</rdf:Description>

</rdf:RDF>

</COPASI>

</annotation>

</species>

<species id="mw7b17f46e_6246_48f0_9aeb_fb89cc666077" name="TSA" compartment="mw9adfae27_c966_47b7_a7b5_9970861ef456" initialAmount="0.0324331443243821">

<annotation>

```
          <COPASI xmlns="http://www.copasi.org/static/sbml">
            <rdf:RDF xmlns:dcterms="http://purl.org/dc/terms/"
xmlns:rdf="http://www.w3.org/1999/02/22-rdf-syntax-ns#">
              <rdf:Description rdf:about="#COPASI38">
                <dcterms:created>
                  <rdf:Description>
                    <dcterms:W3CDTF>2011-06-15T16:42:10Z</dcterms:W3CDTF>
                  </rdf:Description>
                </dcterms:created>
              </rdf:Description>
            </rdf:RDF>
          </COPASI>
        </annotation>
      </species>
      <species id="mw3885b0e0_19bc_4407_a41c_94c1f48987d7" name="OXA"
compartment="mw9adfae27_c966_47b7_a7b5_9970861ef456" initialAmount="0.1">
        <annotation>
          <COPASI xmlns="http://www.copasi.org/static/sbml">
            <rdf:RDF xmlns:dcterms="http://purl.org/dc/terms/"
xmlns:rdf="http://www.w3.org/1999/02/22-rdf-syntax-ns#">
              <rdf:Description rdf:about="#COPASI39">
                <dcterms:created>
                  <rdf:Description>
                    <dcterms:W3CDTF>2011-06-28T01:15:08Z</dcterms:W3CDTF>
                  </rdf:Description>
                </dcterms:created>
              </rdf:Description>
            </rdf:RDF>
          </COPASI>
        </annotation>
      </species>
```

<species id="mwc4d744f3_4e27_4a3b_8596_20885c0a4f55" name="ATPr" compartment="mw9adfae27_c966_47b7_a7b5_9970861ef456" initialAmount="0"/>

<species id="mw29f84bc6_4f93_4a0d_b5ba_3692acbc98b4" name="Mass" compartment="mw9adfae27_c966_47b7_a7b5_9970861ef456" initialAmount="0"/>

<species id="mwd9458308_bcb6_4caf_b7ac_c957a90c648b" name="Sink_DHAP" compartment="mw9adfae27_c966_47b7_a7b5_9970861ef456" initialAmount="0"/>

<species id="mwd6fab0c0_73f8_45ef_b32f_c7113be8258a" name="Sink_E4P" compartment="mw9adfae27_c966_47b7_a7b5_9970861ef456" initialAmount="0"/>

<species id="mw24e36765_3502_4855_9948_95a6bb63a7a1" name="Sink_Ri5P" compartment="mw9adfae27_c966_47b7_a7b5_9970861ef456" initialAmount="0"/>

<species id="mwb7dd85d2_03b5_4642_acac_52cc771e3966" name="Sink_PEP" compartment="mw9adfae27_c966_47b7_a7b5_9970861ef456" initialAmount="0"/>

<species id="mwa5c286c8_0311_47bb_a72d_717e0cdfb845" name="Sink_GAP" compartment="mw9adfae27_c966_47b7_a7b5_9970861ef456" initialAmount="0"/>

<species id="mwb14e508a_9ace_4e3b_9f1e_bf1e08ed092c" name="NADPHr" compartment="mw9adfae27_c966_47b7_a7b5_9970861ef456" initialAmount="0"/>

<species id="mw1e496204_0753_45d3_bd82_9fda788734b7" name="rate_CC_1" compartment="mw9adfae27_c966_47b7_a7b5_9970861ef456" initialAmount="0"/>

<species id="mw87db0fff_0a73_42bf_95d7_1e6713c46e1b" name="rate_CC_2" compartment="mw9adfae27_c966_47b7_a7b5_9970861ef456" initialAmount="0"/>

<species id="mwb01373bb_ffeb_430d_8dff_63c07a625355" name="rate_CC_3" compartment="mw9adfae27_c966_47b7_a7b5_9970861ef456" initialAmount="0"/>

<species id="mwd01c9c47_ecd1_4a66_a003_8e46d77efc4d" name="rate_CC_4" compartment="mw9adfae27_c966_47b7_a7b5_9970861ef456" initialAmount="0"/>

<species id="mw0b858a58_d773_4550_b0e4_2a32b21abb24" name="rate_CC_5" compartment="mw9adfae27_c966_47b7_a7b5_9970861ef456" initialAmount="0"/>

<species id="mw819bc32e_2626_4855_8895_5deebd61c820" name="rate_CC_6" compartment="mw9adfae27_c966_47b7_a7b5_9970861ef456" initialAmount="0"/>

<species id="mwa5cca277_aa07_4dd8_a95a_8bceaf30e70e" name="rate_CC_7" compartment="mw9adfae27_c966_47b7_a7b5_9970861ef456" initialAmount="0"/>

<species id="mw64294e9d_58a0_4ffc_8c8a_21c9d8391e5a" name="rate_CC_8" compartment="mw9adfae27_c966_47b7_a7b5_9970861ef456" initialAmount="0"/>

<species id="mw816c234f_a368_4d43_a45f_5a7d12510976" name="rate_CC_9" compartment="mw9adfae27_c966_47b7_a7b5_9970861ef456" initialAmount="0"/>

<species id="mw9944c18e_1f0c_4a7a_abdb_b1b56d72a673" name="rate_CC_10" compartment="mw9adfae27_c966_47b7_a7b5_9970861ef456" initialAmount="0"/>

<species id="mwd022fa3d_5c26_490a_b4a4_51ddcf64258c" name="rate_CC_11" compartment="mw9adfae27_c966_47b7_a7b5_9970861ef456" initialAmount="0"/>

<species id="mw19a93cbf_18c6_4427_8e26_1fa9e7dad171" name="rate_CC_12" compartment="mw9adfae27_c966_47b7_a7b5_9970861ef456" initialAmount="0"/>

<species id="mw058a38dd_174d_4e6e_a7d9_26b31cc47d39" name="rate_CC_13" compartment="mw9adfae27_c966_47b7_a7b5_9970861ef456" initialAmount="0"/>

<species id="mwfd2ced55_7a6c_4e46_89e8_6e09c111839b" name="rate_PFK" compartment="mw9adfae27_c966_47b7_a7b5_9970861ef456" initialAmount="0"/>

<species id="mwd8b2ff5c_3c77_43ff_8fad_9c93171bb747" name="rate_PGM" compartment="mw9adfae27_c966_47b7_a7b5_9970861ef456" initialAmount="0"/>

<species id="mw8b51e2ce_42bd_422e_96b0_79b39239a4a9" name="rate_ENO" compartment="mw9adfae27_c966_47b7_a7b5_9970861ef456" initialAmount="0"/>

<species id="mw7129d4a5_ea33_4780_a37b_1cc553e6cbe6" name="rate_glyc_GAP" compartment="mw9adfae27_c966_47b7_a7b5_9970861ef456" initialAmount="0"/>

<species id="mwca20acee_79f0_416c_9f8d_d598757960ee" name="rate_PEPsink" compartment="mw9adfae27_c966_47b7_a7b5_9970861ef456" initialAmount="0"/>

<species id="mw2e29aa28_0bc5_4b38_88a0_c885fd689c98" name="Sink_G6P" compartment="mw9adfae27_c966_47b7_a7b5_9970861ef456" initialAmount="0"/>

<species id="mwd279fef9_0d64_43b6_811b_d126265b2623" name="rate_G6Psink" compartment="mw9adfae27_c966_47b7_a7b5_9970861ef456" initialAmount="0"/>

<species id="mw606af7f8_db79_45f3_896c_81838de43916" name="rate_GAPsink" compartment="mw9adfae27_c966_47b7_a7b5_9970861ef456" initialAmount="0"/>

<species id="mw390bea38_1b39_4962_8f10_962e6ca96e3d" name="rate_E4Psink" compartment="mw9adfae27_c966_47b7_a7b5_9970861ef456" initialAmount="0"/>

<species id="mwf6dc3a41_68d1_481a_a511_3aadf99e49b0" name="rate_Ri5Psink" compartment="mw9adfae27_c966_47b7_a7b5_9970861ef456" initialAmount="0"/>

<species id="mw543ef166_8322_44f0_9435_270a9f9672da" name="AceP" compartment="mw9adfae27_c966_47b7_a7b5_9970861ef456" initialAmount="0"/>

<species id="mw92f0b5a5_287d_4b4e_8e20_756a2c2a2fd7" name="Sink_AceP" compartment="mw9adfae27_c966_47b7_a7b5_9970861ef456" initialAmount="0"/>

<species id="mwb8027448_5c51_43ad_9f13_cd37b5418a54" name="P6G" compartment="mw9adfae27_c966_47b7_a7b5_9970861ef456" initialAmount="0"/>

<species id="mw98b97e83_73fb_4ec8_8618_438ef4ddc02c" name="OPP_rate" compartment="mw9adfae27_c966_47b7_a7b5_9970861ef456" initialAmount="0"/>

<species id="mw618971c6_5583_444a_880c_62858550103b" name="rate_GSM1" compartment="mw9adfae27_c966_47b7_a7b5_9970861ef456" initialAmount="0"/>

<species id="mwb8f6ab34_7de8_40a2_be1f_6b28d461b072" name="netC6_PFK" compartment="mw9adfae27_c966_47b7_a7b5_9970861ef456" initialAmount="0"/>

<species id="mw8425df07_de4f_4a79_9f4a_0a9334146b90" name="rate_PKET1" compartment="mw9adfae27_c966_47b7_a7b5_9970861ef456" initialAmount="0"/>

<species id="mw606dccc8_184f_4101_85c0_fff997c104ca" name="rate_PKET2" compartment="mw9adfae27_c966_47b7_a7b5_9970861ef456" initialAmount="0"/>

<species id="mw3c3b7565_7ecb_4d83_a206_818265293cb6" name="Pyr" compartment="mw9adfae27_c966_47b7_a7b5_9970861ef456" initialAmount="0"/>

<species id="mw1dc47a27_16ad_4efd_8f31_09fac64ba939" name="rate_EDglyc" compartment="mw9adfae27_c966_47b7_a7b5_9970861ef456" initialAmount="0"/>

<species id="mw0103addc_790d_4201_8a92_819af4a321ef" name="rate_GPI" compartment="mw9adfae27_c966_47b7_a7b5_9970861ef456" initialAmount="0"/>

<species id="mw2fe938ce_356d_4915_ac09_45c087e56003" name="rate_glucose" compartment="mw9adfae27_c966_47b7_a7b5_9970861ef456" initialAmount="0"/>

<species id="mwafafac3f_863a_498f_94e9_aa0a56a97576" name="rate_PFK1" compartment="mw9adfae27_c966_47b7_a7b5_9970861ef456" initialAmount="0"/>

<species id="mwafe48bf9_dc16_438d_a0de_9e4d98501709" name="rate_PFK2" compartment="mw9adfae27_c966_47b7_a7b5_9970861ef456" initialAmount="0"/>

<species id="mw3d63e90e_4692_4dfd_a839_dd3307b0e55b" name="rate_ALDO1_CC_5" compartment="mw9adfae27_c966_47b7_a7b5_9970861ef456" initialAmount="0"/>

<species id="mwe559609f_0aab_4074_907d_7dd88bc5f228" name="rate_ALDO2_CC_5" compartment="mw9adfae27_c966_47b7_a7b5_9970861ef456" initialAmount="0"/>

<species id="mwb68b7376_788d_498e_b36d_36c89eeb1967" name="rate_PGM1" compartment="mw9adfae27_c966_47b7_a7b5_9970861ef456" initialAmount="0"/>

<species id="mw20119d5d_b91a_40db_882e_a27a7dacd159" name="rate_PGM2" compartment="mw9adfae27_c966_47b7_a7b5_9970861ef456" initialAmount="0"/>

<species id="mw579e0b52_171d_4945_a0c2_8b5ab7cea441" name="rate_PGM3" compartment="mw9adfae27_c966_47b7_a7b5_9970861ef456" initialAmount="0"/>

<species id="mw76598380_dad6_4fbd_a40b_7281ee83b607" name="PhotoRes" compartment="mw9adfae27_c966_47b7_a7b5_9970861ef456" initialAmount="0"/>

<species id="mwf1a22b07_09a4_459c_bc0d_7ff0041d087f" name="net_CC_3" compartment="mw9adfae27_c966_47b7_a7b5_9970861ef456" initialAmount="0"/>

<species id="mw61ee8550_d991_4a56_922f_f261a0a492f4" name="OPP2_rate" compartment="mw9adfae27_c966_47b7_a7b5_9970861ef456" initialAmount="0"/>

<species id="mw3bab02fa_c9cf_4ae8_baa1_491eac4c7a7f" name="rate_PKET1a" compartment="mw9adfae27_c966_47b7_a7b5_9970861ef456" initialAmount="0"/>

<species id="mwc61d80c8_a1a3_4704_b02a_baaff2bc07f2" name="rate_PKET1b" compartment="mw9adfae27_c966_47b7_a7b5_9970861ef456" initialAmount="0"/>

<species id="mw79196e59_a83a_4059_afcd_3fbbb659acf9" name="rate_PKET2a" compartment="mw9adfae27_c966_47b7_a7b5_9970861ef456" initialAmount="0"/>

<species id="mw837df40e_afa2_42bb_88ef_c02281c03e49" name="rate_PKET2b" compartment="mw9adfae27_c966_47b7_a7b5_9970861ef456" initialAmount="0"/>

```xml
<species id="mwf3e38952_b929_46f3_b15d_3e4a170b3863" name="KDPG" compartment="mw9adfae27_c966_47b7_a7b5_9970861ef456" initialAmount="0"/>

<species id="mwc740f83b_ea86_4945_a732_4b08a717ae97" name="CO2" compartment="mw27e3f884_16a3_4659_ac3d_178dbbae0ed4" initialAmount="0.1" boundaryCondition="true" constant="true"/>

<species id="mw19a9030f_8af8_485a_ba7c_5d89c93164cc" name="G6P" compartment="mw27e3f884_16a3_4659_ac3d_178dbbae0ed4" initialAmount="1" boundaryCondition="true" constant="true"/>

</listOfSpecies>

<listOfParameters>

<parameter id="mw3f34c374_f765_4309_b3fd_9064029073d1" name="kf_ATP" value="1.402940331">

<annotation>

<COPASI xmlns="http://www.copasi.org/static/sbml">

<rdf:RDF xmlns:dcterms="http://purl.org/dc/terms/" xmlns:rdf="http://www.w3.org/1999/02/22-rdf-syntax-ns#">

<rdf:Description rdf:about="#COPASI47">

<dcterms:created>

<rdf:Description>

<dcterms:W3CDTF>2010-04-14T15:40:36Z</dcterms:W3CDTF>

</rdf:Description>

</dcterms:created>

</rdf:Description>

</rdf:RDF>

</COPASI>

</annotation>

</parameter>

<parameter id="mw950bbc4b_e9ec_43a5_84c9_010563093363" name="kf_NADPH" value="0.181695036">

<annotation>

<COPASI xmlns="http://www.copasi.org/static/sbml">

<rdf:RDF xmlns:dcterms="http://purl.org/dc/terms/" xmlns:rdf="http://www.w3.org/1999/02/22-rdf-syntax-ns#">

<rdf:Description rdf:about="#COPASI48">
```

```xml
<dcterms:created>
<rdf:Description>
<dcterms:W3CDTF>2010-07-25T02:11:54Z</dcterms:W3CDTF>
</rdf:Description>
</dcterms:created>
</rdf:Description>
</rdf:RDF>
</COPASI>
</annotation>
</parameter>
<parameter id="mw70d358f1_f93f_432d_a6a3_d1aba42c9db7" name="V_PP1" value="0.038"/>
<parameter id="mwc13f7f00_20f5_4411_8231_622067f80bcb" name="V1" value="1.568192157"/>
<parameter id="mw538c4cd4_f0aa_4fd6_8f8a_11b3e718bdca" name="V5" value="1.357814138"/>
<parameter id="mw67b39301_8673_4822_8409_6fb4afa9cfa4" name="V2" value="1.096446984"/>
<parameter id="mw202ff497_360c_4c69_8636_b9de7c9dd611" name="V3" value="1.619516113"/>
<parameter id="mw22fbdd6b_5631_460f_8df1_6a49a247a909" name="V4" value="1.913060355"/>
<parameter id="mw69b6dfd1_e329_427b_a2e2_a5d668d300ca" name="V6" value="2.029800303"/>
<parameter id="mw9e42a158_2ae8_49da_9a6e_635d6a018a2b" name="V7" value="0.67493523"/>
<parameter id="mw2e164e0f_6a15_43c9_a62c_cc3a85074287" name="V8" value="1.299119294"/>
<parameter id="mw676816c3_3653_49ac_9693_a838ec5f9446" name="V9" value="2.0785"/>
<parameter id="mw509e62ff_43c2_439f_a219_323f6f557755" name="V10" value="1.136460632"/>
<parameter id="mwf878e33d_3228_40a3_8fa6_9865294de7a1" name="V11" value="0.444597323"/>
<parameter id="mw8f309108_f60b_445c_af16_065b0479e746" name="V12" value="2.005451913"/>
```

```xml
<parameter id="mw6ab10457_9720_4445_a710_232dd2c4547d" name="V13" value="1.667892984"/>
<parameter id="mwcb0e41ed_453b_4010_8628_3c3dc91f2591" name="V_SS1" value="1.082942006"/>
<parameter id="mwcff4a43c_2777_4532_a542_a418d54de341" name="KE_SS1" value="0.736493671"/>
<parameter id="mwd116d1da_a4e2_47b1_b71c_643640c7faac" name="V_Sink_E4P" value="0.2687053392"/>
<parameter id="mw089625ae_78a0_4e60_953d_7bccf06a6d3f" name="V_Sink_Ri5P" value="0.002"/>
<parameter id="mw45b50366_2e2e_4340_a159_d5255fbb5599" name="V_PP2a" value="0.0007"/>
<parameter id="mw83c2564e_fce9_4d65_ad89_f4ec9d59e973" name="V_PP3" value="0.0042"/>
<parameter id="mw20d5178d_9286_49d1_8c12_d413a39a7d7a" name="V_PP4" value="0.43"/>
<parameter id="mw2d5b6f12_0344_4f87_8e82_bc9605427f64" name="V_PP5" value="0.002"/>
<parameter id="mwf9b6aec2_50da_4731_963c_67dca8babe88" name="V_PP6" value="0.004"/>
<parameter id="mwf1be6830_c2ee_4d96_a417_fb1e9115d10b" name="V_PP7" value="0.013"/>
<parameter id="mwbba6a282_e627_49c3_9f45_889c3993c575" name="V_Sink_PEP" value="0.021"/>
<parameter id="mw02f9621e_b863_4168_a11d_fa2753817db7" name="V_synth_SER" value="0.0008"/>
<parameter id="mw2ec57d34_aee7_4157_a680_a2a0ed50f517" name="V_TSA1" value="0.01"/>
<parameter id="mw4732b2f8_32c5_4514_b508_4dc1858f5f4c" name="V_TSA2" value="0.023"/>
<parameter id="mwaa87b632_3faa_428a_b731_6d7b21f7b26f" name="V_TSA3" value="0.1"/>
<parameter id="mw7bde3bd4_7b60_4626_a4cd_83436934369a" name="V_OXA1" value="0.12"/>
<parameter id="mwca5c5991_e869_4364_8240_76e056ea0d3a" name="V_OXA2" value="0.0002"/>
<parameter id="mw4d8c7a18_e85a_4f29_aca5_7580ecebdacf" name="KE4" value="0.774282734"/>
<parameter id="mw97296fac_b8d0_4286_8c45_a7ae55aeab22" name="KE7" value="1.036991236"/>
<parameter id="mw47ef96f3_f55b_4c2f_aeb6_7b00be2f7c33" name="V_PFK" value="0.343918353"/>
<parameter id="mw8cce0768_8b1e_4e92_a94e_f02e7edd81a9" name="Km112b" value="0.1"/>
```

```xml
<parameter id="mw08c7da94_cfbc_4e2b_aad0_861e59694355" name="V_PP2b" value="0.0005"/>

<parameter id="mw2a28fd49_4cc1_45af_b6c5_f28199557faa" name="Km112a" value="3"/>

<parameter id="mw8e5540c2_ecd1_4e3e_bb5a_651353fa0ec2" name="Kms_enol" value="0.872622166"/>

<parameter id="mwb8a92b6d_ce94_48d0_8b18_58660e599368" name="Kmp_enol" value="1.799549381"/>

<parameter id="mw10c7c233_9065_48c8_8a5c_8a27f4ee15d6" name="K_Sink_PEP" value="0.389230022"/>

<parameter id="mw3c3bc745_1bf5_4845_98a6_dbaa2aedd575" name="V_Sink_GLY" value="0.0002"/>

<parameter id="mw1a0030a7_698d_4add_adca_187e18575335" name="V_Sink_SER" value="0.00194"/>

<parameter id="mwa345d9e1_655b_41de_bcb1_d82b21b3c42c" name="KE5" value="0.005071952"/>

<parameter id="mw3f9f4337_1baa_4946_8e90_d13ca5c3adbf" name="Keq_PGM" value="0.787013653"/>

<parameter id="mw28d0b788_21a0_4f17_ac4c_9b32d44ae058" name="Kmp_PGM_alpha" value="0.699789407"/>

<parameter id="mw7d7f5782_23e4_4797_8dc7_355ec98f4d6a" name="Kms_PGM_alpha" value="0.185455682"/>

<parameter id="mwa577376f_a6e1_47eb_bf4d_2b6dc53f1249" name="Vf_PGM_alpha" value="0.997004957"/>

<parameter id="mw8a2c7a1e_c472_4f06_a4c7_38688feecabc" name="Kmp_PGM_beta" value="1.106740159"/>

<parameter id="mwc0011b36_3da6_4d34_9157_28ac5f77a5df" name="Kms_PGM_beta" value="1.796944911"/>

<parameter id="mw4674ad99_0bbe_4aef_99fa_49937a6f49d7" name="Vf_PGM_beta" value="1.283099826"/>

<parameter id="mwc5650e27_4e7c_4064_8600_9ff3fd5f22ca" name="Kmp_PGM_gama" value="0.685918123"/>

<parameter id="mw56023d39_08c8_49e5_a347_a28c871b8290" name="Kms_PGM_gama" value="0.710421809"/>

<parameter id="mw11591b16_f15e_41a6_890c_7da51ea27d51" name="Vf_PGM_gama" value="1.058569945"/>

<parameter id="mw963788fc_9773_4082_8fe4_0f8ea1e81867" name="Keq_enol" value="1.075635449"/>
```

```xml
<parameter id="mw20c2fea9_d469_4e35_bdf8_38cbe9f5bd92" name="Vf_enol" value="0.968667274"/>

<parameter id="mwf78a87c8_a526_4514_ad30_2df654c3ee39" name="kf_CO2" value="0.402005423"/>

<parameter id="mwf7816b82_0693_49ca_9589_4c1ec5f35110" name="V_Sink_GAP" value="0.00039"/>

<parameter id="mw9c17270d_1b98_4c92_8c4f_0bf91f94ac5c" name="K_Sink_GAP" value="0.1"/>

<parameter id="mw4da83b47_6347_43ed_b1b6_c95ab9075f9d" name="Kgap" value="1.9675848"/>

<parameter id="mw640c5b04_67dd_43d7_8b11_069cf242288b" name="Knadpp" value="0.971222067"/>

<parameter id="mw02a594bf_b380_46b9_acac_e7344c88da82" name="Kpga" value="1.510982799"/>

<parameter id="mw38ba402b_328c_454f_807b_2a5d33b8d48f" name="Vgap_dehyd" value="0.916523336"/>

<parameter id="mwebd2cdb3_d654_4e7e_a036_7180e1269833" name="knadph" value="0.498444608"/>

<parameter id="mwceaf4424_f60b_4cab_8ae8_9826a4f7860c" name="KM61" value="0.503756605"/>

<parameter id="mw9de2bbc0_b1db_4824_9105_762861f97d06" name="KE8" value="1.560413758"/>

<parameter id="mw9d6196a1_62e0_4989_84af_dba7005fa377" name="KE10" value="2.081344186"/>

<parameter id="mwa92c6c71_d522_4d07_9ef1_f6f2c17f9c71" name="KE11" value="1.004500224"/>

<parameter id="mw93964b11_67c1_4727_84cc_949cd4f6ecf4" name="KE12" value="0.726101248"/>

<parameter id="mwf6529569_4b56_44fa_993e_f2741a368c08" name="KM132" value="0.05"/>

<parameter id="mwee200f01_e65f_45cf_b143_f20ca0b735f9" name="K_GOA" value="0.1"/>

<parameter id="mw8120d5c3_18c9_485d_99c5_bdf56d90f8c2" name="V_GLY_syn" value="0.1"/>

<parameter id="mw364ec325_c2af_4413_8934_e8b8af09f5f4" name="KM11" value="1.253438978"/>

<parameter id="mwe3972b9a_3c7e_4eb9_aa83_6bc48e5251c9" name="KM12" value="0.969894284"/>
```

```xml
<parameter id="mw25c38b1f_a0cf_41ea_a0b9_ce591ec4bb41" name="KI11" value="0.922807205"/>

<parameter id="mwef7f5344_1be5_414e_a10b_43c1f1ba59bb" name="KI14" value="1.190147787"/>

<parameter id="mw1aa79476_50cf_4ad8_b34b_1bd559d0e8b7" name="KM13" value="0.876021122"/>

<parameter id="mwb9ff1179_1b4d_4f43_a08e_07a71dc0e53a" name="KI12" value="0.602579799"/>

<parameter id="mwb765042e_909e_4002_adec_27be965841a3" name="KI13" value="0.898563275"/>

<parameter id="mwaa92dfc9_9a33_4094_91a8_216d11908ae3" name="KI15" value="0.402132411"/>

<parameter id="mw9896ea60_a024_4836_a456_1e419a52deb6" name="KE2" value="0.957269047"/>

<parameter id="mw2b7af3fa_4bf7_41ff_9e31_e4d8ae1a0567" name="KM31" value="1.303128177"/>

<parameter id="mw1243747a_17f7_4824_8201_696b9245f34d" name="KM32" value="1.669061041"/>

<parameter id="mwf7770ee0_1efd_4f73_a3f0_925cae9eaca8" name="KI62" value="0.432192112"/>

<parameter id="mwd48591b6_3ea8_4408_9e19_391e457c863d" name="KI61" value="0.898316943"/>

<parameter id="mw459ad12f_127a_4f15_a4ca_dd8d9d3ca505" name="KI135" value="0.932480779"/>

<parameter id="mw620d20e1_7007_4d40_9ee4_67bc35938f33" name="KI131" value="0.338661727"/>

<parameter id="mw3ca15696_2c16_4bbe_9acc_5bb19882a03e" name="KI134" value="1.947628591"/>

<parameter id="mw5011fe71_c328_4107_9245_0861c6dbfb16" name="KI133" value="1.747256569"/>

<parameter id="mwa3e0f729_e97d_443e_a14a_9eb9891474e1" name="KI132" value="0.52967058"/>

<parameter id="mwa97791f8_5c29_40fd_8db4_b61695f0810e" name="KM131" value="1.747163467"/>

<parameter id="mwd7d873d8_453a_431d_a268_fff0b2981096" name="K_Sink_G6P" value="1.854194713"/>

<parameter id="mw6b34d5e4_8388_4d61_837c_26f3c9c09838" name="V_Sink_G6P" value="0.0943097295"/>
```

```xml
<parameter id="mwe2e6283d_1779_48e0_93fd_a0e7744d0f49" name="K_Sink_AceP" value="1"/>
<parameter id="mw72813c68_f9ad_4693_a385_08d49dcb3b9c" name="V_Sink_AceP" value="1"/>
<parameter id="mwa61fcd80_7091_43db_bd8f_5686bc24a7ee" name="K_OXA1" value="2"/>
<parameter id="mw1d4b50a5_6beb_46eb_8599_024c83c627b2" name="K_OXA2" value="5"/>
<parameter id="mwe08f3352_d986_4a9a_acea_33701d19a3b3" name="V_PFK_beta" value="0.518378893"/>
<parameter id="mwa4a1fe58_2c6a_4f99_8001_4a0fedc9a357" name="Kmp_PPP1" value="0.1"/>
<parameter id="mwb23b6c01_848a_44fb_8324_dda82ffcb0c4" name="Kms_PPP1" value="2"/>
<parameter id="mw14fbc3da_1eb6_4470_8032_06f6d5ce7c93" name="V_PPP1" value="0.81"/>
<parameter id="mwf24759b0_36bc_4a60_b869_301f6c1f2d80" name="Kms_nadpp_ppp2" value="1"/>
<parameter id="mw9e08e044_96ba_486f_9778_f5a4ea04cff4" name="Kms_p6g_ppp2" value="1.2"/>
<parameter id="mw376c4606_38fa_4a60_9b24_8ef462cbe2ff" name="V_PPP2" value="0.45"/>
<parameter id="mw03716e78_6785_43c8_9249_0ab554cf9111" name="K_Sink_E4P" value="1.496276951"/>
<parameter id="mw4782b972_ca60_4b9e_96dd_9dba1be35a3e" name="K_Sink_Ri5P" value="0.683251376"/>
<parameter id="mw9415f414_35fe_45d1_a3c7_d7c0b56ef439" name="KI1121" value="94"/>
<parameter id="mwe0680e97_a6e6_4cca_94b1_395c1f98c3b8" name="KI1122" value="2.55"/>
<parameter id="mwe7cd87c9_87ad_458d_b881_b18d32ab3919" name="Km112c" value="0.5"/>
<parameter id="mw273ec436_6071_4799_870d_095c01364042" name="V_PP2c" value="0.001"/>
<parameter id="mwb84f53a1_12bb_45c4_bc48_32fddf334c72" name="V8b" value="0"/>
<parameter id="mw17a7c44a_3eb3_4030_b0d2_f885efc0f98b" name="K_TSA1" value="0.1"/>
<parameter id="mw5768ed07_e6fa_4e82_994f_f0f9a1a0a3a8" name="K_TSA2" value="0.4"/>
<parameter id="mw92d1a502_da48_4a89_9ea0_8831de942be8" name="K_TSA3" value="0.1"/>
<parameter id="mw7b8f321d_ca9c_4a2b_b7e7_44d5fca51f8d" name="K_Sink_SER" value="1"/>
<parameter id="mwdd6e9231_51b9_4b67_b71c_24c2e4af56ba" name="K_synth_SER" value="2"/>
<parameter id="mw84076d99_c4a9_4fcf_b194_46b4503635be" name="K_Sink_GLY" value="1"/>
```

```xml
<parameter id="mwfc079b4e_b465_4bbe_b1ae_11a68246902a" name="Km121" value="0.1"/>
<parameter id="mw216d7d9c_5cbe_472f_a388_64d7d3889347" name="Km1231" value="0.09"/>
<parameter id="mwb4c4eaed_f8bb_4d91_bf8e_f0943ebabb45" name="Km1131" value="0.21"/>
<parameter id="mwbf3b18c6_626f_464e_8498_a175942a2b66" name="Km1132" value="0.25"/>
<parameter id="mw0bdbb111_5454_4f47_9918_8da9990953f9" name="Km1241" value="0.15"/>
<parameter id="mw14d17aaf_3016_4fb0_b90c_6b70ea2eeb64" name="Km1242" value="1.7"/>
<parameter id="mw48abd3c1_84c1_4bfe_9c74_0720c0b36bc2" name="KI124" value="2"/>
<parameter id="mwee6b22b8_8ca2_4b43_8638_750582325854" name="KE113" value="300"/>
<parameter id="mwe32e388e_050d_4321_991e_7156fdc6d321" name="KI113" value="0.36"/>
<parameter id="mw4c0a5f49_cf9c_4137_a4d1_a27a9c633c60" name="KE124" value="607"/>
<parameter id="mw05e98de4_3951_43e5_ac00_ca56fb3f40d3" name="KE123" value="250000"/>
<parameter id="mw55f32731_601d_4813_a73f_80bd3d7c7fa6" name="KI123" value="12"/>
<parameter id="mwc92f9b3a_36c7_4c3f_a179_2eb02421e208" name="KM_ATP_PFK" value="1.01504684"/>
<parameter id="mwfab2bb7b_fa67_47d4_b71e_f36e49d4c2f7" name="KM_F6P_PFK" value="0.986266878"/>
<parameter id="mw9bc87645_2d3f_4a39_8dff_d0074e096984" name="KM_ATP_PFK_beta" value="0.124144755"/>
<parameter id="mwd2e258fa_4740_4b3b_816e_0b0e55bdb005" name="KM_F6P_PFK_beta" value="0.405755263"/>
<parameter id="mw8204a9d2_3043_47db_96f6_061c456138f4" name="KM2_ADP" value="1.220267993"/>
<parameter id="mwcc3e1093_f15d_43ba_803f_07c7f8d7c816" name="KM2_ATP" value="0.308741401"/>
<parameter id="mwdb043b2e_d92d_4148_accd_2ed71aad1897" name="KM2_BPGA" value="0.137012694"/>
<parameter id="mwc2cdab10_3c94_4f4b_b95b_d8af8de7d09e" name="KM2_PGA" value="0.87545294"/>
<parameter id="mw60b27f8d_8f84_4edc_b378_ca1bab69e489" name="KM4_DHAP" value="0.531003962"/>
<parameter id="mw4372d7eb_dfbb_483c_ac0d_eeaa38580e0b" name="KM4_GAP" value="0.379798711"/>
```

```xml
<parameter id="mw6e554aaa_4cb8_46a2_9af0_40c527042d95" name="KM5_8_DHAP" value="0.004082427"/>
<parameter id="mw2ffe78db_96a4_4d1b_a62f_f84d7987e64e" name="KM5_8_E4P" value="0.018870081"/>
<parameter id="mwef61df7c_5b85_41cf_b539_58e829001c39" name="KM5_8_FBP" value="0.343225357"/>
<parameter id="mwb4c2b40c_c637_4023_8d0f_5ed2bc9febf1" name="KM5_8_GAP" value="2.154044217"/>
<parameter id="mw9d553771_b33e_40de_bbc5_22c5804e335a" name="KM5_8_SBP" value="0.872828777"/>
<parameter id="mwb15d9294_9509_4702_9083_ef51d8c235f9" name="KM5b_8b_DHAP" value="0.6438"/>
<parameter id="mwc410441d_bd1d_4085_9e21_08b662e4f672" name="KM5b_8b_E4P" value="0.1581"/>
<parameter id="mw1e28fdab_0576_4966_9927_17b091f2a4b2" name="KM5b_8b_FBP" value="1.772"/>
<parameter id="mw8b39e257_03b8_4927_97c6_4b8444f70966" name="KM5b_8b_GAP" value="2.3765"/>
<parameter id="mw777c1f25_305f_42cf_a010_b537a05884dd" name="KM5b_8b_SBP" value="1.73"/>
<parameter id="mwaad1b589_216c_4c43_92a0_68a64eaa5b3b" name="V5b" value="0"/>
<parameter id="mwac1e88d0_dca5_49b3_9950_4e0560efa626" name="KM9_FBP" value="0.720287962"/>
<parameter id="mw5fc73772_4633_4bf8_9aa6_fbabe153e1c8" name="KM9_SBP" value="0.420060602"/>
<parameter id="mw1940c877_4e88_4a71_957a_5160dc7cfd3c" name="KM7_10_E4P" value="0.791392188"/>
<parameter id="mw7e01e1f4_5a8e_40f5_805c_6bb66e4302d5" name="KM7_10_F6P" value="0.842692338"/>
<parameter id="mwd82e6763_7382_481e_82d1_193b24b364f8" name="KM7_10_GAP" value="1.031932132"/>
<parameter id="mw545ed108_3a36_4f08_880d_e713dae6443a" name="KM7_10_Ri5P" value="0.097093867"/>
<parameter id="mw57be342f_c610_40fc_8742_cd5cac73cf4a" name="KM7_10_S7P" value="0.428923737"/>
<parameter id="mw328820c4_827f_4b41_ace1_19213258ac79" name="KM7_10_Xu5P" value="2.220401121"/>
```

```xml
<parameter id="mwc31a14d7_9c22_4389_99c6_58d27312cdb7" name="KM11_Ri5P" value="0.554712127"/>
<parameter id="mwd908059f_c845_4440_addd_e491a150ff80" name="KM11_Ru5P" value="1.055763099"/>
<parameter id="mw1c952255_2606_4fe2_9e07_47ea2266367e" name="KM12_Ru5P" value="1.389791739"/>
<parameter id="mw9a22bae8_8f9c_4a94_a7ab_127fa8a4d3bd" name="KM12_Xu5P" value="0.664119927"/>
<parameter id="mwb7105ee5_87bd_444a_aa8c_74b7fadd678a" name="KM_Sink_Pyr" value="0.021"/>
<parameter id="mwb2bc9da8_6f54_4986_b142_b256c7b7bc82" name="V_Sink_Pyr" value="0.6"/>
<parameter id="mwbc1c0f08_cf5f_4e06_a14b_d5c559bb147c" name="kf_G6P" value="0.0066"/>
<parameter id="mw467513d1_2f1d_4bc8_b5d4_2707b9c5db6a" name="KM_F6P_SS1" value="0.683262245"/>
<parameter id="mwca8937d4_62a3_442e_bcb6_13c0d0166036" name="KM_G6P_SS1" value="1.08216983"/>
<parameter id="mw514d5907_3da1_4e17_8024_f637af4b38fa" name="KM_PKET1a_AceP" value="1.765818327"/>
<parameter id="mw8b16f07a_00d8_489e_ae07_2dc92af4dfb9" name="KM_PKET1a_E4P" value="0.708995427"/>
<parameter id="mw8e6b4f11_baec_4a0f_983d_3d1d46cb91cb" name="KM_PKET1a_F6P" value="0.195859927"/>
<parameter id="mw9d34e88a_6503_48b9_a787_58c326a932a5" name="KM_PKET1a_GAP" value="0.001101781"/>
<parameter id="mw562c606a_6226_47bc_8fe9_db84aa73a146" name="KM_PKET1a_Xu5P" value="1.255418228"/>
<parameter id="mw066182b3_2182_44b1_b745_125e1968e0bc" name="Keq_PKETa" value="0.2896651"/>
<parameter id="mw6e7fabe6_de80_41fa_9146_5e9b7ec4bc63" name="V_PKET1a" value="1.903146406"/>
<parameter id="mw6dff67cf_5c46_4621_9902_c290a3f63d5d" name="KM_PKET1b_AceP" value="0.674920019"/>
<parameter id="mw9580aa5c_a5df_472e_aad4_d7d8dfaf50c5" name="KM_PKET1b_E4P" value="0.973496654"/>
<parameter id="mw77007da8_e432_48eb_9c52_ed3c2c1b5f8c" name="KM_PKET1b_F6P" value="0.073704394"/>
```

```xml
<parameter id="mwa0585ea2_d52f_4310_adf2_e8de58065ae9" name="KM_PKET1b_GAP" value="0.126696262"/>
<parameter id="mw057e7327_64bf_4b85_bb90_78da6ba5980f" name="KM_PKET1b_Xu5P" value="0.315391885"/>
<parameter id="mw44c8ec20_4a1e_454f_a1a0_42d862b117ae" name="Keq_PKETb" value="0.471060325"/>
<parameter id="mw0ab3c1a9_2d85_4242_b2c8_8668592bc4b4" name="V_PKET1b" value="0.1653682668"/>
<parameter id="mwc6a7cd20_1464_4521_88e6_145752ed585d" name="KM_PKET2b_AceP" value="0.708589676"/>
<parameter id="mw4c5adb36_23ae_4318_8b5f_6f80df2be00a" name="KM_PKET2b_E4P" value="0.001478515"/>
<parameter id="mw08f2e5c2_6905_4c90_8f37_016935f5d8ec" name="KM_PKET2b_F6P" value="0.71897234"/>
<parameter id="mw8d162e7c_404d_4423_8f50_9c55f37e0d2c" name="KM_PKET2b_GAP" value="0.051244493"/>
<parameter id="mwff222c2b_bc40_4d59_bfcd_63aeb04c86bd" name="KM_PKET2b_Xu5P" value="0.79285068"/>
<parameter id="mw5a9817b0_1b0d_4667_a467_59f6072696c8" name="V_PKET2b" value="0.906381388"/>
<parameter id="mw9f4be612_37b6_425f_863f_c3234aaafd9c" name="KM_PKET2a_AceP" value="0.347537253"/>
<parameter id="mw4d62dae0_b6d6_45ba_8fb6_caf10d3295dc" name="KM_PKET2a_E4P" value="0.564845446"/>
<parameter id="mw7c84a538_b915_4a91_8cc2_04c534c01ab8" name="KM_PKET2a_F6P" value="0.21030757"/>
<parameter id="mwa723f027_cc0e_426d_b99e_dd1f00ba7a3e" name="KM_PKET2a_GAP" value="1.219287928"/>
<parameter id="mw457a9b1f_ac25_45b4_adb7_c6c807be3474" name="KM_PKET2a_Xu5P" value="0.669382423"/>
<parameter id="mw9e5312fb_9ca1_4ae7_8fef_e40c70633af3" name="V_PKET2a" value="0.000538102"/>
<parameter id="mw7f5b93ed_2736_43c7_9104_bc8302ad8c01" name="KI_KDPG" value="1.5"/>
<parameter id="mw5ceb33ad_a5ce_4d67_8217_5ac758441102" name="Km_edd_P6G" value="1"/>
<parameter id="mw518ffec0_9f71_433e_9dc8_9ca216769de1" name="V_edd" value="0.0158"/>
```

```
      <parameter id="mw9674d404_dd5e_47e7_a284_60e128055d71" name="Km_eda_KDPG"
value="1"/>
      <parameter id="mwf79253b7_efe2_42f5_bacb_32ceeaccf8cc" name="V_eda" value="0.1"/>
    </listOfParameters>
    <listOfRules>
      <assignmentRule metaid="repeatedAssignment_mw1ddbf5d3_643c_46d3_9775_f1a3eaa0856b"
variable="mwc4d744f3_4e27_4a3b_8596_20885c0a4f55">
        <math xmlns="http://www.w3.org/1998/Math/MathML">
          <apply>
            <divide/>
            <ci> mw8e2cc29c_c73e_4258_b3ba_2356620bb64e </ci>
            <apply>
              <plus/>
              <ci> mw8e2cc29c_c73e_4258_b3ba_2356620bb64e </ci>
              <ci> mwe8d1ae1f_35f9_45fd_bedf_7dbeef3972e2 </ci>
            </apply>
          </apply>
        </math>
      </assignmentRule>
      <assignmentRule metaid="repeatedAssignment_mwa3912842_25ba_4c93_a71f_14fe58e303bb"
variable="mwb14e508a_9ace_4e3b_9f1e_bf1e08ed092c">
        <math xmlns="http://www.w3.org/1998/Math/MathML">
          <apply>
            <divide/>
            <ci> mwd2e6e004_23f5_4cd8_81e0_8385021ec904 </ci>
            <apply>
              <plus/>
              <ci> mwd2e6e004_23f5_4cd8_81e0_8385021ec904 </ci>
              <ci> mw7c834bb1_1865_4950_aa20_f3eceb92b21d </ci>
            </apply>
          </apply>
        </math>
```

```
</assignmentRule>
<assignmentRule metaid="repeatedAssignment_mweb911db8_c719_4c30_81b6_cd198999c8a7" variable="mw1e496204_0753_45d3_bd82_9fda788734b7">
  <math xmlns="http://www.w3.org/1998/Math/MathML">
    <apply>
      <divide/>
      <apply>
        <times/>
        <ci> mw98fecd51_ba08_4727_98ec_04637a47c5ac </ci>
        <apply>
          <divide/>
          <apply>
            <times/>
            <ci> mwc13f7f00_20f5_4411_8231_622067f80bcb </ci>
            <ci> mwf3d533fa_7176_4543_a1c4_f46f4dc4f8f6 </ci>
          </apply>
          <apply>
            <plus/>
            <ci> mwf3d533fa_7176_4543_a1c4_f46f4dc4f8f6 </ci>
            <apply>
              <times/>
              <ci> mw364ec325_c2af_4413_8934_e8b8af09f5f4 </ci>
              <apply>
                <plus/>
                <cn type="integer"> 1 </cn>
                <apply>
                  <divide/>
                  <ci> mw739c8f22_6a61_4dca_8cfe_3c962adb4128 </ci>
                  <ci> mwe3972b9a_3c7e_4eb9_aa83_6bc48e5251c9 </ci>
                </apply>
              </apply>
```

</apply>
</apply>
</apply>
</apply>
<apply>
<plus/>
<ci> mw98fecd51_ba08_4727_98ec_04637a47c5ac </ci>
<apply>
<times/>
<ci> mw1aa79476_50cf_4ad8_b34b_1bd559d0e8b7 </ci>
<apply>
<plus/>
<cn type="integer"> 1 </cn>
<apply>
<divide/>
<ci> mw372b8247_b11e_4d9b_be9b_e742fd2d3853 </ci>
<ci> mw25c38b1f_a0cf_41ea_a0b9_ce591ec4bb41 </ci>
</apply>
<apply>
<divide/>
<ci> mw2a09f89f_5936_4376_a2ab_4ff1bbbd4153 </ci>
<ci> mwb9ff1179_1b4d_4f43_a08e_07a71dc0e53a </ci>
</apply>
<apply>
<divide/>
<ci> mwacf08e72_f001_4a26_962d_729f504b1ad0 </ci>
<ci> mwb765042e_909e_4002_adec_27be965841a3 </ci>
</apply>
<apply>
<divide/>
<ci> mw854ce178_2796_4118_8690_c13951a897c1 </ci>

<ci> mwef7f5344_1be5_414e_a10b_43c1f1ba59bb </ci>

</apply>

<apply>

<divide/>

<ci> mwd2e6e004_23f5_4cd8_81e0_8385021ec904 </ci>

<ci> mwaa92dfc9_9a33_4094_91a8_216d11908ae3 </ci>

</apply>

</apply>

</apply>

</apply>

</apply>

</math>

</assignmentRule>

<assignmentRule metaid="repeatedAssignment_mwd659a62d_f5c9_4438_b3ad_f9e34c00c6c1" variable="mw87db0fff_0a73_42bf_95d7_1e6713c46e1b">

<math xmlns="http://www.w3.org/1998/Math/MathML">

<apply>

<divide/>

<apply>

<divide/>

<apply>

<times/>

<ci> mw67b39301_8673_4822_8409_6fb4afa9cfa4 </ci>

<apply>

<minus/>

<apply>

<times/>

<ci> mw372b8247_b11e_4d9b_be9b_e742fd2d3853 </ci>

<ci> mw8e2cc29c_c73e_4258_b3ba_2356620bb64e </ci>

</apply>

<apply>

```
      <divide/>
      <apply>
        <times/>
        <ci> mwc8b5cb3b_9f99_46df_a6e4_0126749144fc </ci>
        <ci> mwe8d1ae1f_35f9_45fd_bedf_7dbeef3972e2 </ci>
      </apply>
      <ci> mw9896ea60_a024_4836_a456_1e419a52deb6 </ci>
    </apply>
   </apply>
  </apply>
  <apply>
   <times/>
   <ci> mwc2cdab10_3c94_4f4b_b95b_d8af8de7d09e </ci>
   <ci> mwcc3e1093_f15d_43ba_803f_07c7f8d7c816 </ci>
  </apply>
</apply>
<apply>
  <times/>
  <apply>
   <plus/>
   <cn type="integer"> 1 </cn>
   <apply>
    <divide/>
    <ci> mw372b8247_b11e_4d9b_be9b_e742fd2d3853 </ci>
    <ci> mwc2cdab10_3c94_4f4b_b95b_d8af8de7d09e </ci>
   </apply>
   <apply>
    <divide/>
    <ci> mwc8b5cb3b_9f99_46df_a6e4_0126749144fc </ci>
    <ci> mwdb043b2e_d92d_4148_accd_2ed71aad1897 </ci>
   </apply>
```

</apply>
<apply>
<plus/>
<cn type="integer"> 1 </cn>
<apply>
<divide/>
<ci> mw8e2cc29c_c73e_4258_b3ba_2356620bb64e </ci>
<ci> mwcc3e1093_f15d_43ba_803f_07c7f8d7c816 </ci>
</apply>
<apply>
<divide/>
<ci> mwe8d1ae1f_35f9_45fd_bedf_7dbeef3972e2 </ci>
<ci> mw8204a9d2_3043_47db_96f6_061c456138f4 </ci>
</apply>
</apply>
</apply>
</apply>
</math>
</assignmentRule>
<assignmentRule metaid="repeatedAssignment_mw21f5e397_ec4c_425d_8ae2_29465c96867c" variable="mwb01373bb_ffeb_430d_8dff_63c07a625355">
<math xmlns="http://www.w3.org/1998/Math/MathML">
<apply>
<divide/>
<apply>
<times/>
<ci> mw202ff497_360c_4c69_8636_b9de7c9dd611 </ci>
<ci> mwc8b5cb3b_9f99_46df_a6e4_0126749144fc </ci>
<ci> mwd2e6e004_23f5_4cd8_81e0_8385021ec904 </ci>
</apply>
<apply>

```
          <times/>
          <apply>
            <plus/>
            <ci> mwc8b5cb3b_9f99_46df_a6e4_0126749144fc </ci>
            <ci> mw2b7af3fa_4bf7_41ff_9e31_e4d8ae1a0567 </ci>
          </apply>
          <apply>
            <plus/>
            <ci> mwd2e6e004_23f5_4cd8_81e0_8385021ec904 </ci>
            <ci> mw1243747a_17f7_4824_8201_696b9245f34d </ci>
          </apply>
        </apply>
      </apply>
    </math>
  </assignmentRule>
  <assignmentRule metaid="repeatedAssignment_mw0ee5f67e_bac4_4823_87b4_cf1ef6b2d1a9"
variable="mwd01c9c47_ecd1_4a66_a003_8e46d77efc4d">
    <math xmlns="http://www.w3.org/1998/Math/MathML">
      <apply>
        <divide/>
        <apply>
          <divide/>
          <apply>
            <times/>
            <ci> mw22fbdd6b_5631_460f_8df1_6a49a247a909 </ci>
            <apply>
              <minus/>
              <ci> mw5b528d37_b514_46e5_a018_0173afdae714 </ci>
              <apply>
                <divide/>
                <ci> mw8f67b710_8c1c_453f_a7ec_e9b6a55814fa </ci>
```

<ci> mw4d8c7a18_e85a_4f29_aca5_7580ecebdacf </ci>
</apply>
</apply>
</apply>
<ci> mw4372d7eb_dfbb_483c_ac0d_eeaa38580e0b </ci>
</apply>
<apply>
<plus/>
<cn type="integer"> 1 </cn>
<apply>
<divide/>
<ci> mw5b528d37_b514_46e5_a018_0173afdae714 </ci>
<ci> mw4372d7eb_dfbb_483c_ac0d_eeaa38580e0b </ci>
</apply>
<apply>
<divide/>
<ci> mw8f67b710_8c1c_453f_a7ec_e9b6a55814fa </ci>
<ci> mw60b27f8d_8f84_4edc_b378_ca1bab69e489 </ci>
</apply>
</apply>
</apply>
</math>
</assignmentRule>
<assignmentRule metaid="repeatedAssignment_mwb0d1d26f_1ff9_4dae_9751_8fdbf18b8d31" variable="mw0b858a58_d773_4550_b0e4_2a32b21abb24">
<math xmlns="http://www.w3.org/1998/Math/MathML">
<apply>
<plus/>
<apply>
<divide/>
<apply>

```
  <divide/>
  <apply>
    <times/>
    <ci> mw538c4cd4_f0aa_4fd6_8f8a_11b3e718bdca </ci>
    <apply>
      <minus/>
      <apply>
        <times/>
        <ci> mw5b528d37_b514_46e5_a018_0173afdae714 </ci>
        <ci> mw8f67b710_8c1c_453f_a7ec_e9b6a55814fa </ci>
      </apply>
      <apply>
        <divide/>
        <ci> mw2a09f89f_5936_4376_a2ab_4ff1bbbd4153 </ci>
        <ci> mwa345d9e1_655b_41de_bcb1_d82b21b3c42c </ci>
      </apply>
    </apply>
  </apply>
  <apply>
    <times/>
    <ci> mw6e554aaa_4cb8_46a2_9af0_40c527042d95 </ci>
    <ci> mwb4c2b40c_c637_4023_8d0f_5ed2bc9febf1 </ci>
  </apply>
</apply>
<apply>
  <minus/>
  <apply>
    <plus/>
    <apply>
      <times/>
      <apply>
```

<plus/>
<cn type="integer"> 1 </cn>
<apply>
<divide/>
<ci> mw2a09f89f_5936_4376_a2ab_4ff1bbbd4153 </ci>
<ci> mwef61df7c_5b85_41cf_b539_58e829001c39 </ci>
</apply>
</apply>
<apply>
<plus/>
<cn type="integer"> 1 </cn>
<apply>
<divide/>
<ci> mwacf08e72_f001_4a26_962d_729f504b1ad0 </ci>
<ci> mw9d553771_b33e_40de_bbc5_22c5804e335a </ci>
</apply>
</apply>
</apply>
<apply>
<times/>
<apply>
<plus/>
<cn type="integer"> 1 </cn>
<apply>
<divide/>
<ci> mw8f67b710_8c1c_453f_a7ec_e9b6a55814fa </ci>
<ci> mw6e554aaa_4cb8_46a2_9af0_40c527042d95 </ci>
</apply>
</apply>
<apply>
<plus/>

<cn type="integer"> 1 </cn>
<apply>
<divide/>
<ci> mwaa8b93c8_308a_4d19_b732_22cb31f77620 </ci>
<ci> mw2ffe78db_96a4_4d1b_a62f_f84d7987e64e </ci>
</apply>
</apply>
<apply>
<plus/>
<cn type="integer"> 1 </cn>
<apply>
<divide/>
<ci> mw5b528d37_b514_46e5_a018_0173afdae714 </ci>
<ci> mwb4c2b40c_c637_4023_8d0f_5ed2bc9febf1 </ci>
</apply>
</apply>
</apply>
</apply>
<cn type="integer"> 1 </cn>
</apply>
</apply>
<apply>
<divide/>
<apply>
<divide/>
<apply>
<times/>
<ci> mwaad1b589_216c_4c43_92a0_68a64eaa5b3b </ci>
<apply>
<minus/>
<apply>

```
        <times/>
        <ci> mw5b528d37_b514_46e5_a018_0173afdae714 </ci>
        <ci> mw8f67b710_8c1c_453f_a7ec_e9b6a55814fa </ci>
      </apply>
      <apply>
        <divide/>
        <ci> mw2a09f89f_5936_4376_a2ab_4ff1bbbd4153 </ci>
        <ci> mwa345d9e1_655b_41de_bcb1_d82b21b3c42c </ci>
      </apply>
    </apply>
  </apply>
  <apply>
    <times/>
    <ci> mwb15d9294_9509_4702_9083_ef51d8c235f9 </ci>
    <ci> mw8b39e257_03b8_4927_97c6_4b8444f70966 </ci>
  </apply>
</apply>
<apply>
  <minus/>
  <apply>
    <plus/>
    <apply>
      <times/>
      <apply>
        <plus/>
        <cn type="integer"> 1 </cn>
        <apply>
          <divide/>
          <ci> mw2a09f89f_5936_4376_a2ab_4ff1bbbd4153 </ci>
          <ci> mw1e28fdab_0576_4966_9927_17b091f2a4b2 </ci>
        </apply>
```

```
  </apply>
  <apply>
    <plus/>
    <cn type="integer"> 1 </cn>
    <apply>
      <divide/>
      <ci> mwacf08e72_f001_4a26_962d_729f504b1ad0 </ci>
      <ci> mw777c1f25_305f_42cf_a010_b537a05884dd </ci>
    </apply>
  </apply>
</apply>
<apply>
  <times/>
  <apply>
    <plus/>
    <cn type="integer"> 1 </cn>
    <apply>
      <divide/>
      <ci> mw8f67b710_8c1c_453f_a7ec_e9b6a55814fa </ci>
      <ci> mwb15d9294_9509_4702_9083_ef51d8c235f9 </ci>
    </apply>
  </apply>
  <apply>
    <plus/>
    <cn type="integer"> 1 </cn>
    <apply>
      <divide/>
      <ci> mwaa8b93c8_308a_4d19_b732_22cb31f77620 </ci>
      <ci> mwc410441d_bd1d_4085_9e21_08b662e4f672 </ci>
    </apply>
  </apply>
```

```
              <apply>
                <plus/>
                <cn type="integer"> 1 </cn>
                <apply>
                  <divide/>
                  <ci> mw5b528d37_b514_46e5_a018_0173afdae714 </ci>
                  <ci> mw8b39e257_03b8_4927_97c6_4b8444f70966 </ci>
                </apply>
              </apply>
            </apply>
          </apply>
          <cn type="integer"> 1 </cn>
        </apply>
      </apply>
    </apply>
  </math>
</assignmentRule>
<assignmentRule metaid="repeatedAssignment_mwf565f092_a159_44c0_81cd_b11845a64052"
variable="mw819bc32e_2626_4855_8895_5deebd61c820">
  <math xmlns="http://www.w3.org/1998/Math/MathML">
    <apply>
      <divide/>
      <apply>
        <times/>
        <ci> mw69b6dfd1_e329_427b_a2e2_a5d668d300ca </ci>
        <ci> mw2a09f89f_5936_4376_a2ab_4ff1bbbd4153 </ci>
      </apply>
      <apply>
        <plus/>
        <ci> mw2a09f89f_5936_4376_a2ab_4ff1bbbd4153 </ci>
        <apply>
```

```
            <times/>
            <ci> mwceaf4424_f60b_4cab_8ae8_9826a4f7860c </ci>
            <apply>
              <plus/>
              <cn type="integer"> 1 </cn>
              <apply>
                <divide/>
                <ci> mwfcedd013_9e1c_4e21_9a5a_93ddde2ffc11 </ci>
                <ci> mwd48591b6_3ea8_4408_9e19_391e457c863d </ci>
              </apply>
              <apply>
                <divide/>
                <ci> mw854ce178_2796_4118_8690_c13951a897c1 </ci>
                <ci> mwf7770ee0_1efd_4f73_a3f0_925cae9eaca8 </ci>
              </apply>
            </apply>
          </apply>
        </apply>
      </apply>
    </math>
  </assignmentRule>
  <assignmentRule metaid="repeatedAssignment_mw83feb8b2_da4f_46a8_a26c_5886a5d0dd52" variable="mwa5cca277_aa07_4dd8_a95a_8bceaf30e70e">
    <math xmlns="http://www.w3.org/1998/Math/MathML">
      <apply>
        <divide/>
        <apply>
          <divide/>
          <apply>
            <times/>
            <ci> mw9e42a158_2ae8_49da_9a6e_635d6a018a2b </ci>
```

```
    <apply>
     <minus/>
     <apply>
      <times/>
      <ci> mwfcedd013_9e1c_4e21_9a5a_93ddde2ffc11 </ci>
      <ci> mw5b528d37_b514_46e5_a018_0173afdae714 </ci>
     </apply>
     <apply>
      <divide/>
      <apply>
       <times/>
       <ci> mw7c70a014_6b69_42b3_9fbd_1d63cb722ae5 </ci>
       <ci> mwaa8b93c8_308a_4d19_b732_22cb31f77620 </ci>
      </apply>
      <ci> mw97296fac_b8d0_4286_8c45_a7ae55aeab22 </ci>
     </apply>
    </apply>
   </apply>
   <apply>
    <times/>
    <ci> mw7e01e1f4_5a8e_40f5_805c_6bb66e4302d5 </ci>
    <ci> mwd82e6763_7382_481e_82d1_193b24b364f8 </ci>
   </apply>
  </apply>
  <apply>
   <times/>
   <apply>
    <plus/>
    <cn type="integer"> 1 </cn>
    <apply>
     <divide/>
```

<ci> mwfcedd013_9e1c_4e21_9a5a_93ddde2ffc11 </ci>
<ci> mw7e01e1f4_5a8e_40f5_805c_6bb66e4302d5 </ci>
</apply>
<apply>
<divide/>
<ci> mwaa8b93c8_308a_4d19_b732_22cb31f77620 </ci>
<ci> mw1940c877_4e88_4a71_957a_5160dc7cfd3c </ci>
</apply>
</apply>
<apply>
<plus/>
<cn type="integer"> 1 </cn>
<apply>
<divide/>
<ci> mw5b528d37_b514_46e5_a018_0173afdae714 </ci>
<ci> mwd82e6763_7382_481e_82d1_193b24b364f8 </ci>
</apply>
<apply>
<divide/>
<ci> mw7c70a014_6b69_42b3_9fbd_1d63cb722ae5 </ci>
<ci> mw328820c4_827f_4b41_ace1_19213258ac79 </ci>
</apply>
</apply>
<apply>
<plus/>
<cn type="integer"> 1 </cn>
<apply>
<divide/>
<ci> mweadaac0b_cbd7_4eb3_b892_fe6bf884cfc8 </ci>
<ci> mw57be342f_c610_40fc_8742_cd5cac73cf4a </ci>
</apply>

```
            <apply>
              <divide/>
              <ci> mw26816e75_d092_459e_bb3d_70aafa096e4d </ci>
              <ci> mw545ed108_3a36_4f08_880d_e713dae6443a </ci>
            </apply>
          </apply>
        </apply>
      </apply>
    </math>
  </assignmentRule>
  <assignmentRule metaid="repeatedAssignment_mw4411a4ff_25fc_4f89_8a53_e587fc5b7d52"
variable="mw64294e9d_58a0_4ffc_8c8a_21c9d8391e5a">
    <math xmlns="http://www.w3.org/1998/Math/MathML">
      <apply>
        <plus/>
        <apply>
          <divide/>
          <apply>
            <divide/>
            <apply>
              <times/>
              <ci> mw2e164e0f_6a15_43c9_a62c_cc3a85074287 </ci>
              <apply>
                <minus/>
                <apply>
                  <times/>
                  <ci> mw8f67b710_8c1c_453f_a7ec_e9b6a55814fa </ci>
                  <ci> mwaa8b93c8_308a_4d19_b732_22cb31f77620 </ci>
                </apply>
                <apply>
                  <divide/>
```

<ci> mwacf08e72_f001_4a26_962d_729f504b1ad0 </ci>

<ci> mw9de2bbc0_b1db_4824_9105_762861f97d06 </ci>

</apply>

</apply>

</apply>

<apply>

<times/>

<ci> mw6e554aaa_4cb8_46a2_9af0_40c527042d95 </ci>

<ci> mw2ffe78db_96a4_4d1b_a62f_f84d7987e64e </ci>

</apply>

</apply>

<apply>

<minus/>

<apply>

<plus/>

<apply>

<times/>

<apply>

<plus/>

<cn type="integer"> 1 </cn>

<apply>

<divide/>

<ci> mw2a09f89f_5936_4376_a2ab_4ff1bbbd4153 </ci>

<ci> mwef61df7c_5b85_41cf_b539_58e829001c39 </ci>

</apply>

</apply>

<apply>

<plus/>

<cn type="integer"> 1 </cn>

<apply>

<divide/>

<ci> mwacf08e72_f001_4a26_962d_729f504b1ad0 </ci>
<ci> mw9d553771_b33e_40de_bbc5_22c5804e335a </ci>
</apply>
</apply>
</apply>
<apply>
<times/>
<apply>
<plus/>
<cn type="integer"> 1 </cn>
<apply>
<divide/>
<ci> mw8f67b710_8c1c_453f_a7ec_e9b6a55814fa </ci>
<ci> mw6e554aaa_4cb8_46a2_9af0_40c527042d95 </ci>
</apply>
</apply>
<apply>
<plus/>
<cn type="integer"> 1 </cn>
<apply>
<divide/>
<ci> mwaa8b93c8_308a_4d19_b732_22cb31f77620 </ci>
<ci> mw2ffe78db_96a4_4d1b_a62f_f84d7987e64e </ci>
</apply>
</apply>
<apply>
<plus/>
<cn type="integer"> 1 </cn>
<apply>
<divide/>
<ci> mw5b528d37_b514_46e5_a018_0173afdae714 </ci>

```
          <ci> mwb4c2b40c_c637_4023_8d0f_5ed2bc9febf1 </ci>
         </apply>
        </apply>
       </apply>
      </apply>
      <cn type="integer"> 1 </cn>
     </apply>
    </apply>
    <apply>
     <divide/>
     <apply>
      <divide/>
      <apply>
       <times/>
       <ci> mwb84f53a1_12bb_45c4_bc48_32fddf334c72 </ci>
       <apply>
        <minus/>
        <apply>
         <times/>
         <ci> mw8f67b710_8c1c_453f_a7ec_e9b6a55814fa </ci>
         <ci> mwaa8b93c8_308a_4d19_b732_22cb31f77620 </ci>
        </apply>
        <apply>
         <divide/>
         <ci> mwacf08e72_f001_4a26_962d_729f504b1ad0 </ci>
         <ci> mw9de2bbc0_b1db_4824_9105_762861f97d06 </ci>
        </apply>
       </apply>
      </apply>
      <apply>
       <times/>
```

```
    <ci> mwb15d9294_9509_4702_9083_ef51d8c235f9 </ci>
    <ci> mwc410441d_bd1d_4085_9e21_08b662e4f672 </ci>
  </apply>
</apply>
<apply>
  <minus/>
  <apply>
    <plus/>
    <apply>
      <times/>
      <apply>
        <plus/>
        <cn type="integer"> 1 </cn>
        <apply>
          <divide/>
          <ci> mw2a09f89f_5936_4376_a2ab_4ff1bbbd4153 </ci>
          <ci> mw1e28fdab_0576_4966_9927_17b091f2a4b2 </ci>
        </apply>
      </apply>
      <apply>
        <plus/>
        <cn type="integer"> 1 </cn>
        <apply>
          <divide/>
          <ci> mwacf08e72_f001_4a26_962d_729f504b1ad0 </ci>
          <ci> mw777c1f25_305f_42cf_a010_b537a05884dd </ci>
        </apply>
      </apply>
    </apply>
    <apply>
      <times/>
```

```
   <apply>
    <plus/>
    <cn type="integer"> 1 </cn>
    <apply>
     <divide/>
     <ci> mw8f67b710_8c1c_453f_a7ec_e9b6a55814fa </ci>
     <ci> mwb15d9294_9509_4702_9083_ef51d8c235f9 </ci>
    </apply>
   </apply>
   <apply>
    <plus/>
    <cn type="integer"> 1 </cn>
    <apply>
     <divide/>
     <ci> mwaa8b93c8_308a_4d19_b732_22cb31f77620 </ci>
     <ci> mwc410441d_bd1d_4085_9e21_08b662e4f672 </ci>
    </apply>
   </apply>
   <apply>
    <plus/>
    <cn type="integer"> 1 </cn>
    <apply>
     <divide/>
     <ci> mw5b528d37_b514_46e5_a018_0173afdae714 </ci>
     <ci> mw8b39e257_03b8_4927_97c6_4b8444f70966 </ci>
    </apply>
   </apply>
  </apply>
 </apply>
 <cn type="integer"> 1 </cn>
</apply>
```

```
      </apply>
     </apply>
    </math>
   </assignmentRule>
   <assignmentRule metaid="repeatedAssignment_mw4598e041_4a8b_472f_8193_2a60c9b42201" variable="mw816c234f_a368_4d43_a45f_5a7d12510976">
    <math xmlns="http://www.w3.org/1998/Math/MathML">
     <apply>
      <divide/>
      <apply>
       <times/>
       <ci> mw676816c3_3653_49ac_9693_a838ec5f9446 </ci>
       <ci> mwacf08e72_f001_4a26_962d_729f504b1ad0 </ci>
      </apply>
      <apply>
       <plus/>
       <ci> mwacf08e72_f001_4a26_962d_729f504b1ad0 </ci>
       <apply>
        <times/>
        <ci> mw5fc73772_4633_4bf8_9aa6_fbabe153e1c8 </ci>
        <apply>
         <plus/>
         <cn type="integer"> 1 </cn>
         <apply>
          <divide/>
          <ci> mw2a09f89f_5936_4376_a2ab_4ff1bbbd4153 </ci>
          <ci> mwac1e88d0_dca5_49b3_9950_4e0560efa626 </ci>
         </apply>
        </apply>
       </apply>
      </apply>
```

```
      </apply>
     </math>
    </assignmentRule>
    <assignmentRule metaid="repeatedAssignment_mwf21a3104_1a8f_4068_950c_6e515f0b09f7"
variable="mw9944c18e_1f0c_4a7a_abdb_b1b56d72a673">
     <math xmlns="http://www.w3.org/1998/Math/MathML">
      <apply>
       <divide/>
       <apply>
        <divide/>
        <apply>
         <times/>
         <ci> mw509e62ff_43c2_439f_a219_323f6f557755 </ci>
         <apply>
          <minus/>
          <apply>
           <times/>
           <ci> mw5b528d37_b514_46e5_a018_0173afdae714 </ci>
           <ci> mweadaac0b_cbd7_4eb3_b892_fe6bf884cfc8 </ci>
          </apply>
          <apply>
           <divide/>
           <apply>
            <times/>
            <ci> mw26816e75_d092_459e_bb3d_70aafa096e4d </ci>
            <ci> mw7c70a014_6b69_42b3_9fbd_1d63cb722ae5 </ci>
           </apply>
           <ci> mw9d6196a1_62e0_4989_84af_dba7005fa377 </ci>
          </apply>
         </apply>
        </apply>
```

```
  <apply>
   <times/>
   <ci> mw57be342f_c610_40fc_8742_cd5cac73cf4a </ci>
   <ci> mwd82e6763_7382_481e_82d1_193b24b364f8 </ci>
  </apply>
 </apply>
 <apply>
  <times/>
  <apply>
   <plus/>
   <cn type="integer"> 1 </cn>
   <apply>
    <divide/>
    <ci> mwfcedd013_9e1c_4e21_9a5a_93ddde2ffc11 </ci>
    <ci> mw7e01e1f4_5a8e_40f5_805c_6bb66e4302d5 </ci>
   </apply>
   <apply>
    <divide/>
    <ci> mwaa8b93c8_308a_4d19_b732_22cb31f77620 </ci>
    <ci> mw1940c877_4e88_4a71_957a_5160dc7cfd3c </ci>
   </apply>
  </apply>
  <apply>
   <plus/>
   <cn type="integer"> 1 </cn>
   <apply>
    <divide/>
    <ci> mw5b528d37_b514_46e5_a018_0173afdae714 </ci>
    <ci> mwd82e6763_7382_481e_82d1_193b24b364f8 </ci>
   </apply>
   <apply>
```

```
          <divide/>
          <ci> mw7c70a014_6b69_42b3_9fbd_1d63cb722ae5 </ci>
          <ci> mw328820c4_827f_4b41_ace1_19213258ac79 </ci>
        </apply>
      </apply>
      <apply>
        <plus/>
        <cn type="integer"> 1 </cn>
        <apply>
          <divide/>
          <ci> mweadaac0b_cbd7_4eb3_b892_fe6bf884cfc8 </ci>
          <ci> mw57be342f_c610_40fc_8742_cd5cac73cf4a </ci>
        </apply>
        <apply>
          <divide/>
          <ci> mw26816e75_d092_459e_bb3d_70aafa096e4d </ci>
          <ci> mw545ed108_3a36_4f08_880d_e713dae6443a </ci>
        </apply>
      </apply>
    </apply>
  </apply>
</math>
</assignmentRule>
<assignmentRule metaid="repeatedAssignment_mw8bb04128_4442_4363_abf7_795cfe126b52"
variable="mwd022fa3d_5c26_490a_b4a4_51ddcf64258c">
<math xmlns="http://www.w3.org/1998/Math/MathML">
  <apply>
    <divide/>
    <apply>
      <divide/>
      <apply>
```

```
          <times/>
          <ci> mwf878e33d_3228_40a3_8fa6_9865294de7a1 </ci>
          <apply>
            <minus/>
            <ci> mw26816e75_d092_459e_bb3d_70aafa096e4d </ci>
            <apply>
              <divide/>
              <ci> mw1d7eb0d8_a048_4013_9bbd_c347637cc6b9 </ci>
              <ci> mwa92c6c71_d522_4d07_9ef1_f6f2c17f9c71 </ci>
            </apply>
          </apply>
        </apply>
        <ci> mwc31a14d7_9c22_4389_99c6_58d27312cdb7 </ci>
      </apply>
      <apply>
        <plus/>
        <cn type="integer"> 1 </cn>
        <apply>
          <divide/>
          <ci> mw1d7eb0d8_a048_4013_9bbd_c347637cc6b9 </ci>
          <ci> mwd908059f_c845_4440_addd_e491a150ff80 </ci>
        </apply>
        <apply>
          <divide/>
          <ci> mw26816e75_d092_459e_bb3d_70aafa096e4d </ci>
          <ci> mwc31a14d7_9c22_4389_99c6_58d27312cdb7 </ci>
        </apply>
      </apply>
    </apply>
  </math>
</assignmentRule>
```

<assignmentRule metaid="repeatedAssignment_mw712cfe94_df92_4f4f_bde4_f89fb9e10b83" variable="mw19a93cbf_18c6_4427_8e26_1fa9e7dad171">
<math xmlns="http://www.w3.org/1998/Math/MathML">
<apply>
<divide/>
<apply>
<divide/>
<apply>
<times/>
<ci> mw8f309108_f60b_445c_af16_065b0479e746 </ci>
<apply>
<minus/>
<ci> mw7c70a014_6b69_42b3_9fbd_1d63cb722ae5 </ci>
<apply>
<divide/>
<ci> mw1d7eb0d8_a048_4013_9bbd_c347637cc6b9 </ci>
<ci> mw93964b11_67c1_4727_84cc_949cd4f6ecf4 </ci>
</apply>
</apply>
</apply>
<ci> mw9a22bae8_8f9c_4a94_a7ab_127fa8a4d3bd </ci>
</apply>
<apply>
<plus/>
<cn type="integer"> 1 </cn>
<apply>
<divide/>
<ci> mw1d7eb0d8_a048_4013_9bbd_c347637cc6b9 </ci>
<ci> mw1c952255_2606_4fe2_9e07_47ea2266367e </ci>
</apply>
<apply>

<divide/>

<ci> mw7c70a014_6b69_42b3_9fbd_1d63cb722ae5 </ci>

<ci> mw9a22bae8_8f9c_4a94_a7ab_127fa8a4d3bd </ci>

</apply>

</apply>

</apply>

</math>

</assignmentRule>

<assignmentRule metaid="repeatedAssignment_mwb5f71d9a_64b6_422e_9be7_a984c3eb9eb8" variable="mw058a38dd_174d_4e6e_a7d9_26b31cc47d39">

<math xmlns="http://www.w3.org/1998/Math/MathML">

<apply>

<divide/>

<apply>

<times/>

<ci> mw6ab10457_9720_4445_a710_232dd2c4547d </ci>

<ci> mw8e2cc29c_c73e_4258_b3ba_2356620bb64e </ci>

<ci> mw1d7eb0d8_a048_4013_9bbd_c347637cc6b9 </ci>

</apply>

<apply>

<times/>

<apply>

<plus/>

<apply>

<times/>

<ci> mw8e2cc29c_c73e_4258_b3ba_2356620bb64e </ci>

<apply>

<plus/>

<cn type="integer"> 1 </cn>

<apply>

<divide/>

```
      <ci> mwe8d1ae1f_35f9_45fd_bedf_7dbeef3972e2 </ci>
      <ci> mw3ca15696_2c16_4bbe_9acc_5bb19882a03e </ci>
     </apply>
    </apply>
   </apply>
   <apply>
    <times/>
    <ci> mwf6529569_4b56_44fa_993e_f2741a368c08 </ci>
    <apply>
     <plus/>
     <cn type="integer"> 1 </cn>
     <apply>
      <divide/>
      <ci> mwe8d1ae1f_35f9_45fd_bedf_7dbeef3972e2 </ci>
      <ci> mw459ad12f_127a_4f15_a4ca_dd8d9d3ca505 </ci>
     </apply>
    </apply>
   </apply>
  </apply>
  <apply>
   <plus/>
   <ci> mw1d7eb0d8_a048_4013_9bbd_c347637cc6b9 </ci>
   <apply>
    <times/>
    <ci> mwa97791f8_5c29_40fd_8db4_b61695f0810e </ci>
    <apply>
     <plus/>
     <cn type="integer"> 1 </cn>
     <apply>
      <divide/>
      <ci> mw372b8247_b11e_4d9b_be9b_e742fd2d3853 </ci>
```

<ci> mw620d20e1_7007_4d40_9ee4_67bc35938f33 </ci>
</apply>
<apply>
<divide/>
<ci> mw98fecd51_ba08_4727_98ec_04637a47c5ac </ci>
<ci> mwa3e0f729_e97d_443e_a14a_9eb9891474e1 </ci>
</apply>
<apply>
<divide/>
<ci> mw854ce178_2796_4118_8690_c13951a897c1 </ci>
<ci> mw5011fe71_c328_4107_9245_0861c6dbfb16 </ci>
</apply>
</apply>
</apply>
</apply>
</apply>
</apply>
</math>
</assignmentRule>
<assignmentRule metaid="repeatedAssignment_mwe6aa35ed_3563_44fb_b090_a9e35076253f" variable="mwfd2ced55_7a6c_4e46_89e8_6e09c111839b">
<math xmlns="http://www.w3.org/1998/Math/MathML">
<apply>
<plus/>
<apply>
<divide/>
<apply>
<divide/>
<apply>
<times/>
<ci> mw47ef96f3_f55b_4c2f_aeb6_7b00be2f7c33 </ci>

```
      <ci> mwfcedd013_9e1c_4e21_9a5a_93ddde2ffc11 </ci>
      <ci> mw8e2cc29c_c73e_4258_b3ba_2356620bb64e </ci>
    </apply>
    <apply>
      <times/>
      <ci> mwfab2bb7b_fa67_47d4_b71e_f36e49d4c2f7 </ci>
      <ci> mwc92f9b3a_36c7_4c3f_a179_2eb02421e208 </ci>
    </apply>
  </apply>
  <apply>
    <plus/>
    <cn type="integer"> 1 </cn>
    <apply>
      <times/>
      <apply>
        <divide/>
        <ci> mw8e2cc29c_c73e_4258_b3ba_2356620bb64e </ci>
        <ci> mwc92f9b3a_36c7_4c3f_a179_2eb02421e208 </ci>
      </apply>
      <apply>
        <divide/>
        <ci> mwfcedd013_9e1c_4e21_9a5a_93ddde2ffc11 </ci>
        <ci> mwfab2bb7b_fa67_47d4_b71e_f36e49d4c2f7 </ci>
      </apply>
    </apply>
  </apply>
</apply>
<apply>
  <divide/>
  <apply>
    <divide/>
```

```
    <apply>
     <times/>
     <ci> mwe08f3352_d986_4a9a_acea_33701d19a3b3 </ci>
     <ci> mwfcedd013_9e1c_4e21_9a5a_93ddde2ffc11 </ci>
     <ci> mw8e2cc29c_c73e_4258_b3ba_2356620bb64e </ci>
    </apply>
    <apply>
     <times/>
     <ci> mwd2e258fa_4740_4b3b_816e_0b0e55bdb005 </ci>
     <ci> mw9bc87645_2d3f_4a39_8dff_d0074e096984 </ci>
    </apply>
   </apply>
   <apply>
    <plus/>
    <cn type="integer"> 1 </cn>
    <apply>
     <times/>
     <apply>
      <divide/>
      <ci> mw8e2cc29c_c73e_4258_b3ba_2356620bb64e </ci>
      <ci> mw9bc87645_2d3f_4a39_8dff_d0074e096984 </ci>
     </apply>
     <apply>
      <divide/>
      <ci> mwfcedd013_9e1c_4e21_9a5a_93ddde2ffc11 </ci>
      <ci> mwd2e258fa_4740_4b3b_816e_0b0e55bdb005 </ci>
     </apply>
    </apply>
   </apply>
  </apply>
</apply>
```

```
</math>
</assignmentRule>
<assignmentRule metaid="repeatedAssignment_mw021d0e35_8298_4e2e_87fa_ac2c4ad5cd4b" variable="mwd8b2ff5c_3c77_43ff_8fad_9c93171bb747">
<math xmlns="http://www.w3.org/1998/Math/MathML">
<apply>
<plus/>
<apply>
<divide/>
<apply>
<times/>
<ci> mwa577376f_a6e1_47eb_bf4d_2b6dc53f1249 </ci>
<apply>
<minus/>
<ci> mw372b8247_b11e_4d9b_be9b_e742fd2d3853 </ci>
<apply>
<divide/>
<ci> mwc25bd841_1335_4e94_9ffa_621b205bc0da </ci>
<ci> mw3f9f4337_1baa_4946_8e90_d13ca5c3adbf </ci>
</apply>
</apply>
</apply>
<apply>
<times/>
<ci> mw7d7f5782_23e4_4797_8dc7_355ec98f4d6a </ci>
<apply>
<plus/>
<cn type="integer"> 1 </cn>
<apply>
<divide/>
<ci> mw372b8247_b11e_4d9b_be9b_e742fd2d3853 </ci>
```

<ci> mw7d7f5782_23e4_4797_8dc7_355ec98f4d6a </ci>
</apply>
<apply>
<divide/>
<ci> mwc25bd841_1335_4e94_9ffa_621b205bc0da </ci>
<ci> mw28d0b788_21a0_4f17_ac4c_9b32d44ae058 </ci>
</apply>
</apply>
</apply>
</apply>
<apply>
<divide/>
<apply>
<times/>
<ci> mw4674ad99_0bbe_4aef_99fa_49937a6f49d7 </ci>
<apply>
<minus/>
<ci> mw372b8247_b11e_4d9b_be9b_e742fd2d3853 </ci>
<apply>
<divide/>
<ci> mwc25bd841_1335_4e94_9ffa_621b205bc0da </ci>
<ci> mw3f9f4337_1baa_4946_8e90_d13ca5c3adbf </ci>
</apply>
</apply>
</apply>
<apply>
<times/>
<ci> mwc0011b36_3da6_4d34_9157_28ac5f77a5df </ci>
<apply>
<plus/>
<cn type="integer"> 1 </cn>

<apply>
<divide/>
<ci> mw372b8247_b11e_4d9b_be9b_e742fd2d3853 </ci>
<ci> mwc0011b36_3da6_4d34_9157_28ac5f77a5df </ci>
</apply>
<apply>
<divide/>
<ci> mwc25bd841_1335_4e94_9ffa_621b205bc0da </ci>
<ci> mw8a2c7a1e_c472_4f06_a4c7_38688feecabc </ci>
</apply>
</apply>
</apply>
</apply>
<apply>
<divide/>
<apply>
<times/>
<ci> mw11591b16_f15e_41a6_890c_7da51ea27d51 </ci>
<apply>
<minus/>
<ci> mw372b8247_b11e_4d9b_be9b_e742fd2d3853 </ci>
<apply>
<divide/>
<ci> mwc25bd841_1335_4e94_9ffa_621b205bc0da </ci>
<ci> mw3f9f4337_1baa_4946_8e90_d13ca5c3adbf </ci>
</apply>
</apply>
</apply>
<apply>
<times/>
<ci> mw56023d39_08c8_49e5_a347_a28c871b8290 </ci>

```
            <apply>
              <plus/>
              <cn type="integer"> 1 </cn>
              <apply>
                <divide/>
                <ci> mw372b8247_b11e_4d9b_be9b_e742fd2d3853 </ci>
                <ci> mw56023d39_08c8_49e5_a347_a28c871b8290 </ci>
              </apply>
              <apply>
                <divide/>
                <ci> mwc25bd841_1335_4e94_9ffa_621b205bc0da </ci>
                <ci> mwc5650e27_4e7c_4064_8600_9ff3fd5f22ca </ci>
              </apply>
            </apply>
          </apply>
        </apply>
      </apply>
    </math>
  </assignmentRule>
  <assignmentRule metaid="repeatedAssignment_mw21c8b223_0a70_4532_992e_5a14921f8148"
variable="mw8b51e2ce_42bd_422e_96b0_79b39239a4a9">
    <math xmlns="http://www.w3.org/1998/Math/MathML">
      <apply>
        <divide/>
        <apply>
          <times/>
          <ci> mw20c2fea9_d469_4e35_bdf8_38cbe9f5bd92 </ci>
          <apply>
            <minus/>
            <ci> mwc25bd841_1335_4e94_9ffa_621b205bc0da </ci>
            <apply>
```

<divide/>
<ci> mw6951dd88_19cf_4045_95c4_0e8e2bf976c1 </ci>
<ci> mw963788fc_9773_4082_8fe4_0f8ea1e81867 </ci>
</apply>
</apply>
</apply>
<apply>
<times/>
<ci> mw8e5540c2_ecd1_4e3e_bb5a_651353fa0ec2 </ci>
<apply>
<plus/>
<cn type="integer"> 1 </cn>
<apply>
<divide/>
<ci> mwc25bd841_1335_4e94_9ffa_621b205bc0da </ci>
<ci> mw8e5540c2_ecd1_4e3e_bb5a_651353fa0ec2 </ci>
</apply>
<apply>
<divide/>
<ci> mw6951dd88_19cf_4045_95c4_0e8e2bf976c1 </ci>
<ci> mwb8a92b6d_ce94_48d0_8b18_58660e599368 </ci>
</apply>
</apply>
</apply>
</apply>
</math>
</assignmentRule>
<assignmentRule metaid="repeatedAssignment_mw09791861_4454_4b29_ab4c_29181a3c7fa6" variable="mw7129d4a5_ea33_4780_a37b_1cc553e6cbe6">
<math xmlns="http://www.w3.org/1998/Math/MathML">
<apply>

<divide/>
<apply>
<times/>
<ci> mw38ba402b_328c_454f_807b_2a5d33b8d48f </ci>
<ci> mw5b528d37_b514_46e5_a018_0173afdae714 </ci>
<ci> mw7c834bb1_1865_4950_aa20_f3eceb92b21d </ci>
</apply>
<apply>
<times/>
<apply>
<plus/>
<cn type="integer"> 1 </cn>
<apply>
<divide/>
<ci> mw5b528d37_b514_46e5_a018_0173afdae714 </ci>
<ci> mw4da83b47_6347_43ed_b1b6_c95ab9075f9d </ci>
</apply>
<apply>
<divide/>
<ci> mw372b8247_b11e_4d9b_be9b_e742fd2d3853 </ci>
<ci> mw02a594bf_b380_46b9_acac_e7344c88da82 </ci>
</apply>
</apply>
<apply>
<plus/>
<cn type="integer"> 1 </cn>
<apply>
<divide/>
<ci> mw7c834bb1_1865_4950_aa20_f3eceb92b21d </ci>
<ci> mw640c5b04_67dd_43d7_8b11_069cf242288b </ci>
</apply>

<apply>
<divide/>
<ci> mwd2e6e004_23f5_4cd8_81e0_8385021ec904 </ci>
<ci> mwebd2cdb3_d654_4e7e_a036_7180e1269833 </ci>
</apply>
</apply>
</apply>
</apply>
</math>
</assignmentRule>
<assignmentRule metaid="repeatedAssignment_mw1e74a2f6_9bf5_4c13_a541_8b2462f7d686" variable="mwca20acee_79f0_416c_9f8d_d598757960ee">
<math xmlns="http://www.w3.org/1998/Math/MathML">
<apply>
<divide/>
<apply>
<times/>
<ci> mwbba6a282_e627_49c3_9f45_889c3993c575 </ci>
<ci> mw6951dd88_19cf_4045_95c4_0e8e2bf976c1 </ci>
</apply>
<apply>
<plus/>
<ci> mw10c7c233_9065_48c8_8a5c_8a27f4ee15d6 </ci>
<ci> mw6951dd88_19cf_4045_95c4_0e8e2bf976c1 </ci>
</apply>
</apply>
</math>
</assignmentRule>
<assignmentRule metaid="repeatedAssignment_mw50e593cc_e9d8_433d_9553_ff83cb187a91" variable="mwd279fef9_0d64_43b6_811b_d126265b2623">
<math xmlns="http://www.w3.org/1998/Math/MathML">
<apply>

```
        <divide/>
        <apply>
          <times/>
          <ci> mw6b34d5e4_8388_4d61_837c_26f3c9c09838 </ci>
          <ci> mw3a6830cd_1b04_49df_b4fe_fc7a6362142a </ci>
        </apply>
        <apply>
          <plus/>
          <ci> mwd7d873d8_453a_431d_a268_fff0b2981096 </ci>
          <ci> mw3a6830cd_1b04_49df_b4fe_fc7a6362142a </ci>
        </apply>
      </apply>
    </math>
  </assignmentRule>
  <assignmentRule metaid="repeatedAssignment_mw16cd0155_6990_49b8_b223_6de8e146c3ca" variable="mw606af7f8_db79_45f3_896c_81838de43916">
    <math xmlns="http://www.w3.org/1998/Math/MathML">
      <apply>
        <divide/>
        <apply>
          <times/>
          <ci> mwf7816b82_0693_49ca_9589_4c1ec5f35110 </ci>
          <ci> mw5b528d37_b514_46e5_a018_0173afdae714 </ci>
        </apply>
        <apply>
          <plus/>
          <ci> mw9c17270d_1b98_4c92_8c4f_0bf91f94ac5c </ci>
          <ci> mw5b528d37_b514_46e5_a018_0173afdae714 </ci>
        </apply>
      </apply>
    </math>
```

```
      </assignmentRule>
      <assignmentRule metaid="repeatedAssignment_mw79656c4d_a362_45aa_b521_307999b012c4" variable="mw390bea38_1b39_4962_8f10_962e6ca96e3d">
        <math xmlns="http://www.w3.org/1998/Math/MathML">
          <apply>
            <divide/>
            <apply>
              <times/>
              <ci> mwd116d1da_a4e2_47b1_b71c_643640c7faac </ci>
              <ci> mwaa8b93c8_308a_4d19_b732_22cb31f77620 </ci>
            </apply>
            <apply>
              <plus/>
              <ci> mw03716e78_6785_43c8_9249_0ab554cf9111 </ci>
              <ci> mwaa8b93c8_308a_4d19_b732_22cb31f77620 </ci>
            </apply>
          </apply>
        </math>
      </assignmentRule>
      <assignmentRule metaid="repeatedAssignment_mw78058ad3_0bce_4978_86b5_a6eb34c5373b" variable="mwf6dc3a41_68d1_481a_a511_3aadf99e49b0">
        <math xmlns="http://www.w3.org/1998/Math/MathML">
          <apply>
            <divide/>
            <apply>
              <times/>
              <ci> mw089625ae_78a0_4e60_953d_7bccf06a6d3f </ci>
              <ci> mw26816e75_d092_459e_bb3d_70aafa096e4d </ci>
            </apply>
            <apply>
              <plus/>
              <ci> mw4782b972_ca60_4b9e_96dd_9dba1be35a3e </ci>
```

```
      &lt;ci&gt; mw26816e75_d092_459e_bb3d_70aafa096e4d &lt;/ci&gt;
     &lt;/apply&gt;
    &lt;/apply&gt;
   &lt;/math&gt;
  &lt;/assignmentRule&gt;
  <assignmentRule metaid="repeatedAssignment_mw927f4106_e784_4955_a825_1c2a55051829"
variable="mw8425df07_de4f_4a79_9f4a_0a9334146b90">
   &lt;math xmlns="http://www.w3.org/1998/Math/MathML"&gt;
    &lt;apply&gt;
     &lt;plus/&gt;
     &lt;apply&gt;
      &lt;divide/&gt;
      &lt;apply&gt;
       &lt;divide/&gt;
       &lt;apply&gt;
        &lt;times/&gt;
        &lt;ci&gt; mw6e7fabe6_de80_41fa_9146_5e9b7ec4bc63 &lt;/ci&gt;
        &lt;apply&gt;
         &lt;minus/&gt;
         &lt;ci&gt; mwfcedd013_9e1c_4e21_9a5a_93ddde2ffc11 &lt;/ci&gt;
         &lt;apply&gt;
          &lt;divide/&gt;
          &lt;apply&gt;
           &lt;times/&gt;
           &lt;ci&gt; mw543ef166_8322_44f0_9435_270a9f9672da &lt;/ci&gt;
           &lt;ci&gt; mwaa8b93c8_308a_4d19_b732_22cb31f77620 &lt;/ci&gt;
          &lt;/apply&gt;
          &lt;ci&gt; mw066182b3_2182_44b1_b745_125e1968e0bc &lt;/ci&gt;
         &lt;/apply&gt;
        &lt;/apply&gt;
       &lt;/apply&gt;
```

<ci> mw8e6b4f11_baec_4a0f_983d_3d1d46cb91cb </ci>
</apply>
<apply>
<times/>
<apply>
<plus/>
<cn type="integer"> 1 </cn>
<apply>
<divide/>
<ci> mwfcedd013_9e1c_4e21_9a5a_93ddde2ffc11 </ci>
<ci> mw8e6b4f11_baec_4a0f_983d_3d1d46cb91cb </ci>
</apply>
<apply>
<divide/>
<ci> mwaa8b93c8_308a_4d19_b732_22cb31f77620 </ci>
<ci> mw8b16f07a_00d8_489e_ae07_2dc92af4dfb9 </ci>
</apply>
</apply>
<apply>
<plus/>
<cn type="integer"> 1 </cn>
<apply>
<divide/>
<ci> mw543ef166_8322_44f0_9435_270a9f9672da </ci>
<ci> mw514d5907_3da1_4e17_8024_f637af4b38fa </ci>
</apply>
</apply>
<apply>
<plus/>
<cn type="integer"> 1 </cn>
<apply>

<divide/>
<ci> mw7c70a014_6b69_42b3_9fbd_1d63cb722ae5 </ci>
<ci> mw562c606a_6226_47bc_8fe9_db84aa73a146 </ci>
</apply>
<apply>
<divide/>
<ci> mw5b528d37_b514_46e5_a018_0173afdae714 </ci>
<ci> mw9d34e88a_6503_48b9_a787_58c326a932a5 </ci>
</apply>
</apply>
</apply>
</apply>
<apply>
<divide/>
<apply>
<divide/>
<apply>
<times/>
<ci> mw0ab3c1a9_2d85_4242_b2c8_8668592bc4b4 </ci>
<apply>
<minus/>
<ci> mw7c70a014_6b69_42b3_9fbd_1d63cb722ae5 </ci>
<apply>
<divide/>
<apply>
<times/>
<ci> mw543ef166_8322_44f0_9435_270a9f9672da </ci>
<ci> mw5b528d37_b514_46e5_a018_0173afdae714 </ci>
</apply>
<ci> mw44c8ec20_4a1e_454f_a1a0_42d862b117ae </ci>
</apply>

</apply>
</apply>
<ci> mw057e7327_64bf_4b85_bb90_78da6ba5980f </ci>
</apply>
<apply>
<times/>
<apply>
<plus/>
<cn type="integer"> 1 </cn>
<apply>
<divide/>
<ci> mwfcedd013_9e1c_4e21_9a5a_93ddde2ffc11 </ci>
<ci> mw77007da8_e432_48eb_9c52_ed3c2c1b5f8c </ci>
</apply>
<apply>
<divide/>
<ci> mwaa8b93c8_308a_4d19_b732_22cb31f77620 </ci>
<ci> mw9580aa5c_a5df_472e_aad4_d7d8dfaf50c5 </ci>
</apply>
</apply>
<apply>
<plus/>
<cn type="integer"> 1 </cn>
<apply>
<divide/>
<ci> mw543ef166_8322_44f0_9435_270a9f9672da </ci>
<ci> mw6dff67cf_5c46_4621_9902_c290a3f63d5d </ci>
</apply>
</apply>
<apply>
<plus/>

<cn type="integer"> 1 </cn>
<apply>
<divide/>
<ci> mw7c70a014_6b69_42b3_9fbd_1d63cb722ae5 </ci>
<ci> mw057e7327_64bf_4b85_bb90_78da6ba5980f </ci>
</apply>
<apply>
<divide/>
<ci> mw5b528d37_b514_46e5_a018_0173afdae714 </ci>
<ci> mwa0585ea2_d52f_4310_adf2_e8de58065ae9 </ci>
</apply>
</apply>
</apply>
</apply>
</apply>
</math>
</assignmentRule>
<assignmentRule metaid="repeatedAssignment_mw16935201_88ab_40a9_b992_4f41c810e6e8" variable="mw606dccc8_184f_4101_85c0_fff997c104ca">
<math xmlns="http://www.w3.org/1998/Math/MathML">
<apply>
<plus/>
<apply>
<divide/>
<apply>
<divide/>
<apply>
<times/>
<ci> mw9e5312fb_9ca1_4ae7_8fef_e40c70633af3 </ci>
<apply>
<minus/>

```
      <ci> mwfcedd013_9e1c_4e21_9a5a_93ddde2ffc11 </ci>
      <apply>
        <divide/>
        <apply>
          <times/>
          <ci> mw543ef166_8322_44f0_9435_270a9f9672da </ci>
          <ci> mwaa8b93c8_308a_4d19_b732_22cb31f77620 </ci>
        </apply>
        <ci> mw066182b3_2182_44b1_b745_125e1968e0bc </ci>
      </apply>
    </apply>
  </apply>
  <ci> mw7c84a538_b915_4a91_8cc2_04c534c01ab8 </ci>
</apply>
<apply>
  <times/>
  <apply>
    <plus/>
    <cn type="integer"> 1 </cn>
    <apply>
      <divide/>
      <ci> mwfcedd013_9e1c_4e21_9a5a_93ddde2ffc11 </ci>
      <ci> mw7c84a538_b915_4a91_8cc2_04c534c01ab8 </ci>
    </apply>
    <apply>
      <divide/>
      <ci> mwaa8b93c8_308a_4d19_b732_22cb31f77620 </ci>
      <ci> mw4d62dae0_b6d6_45ba_8fb6_caf10d3295dc </ci>
    </apply>
  </apply>
  <apply>
```

```
      <plus/>
      <cn type="integer"> 1 </cn>
      <apply>
        <divide/>
        <ci> mw543ef166_8322_44f0_9435_270a9f9672da </ci>
        <ci> mw9f4be612_37b6_425f_863f_c3234aaafd9c </ci>
      </apply>
    </apply>
    <apply>
      <plus/>
      <cn type="integer"> 1 </cn>
      <apply>
        <divide/>
        <ci> mw7c70a014_6b69_42b3_9fbd_1d63cb722ae5 </ci>
        <ci> mw457a9b1f_ac25_45b4_adb7_c6c807be3474 </ci>
      </apply>
      <apply>
        <divide/>
        <ci> mw5b528d37_b514_46e5_a018_0173afdae714 </ci>
        <ci> mwa723f027_cc0e_426d_b99e_dd1f00ba7a3e </ci>
      </apply>
    </apply>
  </apply>
</apply>
<apply>
  <divide/>
  <apply>
    <divide/>
    <apply>
      <times/>
      <ci> mw5a9817b0_1b0d_4667_a467_59f6072696c8 </ci>
```

```
    <apply>
      <minus/>
      <ci> mw7c70a014_6b69_42b3_9fbd_1d63cb722ae5 </ci>
      <apply>
        <divide/>
        <apply>
          <times/>
          <ci> mw543ef166_8322_44f0_9435_270a9f9672da </ci>
          <ci> mw5b528d37_b514_46e5_a018_0173afdae714 </ci>
        </apply>
        <ci> mw44c8ec20_4a1e_454f_a1a0_42d862b117ae </ci>
      </apply>
    </apply>
  </apply>
  <ci> mwff222c2b_bc40_4d59_bfcd_63aeb04c86bd </ci>
</apply>
<apply>
  <times/>
  <apply>
    <plus/>
    <cn type="integer"> 1 </cn>
    <apply>
      <divide/>
      <ci> mwfcedd013_9e1c_4e21_9a5a_93ddde2ffc11 </ci>
      <ci> mw08f2e5c2_6905_4c90_8f37_016935f5d8ec </ci>
    </apply>
    <apply>
      <divide/>
      <ci> mwaa8b93c8_308a_4d19_b732_22cb31f77620 </ci>
      <ci> mw4c5adb36_23ae_4318_8b5f_6f80df2be00a </ci>
    </apply>
```

</apply>
<apply>
<plus/>
<cn type="integer"> 1 </cn>
<apply>
<divide/>
<ci> mw543ef166_8322_44f0_9435_270a9f9672da </ci>
<ci> mwc6a7cd20_1464_4521_88e6_145752ed585d </ci>
</apply>
</apply>
<apply>
<plus/>
<cn type="integer"> 1 </cn>
<apply>
<divide/>
<ci> mw7c70a014_6b69_42b3_9fbd_1d63cb722ae5 </ci>
<ci> mwff222c2b_bc40_4d59_bfcd_63aeb04c86bd </ci>
</apply>
<apply>
<divide/>
<ci> mw5b528d37_b514_46e5_a018_0173afdae714 </ci>
<ci> mw8d162e7c_404d_4423_8f50_9c55f37e0d2c </ci>
</apply>
</apply>
</apply>
</apply>
</apply>
</math>
</assignmentRule>
<assignmentRule metaid="repeatedAssignment_mw30df46b3_b460_4f31_991f_2706ef1e5ecf" variable="mw98b97e83_73fb_4ec8_8618_438ef4ddc02c">

```
<math xmlns="http://www.w3.org/1998/Math/MathML">
  <apply>
    <divide/>
    <apply>
      <times/>
      <ci> mw14fbc3da_1eb6_4470_8032_06f6d5ce7c93 </ci>
      <ci> mw3a6830cd_1b04_49df_b4fe_fc7a6362142a </ci>
    </apply>
    <apply>
      <times/>
      <ci> mwb23b6c01_848a_44fb_8324_dda82ffcb0c4 </ci>
      <apply>
        <plus/>
        <cn type="integer"> 1 </cn>
        <apply>
          <divide/>
          <ci> mw3a6830cd_1b04_49df_b4fe_fc7a6362142a </ci>
          <ci> mwb23b6c01_848a_44fb_8324_dda82ffcb0c4 </ci>
        </apply>
        <apply>
          <divide/>
          <ci> mwb8027448_5c51_43ad_9f13_cd37b5418a54 </ci>
          <ci> mwa4a1fe58_2c6a_4f99_8001_4a0fedc9a357 </ci>
        </apply>
      </apply>
    </apply>
  </apply>
</math>
</assignmentRule>
<assignmentRule
metaid="repeatedAssignment_mwd2375a79_d90a_400b_ad83_bb1bc9657092"
variable="mw29f84bc6_4f93_4a0d_b5ba_3692acbc98b4">
```

```
      <math xmlns="http://www.w3.org/1998/Math/MathML">
        <apply>
          <plus/>
          <ci> mwb7dd85d2_03b5_4642_acac_52cc771e3966 </ci>
          <ci> mwd9458308_bcb6_4caf_b7ac_c957a90c648b </ci>
          <ci> mwd6fab0c0_73f8_45ef_b32f_c7113be8258a </ci>
          <ci> mw24e36765_3502_4855_9948_95a6bb63a7a1 </ci>
          <ci> mw92f0b5a5_287d_4b4e_8e20_756a2c2a2fd7 </ci>
          <ci> mw11f497f7_cda2_4e49_a7e3_f2afaa053b5d </ci>
          <ci> mw99ae2c3c_4850_462a_8eb9_29ce642d9a9c </ci>
          <ci> mw5ef07c48_166e_4959_9987_d5fc6d58add4 </ci>
          <ci> mw6b76fe71_4295_4311_999a_67179a3d5a74 </ci>
        </apply>
      </math>
    </assignmentRule>
    <assignmentRule metaid="repeatedAssignment_mw2cba9b17_6e6a_4929_8e5b_a94905a1508e" variable="mw618971c6_5583_444a_880c_62858550103b">
      <math xmlns="http://www.w3.org/1998/Math/MathML">
        <apply>
          <divide/>
          <apply>
            <times/>
            <ci> mw02f9621e_b863_4168_a11d_fa2753817db7 </ci>
            <ci> mw372b8247_b11e_4d9b_be9b_e742fd2d3853 </ci>
          </apply>
          <apply>
            <plus/>
            <ci> mwdd6e9231_51b9_4b67_b71c_24c2e4af56ba </ci>
            <ci> mw372b8247_b11e_4d9b_be9b_e742fd2d3853 </ci>
          </apply>
        </apply>
```

</math>

</assignmentRule>

<assignmentRule metaid="repeatedAssignment_mw8df07b8f_6063_435d_9fd3_a17190cd0e1e" variable="mwb8f6ab34_7de8_40a2_be1f_6b28d461b072">

<math xmlns="http://www.w3.org/1998/Math/MathML">

<apply>

<minus/>

<ci> mw819bc32e_2626_4855_8895_5deebd61c820 </ci>

<ci> mwfd2ced55_7a6c_4e46_89e8_6e09c111839b </ci>

</apply>

</math>

</assignmentRule>

<assignmentRule metaid="repeatedAssignment_mw0fab8823_2881_412b_9920_399c84b067a4" variable="mw1dc47a27_16ad_4efd_8f31_09fac64ba939">

<math xmlns="http://www.w3.org/1998/Math/MathML">

<apply>

<divide/>

<apply>

<divide/>

<apply>

<times/>

<ci> mw518ffec0_9f71_433e_9dc8_9ca216769de1 </ci>

<ci> mwb8027448_5c51_43ad_9f13_cd37b5418a54 </ci>

</apply>

<apply>

<plus/>

<ci> mwb8027448_5c51_43ad_9f13_cd37b5418a54 </ci>

<ci> mw5ceb33ad_a5ce_4d67_8217_5ac758441102 </ci>

</apply>

</apply>

<apply>

<plus/>

```
            <cn type="integer"> 1 </cn>
            <apply>
              <divide/>
              <ci> mwf3e38952_b929_46f3_b15d_3e4a170b3863 </ci>
              <ci> mw7f5b93ed_2736_43c7_9104_bc8302ad8c01 </ci>
            </apply>
          </apply>
        </apply>
      </math>
    </assignmentRule>
    <assignmentRule metaid="repeatedAssignment_mwc9e2b7c1_5daa_4613_ae30_8bcc1a7dc15b"
variable="mw0103addc_790d_4201_8a92_819af4a321ef">
      <math xmlns="http://www.w3.org/1998/Math/MathML">
        <apply>
          <divide/>
          <apply>
            <times/>
            <ci> mwcb0e41ed_453b_4010_8628_3c3dc91f2591 </ci>
            <apply>
              <minus/>
              <apply>
                <times/>
                <ci> mwcff4a43c_2777_4532_a542_a418d54de341 </ci>
                <ci> mwfcedd013_9e1c_4e21_9a5a_93ddde2ffc11 </ci>
              </apply>
              <ci> mw3a6830cd_1b04_49df_b4fe_fc7a6362142a </ci>
            </apply>
          </apply>
          <apply>
            <plus/>
            <ci> mwfcedd013_9e1c_4e21_9a5a_93ddde2ffc11 </ci>
```

```
<ci> mw3a6830cd_1b04_49df_b4fe_fc7a6362142a </ci>
</apply>
</apply>
</math>
</assignmentRule>
<assignmentRule metaid="repeatedAssignment_mw57629939_f66d_499f_a95b_9e98260c849e" variable="mw2fe938ce_356d_4915_ac09_45c087e56003">
<math xmlns="http://www.w3.org/1998/Math/MathML">
<apply>
<times/>
<ci> mwbc1c0f08_cf5f_4e06_a14b_d5c559bb147c </ci>
<ci> mw19a9030f_8af8_485a_ba7c_5d89c93164cc </ci>
</apply>
</math>
</assignmentRule>
<assignmentRule metaid="repeatedAssignment_mw5dac9645_10a4_425f_a586_bb5f034f9ce4" variable="mwafafac3f_863a_498f_94e9_aa0a56a97576">
<math xmlns="http://www.w3.org/1998/Math/MathML">
<apply>
<divide/>
<apply>
<divide/>
<apply>
<times/>
<ci> mw47ef96f3_f55b_4c2f_aeb6_7b00be2f7c33 </ci>
<ci> mwfcedd013_9e1c_4e21_9a5a_93ddde2ffc11 </ci>
<ci> mw8e2cc29c_c73e_4258_b3ba_2356620bb64e </ci>
</apply>
<apply>
<times/>
<ci> mwfab2bb7b_fa67_47d4_b71e_f36e49d4c2f7 </ci>
<ci> mwc92f9b3a_36c7_4c3f_a179_2eb02421e208 </ci>
```

```
          </apply>
        </apply>
        <apply>
          <plus/>
          <cn type="integer"> 1 </cn>
          <apply>
            <times/>
            <apply>
              <divide/>
              <ci> mw8e2cc29c_c73e_4258_b3ba_2356620bb64e </ci>
              <ci> mwc92f9b3a_36c7_4c3f_a179_2eb02421e208 </ci>
            </apply>
            <apply>
              <divide/>
              <ci> mwfcedd013_9e1c_4e21_9a5a_93ddde2ffc11 </ci>
              <ci> mwfab2bb7b_fa67_47d4_b71e_f36e49d4c2f7 </ci>
            </apply>
          </apply>
        </apply>
      </apply>
    </math>
  </assignmentRule>
  <assignmentRule metaid="repeatedAssignment_mwd563acca_f74d_4e2c_bc34_37f384b7ff52" variable="mwafe48bf9_dc16_438d_a0de_9e4d98501709">
    <math xmlns="http://www.w3.org/1998/Math/MathML">
      <apply>
        <divide/>
        <apply>
          <divide/>
          <apply>
            <times/>
```

```
        <ci> mwe08f3352_d986_4a9a_acea_33701d19a3b3 </ci>
        <ci> mwfcedd013_9e1c_4e21_9a5a_93ddde2ffc11 </ci>
        <ci> mw8e2cc29c_c73e_4258_b3ba_2356620bb64e </ci>
      </apply>
      <apply>
        <times/>
        <ci> mwd2e258fa_4740_4b3b_816e_0b0e55bdb005 </ci>
        <ci> mw9bc87645_2d3f_4a39_8dff_d0074e096984 </ci>
      </apply>
    </apply>
    <apply>
      <plus/>
      <cn type="integer"> 1 </cn>
      <apply>
        <times/>
        <apply>
          <divide/>
          <ci> mw8e2cc29c_c73e_4258_b3ba_2356620bb64e </ci>
          <ci> mw9bc87645_2d3f_4a39_8dff_d0074e096984 </ci>
        </apply>
        <apply>
          <divide/>
          <ci> mwfcedd013_9e1c_4e21_9a5a_93ddde2ffc11 </ci>
          <ci> mwd2e258fa_4740_4b3b_816e_0b0e55bdb005 </ci>
        </apply>
      </apply>
    </apply>
  </apply>
</math>
</assignmentRule>
```

<assignmentRule metaid="repeatedAssignment_mw2bbd8047_56ad_44cd_a00a_d65dfe160d2e" variable="mw3d63e90e_4692_4dfd_a839_dd3307b0e55b">
<math xmlns="http://www.w3.org/1998/Math/MathML">
<apply>
<divide/>
<apply>
<divide/>
<apply>
<times/>
<ci> mw538c4cd4_f0aa_4fd6_8f8a_11b3e718bdca </ci>
<apply>
<minus/>
<apply>
<times/>
<ci> mw5b528d37_b514_46e5_a018_0173afdae714 </ci>
<ci> mw8f67b710_8c1c_453f_a7ec_e9b6a55814fa </ci>
</apply>
<apply>
<divide/>
<ci> mw2a09f89f_5936_4376_a2ab_4ff1bbbd4153 </ci>
<ci> mwa345d9e1_655b_41de_bcb1_d82b21b3c42c </ci>
</apply>
</apply>
</apply>
<apply>
<times/>
<ci> mw6e554aaa_4cb8_46a2_9af0_40c527042d95 </ci>
<ci> mwb4c2b40c_c637_4023_8d0f_5ed2bc9febf1 </ci>
</apply>
</apply>
<apply>

<minus/>
<apply>
<plus/>
<apply>
<times/>
<apply>
<plus/>
<cn type="integer"> 1 </cn>
<apply>
<divide/>
<ci> mw2a09f89f_5936_4376_a2ab_4ff1bbbd4153 </ci>
<ci> mwef61df7c_5b85_41cf_b539_58e829001c39 </ci>
</apply>
</apply>
<apply>
<plus/>
<cn type="integer"> 1 </cn>
<apply>
<divide/>
<ci> mwacf08e72_f001_4a26_962d_729f504b1ad0 </ci>
<ci> mw9d553771_b33e_40de_bbc5_22c5804e335a </ci>
</apply>
</apply>
</apply>
<apply>
<times/>
<apply>
<plus/>
<cn type="integer"> 1 </cn>
<apply>
<divide/>

```
              <ci> mw8f67b710_8c1c_453f_a7ec_e9b6a55814fa </ci>
              <ci> mw6e554aaa_4cb8_46a2_9af0_40c527042d95 </ci>
            </apply>
          </apply>
          <apply>
            <plus/>
            <cn type="integer"> 1 </cn>
            <apply>
              <divide/>
              <ci> mwaa8b93c8_308a_4d19_b732_22cb31f77620 </ci>
              <ci> mw2ffe78db_96a4_4d1b_a62f_f84d7987e64e </ci>
            </apply>
          </apply>
          <apply>
            <plus/>
            <cn type="integer"> 1 </cn>
            <apply>
              <divide/>
              <ci> mw5b528d37_b514_46e5_a018_0173afdae714 </ci>
              <ci> mwb4c2b40c_c637_4023_8d0f_5ed2bc9febf1 </ci>
            </apply>
          </apply>
        </apply>
      </apply>
      <cn type="integer"> 1 </cn>
    </apply>
  </apply>
</math>
</assignmentRule>
<assignmentRule metaid="repeatedAssignment_mw11c2dfc6_637a_4098_9369_89be59ff85c9"
variable="mwe559609f_0aab_4074_907d_7dd88bc5f228">
```

```
<math xmlns="http://www.w3.org/1998/Math/MathML">
  <apply>
    <divide/>
    <apply>
      <divide/>
      <apply>
        <times/>
        <ci> mwaad1b589_216c_4c43_92a0_68a64eaa5b3b </ci>
        <apply>
          <minus/>
          <apply>
            <times/>
            <ci> mw5b528d37_b514_46e5_a018_0173afdae714 </ci>
            <ci> mw8f67b710_8c1c_453f_a7ec_e9b6a55814fa </ci>
          </apply>
          <apply>
            <divide/>
            <ci> mw2a09f89f_5936_4376_a2ab_4ff1bbbd4153 </ci>
            <ci> mwa345d9e1_655b_41de_bcb1_d82b21b3c42c </ci>
          </apply>
        </apply>
      </apply>
      <apply>
        <times/>
        <ci> mwb15d9294_9509_4702_9083_ef51d8c235f9 </ci>
        <ci> mw8b39e257_03b8_4927_97c6_4b8444f70966 </ci>
      </apply>
    </apply>
    <apply>
      <minus/>
      <apply>
```

<plus/>
<apply>
<times/>
<apply>
<plus/>
<cn type="integer"> 1 </cn>
<apply>
<divide/>
<ci> mw2a09f89f_5936_4376_a2ab_4ff1bbbd4153 </ci>
<ci> mw1e28fdab_0576_4966_9927_17b091f2a4b2 </ci>
</apply>
</apply>
<apply>
<plus/>
<cn type="integer"> 1 </cn>
<apply>
<divide/>
<ci> mwacf08e72_f001_4a26_962d_729f504b1ad0 </ci>
<ci> mw777c1f25_305f_42cf_a010_b537a05884dd </ci>
</apply>
</apply>
</apply>
<apply>
<times/>
<apply>
<plus/>
<cn type="integer"> 1 </cn>
<apply>
<divide/>
<ci> mw8f67b710_8c1c_453f_a7ec_e9b6a55814fa </ci>
<ci> mwb15d9294_9509_4702_9083_ef51d8c235f9 </ci>

```
              </apply>
            </apply>
            <apply>
              <plus/>
              <cn type="integer"> 1 </cn>
              <apply>
                <divide/>
                <ci> mwaa8b93c8_308a_4d19_b732_22cb31f77620 </ci>
                <ci> mwc410441d_bd1d_4085_9e21_08b662e4f672 </ci>
              </apply>
            </apply>
            <apply>
              <plus/>
              <cn type="integer"> 1 </cn>
              <apply>
                <divide/>
                <ci> mw5b528d37_b514_46e5_a018_0173afdae714 </ci>
                <ci> mw8b39e257_03b8_4927_97c6_4b8444f70966 </ci>
              </apply>
            </apply>
          </apply>
        </apply>
        <cn type="integer"> 1 </cn>
      </apply>
    </apply>
  </math>
</assignmentRule>
<assignmentRule metaid="repeatedAssignment_mw9005099b_7ff1_4351_8252_69a79bdf9e61"
variable="mwb68b7376_788d_498e_b36d_36c89eeb1967">
  <math xmlns="http://www.w3.org/1998/Math/MathML">
    <apply>
```

```
<divide/>
<apply>
 <times/>
 <ci> mwa577376f_a6e1_47eb_bf4d_2b6dc53f1249 </ci>
 <apply>
  <minus/>
  <ci> mw372b8247_b11e_4d9b_be9b_e742fd2d3853 </ci>
  <apply>
   <divide/>
   <ci> mwc25bd841_1335_4e94_9ffa_621b205bc0da </ci>
   <ci> mw3f9f4337_1baa_4946_8e90_d13ca5c3adbf </ci>
  </apply>
 </apply>
</apply>
<apply>
 <times/>
 <ci> mw7d7f5782_23e4_4797_8dc7_355ec98f4d6a </ci>
 <apply>
  <plus/>
  <cn type="integer"> 1 </cn>
  <apply>
   <divide/>
   <ci> mw372b8247_b11e_4d9b_be9b_e742fd2d3853 </ci>
   <ci> mw7d7f5782_23e4_4797_8dc7_355ec98f4d6a </ci>
  </apply>
  <apply>
   <divide/>
   <ci> mwc25bd841_1335_4e94_9ffa_621b205bc0da </ci>
   <ci> mw28d0b788_21a0_4f17_ac4c_9b32d44ae058 </ci>
  </apply>
 </apply>
```

</apply>
</apply>
</math>
</assignmentRule>
<assignmentRule metaid="repeatedAssignment_mw9c26871a_7d6b_4317_add0_f880910f505a" variable="mw20119d5d_b91a_40db_882e_a27a7dacd159">
<math xmlns="http://www.w3.org/1998/Math/MathML">
<apply>
<divide/>
<apply>
<times/>
<ci> mw4674ad99_0bbe_4aef_99fa_49937a6f49d7 </ci>
<apply>
<minus/>
<ci> mw372b8247_b11e_4d9b_be9b_e742fd2d3853 </ci>
<apply>
<divide/>
<ci> mwc25bd841_1335_4e94_9ffa_621b205bc0da </ci>
<ci> mw3f9f4337_1baa_4946_8e90_d13ca5c3adbf </ci>
</apply>
</apply>
</apply>
<apply>
<times/>
<ci> mwc0011b36_3da6_4d34_9157_28ac5f77a5df </ci>
<apply>
<plus/>
<cn type="integer"> 1 </cn>
<apply>
<divide/>
<ci> mw372b8247_b11e_4d9b_be9b_e742fd2d3853 </ci>

<ci> mwc0011b36_3da6_4d34_9157_28ac5f77a5df </ci>

</apply>

<apply>

<divide/>

<ci> mwc25bd841_1335_4e94_9ffa_621b205bc0da </ci>

<ci> mw8a2c7a1e_c472_4f06_a4c7_38688feecabc </ci>

</apply>

</apply>

</apply>

</apply>

</math>

</assignmentRule>

<assignmentRule metaid="repeatedAssignment_mw5ec48354_b206_4ea8_9e6e_9f2530b4179e" variable="mw579e0b52_171d_4945_a0c2_8b5ab7cea441">

<math xmlns="http://www.w3.org/1998/Math/MathML">

<apply>

<divide/>

<apply>

<times/>

<ci> mw11591b16_f15e_41a6_890c_7da51ea27d51 </ci>

<apply>

<minus/>

<ci> mw372b8247_b11e_4d9b_be9b_e742fd2d3853 </ci>

<apply>

<divide/>

<ci> mwc25bd841_1335_4e94_9ffa_621b205bc0da </ci>

<ci> mw3f9f4337_1baa_4946_8e90_d13ca5c3adbf </ci>

</apply>

</apply>

</apply>

<apply>

```
            <times/>
            <ci> mw56023d39_08c8_49e5_a347_a28c871b8290 </ci>
            <apply>
              <plus/>
              <cn type="integer"> 1 </cn>
              <apply>
                <divide/>
                <ci> mw372b8247_b11e_4d9b_be9b_e742fd2d3853 </ci>
                <ci> mw56023d39_08c8_49e5_a347_a28c871b8290 </ci>
              </apply>
              <apply>
                <divide/>
                <ci> mwc25bd841_1335_4e94_9ffa_621b205bc0da </ci>
                <ci> mwc5650e27_4e7c_4064_8600_9ff3fd5f22ca </ci>
              </apply>
            </apply>
          </apply>
        </apply>
      </math>
    </assignmentRule>
    <assignmentRule metaid="repeatedAssignment_mwe257fdc8_402c_4ef4_b105_ca4b16da3ee0"
variable="mw76598380_dad6_4fbd_a40b_7281ee83b607">
      <math xmlns="http://www.w3.org/1998/Math/MathML">
        <apply>
          <divide/>
          <apply>
            <times/>
            <ci> mw98fecd51_ba08_4727_98ec_04637a47c5ac </ci>
            <apply>
              <divide/>
              <apply>
```

```
      <times/>
      <ci> mw70d358f1_f93f_432d_a6a3_d1aba42c9db7 </ci>
      <ci> mwf3d533fa_7176_4543_a1c4_f46f4dc4f8f6 </ci>
    </apply>
    <apply>
      <plus/>
      <ci> mwf3d533fa_7176_4543_a1c4_f46f4dc4f8f6 </ci>
      <apply>
        <times/>
        <ci> mw364ec325_c2af_4413_8934_e8b8af09f5f4 </ci>
        <apply>
          <plus/>
          <cn type="integer"> 1 </cn>
          <apply>
            <divide/>
            <ci> mw739c8f22_6a61_4dca_8cfe_3c962adb4128 </ci>
            <ci> mwe3972b9a_3c7e_4eb9_aa83_6bc48e5251c9 </ci>
          </apply>
        </apply>
      </apply>
    </apply>
  </apply>
</apply>
<apply>
  <plus/>
  <ci> mw98fecd51_ba08_4727_98ec_04637a47c5ac </ci>
  <apply>
    <times/>
    <ci> mw1aa79476_50cf_4ad8_b34b_1bd559d0e8b7 </ci>
    <apply>
      <plus/>
```

<cn type="integer"> 1 </cn>
<apply>
<divide/>
<ci> mw372b8247_b11e_4d9b_be9b_e742fd2d3853 </ci>
<ci> mw25c38b1f_a0cf_41ea_a0b9_ce591ec4bb41 </ci>
</apply>
<apply>
<divide/>
<ci> mw2a09f89f_5936_4376_a2ab_4ff1bbbd4153 </ci>
<ci> mwb9ff1179_1b4d_4f43_a08e_07a71dc0e53a </ci>
</apply>
<apply>
<divide/>
<ci> mwacf08e72_f001_4a26_962d_729f504b1ad0 </ci>
<ci> mwb765042e_909e_4002_adec_27be965841a3 </ci>
</apply>
<apply>
<divide/>
<ci> mw854ce178_2796_4118_8690_c13951a897c1 </ci>
<ci> mwef7f5344_1be5_414e_a10b_43c1f1ba59bb </ci>
</apply>
<apply>
<divide/>
<ci> mwd2e6e004_23f5_4cd8_81e0_8385021ec904 </ci>
<ci> mwaa92dfc9_9a33_4094_91a8_216d11908ae3 </ci>
</apply>
</apply>
</apply>
</apply>
</apply>
</math>

</assignmentRule>
<assignmentRule metaid="repeatedAssignment_mwad7f4c16_6202_4161_bbb1_2417a9e03b71" variable="mwf1a22b07_09a4_459c_bc0d_7ff0041d087f">
<math xmlns="http://www.w3.org/1998/Math/MathML">
<apply>
<minus/>
<apply>
<divide/>
<apply>
<times/>
<ci> mw202ff497_360c_4c69_8636_b9de7c9dd611 </ci>
<ci> mwc8b5cb3b_9f99_46df_a6e4_0126749144fc </ci>
<ci> mwd2e6e004_23f5_4cd8_81e0_8385021ec904 </ci>
</apply>
<apply>
<times/>
<apply>
<plus/>
<ci> mwc8b5cb3b_9f99_46df_a6e4_0126749144fc </ci>
<ci> mw2b7af3fa_4bf7_41ff_9e31_e4d8ae1a0567 </ci>
</apply>
<apply>
<plus/>
<ci> mwd2e6e004_23f5_4cd8_81e0_8385021ec904 </ci>
<ci> mw1243747a_17f7_4824_8201_696b9245f34d </ci>
</apply>
</apply>
</apply>
<apply>
<divide/>
<apply>

<times/>
<ci> mw38ba402b_328c_454f_807b_2a5d33b8d48f </ci>
<ci> mw5b528d37_b514_46e5_a018_0173afdae714 </ci>
<ci> mw7c834bb1_1865_4950_aa20_f3eceb92b21d </ci>
</apply>
<apply>
<times/>
<apply>
<plus/>
<cn type="integer"> 1 </cn>
<apply>
<divide/>
<ci> mw5b528d37_b514_46e5_a018_0173afdae714 </ci>
<ci> mw4da83b47_6347_43ed_b1b6_c95ab9075f9d </ci>
</apply>
<apply>
<divide/>
<ci> mw372b8247_b11e_4d9b_be9b_e742fd2d3853 </ci>
<ci> mw02a594bf_b380_46b9_acac_e7344c88da82 </ci>
</apply>
</apply>
<apply>
<plus/>
<cn type="integer"> 1 </cn>
<apply>
<divide/>
<ci> mw7c834bb1_1865_4950_aa20_f3eceb92b21d </ci>
<ci> mw640c5b04_67dd_43d7_8b11_069cf242288b </ci>
</apply>
<apply>
<divide/>

<ci> mwd2e6e004_23f5_4cd8_81e0_8385021ec904 </ci>

<ci> mwebd2cdb3_d654_4e7e_a036_7180e1269833 </ci>

</apply>

</apply>

</apply>

</apply>

</apply>

</math>

</assignmentRule>

<assignmentRule metaid="repeatedAssignment_mwc2ea13c4_af23_483b_be7c_9d849cee84c3" variable="mw61ee8550_d991_4a56_922f_f261a0a492f4">

<math xmlns="http://www.w3.org/1998/Math/MathML">

<apply>

<divide/>

<apply>

<times/>

<ci> mw376c4606_38fa_4a60_9b24_8ef462cbe2ff </ci>

<ci> mwb8027448_5c51_43ad_9f13_cd37b5418a54 </ci>

<ci> mw7c834bb1_1865_4950_aa20_f3eceb92b21d </ci>

</apply>

<apply>

<times/>

<apply>

<plus/>

<ci> mwb8027448_5c51_43ad_9f13_cd37b5418a54 </ci>

<ci> mw9e08e044_96ba_486f_9778_f5a4ea04cff4 </ci>

</apply>

<apply>

<plus/>

<ci> mw7c834bb1_1865_4950_aa20_f3eceb92b21d </ci>

<ci> mwf24759b0_36bc_4a60_b869_301f6c1f2d80 </ci>

</apply>
</apply>
</apply>
</math>
</assignmentRule>
<assignmentRule metaid="repeatedAssignment_mw79d2ca54_dcd9_48b4_97cd_4339742c066c" variable="mw3bab02fa_c9cf_4ae8_baa1_491eac4c7a7f">
<math xmlns="http://www.w3.org/1998/Math/MathML">
<apply>
<divide/>
<apply>
<divide/>
<apply>
<times/>
<ci> mw6e7fabe6_de80_41fa_9146_5e9b7ec4bc63 </ci>
<apply>
<minus/>
<ci> mwfcedd013_9e1c_4e21_9a5a_93ddde2ffc11 </ci>
<apply>
<divide/>
<apply>
<times/>
<ci> mw543ef166_8322_44f0_9435_270a9f9672da </ci>
<ci> mwaa8b93c8_308a_4d19_b732_22cb31f77620 </ci>
</apply>
<ci> mw066182b3_2182_44b1_b745_125e1968e0bc </ci>
</apply>
</apply>
</apply>
<ci> mw8e6b4f11_baec_4a0f_983d_3d1d46cb91cb </ci>
</apply>

```
<apply>
  <times/>
  <apply>
    <plus/>
    <cn type="integer"> 1 </cn>
    <apply>
      <divide/>
      <ci> mwfcedd013_9e1c_4e21_9a5a_93ddde2ffc11 </ci>
      <ci> mw8e6b4f11_baec_4a0f_983d_3d1d46cb91cb </ci>
    </apply>
    <apply>
      <divide/>
      <ci> mwaa8b93c8_308a_4d19_b732_22cb31f77620 </ci>
      <ci> mw8b16f07a_00d8_489e_ae07_2dc92af4dfb9 </ci>
    </apply>
  </apply>
  <apply>
    <plus/>
    <cn type="integer"> 1 </cn>
    <apply>
      <divide/>
      <ci> mw543ef166_8322_44f0_9435_270a9f9672da </ci>
      <ci> mw514d5907_3da1_4e17_8024_f637af4b38fa </ci>
    </apply>
  </apply>
  <apply>
    <plus/>
    <cn type="integer"> 1 </cn>
    <apply>
      <divide/>
      <ci> mw7c70a014_6b69_42b3_9fbd_1d63cb722ae5 </ci>
```

<ci> mw562c606a_6226_47bc_8fe9_db84aa73a146 </ci>
</apply>
<apply>
<divide/>
<ci> mw5b528d37_b514_46e5_a018_0173afdae714 </ci>
<ci> mw9d34e88a_6503_48b9_a787_58c326a932a5 </ci>
</apply>
</apply>
</apply>
</apply>
</math>
</assignmentRule>
<assignmentRule metaid="repeatedAssignment_mw75afd8ef_040c_4930_ad1c_8e9b4cb323a4" variable="mwc61d80c8_a1a3_4704_b02a_baaff2bc07f2">
<math xmlns="http://www.w3.org/1998/Math/MathML">
<apply>
<divide/>
<apply>
<divide/>
<apply>
<times/>
<ci> mw0ab3c1a9_2d85_4242_b2c8_8668592bc4b4 </ci>
<apply>
<minus/>
<ci> mw7c70a014_6b69_42b3_9fbd_1d63cb722ae5 </ci>
<apply>
<divide/>
<apply>
<times/>
<ci> mw543ef166_8322_44f0_9435_270a9f9672da </ci>
<ci> mw5b528d37_b514_46e5_a018_0173afdae714 </ci>

</apply>
<ci> mw44c8ec20_4a1e_454f_a1a0_42d862b117ae </ci>
</apply>
</apply>
</apply>
<ci> mw057e7327_64bf_4b85_bb90_78da6ba5980f </ci>
</apply>
<apply>
<times/>
<apply>
<plus/>
<cn type="integer"> 1 </cn>
<apply>
<divide/>
<ci> mwfcedd013_9e1c_4e21_9a5a_93ddde2ffc11 </ci>
<ci> mw77007da8_e432_48eb_9c52_ed3c2c1b5f8c </ci>
</apply>
<apply>
<divide/>
<ci> mwaa8b93c8_308a_4d19_b732_22cb31f77620 </ci>
<ci> mw9580aa5c_a5df_472e_aad4_d7d8dfaf50c5 </ci>
</apply>
</apply>
<apply>
<plus/>
<cn type="integer"> 1 </cn>
<apply>
<divide/>
<ci> mw543ef166_8322_44f0_9435_270a9f9672da </ci>
<ci> mw6dff67cf_5c46_4621_9902_c290a3f63d5d </ci>
</apply>

```
        </apply>
        <apply>
          <plus/>
          <cn type="integer"> 1 </cn>
          <apply>
            <divide/>
            <ci> mw7c70a014_6b69_42b3_9fbd_1d63cb722ae5 </ci>
            <ci> mw057e7327_64bf_4b85_bb90_78da6ba5980f </ci>
          </apply>
          <apply>
            <divide/>
            <ci> mw5b528d37_b514_46e5_a018_0173afdae714 </ci>
            <ci> mwa0585ea2_d52f_4310_adf2_e8de58065ae9 </ci>
          </apply>
        </apply>
      </apply>
    </apply>
  </math>
</assignmentRule>
<assignmentRule metaid="repeatedAssignment_mw529c69c6_ccf7_4b92_9b86_b14382ac4eea" variable="mw79196e59_a83a_4059_afcd_3fbbb659acf9">
  <math xmlns="http://www.w3.org/1998/Math/MathML">
    <apply>
      <divide/>
      <apply>
        <divide/>
        <apply>
          <times/>
          <ci> mw9e5312fb_9ca1_4ae7_8fef_e40c70633af3 </ci>
          <apply>
            <minus/>
```

<ci> mwfcedd013_9e1c_4e21_9a5a_93ddde2ffc11 </ci>
<apply>
<divide/>
<apply>
<times/>
<ci> mw543ef166_8322_44f0_9435_270a9f9672da </ci>
<ci> mwaa8b93c8_308a_4d19_b732_22cb31f77620 </ci>
</apply>
<ci> mw066182b3_2182_44b1_b745_125e1968e0bc </ci>
</apply>
</apply>
</apply>
<ci> mw7c84a538_b915_4a91_8cc2_04c534c01ab8 </ci>
</apply>
<apply>
<times/>
<apply>
<plus/>
<cn type="integer"> 1 </cn>
<apply>
<divide/>
<ci> mwfcedd013_9e1c_4e21_9a5a_93ddde2ffc11 </ci>
<ci> mw7c84a538_b915_4a91_8cc2_04c534c01ab8 </ci>
</apply>
<apply>
<divide/>
<ci> mwaa8b93c8_308a_4d19_b732_22cb31f77620 </ci>
<ci> mw4d62dae0_b6d6_45ba_8fb6_caf10d3295dc </ci>
</apply>
</apply>
<apply>

```
            <plus/>
            <cn type="integer"> 1 </cn>
            <apply>
              <divide/>
              <ci> mw543ef166_8322_44f0_9435_270a9f9672da </ci>
              <ci> mw9f4be612_37b6_425f_863f_c3234aaafd9c </ci>
            </apply>
          </apply>
          <apply>
            <plus/>
            <cn type="integer"> 1 </cn>
            <apply>
              <divide/>
              <ci> mw7c70a014_6b69_42b3_9fbd_1d63cb722ae5 </ci>
              <ci> mw457a9b1f_ac25_45b4_adb7_c6c807be3474 </ci>
            </apply>
            <apply>
              <divide/>
              <ci> mw5b528d37_b514_46e5_a018_0173afdae714 </ci>
              <ci> mwa723f027_cc0e_426d_b99e_dd1f00ba7a3e </ci>
            </apply>
          </apply>
        </apply>
      </apply>
    </math>
  </assignmentRule>
  <assignmentRule metaid="repeatedAssignment_mw65e8f9d3_85de_447b_a1e1_4c7b86b9fe50"
variable="mw837df40e_afa2_42bb_88ef_c02281c03e49">
    <math xmlns="http://www.w3.org/1998/Math/MathML">
      <apply>
        <divide/>
```

```
<apply>
  <divide/>
  <apply>
    <times/>
    <ci> mw5a9817b0_1b0d_4667_a467_59f6072696c8 </ci>
    <apply>
      <minus/>
      <ci> mw7c70a014_6b69_42b3_9fbd_1d63cb722ae5 </ci>
      <apply>
        <divide/>
        <apply>
          <times/>
          <ci> mw543ef166_8322_44f0_9435_270a9f9672da </ci>
          <ci> mw5b528d37_b514_46e5_a018_0173afdae714 </ci>
        </apply>
        <ci> mw44c8ec20_4a1e_454f_a1a0_42d862b117ae </ci>
      </apply>
    </apply>
  </apply>
  <ci> mwff222c2b_bc40_4d59_bfcd_63aeb04c86bd </ci>
</apply>
<apply>
  <times/>
  <apply>
    <plus/>
    <cn type="integer"> 1 </cn>
    <apply>
      <divide/>
      <ci> mwfcedd013_9e1c_4e21_9a5a_93ddde2ffc11 </ci>
      <ci> mw08f2e5c2_6905_4c90_8f37_016935f5d8ec </ci>
    </apply>
```

```
      <apply>
        <divide/>
        <ci> mwaa8b93c8_308a_4d19_b732_22cb31f77620 </ci>
        <ci> mw4c5adb36_23ae_4318_8b5f_6f80df2be00a </ci>
      </apply>
    </apply>
    <apply>
      <plus/>
      <cn type="integer"> 1 </cn>
      <apply>
        <divide/>
        <ci> mw543ef166_8322_44f0_9435_270a9f9672da </ci>
        <ci> mwc6a7cd20_1464_4521_88e6_145752ed585d </ci>
      </apply>
    </apply>
    <apply>
      <plus/>
      <cn type="integer"> 1 </cn>
      <apply>
        <divide/>
        <ci> mw7c70a014_6b69_42b3_9fbd_1d63cb722ae5 </ci>
        <ci> mwff222c2b_bc40_4d59_bfcd_63aeb04c86bd </ci>
      </apply>
      <apply>
        <divide/>
        <ci> mw5b528d37_b514_46e5_a018_0173afdae714 </ci>
        <ci> mw8d162e7c_404d_4423_8f50_9c55f37e0d2c </ci>
      </apply>
    </apply>
  </apply>
</apply>
```

```
        </math>
      </assignmentRule>
    </listOfRules>
    <listOfReactions>
      <reaction id="mw15ff2a0e_c8d7_4775_a757_45e50437c52f" name="CO2 import"
reversible="false" fast="false">
        <listOfReactants>
          <speciesReference species="mwc740f83b_ea86_4945_a732_4b08a717ae97"/>
        </listOfReactants>
        <listOfProducts>
          <speciesReference species="mwf3d533fa_7176_4543_a1c4_f46f4dc4f8f6"/>
        </listOfProducts>
        <kineticLaw>
          <math xmlns="http://www.w3.org/1998/Math/MathML">
            <apply>
              <times/>
              <ci> mwf78a87c8_a526_4514_ad30_2df654c3ee39 </ci>
              <ci> mwc740f83b_ea86_4945_a732_4b08a717ae97 </ci>
            </apply>
          </math>
        </kineticLaw>
      </reaction>
      <reaction id="mwd121108f_50d9_4992_ab39_9051c116661d" name="LIGHT_1 (ATP synthase)"
reversible="false" fast="false">
        <annotation>
          <COPASI xmlns="http://www.copasi.org/static/sbml">
            <rdf:RDF xmlns:dcterms="http://purl.org/dc/terms/"
xmlns:rdf="http://www.w3.org/1999/02/22-rdf-syntax-ns#">
              <rdf:Description rdf:about="#COPASI65">
                <dcterms:created>
                  <rdf:Description>
                    <dcterms:W3CDTF>2010-05-24T02:11:27Z</dcterms:W3CDTF>
```

```
          </rdf:Description>
        </dcterms:created>
      </rdf:Description>
    </rdf:RDF>
  </COPASI>
</annotation>
<listOfReactants>
  <speciesReference species="mw854ce178_2796_4118_8690_c13951a897c1"/>
  <speciesReference species="mwe8d1ae1f_35f9_45fd_bedf_7dbeef3972e2"/>
</listOfReactants>
<listOfProducts>
  <speciesReference species="mw8e2cc29c_c73e_4258_b3ba_2356620bb64e"/>
</listOfProducts>
<kineticLaw>
  <math xmlns="http://www.w3.org/1998/Math/MathML">
    <apply>
      <times/>
      <ci> mw3f34c374_f765_4309_b3fd_9064029073d1 </ci>
      <ci> mw854ce178_2796_4118_8690_c13951a897c1 </ci>
      <ci> mwe8d1ae1f_35f9_45fd_bedf_7dbeef3972e2 </ci>
    </apply>
  </math>
</kineticLaw>
</reaction>
<reaction id="mw3dff9642_a267_4ad3_95ac_d074da4c4474" name="LIGHT_2"
reversible="false" fast="false">
<annotation>
  <COPASI xmlns="http://www.copasi.org/static/sbml">
    <rdf:RDF xmlns:dcterms="http://purl.org/dc/terms/"
xmlns:rdf="http://www.w3.org/1999/02/22-rdf-syntax-ns#">
      <rdf:Description rdf:about="#COPASI66">
        <dcterms:created>
```

```
              <rdf:Description>
                <dcterms:W3CDTF>2010-06-09T00:18:40Z</dcterms:W3CDTF>
              </rdf:Description>
            </dcterms:created>
          </rdf:Description>
        </rdf:RDF>
      </COPASI>
    </annotation>
    <listOfReactants>
      <speciesReference species="mw7c834bb1_1865_4950_aa20_f3eceb92b21d"/>
    </listOfReactants>
    <listOfProducts>
      <speciesReference species="mwd2e6e004_23f5_4cd8_81e0_8385021ec904"/>
    </listOfProducts>
    <kineticLaw>
      <math xmlns="http://www.w3.org/1998/Math/MathML">
        <apply>
          <times/>
          <ci> mw950bbc4b_e9ec_43a5_84c9_010563093363 </ci>
          <ci> mw7c834bb1_1865_4950_aa20_f3eceb92b21d </ci>
        </apply>
      </math>
    </kineticLaw>
  </reaction>
  <reaction id="mw8aea24fe_27ea_4574_8d64_3b739e66ad8a" name="CC_1 (RuBisCO)"
reversible="false" fast="false">
    <annotation>
      <COPASI xmlns="http://www.copasi.org/static/sbml">
        <rdf:RDF xmlns:dcterms="http://purl.org/dc/terms/"
xmlns:rdf="http://www.w3.org/1999/02/22-rdf-syntax-ns#">
          <rdf:Description rdf:about="#COPASI49">
            <dcterms:created>
```

```
          <rdf:Description>
            <dcterms:W3CDTF>2010-04-14T14:47:32Z</dcterms:W3CDTF>
          </rdf:Description>
        </dcterms:created>
      </rdf:Description>
    </rdf:RDF>
  </COPASI>
</annotation>
<listOfReactants>
  <speciesReference species="mw98fecd51_ba08_4727_98ec_04637a47c5ac"/>
  <speciesReference species="mwf3d533fa_7176_4543_a1c4_f46f4dc4f8f6"/>
  <speciesReference species="mw739c8f22_6a61_4dca_8cfe_3c962adb4128"/>
  <speciesReference species="mw2a09f89f_5936_4376_a2ab_4ff1bbbd4153"/>
  <speciesReference species="mwacf08e72_f001_4a26_962d_729f504b1ad0"/>
  <speciesReference species="mw854ce178_2796_4118_8690_c13951a897c1"/>
  <speciesReference species="mwd2e6e004_23f5_4cd8_81e0_8385021ec904"/>
</listOfReactants>
<listOfProducts>
  <speciesReference species="mw372b8247_b11e_4d9b_be9b_e742fd2d3853"
stoichiometry="2"/>
  <speciesReference species="mw739c8f22_6a61_4dca_8cfe_3c962adb4128"/>
  <speciesReference species="mw2a09f89f_5936_4376_a2ab_4ff1bbbd4153"/>
  <speciesReference species="mwacf08e72_f001_4a26_962d_729f504b1ad0"/>
  <speciesReference species="mw854ce178_2796_4118_8690_c13951a897c1"/>
  <speciesReference species="mwd2e6e004_23f5_4cd8_81e0_8385021ec904"/>
</listOfProducts>
<kineticLaw>
  <math xmlns="http://www.w3.org/1998/Math/MathML">
    <apply>
      <divide/>
      <apply>
```

```
  <times/>
  <ci> mw98fecd51_ba08_4727_98ec_04637a47c5ac </ci>
  <apply>
    <divide/>
    <apply>
      <times/>
      <ci> mwc13f7f00_20f5_4411_8231_622067f80bcb </ci>
      <ci> mwf3d533fa_7176_4543_a1c4_f46f4dc4f8f6 </ci>
    </apply>
    <apply>
      <plus/>
      <ci> mwf3d533fa_7176_4543_a1c4_f46f4dc4f8f6 </ci>
      <apply>
        <times/>
        <ci> mw364ec325_c2af_4413_8934_e8b8af09f5f4 </ci>
        <apply>
          <plus/>
          <cn type="integer"> 1 </cn>
          <apply>
            <divide/>
            <ci> mw739c8f22_6a61_4dca_8cfe_3c962adb4128 </ci>
            <ci> mwe3972b9a_3c7e_4eb9_aa83_6bc48e5251c9 </ci>
          </apply>
        </apply>
      </apply>
    </apply>
  </apply>
</apply>
<apply>
  <plus/>
  <ci> mw98fecd51_ba08_4727_98ec_04637a47c5ac </ci>
```

<apply>
<times/>
<ci> mw1aa79476_50cf_4ad8_b34b_1bd559d0e8b7 </ci>
<apply>
<plus/>
<cn type="integer"> 1 </cn>
<apply>
<divide/>
<ci> mw372b8247_b11e_4d9b_be9b_e742fd2d3853 </ci>
<ci> mw25c38b1f_a0cf_41ea_a0b9_ce591ec4bb41 </ci>
</apply>
<apply>
<divide/>
<ci> mw2a09f89f_5936_4376_a2ab_4ff1bbbd4153 </ci>
<ci> mwb9ff1179_1b4d_4f43_a08e_07a71dc0e53a </ci>
</apply>
<apply>
<divide/>
<ci> mwacf08e72_f001_4a26_962d_729f504b1ad0 </ci>
<ci> mwb765042e_909e_4002_adec_27be965841a3 </ci>
</apply>
<apply>
<divide/>
<ci> mw854ce178_2796_4118_8690_c13951a897c1 </ci>
<ci> mwef7f5344_1be5_414e_a10b_43c1f1ba59bb </ci>
</apply>
<apply>
<divide/>
<ci> mwd2e6e004_23f5_4cd8_81e0_8385021ec904 </ci>
<ci> mwaa92dfc9_9a33_4094_91a8_216d11908ae3 </ci>
</apply>

```
            </apply>
           </apply>
          </apply>
         </apply>
        </math>
       </kineticLaw>
      </reaction>
      <reaction id="mw14a87fbb_e4e9_4201_b249_eb6d74860cce" name="CC_2 (phosphoglycerate
kinase)" fast="false">
       <notes>
        <body xmlns="http://www.w3.org/1999/xhtml">
         <pre>Vmax sn&#xeD;&#x17e;eno z 10.3 na 2</pre>
        </body>
       </notes>
       <annotation>
        <COPASI xmlns="http://www.copasi.org/static/sbml">
         <rdf:RDF xmlns:dcterms="http://purl.org/dc/terms/"
xmlns:rdf="http://www.w3.org/1999/02/22-rdf-syntax-ns#">
          <rdf:Description rdf:about="#COPASI50">
           <dcterms:created>
            <rdf:Description>
             <dcterms:W3CDTF>2010-04-14T15:57:26Z</dcterms:W3CDTF>
            </rdf:Description>
           </dcterms:created>
          </rdf:Description>
         </rdf:RDF>
        </COPASI>
       </annotation>
       <listOfReactants>
        <speciesReference species="mw372b8247_b11e_4d9b_be9b_e742fd2d3853"/>
        <speciesReference species="mw8e2cc29c_c73e_4258_b3ba_2356620bb64e"/>
       </listOfReactants>
```

```xml
<listOfProducts>
  <speciesReference species="mwc8b5cb3b_9f99_46df_a6e4_0126749144fc"/>
  <speciesReference species="mwe8d1ae1f_35f9_45fd_bedf_7dbeef3972e2"/>
</listOfProducts>
<kineticLaw>
  <math xmlns="http://www.w3.org/1998/Math/MathML">
    <apply>
      <divide/>
      <apply>
        <divide/>
        <apply>
          <times/>
          <ci> mw67b39301_8673_4822_8409_6fb4afa9cfa4 </ci>
          <apply>
            <minus/>
            <apply>
              <times/>
              <ci> mw372b8247_b11e_4d9b_be9b_e742fd2d3853 </ci>
              <ci> mw8e2cc29c_c73e_4258_b3ba_2356620bb64e </ci>
            </apply>
            <apply>
              <divide/>
              <apply>
                <times/>
                <ci> mwc8b5cb3b_9f99_46df_a6e4_0126749144fc </ci>
                <ci> mwe8d1ae1f_35f9_45fd_bedf_7dbeef3972e2 </ci>
              </apply>
              <ci> mw9896ea60_a024_4836_a456_1e419a52deb6 </ci>
            </apply>
          </apply>
        </apply>
```

<apply>
<times/>
<ci> mwc2cdab10_3c94_4f4b_b95b_d8af8de7d09e </ci>
<ci> mwcc3e1093_f15d_43ba_803f_07c7f8d7c816 </ci>
</apply>
</apply>
<apply>
<times/>
<apply>
<plus/>
<cn type="integer"> 1 </cn>
<apply>
<divide/>
<ci> mw372b8247_b11e_4d9b_be9b_e742fd2d3853 </ci>
<ci> mwc2cdab10_3c94_4f4b_b95b_d8af8de7d09e </ci>
</apply>
<apply>
<divide/>
<ci> mwc8b5cb3b_9f99_46df_a6e4_0126749144fc </ci>
<ci> mwdb043b2e_d92d_4148_accd_2ed71aad1897 </ci>
</apply>
</apply>
<apply>
<plus/>
<cn type="integer"> 1 </cn>
<apply>
<divide/>
<ci> mw8e2cc29c_c73e_4258_b3ba_2356620bb64e </ci>
<ci> mwcc3e1093_f15d_43ba_803f_07c7f8d7c816 </ci>
</apply>
<apply>

<divide/>

<ci> mwe8d1ae1f_35f9_45fd_bedf_7dbeef3972e2 </ci>

<ci> mw8204a9d2_3043_47db_96f6_061c456138f4 </ci>

</apply>

</apply>

</apply>

</apply>

</math>

</kineticLaw>

</reaction>

<reaction id="mwb833460f_da31_4272_91cc_dc4c130b0bdd" name="CC_3 (glyceraldehyde 3-phosphate dehydrogenase)" reversible="false" fast="false">

<notes>

cannot fit the parameters, even irreversible, model is super sensitive

</notes>

<annotation>

<COPASI xmlns="http://www.copasi.org/static/sbml">

<rdf:RDF xmlns:dcterms="http://purl.org/dc/terms/" xmlns:rdf="http://www.w3.org/1999/02/22-rdf-syntax-ns#">

<rdf:Description rdf:about="#COPASI51">

<dcterms:created>

<rdf:Description>

<dcterms:W3CDTF>2010-04-14T16:10:42Z</dcterms:W3CDTF>

</rdf:Description>

</dcterms:created>

</rdf:Description>

</rdf:RDF>

</COPASI>

</annotation>

<listOfReactants>

<speciesReference species="mwc8b5cb3b_9f99_46df_a6e4_0126749144fc"/>

<speciesReference species="mwd2e6e004_23f5_4cd8_81e0_8385021ec904"/>

```
</listOfReactants>
<listOfProducts>
 <speciesReference species="mw5b528d37_b514_46e5_a018_0173afdae714"/>
 <speciesReference species="mw7c834bb1_1865_4950_aa20_f3eceb92b21d"/>
</listOfProducts>
<kineticLaw>
 <math xmlns="http://www.w3.org/1998/Math/MathML">
  <apply>
   <divide/>
   <apply>
    <times/>
    <ci> mw202ff497_360c_4c69_8636_b9de7c9dd611 </ci>
    <ci> mwc8b5cb3b_9f99_46df_a6e4_0126749144fc </ci>
    <ci> mwd2e6e004_23f5_4cd8_81e0_8385021ec904 </ci>
   </apply>
   <apply>
    <times/>
    <apply>
     <plus/>
     <ci> mwc8b5cb3b_9f99_46df_a6e4_0126749144fc </ci>
     <ci> mw2b7af3fa_4bf7_41ff_9e31_e4d8ae1a0567 </ci>
    </apply>
    <apply>
     <plus/>
     <ci> mwd2e6e004_23f5_4cd8_81e0_8385021ec904 </ci>
     <ci> mw1243747a_17f7_4824_8201_696b9245f34d </ci>
    </apply>
   </apply>
  </apply>
 </math>
</kineticLaw>
```

```
</reaction>
<reaction id="mw0bfdec44_82eb_4c8c_8f58_513703b26f6d" name="CC_4 (triose phosphate isomerase)" fast="false">
<annotation>
<COPASI xmlns="http://www.copasi.org/static/sbml">
<rdf:RDF xmlns:dcterms="http://purl.org/dc/terms/" xmlns:rdf="http://www.w3.org/1999/02/22-rdf-syntax-ns#">
<rdf:Description rdf:about="#COPASI52">
<dcterms:created>
<rdf:Description>
<dcterms:W3CDTF>2010-04-14T16:18:37Z</dcterms:W3CDTF>
</rdf:Description>
</dcterms:created>
</rdf:Description>
</rdf:RDF>
</COPASI>
</annotation>
<listOfReactants>
<speciesReference species="mw5b528d37_b514_46e5_a018_0173afdae714"/>
</listOfReactants>
<listOfProducts>
<speciesReference species="mw8f67b710_8c1c_453f_a7ec_e9b6a55814fa"/>
</listOfProducts>
<kineticLaw>
<math xmlns="http://www.w3.org/1998/Math/MathML">
<apply>
<divide/>
<apply>
<divide/>
<apply>
<times/>
<ci> mw22fbdd6b_5631_460f_8df1_6a49a247a909 </ci>
```

```
      <apply>
       <minus/>
       <ci> mw5b528d37_b514_46e5_a018_0173afdae714 </ci>
       <apply>
        <divide/>
        <ci> mw8f67b710_8c1c_453f_a7ec_e9b6a55814fa </ci>
        <ci> mw4d8c7a18_e85a_4f29_aca5_7580ecebdacf </ci>
       </apply>
      </apply>
     </apply>
     <ci> mw4372d7eb_dfbb_483c_ac0d_eeaa38580e0b </ci>
    </apply>
    <apply>
     <plus/>
     <cn type="integer"> 1 </cn>
     <apply>
      <divide/>
      <ci> mw5b528d37_b514_46e5_a018_0173afdae714 </ci>
      <ci> mw4372d7eb_dfbb_483c_ac0d_eeaa38580e0b </ci>
     </apply>
     <apply>
      <divide/>
      <ci> mw8f67b710_8c1c_453f_a7ec_e9b6a55814fa </ci>
      <ci> mw60b27f8d_8f84_4edc_b378_ca1bab69e489 </ci>
     </apply>
    </apply>
   </apply>
  </math>
 </kineticLaw>
</reaction>
```

```
<reaction id="mw785e70cf_68a3_43f6_9e70_447058bb9f77" name="CC_5 (aldolase) alpha"
fast="false">
  <annotation>
    <COPASI xmlns="http://www.copasi.org/static/sbml">
      <rdf:RDF xmlns:dcterms="http://purl.org/dc/terms/"
xmlns:rdf="http://www.w3.org/1999/02/22-rdf-syntax-ns#">
        <rdf:Description rdf:about="#COPASI53">
          <dcterms:created>
            <rdf:Description>
              <dcterms:W3CDTF>2010-04-14T16:46:06Z</dcterms:W3CDTF>
            </rdf:Description>
          </dcterms:created>
        </rdf:Description>
      </rdf:RDF>
    </COPASI>
  </annotation>
  <listOfReactants>
    <speciesReference species="mw5b528d37_b514_46e5_a018_0173afdae714"/>
    <speciesReference species="mw8f67b710_8c1c_453f_a7ec_e9b6a55814fa"/>
    <speciesReference species="mwacf08e72_f001_4a26_962d_729f504b1ad0"/>
    <speciesReference species="mwaa8b93c8_308a_4d19_b732_22cb31f77620"/>
  </listOfReactants>
  <listOfProducts>
    <speciesReference species="mw2a09f89f_5936_4376_a2ab_4ff1bbbd4153"/>
    <speciesReference species="mwacf08e72_f001_4a26_962d_729f504b1ad0"/>
    <speciesReference species="mwaa8b93c8_308a_4d19_b732_22cb31f77620"/>
  </listOfProducts>
  <kineticLaw>
    <math xmlns="http://www.w3.org/1998/Math/MathML">
      <apply>
        <divide/>
        <apply>
```

<divide/>
<apply>
<times/>
<ci> mw538c4cd4_f0aa_4fd6_8f8a_11b3e718bdca </ci>
<apply>
<minus/>
<apply>
<times/>
<ci> mw5b528d37_b514_46e5_a018_0173afdae714 </ci>
<ci> mw8f67b710_8c1c_453f_a7ec_e9b6a55814fa </ci>
</apply>
<apply>
<divide/>
<ci> mw2a09f89f_5936_4376_a2ab_4ff1bbbd4153 </ci>
<ci> mwa345d9e1_655b_41de_bcb1_d82b21b3c42c </ci>
</apply>
</apply>
</apply>
<apply>
<times/>
<ci> mw6e554aaa_4cb8_46a2_9af0_40c527042d95 </ci>
<ci> mwb4c2b40c_c637_4023_8d0f_5ed2bc9febf1 </ci>
</apply>
</apply>
<apply>
<minus/>
<apply>
<plus/>
<apply>
<times/>
<apply>

<plus/>
<cn type="integer"> 1 </cn>
<apply>
<divide/>
<ci> mw2a09f89f_5936_4376_a2ab_4ff1bbbd4153 </ci>
<ci> mwef61df7c_5b85_41cf_b539_58e829001c39 </ci>
</apply>
</apply>
<apply>
<plus/>
<cn type="integer"> 1 </cn>
<apply>
<divide/>
<ci> mwacf08e72_f001_4a26_962d_729f504b1ad0 </ci>
<ci> mw9d553771_b33e_40de_bbc5_22c5804e335a </ci>
</apply>
</apply>
</apply>
<apply>
<times/>
<apply>
<plus/>
<cn type="integer"> 1 </cn>
<apply>
<divide/>
<ci> mw8f67b710_8c1c_453f_a7ec_e9b6a55814fa </ci>
<ci> mw6e554aaa_4cb8_46a2_9af0_40c527042d95 </ci>
</apply>
</apply>
<apply>
<plus/>

```
              <cn type="integer"> 1 </cn>
              <apply>
                <divide/>
                <ci> mwaa8b93c8_308a_4d19_b732_22cb31f77620 </ci>
                <ci> mw2ffe78db_96a4_4d1b_a62f_f84d7987e64e </ci>
              </apply>
            </apply>
            <apply>
              <plus/>
              <cn type="integer"> 1 </cn>
              <apply>
                <divide/>
                <ci> mw5b528d37_b514_46e5_a018_0173afdae714 </ci>
                <ci> mwb4c2b40c_c637_4023_8d0f_5ed2bc9febf1 </ci>
              </apply>
            </apply>
          </apply>
        </apply>
        <cn type="integer"> 1 </cn>
      </apply>
    </apply>
   </math>
  </kineticLaw>
 </reaction>
 <reaction id="mw57cd0afb_bfa4_4b7f_9dcc_fd3be5dfd9b0" name="CC_5 (aldolase) beta"
fast="false">
  <listOfReactants>
   <speciesReference species="mw5b528d37_b514_46e5_a018_0173afdae714"/>
   <speciesReference species="mw8f67b710_8c1c_453f_a7ec_e9b6a55814fa"/>
   <speciesReference species="mwacf08e72_f001_4a26_962d_729f504b1ad0"/>
   <speciesReference species="mwaa8b93c8_308a_4d19_b732_22cb31f77620"/>
```

```
</listOfReactants>
<listOfProducts>
  <speciesReference species="mw2a09f89f_5936_4376_a2ab_4ff1bbbd4153"/>
  <speciesReference species="mwacf08e72_f001_4a26_962d_729f504b1ad0"/>
  <speciesReference species="mwaa8b93c8_308a_4d19_b732_22cb31f77620"/>
</listOfProducts>
<kineticLaw>
  <math xmlns="http://www.w3.org/1998/Math/MathML">
    <apply>
      <divide/>
      <apply>
        <divide/>
        <apply>
          <times/>
          <ci> mwaad1b589_216c_4c43_92a0_68a64eaa5b3b </ci>
          <apply>
            <minus/>
            <apply>
              <times/>
              <ci> mw5b528d37_b514_46e5_a018_0173afdae714 </ci>
              <ci> mw8f67b710_8c1c_453f_a7ec_e9b6a55814fa </ci>
            </apply>
            <apply>
              <divide/>
              <ci> mw2a09f89f_5936_4376_a2ab_4ff1bbbd4153 </ci>
              <ci> mwa345d9e1_655b_41de_bcb1_d82b21b3c42c </ci>
            </apply>
          </apply>
        </apply>
        <apply>
          <times/>
```

<ci> mwb15d9294_9509_4702_9083_ef51d8c235f9 </ci>
<ci> mw8b39e257_03b8_4927_97c6_4b8444f70966 </ci>
</apply>
</apply>
<apply>
<minus/>
<apply>
<plus/>
<apply>
<times/>
<apply>
<plus/>
<cn type="integer"> 1 </cn>
<apply>
<divide/>
<ci> mw2a09f89f_5936_4376_a2ab_4ff1bbbd4153 </ci>
<ci> mw1e28fdab_0576_4966_9927_17b091f2a4b2 </ci>
</apply>
</apply>
<apply>
<plus/>
<cn type="integer"> 1 </cn>
<apply>
<divide/>
<ci> mwacf08e72_f001_4a26_962d_729f504b1ad0 </ci>
<ci> mw777c1f25_305f_42cf_a010_b537a05884dd </ci>
</apply>
</apply>
</apply>
<apply>
<times/>

```
    <apply>
     <plus/>
     <cn type="integer"> 1 </cn>
     <apply>
      <divide/>
      <ci> mw8f67b710_8c1c_453f_a7ec_e9b6a55814fa </ci>
      <ci> mwb15d9294_9509_4702_9083_ef51d8c235f9 </ci>
     </apply>
    </apply>
    <apply>
     <plus/>
     <cn type="integer"> 1 </cn>
     <apply>
      <divide/>
      <ci> mwaa8b93c8_308a_4d19_b732_22cb31f77620 </ci>
      <ci> mwc410441d_bd1d_4085_9e21_08b662e4f672 </ci>
     </apply>
    </apply>
    <apply>
     <plus/>
     <cn type="integer"> 1 </cn>
     <apply>
      <divide/>
      <ci> mw5b528d37_b514_46e5_a018_0173afdae714 </ci>
      <ci> mw8b39e257_03b8_4927_97c6_4b8444f70966 </ci>
     </apply>
    </apply>
   </apply>
  </apply>
  <cn type="integer"> 1 </cn>
</apply>
```

```
        </apply>
      </math>
    </kineticLaw>
  </reaction>
  <reaction id="mw8663fe1c_e3bb_4492_bf4d_3636ff027c3c" name="CC_6 alpha (fructose-1,6-bisphosphatase)" reversible="false" fast="false">
    <annotation>
      <COPASI xmlns="http://www.copasi.org/static/sbml">
        <rdf:RDF xmlns:dcterms="http://purl.org/dc/terms/" xmlns:rdf="http://www.w3.org/1999/02/22-rdf-syntax-ns#">
          <rdf:Description rdf:about="#COPASI54">
            <dcterms:created>
              <rdf:Description>
                <dcterms:W3CDTF>2010-04-14T16:52:19Z</dcterms:W3CDTF>
              </rdf:Description>
            </dcterms:created>
          </rdf:Description>
        </rdf:RDF>
      </COPASI>
    </annotation>
    <listOfReactants>
      <speciesReference species="mw2a09f89f_5936_4376_a2ab_4ff1bbbd4153"/>
    </listOfReactants>
    <listOfProducts>
      <speciesReference species="mwfcedd013_9e1c_4e21_9a5a_93ddde2ffc11"/>
      <speciesReference species="mw854ce178_2796_4118_8690_c13951a897c1"/>
    </listOfProducts>
    <kineticLaw>
      <math xmlns="http://www.w3.org/1998/Math/MathML">
        <apply>
          <divide/>
          <apply>
```

```
          <times/>
          <ci> mw69b6dfd1_e329_427b_a2e2_a5d668d300ca </ci>
          <ci> mw2a09f89f_5936_4376_a2ab_4ff1bbbd4153 </ci>
        </apply>
        <apply>
          <plus/>
          <ci> mw2a09f89f_5936_4376_a2ab_4ff1bbbd4153 </ci>
          <apply>
            <times/>
            <ci> mwceaf4424_f60b_4cab_8ae8_9826a4f7860c </ci>
            <apply>
              <plus/>
              <cn type="integer"> 1 </cn>
              <apply>
                <divide/>
                <ci> mwfcedd013_9e1c_4e21_9a5a_93ddde2ffc11 </ci>
                <ci> mwd48591b6_3ea8_4408_9e19_391e457c863d </ci>
              </apply>
              <apply>
                <divide/>
                <ci> mw854ce178_2796_4118_8690_c13951a897c1 </ci>
                <ci> mwf7770ee0_1efd_4f73_a3f0_925cae9eaca8 </ci>
              </apply>
            </apply>
          </apply>
        </apply>
      </apply>
    </math>
  </kineticLaw>
</reaction>
```

```
<reaction id="mw2e8a4120_e781_485a_b3ef_a9dbad603dbc" name="CC_7 (transketolase)"
fast="false">
 <annotation>
  <COPASI xmlns="http://www.copasi.org/static/sbml">
   <rdf:RDF xmlns:dcterms="http://purl.org/dc/terms/"
xmlns:rdf="http://www.w3.org/1999/02/22-rdf-syntax-ns#">
    <rdf:Description rdf:about="#COPASI55">
     <dcterms:created>
      <rdf:Description>
       <dcterms:W3CDTF>2010-04-15T18:06:55Z</dcterms:W3CDTF>
      </rdf:Description>
     </dcterms:created>
    </rdf:Description>
   </rdf:RDF>
  </COPASI>
 </annotation>
 <listOfReactants>
  <speciesReference species="mwfcedd013_9e1c_4e21_9a5a_93ddde2ffc11"/>
  <speciesReference species="mw5b528d37_b514_46e5_a018_0173afdae714"/>
  <speciesReference species="mweadaac0b_cbd7_4eb3_b892_fe6bf884cfc8"/>
  <speciesReference species="mw26816e75_d092_459e_bb3d_70aafa096e4d"/>
 </listOfReactants>
 <listOfProducts>
  <speciesReference species="mwaa8b93c8_308a_4d19_b732_22cb31f77620"/>
  <speciesReference species="mw7c70a014_6b69_42b3_9fbd_1d63cb722ae5"/>
  <speciesReference species="mweadaac0b_cbd7_4eb3_b892_fe6bf884cfc8"/>
  <speciesReference species="mw26816e75_d092_459e_bb3d_70aafa096e4d"/>
 </listOfProducts>
 <kineticLaw>
  <math xmlns="http://www.w3.org/1998/Math/MathML">
   <apply>
    <divide/>
```

<apply>
  <divide/>
  <apply>
    <times/>
    <ci> mw9e42a158_2ae8_49da_9a6e_635d6a018a2b </ci>
    <apply>
      <minus/>
      <apply>
        <times/>
        <ci> mwfcedd013_9e1c_4e21_9a5a_93ddde2ffc11 </ci>
        <ci> mw5b528d37_b514_46e5_a018_0173afdae714 </ci>
      </apply>
      <apply>
        <divide/>
        <apply>
          <times/>
          <ci> mw7c70a014_6b69_42b3_9fbd_1d63cb722ae5 </ci>
          <ci> mwaa8b93c8_308a_4d19_b732_22cb31f77620 </ci>
        </apply>
        <ci> mw97296fac_b8d0_4286_8c45_a7ae55aeab22 </ci>
      </apply>
    </apply>
  </apply>
  <apply>
    <times/>
    <ci> mw7e01e1f4_5a8e_40f5_805c_6bb66e4302d5 </ci>
    <ci> mwd82e6763_7382_481e_82d1_193b24b364f8 </ci>
  </apply>
</apply>
<apply>
  <times/>

```
<apply>
  <plus/>
  <cn type="integer"> 1 </cn>
  <apply>
    <divide/>
    <ci> mwfcedd013_9e1c_4e21_9a5a_93ddde2ffc11 </ci>
    <ci> mw7e01e1f4_5a8e_40f5_805c_6bb66e4302d5 </ci>
  </apply>
  <apply>
    <divide/>
    <ci> mwaa8b93c8_308a_4d19_b732_22cb31f77620 </ci>
    <ci> mw1940c877_4e88_4a71_957a_5160dc7cfd3c </ci>
  </apply>
</apply>
<apply>
  <plus/>
  <cn type="integer"> 1 </cn>
  <apply>
    <divide/>
    <ci> mw5b528d37_b514_46e5_a018_0173afdae714 </ci>
    <ci> mwd82e6763_7382_481e_82d1_193b24b364f8 </ci>
  </apply>
  <apply>
    <divide/>
    <ci> mw7c70a014_6b69_42b3_9fbd_1d63cb722ae5 </ci>
    <ci> mw328820c4_827f_4b41_ace1_19213258ac79 </ci>
  </apply>
</apply>
<apply>
  <plus/>
  <cn type="integer"> 1 </cn>
```

```
              <apply>
                <divide/>
                <ci> mweadaac0b_cbd7_4eb3_b892_fe6bf884cfc8 </ci>
                <ci> mw57be342f_c610_40fc_8742_cd5cac73cf4a </ci>
              </apply>
              <apply>
                <divide/>
                <ci> mw26816e75_d092_459e_bb3d_70aafa096e4d </ci>
                <ci> mw545ed108_3a36_4f08_880d_e713dae6443a </ci>
              </apply>
            </apply>
          </apply>
        </apply>
      </math>
    </kineticLaw>
  </reaction>
  <reaction id="mw9d0429c1_30f9_42d4_85bf_92d84f7fe540" name="CC_8 (aldolase)"
fast="false">
    <annotation>
      <COPASI xmlns="http://www.copasi.org/static/sbml">
        <rdf:RDF xmlns:dcterms="http://purl.org/dc/terms/"
xmlns:rdf="http://www.w3.org/1999/02/22-rdf-syntax-ns#">
          <rdf:Description rdf:about="#COPASI56">
            <dcterms:created>
              <rdf:Description>
                <dcterms:W3CDTF>2010-04-20T11:20:56Z</dcterms:W3CDTF>
              </rdf:Description>
            </dcterms:created>
          </rdf:Description>
        </rdf:RDF>
      </COPASI>
    </annotation>
```

```
<listOfReactants>
  <speciesReference species="mw8f67b710_8c1c_453f_a7ec_e9b6a55814fa"/>
  <speciesReference species="mwaa8b93c8_308a_4d19_b732_22cb31f77620"/>
  <speciesReference species="mw2a09f89f_5936_4376_a2ab_4ff1bbbd4153"/>
  <speciesReference species="mw5b528d37_b514_46e5_a018_0173afdae714"/>
</listOfReactants>
<listOfProducts>
  <speciesReference species="mwacf08e72_f001_4a26_962d_729f504b1ad0"/>
  <speciesReference species="mw2a09f89f_5936_4376_a2ab_4ff1bbbd4153"/>
  <speciesReference species="mw5b528d37_b514_46e5_a018_0173afdae714"/>
</listOfProducts>
<kineticLaw>
  <math xmlns="http://www.w3.org/1998/Math/MathML">
    <apply>
      <divide/>
      <apply>
        <divide/>
        <apply>
          <times/>
          <ci> mw2e164e0f_6a15_43c9_a62c_cc3a85074287 </ci>
          <apply>
            <minus/>
            <apply>
              <times/>
              <ci> mw8f67b710_8c1c_453f_a7ec_e9b6a55814fa </ci>
              <ci> mwaa8b93c8_308a_4d19_b732_22cb31f77620 </ci>
            </apply>
            <apply>
              <divide/>
              <ci> mwacf08e72_f001_4a26_962d_729f504b1ad0 </ci>
              <ci> mw9de2bbc0_b1db_4824_9105_762861f97d06 </ci>
```

</apply>
</apply>
</apply>
<apply>
<times/>
<ci> mw6e554aaa_4cb8_46a2_9af0_40c527042d95 </ci>
<ci> mw2ffe78db_96a4_4d1b_a62f_f84d7987e64e </ci>
</apply>
</apply>
<apply>
<minus/>
<apply>
<plus/>
<apply>
<times/>
<apply>
<plus/>
<cn type="integer"> 1 </cn>
<apply>
<divide/>
<ci> mw2a09f89f_5936_4376_a2ab_4ff1bbbd4153 </ci>
<ci> mwef61df7c_5b85_41cf_b539_58e829001c39 </ci>
</apply>
</apply>
<apply>
<plus/>
<cn type="integer"> 1 </cn>
<apply>
<divide/>
<ci> mwacf08e72_f001_4a26_962d_729f504b1ad0 </ci>
<ci> mw9d553771_b33e_40de_bbc5_22c5804e335a </ci>

</apply>
</apply>
</apply>
<apply>
<times/>
<apply>
<plus/>
<cn type="integer"> 1 </cn>
<apply>
<divide/>
<ci> mw8f67b710_8c1c_453f_a7ec_e9b6a55814fa </ci>
<ci> mw6e554aaa_4cb8_46a2_9af0_40c527042d95 </ci>
</apply>
</apply>
<apply>
<plus/>
<cn type="integer"> 1 </cn>
<apply>
<divide/>
<ci> mwaa8b93c8_308a_4d19_b732_22cb31f77620 </ci>
<ci> mw2ffe78db_96a4_4d1b_a62f_f84d7987e64e </ci>
</apply>
</apply>
<apply>
<plus/>
<cn type="integer"> 1 </cn>
<apply>
<divide/>
<ci> mw5b528d37_b514_46e5_a018_0173afdae714 </ci>
<ci> mwb4c2b40c_c637_4023_8d0f_5ed2bc9febf1 </ci>
</apply>

</apply>
</apply>
</apply>
<cn type="integer"> 1 </cn>
</apply>
</apply>
</math>
</kineticLaw>
</reaction>
<reaction id="mw9ebb86ff_7ac3_453d_ab63_0298b48a5207" name="CC_8 (aldolase) beta" fast="false">
<listOfReactants>
<speciesReference species="mw8f67b710_8c1c_453f_a7ec_e9b6a55814fa"/>
<speciesReference species="mwaa8b93c8_308a_4d19_b732_22cb31f77620"/>
<speciesReference species="mw2a09f89f_5936_4376_a2ab_4ff1bbbd4153"/>
<speciesReference species="mw5b528d37_b514_46e5_a018_0173afdae714"/>
</listOfReactants>
<listOfProducts>
<speciesReference species="mwacf08e72_f001_4a26_962d_729f504b1ad0"/>
<speciesReference species="mw2a09f89f_5936_4376_a2ab_4ff1bbbd4153"/>
<speciesReference species="mw5b528d37_b514_46e5_a018_0173afdae714"/>
</listOfProducts>
<kineticLaw>
<math xmlns="http://www.w3.org/1998/Math/MathML">
<apply>
<divide/>
<apply>
<divide/>
<apply>
<times/>
<ci> mwb84f53a1_12bb_45c4_bc48_32fddf334c72 </ci>

<apply>
<minus/>
<apply>
<times/>
<ci> mw8f67b710_8c1c_453f_a7ec_e9b6a55814fa </ci>
<ci> mwaa8b93c8_308a_4d19_b732_22cb31f77620 </ci>
</apply>
<apply>
<divide/>
<ci> mwacf08e72_f001_4a26_962d_729f504b1ad0 </ci>
<ci> mw9de2bbc0_b1db_4824_9105_762861f97d06 </ci>
</apply>
</apply>
</apply>
<apply>
<times/>
<ci> mwb15d9294_9509_4702_9083_ef51d8c235f9 </ci>
<ci> mwc410441d_bd1d_4085_9e21_08b662e4f672 </ci>
</apply>
</apply>
<apply>
<minus/>
<apply>
<plus/>
<apply>
<times/>
<apply>
<plus/>
<cn type="integer"> 1 </cn>
<apply>
<divide/>

```
      <ci> mw2a09f89f_5936_4376_a2ab_4ff1bbbd4153 </ci>
      <ci> mw1e28fdab_0576_4966_9927_17b091f2a4b2 </ci>
    </apply>
  </apply>
  <apply>
    <plus/>
    <cn type="integer"> 1 </cn>
    <apply>
      <divide/>
      <ci> mwacf08e72_f001_4a26_962d_729f504b1ad0 </ci>
      <ci> mw777c1f25_305f_42cf_a010_b537a05884dd </ci>
    </apply>
  </apply>
</apply>
<apply>
  <times/>
  <apply>
    <plus/>
    <cn type="integer"> 1 </cn>
    <apply>
      <divide/>
      <ci> mw8f67b710_8c1c_453f_a7ec_e9b6a55814fa </ci>
      <ci> mwb15d9294_9509_4702_9083_ef51d8c235f9 </ci>
    </apply>
  </apply>
  <apply>
    <plus/>
    <cn type="integer"> 1 </cn>
    <apply>
      <divide/>
      <ci> mwaa8b93c8_308a_4d19_b732_22cb31f77620 </ci>
```

```
              <ci> mwc410441d_bd1d_4085_9e21_08b662e4f672 </ci>
             </apply>
            </apply>
            <apply>
             <plus/>
             <cn type="integer"> 1 </cn>
             <apply>
              <divide/>
              <ci> mw5b528d37_b514_46e5_a018_0173afdae714 </ci>
              <ci> mw8b39e257_03b8_4927_97c6_4b8444f70966 </ci>
             </apply>
            </apply>
           </apply>
          </apply>
          <cn type="integer"> 1 </cn>
         </apply>
        </apply>
       </math>
      </kineticLaw>
     </reaction>
     <reaction id="mw0e677a89_165f_412a_976b_5f41a99b19cf" name="CC_9 (sedoheptulose-1,7
bisphosphatase)" reversible="false" fast="false">
      <annotation>
       <COPASI xmlns="http://www.copasi.org/static/sbml">
        <rdf:RDF xmlns:dcterms="http://purl.org/dc/terms/"
xmlns:rdf="http://www.w3.org/1999/02/22-rdf-syntax-ns#">
         <rdf:Description rdf:about="#COPASI57">
          <dcterms:created>
           <rdf:Description>
            <dcterms:W3CDTF>2010-04-21T02:31:25Z</dcterms:W3CDTF>
           </rdf:Description>
          </dcterms:created>
```

```
      </rdf:Description>
    </rdf:RDF>
  </COPASI>
</annotation>
<listOfReactants>
  <speciesReference species="mwacf08e72_f001_4a26_962d_729f504b1ad0"/>
  <speciesReference species="mw2a09f89f_5936_4376_a2ab_4ff1bbbd4153"/>
</listOfReactants>
<listOfProducts>
  <speciesReference species="mweadaac0b_cbd7_4eb3_b892_fe6bf884cfc8"/>
  <speciesReference species="mw2a09f89f_5936_4376_a2ab_4ff1bbbd4153"/>
</listOfProducts>
<kineticLaw>
  <math xmlns="http://www.w3.org/1998/Math/MathML">
    <apply>
      <divide/>
      <apply>
        <times/>
        <ci> mw676816c3_3653_49ac_9693_a838ec5f9446 </ci>
        <ci> mwacf08e72_f001_4a26_962d_729f504b1ad0 </ci>
      </apply>
      <apply>
        <plus/>
        <ci> mwacf08e72_f001_4a26_962d_729f504b1ad0 </ci>
        <apply>
          <times/>
          <ci> mw5fc73772_4633_4bf8_9aa6_fbabe153e1c8 </ci>
          <apply>
            <plus/>
            <cn type="integer"> 1 </cn>
            <apply>
```

```
              <divide/>
              <ci> mw2a09f89f_5936_4376_a2ab_4ff1bbbd4153 </ci>
              <ci> mwac1e88d0_dca5_49b3_9950_4e0560efa626 </ci>
             </apply>
            </apply>
           </apply>
          </apply>
         </apply>
        </math>
       </kineticLaw>
      </reaction>
      <reaction id="mw460ac88c_c88a_4fcb_8b3b_4e3d733edb89" name="CC_10 (transketolase)"
fast="false">
       <annotation>
        <COPASI xmlns="http://www.copasi.org/static/sbml">
         <rdf:RDF xmlns:dcterms="http://purl.org/dc/terms/"
xmlns:rdf="http://www.w3.org/1999/02/22-rdf-syntax-ns#">
          <rdf:Description rdf:about="#COPASI58">
           <dcterms:created>
            <rdf:Description>
             <dcterms:W3CDTF>2010-04-21T03:46:16Z</dcterms:W3CDTF>
            </rdf:Description>
           </dcterms:created>
          </rdf:Description>
         </rdf:RDF>
        </COPASI>
       </annotation>
       <listOfReactants>
        <speciesReference species="mweadaac0b_cbd7_4eb3_b892_fe6bf884cfc8"/>
        <speciesReference species="mw5b528d37_b514_46e5_a018_0173afdae714"/>
        <speciesReference species="mwfcedd013_9e1c_4e21_9a5a_93ddde2ffc11"/>
        <speciesReference species="mwaa8b93c8_308a_4d19_b732_22cb31f77620"/>
```

```
</listOfReactants>
<listOfProducts>
  <speciesReference species="mw26816e75_d092_459e_bb3d_70aafa096e4d"/>
  <speciesReference species="mw7c70a014_6b69_42b3_9fbd_1d63cb722ae5"/>
  <speciesReference species="mwfcedd013_9e1c_4e21_9a5a_93ddde2ffc11"/>
  <speciesReference species="mwaa8b93c8_308a_4d19_b732_22cb31f77620"/>
</listOfProducts>
<kineticLaw>
  <math xmlns="http://www.w3.org/1998/Math/MathML">
    <apply>
      <divide/>
      <apply>
        <divide/>
        <apply>
          <times/>
          <ci> mw509e62ff_43c2_439f_a219_323f6f557755 </ci>
          <apply>
            <minus/>
            <apply>
              <times/>
              <ci> mw5b528d37_b514_46e5_a018_0173afdae714 </ci>
              <ci> mweadaac0b_cbd7_4eb3_b892_fe6bf884cfc8 </ci>
            </apply>
            <apply>
              <divide/>
              <apply>
                <times/>
                <ci> mw26816e75_d092_459e_bb3d_70aafa096e4d </ci>
                <ci> mw7c70a014_6b69_42b3_9fbd_1d63cb722ae5 </ci>
              </apply>
              <ci> mw9d6196a1_62e0_4989_84af_dba7005fa377 </ci>
```

```
      </apply>
    </apply>
  </apply>
  <apply>
    <times/>
    <ci> mw57be342f_c610_40fc_8742_cd5cac73cf4a </ci>
    <ci> mwd82e6763_7382_481e_82d1_193b24b364f8 </ci>
  </apply>
</apply>
<apply>
  <times/>
  <apply>
    <plus/>
    <cn type="integer"> 1 </cn>
    <apply>
      <divide/>
      <ci> mwfcedd013_9e1c_4e21_9a5a_93ddde2ffc11 </ci>
      <ci> mw7e01e1f4_5a8e_40f5_805c_6bb66e4302d5 </ci>
    </apply>
    <apply>
      <divide/>
      <ci> mwaa8b93c8_308a_4d19_b732_22cb31f77620 </ci>
      <ci> mw1940c877_4e88_4a71_957a_5160dc7cfd3c </ci>
    </apply>
  </apply>
  <apply>
    <plus/>
    <cn type="integer"> 1 </cn>
    <apply>
      <divide/>
      <ci> mw5b528d37_b514_46e5_a018_0173afdae714 </ci>
```

<ci> mwd82e6763_7382_481e_82d1_193b24b364f8 </ci>

</apply>

<apply>

<divide/>

<ci> mw7c70a014_6b69_42b3_9fbd_1d63cb722ae5 </ci>

<ci> mw328820c4_827f_4b41_ace1_19213258ac79 </ci>

</apply>

</apply>

<apply>

<plus/>

<cn type="integer"> 1 </cn>

<apply>

<divide/>

<ci> mweadaac0b_cbd7_4eb3_b892_fe6bf884cfc8 </ci>

<ci> mw57be342f_c610_40fc_8742_cd5cac73cf4a </ci>

</apply>

<apply>

<divide/>

<ci> mw26816e75_d092_459e_bb3d_70aafa096e4d </ci>

<ci> mw545ed108_3a36_4f08_880d_e713dae6443a </ci>

</apply>

</apply>

</apply>

</apply>

</math>

</kineticLaw>

</reaction>

<reaction id="mw3ce0371b_d7fd_4f87_8028_792f40e8ed2f" name="C_11 (phosphopentose isomerase)" fast="false">

<annotation>

<COPASI xmlns="http://www.copasi.org/static/sbml">

```
            <rdf:RDF xmlns:dcterms="http://purl.org/dc/terms/"
xmlns:rdf="http://www.w3.org/1999/02/22-rdf-syntax-ns#">
              <rdf:Description rdf:about="#COPASI59">
                <dcterms:created>
                  <rdf:Description>
                    <dcterms:W3CDTF>2010-04-23T03:43:39Z</dcterms:W3CDTF>
                  </rdf:Description>
                </dcterms:created>
              </rdf:Description>
            </rdf:RDF>
          </COPASI>
        </annotation>
        <listOfReactants>
          <speciesReference species="mw26816e75_d092_459e_bb3d_70aafa096e4d"/>
        </listOfReactants>
        <listOfProducts>
          <speciesReference species="mw1d7eb0d8_a048_4013_9bbd_c347637cc6b9"/>
        </listOfProducts>
        <kineticLaw>
          <math xmlns="http://www.w3.org/1998/Math/MathML">
            <apply>
              <divide/>
              <apply>
                <divide/>
                <apply>
                  <times/>
                  <ci> mwf878e33d_3228_40a3_8fa6_9865294de7a1 </ci>
                  <apply>
                    <minus/>
                    <ci> mw26816e75_d092_459e_bb3d_70aafa096e4d </ci>
                    <apply>
```

<divide/>

<ci> mw1d7eb0d8_a048_4013_9bbd_c347637cc6b9 </ci>

<ci> mwa92c6c71_d522_4d07_9ef1_f6f2c17f9c71 </ci>

</apply>

</apply>

</apply>

<ci> mwc31a14d7_9c22_4389_99c6_58d27312cdb7 </ci>

</apply>

<apply>

<plus/>

<cn type="integer"> 1 </cn>

<apply>

<divide/>

<ci> mw1d7eb0d8_a048_4013_9bbd_c347637cc6b9 </ci>

<ci> mwd908059f_c845_4440_addd_e491a150ff80 </ci>

</apply>

<apply>

<divide/>

<ci> mw26816e75_d092_459e_bb3d_70aafa096e4d </ci>

<ci> mwc31a14d7_9c22_4389_99c6_58d27312cdb7 </ci>

</apply>

</apply>

</apply>

</math>

</kineticLaw>

</reaction>

<reaction id="mw4e4ee494_854b_42e2_9c70_e64e65b3096e" name="C_12 (phosphopentose epimerase)" fast="false">

<annotation>

<COPASI xmlns="http://www.copasi.org/static/sbml">

<rdf:RDF xmlns:dcterms="http://purl.org/dc/terms/" xmlns:rdf="http://www.w3.org/1999/02/22-rdf-syntax-ns#">

```
      <rdf:Description rdf:about="#COPASI60">
        <dcterms:created>
          <rdf:Description>
            <dcterms:W3CDTF>2010-04-23T03:43:48Z</dcterms:W3CDTF>
          </rdf:Description>
        </dcterms:created>
      </rdf:Description>
    </rdf:RDF>
  </COPASI>
</annotation>
<listOfReactants>
  <speciesReference species="mw7c70a014_6b69_42b3_9fbd_1d63cb722ae5"/>
</listOfReactants>
<listOfProducts>
  <speciesReference species="mw1d7eb0d8_a048_4013_9bbd_c347637cc6b9"/>
</listOfProducts>
<kineticLaw>
  <math xmlns="http://www.w3.org/1998/Math/MathML">
    <apply>
      <divide/>
      <apply>
        <divide/>
        <apply>
          <times/>
          <ci> mw8f309108_f60b_445c_af16_065b0479e746 </ci>
          <apply>
            <minus/>
            <ci> mw7c70a014_6b69_42b3_9fbd_1d63cb722ae5 </ci>
            <apply>
              <divide/>
              <ci> mw1d7eb0d8_a048_4013_9bbd_c347637cc6b9 </ci>
```

<ci> mw93964b11_67c1_4727_84cc_949cd4f6ecf4 </ci>

</apply>

</apply>

</apply>

<ci> mw9a22bae8_8f9c_4a94_a7ab_127fa8a4d3bd </ci>

</apply>

<apply>

<plus/>

<cn type="integer"> 1 </cn>

<apply>

<divide/>

<ci> mw1d7eb0d8_a048_4013_9bbd_c347637cc6b9 </ci>

<ci> mw1c952255_2606_4fe2_9e07_47ea2266367e </ci>

</apply>

<apply>

<divide/>

<ci> mw7c70a014_6b69_42b3_9fbd_1d63cb722ae5 </ci>

<ci> mw9a22bae8_8f9c_4a94_a7ab_127fa8a4d3bd </ci>

</apply>

</apply>

</apply>

</math>

</kineticLaw>

</reaction>

<reaction id="mw29b77153_fb0e_4f57_ac04_df8afa1d9e23" name="C_13 (phosphoribulokinase)" reversible="false" fast="false">

<annotation>

<COPASI xmlns="http://www.copasi.org/static/sbml">

<rdf:RDF xmlns:dcterms="http://purl.org/dc/terms/" xmlns:rdf="http://www.w3.org/1999/02/22-rdf-syntax-ns#">

<rdf:Description rdf:about="#COPASI61">

<dcterms:created>

```
        <rdf:Description>
          <dcterms:W3CDTF>2010-04-23T03:48:04Z</dcterms:W3CDTF>
        </rdf:Description>
      </dcterms:created>
    </rdf:Description>
  </rdf:RDF>
 </COPASI>
</annotation>
<listOfReactants>
 <speciesReference species="mw1d7eb0d8_a048_4013_9bbd_c347637cc6b9"/>
 <speciesReference species="mw8e2cc29c_c73e_4258_b3ba_2356620bb64e"/>
 <speciesReference species="mw854ce178_2796_4118_8690_c13951a897c1"/>
 <speciesReference species="mw372b8247_b11e_4d9b_be9b_e742fd2d3853"/>
</listOfReactants>
<listOfProducts>
 <speciesReference species="mw98fecd51_ba08_4727_98ec_04637a47c5ac"/>
 <speciesReference species="mwe8d1ae1f_35f9_45fd_bedf_7dbeef3972e2"/>
 <speciesReference species="mw854ce178_2796_4118_8690_c13951a897c1"/>
 <speciesReference species="mw372b8247_b11e_4d9b_be9b_e742fd2d3853"/>
</listOfProducts>
<kineticLaw>
 <math xmlns="http://www.w3.org/1998/Math/MathML">
  <apply>
   <divide/>
   <apply>
    <times/>
    <ci> mw6ab10457_9720_4445_a710_232dd2c4547d </ci>
    <ci> mw8e2cc29c_c73e_4258_b3ba_2356620bb64e </ci>
    <ci> mw1d7eb0d8_a048_4013_9bbd_c347637cc6b9 </ci>
   </apply>
   <apply>
```

```
<times/>
<apply>
  <plus/>
  <apply>
    <times/>
    <ci> mw8e2cc29c_c73e_4258_b3ba_2356620bb64e </ci>
    <apply>
      <plus/>
      <cn type="integer"> 1 </cn>
      <apply>
        <divide/>
        <ci> mwe8d1ae1f_35f9_45fd_bedf_7dbeef3972e2 </ci>
        <ci> mw3ca15696_2c16_4bbe_9acc_5bb19882a03e </ci>
      </apply>
    </apply>
  </apply>
  <apply>
    <times/>
    <ci> mwf6529569_4b56_44fa_993e_f2741a368c08 </ci>
    <apply>
      <plus/>
      <cn type="integer"> 1 </cn>
      <apply>
        <divide/>
        <ci> mwe8d1ae1f_35f9_45fd_bedf_7dbeef3972e2 </ci>
        <ci> mw459ad12f_127a_4f15_a4ca_dd8d9d3ca505 </ci>
      </apply>
    </apply>
  </apply>
</apply>
<apply>
```

```
            <plus/>
            <ci> mw1d7eb0d8_a048_4013_9bbd_c347637cc6b9 </ci>
            <apply>
              <times/>
              <ci> mwa97791f8_5c29_40fd_8db4_b61695f0810e </ci>
              <apply>
                <plus/>
                <cn type="integer"> 1 </cn>
                <apply>
                  <divide/>
                  <ci> mw372b8247_b11e_4d9b_be9b_e742fd2d3853 </ci>
                  <ci> mw620d20e1_7007_4d40_9ee4_67bc35938f33 </ci>
                </apply>
                <apply>
                  <divide/>
                  <ci> mw98fecd51_ba08_4727_98ec_04637a47c5ac </ci>
                  <ci> mwa3e0f729_e97d_443e_a14a_9eb9891474e1 </ci>
                </apply>
                <apply>
                  <divide/>
                  <ci> mw854ce178_2796_4118_8690_c13951a897c1 </ci>
                  <ci> mw5011fe71_c328_4107_9245_0861c6dbfb16 </ci>
                </apply>
              </apply>
            </apply>
          </apply>
        </apply>
      </apply>
    </math>
  </kineticLaw>
</reaction>
```

<reaction id="mwae845a07_5d44_43f8_af3a_2db37714cf6a" name="GPI (glucosephosphate isomerase)" fast="false">
<annotation>
<COPASI xmlns="http://www.copasi.org/static/sbml">
<rdf:RDF xmlns:dcterms="http://purl.org/dc/terms/" xmlns:rdf="http://www.w3.org/1999/02/22-rdf-syntax-ns#">
<rdf:Description rdf:about="#COPASI62">
<dcterms:created>
<rdf:Description>
<dcterms:W3CDTF>2010-04-29T13:08:18Z</dcterms:W3CDTF>
</rdf:Description>
</dcterms:created>
</rdf:Description>
</rdf:RDF>
</COPASI>
</annotation>
<listOfReactants>
<speciesReference species="mwfcedd013_9e1c_4e21_9a5a_93ddde2ffc11"/>
</listOfReactants>
<listOfProducts>
<speciesReference species="mw3a6830cd_1b04_49df_b4fe_fc7a6362142a"/>
</listOfProducts>
<kineticLaw>
<math xmlns="http://www.w3.org/1998/Math/MathML">
<apply>
<divide/>
<apply>
<divide/>
<apply>
<times/>
<ci> mwcb0e41ed_453b_4010_8628_3c3dc91f2591 </ci>
<apply>

```
          <minus/>
          <ci> mwfcedd013_9e1c_4e21_9a5a_93ddde2ffc11 </ci>
          <apply>
            <divide/>
            <ci> mw3a6830cd_1b04_49df_b4fe_fc7a6362142a </ci>
            <ci> mwcff4a43c_2777_4532_a542_a418d54de341 </ci>
          </apply>
        </apply>
      </apply>
      <ci> mw467513d1_2f1d_4bc8_b5d4_2707b9c5db6a </ci>
    </apply>
    <apply>
      <plus/>
      <apply>
        <divide/>
        <apply>
          <times/>
          <cn type="integer"> 1 </cn>
          <ci> mwfcedd013_9e1c_4e21_9a5a_93ddde2ffc11 </ci>
        </apply>
        <ci> mw467513d1_2f1d_4bc8_b5d4_2707b9c5db6a </ci>
      </apply>
      <apply>
        <divide/>
        <ci> mw3a6830cd_1b04_49df_b4fe_fc7a6362142a </ci>
        <ci> mwca8937d4_62a3_442e_bcb6_13c0d0166036 </ci>
      </apply>
    </apply>
  </apply>
</math>
</kineticLaw>
```

```
    </reaction>
    <reaction id="mw8637137f_b4ad_4198_87ed_0ac3047a2575" name="OPP_1 (glucose-6-
phosphate dehydrogenase)" reversible="false" fast="false">
      <listOfReactants>
        <speciesReference species="mw3a6830cd_1b04_49df_b4fe_fc7a6362142a"/>
      </listOfReactants>
      <listOfProducts>
        <speciesReference species="mwb8027448_5c51_43ad_9f13_cd37b5418a54"/>
      </listOfProducts>
      <kineticLaw>
        <math xmlns="http://www.w3.org/1998/Math/MathML">
          <apply>
            <divide/>
            <apply>
              <times/>
              <ci> mw14fbc3da_1eb6_4470_8032_06f6d5ce7c93 </ci>
              <ci> mw3a6830cd_1b04_49df_b4fe_fc7a6362142a </ci>
            </apply>
            <apply>
              <times/>
              <ci> mwb23b6c01_848a_44fb_8324_dda82ffcb0c4 </ci>
              <apply>
                <plus/>
                <cn type="integer"> 1 </cn>
                <apply>
                  <divide/>
                  <ci> mw3a6830cd_1b04_49df_b4fe_fc7a6362142a </ci>
                  <ci> mwb23b6c01_848a_44fb_8324_dda82ffcb0c4 </ci>
                </apply>
                <apply>
                  <divide/>
```

<ci> mwb8027448_5c51_43ad_9f13_cd37b5418a54 </ci>

<ci> mwa4a1fe58_2c6a_4f99_8001_4a0fedc9a357 </ci>

</apply>

</apply>

</apply>

</apply>

</math>

</kineticLaw>

</reaction>

<reaction id="mw5cdf9207_718d_4ab2_b636_980c19dc923f" name="OPP_2 (phosphogluconate dehydrogenase)" reversible="false" fast="false">

<listOfReactants>

<speciesReference species="mwb8027448_5c51_43ad_9f13_cd37b5418a54"/>

<speciesReference species="mw7c834bb1_1865_4950_aa20_f3eceb92b21d"/>

</listOfReactants>

<listOfProducts>

<speciesReference species="mw1d7eb0d8_a048_4013_9bbd_c347637cc6b9"/>

<speciesReference species="mwd2e6e004_23f5_4cd8_81e0_8385021ec904"/>

</listOfProducts>

<kineticLaw>

<math xmlns="http://www.w3.org/1998/Math/MathML">

<apply>

<divide/>

<apply>

<times/>

<ci> mw376c4606_38fa_4a60_9b24_8ef462cbe2ff </ci>

<ci> mwb8027448_5c51_43ad_9f13_cd37b5418a54 </ci>

<ci> mw7c834bb1_1865_4950_aa20_f3eceb92b21d </ci>

</apply>

<apply>

<times/>

<apply>

<plus/>

<ci> mwb8027448_5c51_43ad_9f13_cd37b5418a54 </ci>

<ci> mw9e08e044_96ba_486f_9778_f5a4ea04cff4 </ci>

</apply>

<apply>

<plus/>

<ci> mw7c834bb1_1865_4950_aa20_f3eceb92b21d </ci>

<ci> mwf24759b0_36bc_4a60_b869_301f6c1f2d80 </ci>

</apply>

</apply>

</apply>

</math>

</kineticLaw>

</reaction>

<reaction id="mw0c7ccba5_ecc0_435b_be03_9c22d2e48b42" name="GSM_1 (simplified phosphoserine transaminase)" reversible="false" fast="false">

<annotation>

<COPASI xmlns="http://www.copasi.org/static/sbml">

<rdf:RDF xmlns:dcterms="http://purl.org/dc/terms/" xmlns:rdf="http://www.w3.org/1999/02/22-rdf-syntax-ns#">

<rdf:Description rdf:about="#COPASI81">

<dcterms:created>

<rdf:Description>

<dcterms:W3CDTF>2011-06-15T16:11:22Z</dcterms:W3CDTF>

</rdf:Description>

</dcterms:created>

</rdf:Description>

</rdf:RDF>

</COPASI>

</annotation>

<listOfReactants>

```
        &lt;speciesReference species="mw372b8247_b11e_4d9b_be9b_e742fd2d3853"/&gt;
      &lt;/listOfReactants&gt;
      &lt;listOfProducts&gt;
        &lt;speciesReference species="mw7bb27e61_6440_4c3b_af0a_6c1ecd36ff29"/&gt;
      &lt;/listOfProducts&gt;
      &lt;kineticLaw&gt;
        &lt;math xmlns="http://www.w3.org/1998/Math/MathML"&gt;
          &lt;apply&gt;
            &lt;divide/&gt;
            &lt;apply&gt;
              &lt;times/&gt;
              &lt;ci&gt; mw02f9621e_b863_4168_a11d_fa2753817db7 &lt;/ci&gt;
              &lt;ci&gt; mw372b8247_b11e_4d9b_be9b_e742fd2d3853 &lt;/ci&gt;
            &lt;/apply&gt;
            &lt;apply&gt;
              &lt;plus/&gt;
              &lt;ci&gt; mwdd6e9231_51b9_4b67_b71c_24c2e4af56ba &lt;/ci&gt;
              &lt;ci&gt; mw372b8247_b11e_4d9b_be9b_e742fd2d3853 &lt;/ci&gt;
            &lt;/apply&gt;
          &lt;/apply&gt;
        &lt;/math&gt;
      &lt;/kineticLaw&gt;
    &lt;/reaction&gt;
    <reaction id="mwb19ebd38_ca9d_48eb_ab86_c557ab2570e8" name="GSM_2 (glycine
transaminase)" reversible="false" fast="false">
      &lt;listOfReactants&gt;
        &lt;speciesReference species="mwb970288f_4109_4c97_90b7_cc60512dc613"/&gt;
      &lt;/listOfReactants&gt;
      &lt;listOfProducts&gt;
        &lt;speciesReference species="mw3d986236_5c95_4128_9ee4_95d616f64d6f"/&gt;
      &lt;/listOfProducts&gt;
```

```
        <kineticLaw>
          <math xmlns="http://www.w3.org/1998/Math/MathML">
            <apply>
              <divide/>
              <apply>
                <times/>
                <ci> mw8120d5c3_18c9_485d_99c5_bdf56d90f8c2 </ci>
                <ci> mwb970288f_4109_4c97_90b7_cc60512dc613 </ci>
              </apply>
              <apply>
                <plus/>
                <ci> mwee200f01_e65f_45cf_b143_f20ca0b735f9 </ci>
                <ci> mwb970288f_4109_4c97_90b7_cc60512dc613 </ci>
              </apply>
            </apply>
          </math>
        </kineticLaw>
      </reaction>
      <reaction id="mw341c1f65_9b3f_4667_a8e1_8ca7716a95c8" name="PP_1 (RuBisCO)" reversible="false" fast="false">
        <listOfReactants>
          <speciesReference species="mw98fecd51_ba08_4727_98ec_04637a47c5ac"/>
          <speciesReference species="mwf3d533fa_7176_4543_a1c4_f46f4dc4f8f6"/>
          <speciesReference species="mw739c8f22_6a61_4dca_8cfe_3c962adb4128"/>
          <speciesReference species="mw372b8247_b11e_4d9b_be9b_e742fd2d3853"/>
          <speciesReference species="mw2a09f89f_5936_4376_a2ab_4ff1bbbd4153"/>
          <speciesReference species="mwacf08e72_f001_4a26_962d_729f504b1ad0"/>
          <speciesReference species="mw854ce178_2796_4118_8690_c13951a897c1"/>
          <speciesReference species="mwd2e6e004_23f5_4cd8_81e0_8385021ec904"/>
        </listOfReactants>
        <listOfProducts>
```

```
 <speciesReference species="mw04176f80_f815_4c8f_a0d2_7625e7c0868b"/>
 <speciesReference species="mw372b8247_b11e_4d9b_be9b_e742fd2d3853"/>
 <speciesReference species="mw739c8f22_6a61_4dca_8cfe_3c962adb4128"/>
 <speciesReference species="mw2a09f89f_5936_4376_a2ab_4ff1bbbd4153"/>
 <speciesReference species="mwacf08e72_f001_4a26_962d_729f504b1ad0"/>
 <speciesReference species="mw854ce178_2796_4118_8690_c13951a897c1"/>
 <speciesReference species="mwd2e6e004_23f5_4cd8_81e0_8385021ec904"/>
</listOfProducts>
<kineticLaw>
 <math xmlns="http://www.w3.org/1998/Math/MathML">
  <apply>
   <divide/>
   <apply>
    <times/>
    <ci> mw98fecd51_ba08_4727_98ec_04637a47c5ac </ci>
    <apply>
     <divide/>
     <apply>
      <times/>
      <ci> mw70d358f1_f93f_432d_a6a3_d1aba42c9db7 </ci>
      <ci> mwf3d533fa_7176_4543_a1c4_f46f4dc4f8f6 </ci>
     </apply>
     <apply>
      <plus/>
      <ci> mwf3d533fa_7176_4543_a1c4_f46f4dc4f8f6 </ci>
      <apply>
       <times/>
       <ci> mw364ec325_c2af_4413_8934_e8b8af09f5f4 </ci>
       <apply>
        <plus/>
        <cn type="integer"> 1 </cn>
```

<apply>
<divide/>
<ci> mw739c8f22_6a61_4dca_8cfe_3c962adb4128 </ci>
<ci> mwe3972b9a_3c7e_4eb9_aa83_6bc48e5251c9 </ci>
</apply>
</apply>
</apply>
</apply>
</apply>
</apply>
<apply>
<plus/>
<ci> mw98fecd51_ba08_4727_98ec_04637a47c5ac </ci>
<apply>
<times/>
<ci> mw1aa79476_50cf_4ad8_b34b_1bd559d0e8b7 </ci>
<apply>
<plus/>
<cn type="integer"> 1 </cn>
<apply>
<divide/>
<ci> mw372b8247_b11e_4d9b_be9b_e742fd2d3853 </ci>
<ci> mw25c38b1f_a0cf_41ea_a0b9_ce591ec4bb41 </ci>
</apply>
<apply>
<divide/>
<ci> mw2a09f89f_5936_4376_a2ab_4ff1bbbd4153 </ci>
<ci> mwb9ff1179_1b4d_4f43_a08e_07a71dc0e53a </ci>
</apply>
<apply>
<divide/>

```
<ci> mwacf08e72_f001_4a26_962d_729f504b1ad0 </ci>
<ci> mwb765042e_909e_4002_adec_27be965841a3 </ci>
</apply>
<apply>
<divide/>
<ci> mw854ce178_2796_4118_8690_c13951a897c1 </ci>
<ci> mwef7f5344_1be5_414e_a10b_43c1f1ba59bb </ci>
</apply>
<apply>
<divide/>
<ci> mwd2e6e004_23f5_4cd8_81e0_8385021ec904 </ci>
<ci> mwaa92dfc9_9a33_4094_91a8_216d11908ae3 </ci>
</apply>
</apply>
</apply>
</apply>
</apply>
</math>
</kineticLaw>
</reaction>
<reaction id="mw16acbefd_0bfd_415e_ba36_7974b1b0919d" name="PP_2a (phosphoglycolate phosphatase)" reversible="false" fast="false">
<annotation>
<COPASI xmlns="http://www.copasi.org/static/sbml">
<rdf:RDF xmlns:dcterms="http://purl.org/dc/terms/" xmlns:rdf="http://www.w3.org/1999/02/22-rdf-syntax-ns#">
<rdf:Description rdf:about="#COPASI70">
<dcterms:created>
<rdf:Description>
<dcterms:W3CDTF>2011-05-09T23:56:05Z</dcterms:W3CDTF>
</rdf:Description>
</dcterms:created>
```

```
      </rdf:Description>
    </rdf:RDF>
  </COPASI>
</annotation>
<listOfReactants>
  <speciesReference species="mw04176f80_f815_4c8f_a0d2_7625e7c0868b"/>
</listOfReactants>
<listOfProducts>
  <speciesReference species="mwd946c4b9_b1d9_4b6d_9616_2f03e37dc588"/>
  <speciesReference species="mw854ce178_2796_4118_8690_c13951a897c1"/>
</listOfProducts>
<kineticLaw>
  <math xmlns="http://www.w3.org/1998/Math/MathML">
    <apply>
      <divide/>
      <apply>
        <times/>
        <ci> mw45b50366_2e2e_4340_a159_d5255fbb5599 </ci>
        <ci> mw04176f80_f815_4c8f_a0d2_7625e7c0868b </ci>
      </apply>
      <apply>
        <plus/>
        <ci> mw04176f80_f815_4c8f_a0d2_7625e7c0868b </ci>
        <apply>
          <times/>
          <ci> mw2a28fd49_4cc1_45af_b6c5_f28199557faa </ci>
          <apply>
            <plus/>
            <cn type="integer"> 1 </cn>
            <apply>
              <divide/>
```

```
            <ci> mwd946c4b9_b1d9_4b6d_9616_2f03e37dc588 </ci>
            <ci> mw9415f414_35fe_45d1_a3c7_d7c0b56ef439 </ci>
           </apply>
          </apply>
          <apply>
           <plus/>
           <cn type="integer"> 1 </cn>
           <apply>
            <divide/>
            <ci> mw854ce178_2796_4118_8690_c13951a897c1 </ci>
            <ci> mwe0680e97_a6e6_4cca_94b1_395c1f98c3b8 </ci>
           </apply>
          </apply>
         </apply>
        </apply>
       </apply>
      </math>
     </kineticLaw>
    </reaction>
    <reaction id="mw01b5e11e_55fb_44a3_80b6_83165395d922" name="PP_2b (phosphoglycolate
phosphatase)" reversible="false" fast="false">
     <listOfReactants>
      <speciesReference species="mw04176f80_f815_4c8f_a0d2_7625e7c0868b"/>
     </listOfReactants>
     <listOfProducts>
      <speciesReference species="mwd946c4b9_b1d9_4b6d_9616_2f03e37dc588"/>
      <speciesReference species="mw854ce178_2796_4118_8690_c13951a897c1"/>
     </listOfProducts>
     <kineticLaw>
      <math xmlns="http://www.w3.org/1998/Math/MathML">
       <apply>
```

```
<divide/>
<apply>
 <times/>
 <ci> mw08c7da94_cfbc_4e2b_aad0_861e59694355 </ci>
 <ci> mw04176f80_f815_4c8f_a0d2_7625e7c0868b </ci>
</apply>
<apply>
 <plus/>
 <ci> mw04176f80_f815_4c8f_a0d2_7625e7c0868b </ci>
 <apply>
  <times/>
  <ci> mw8cce0768_8b1e_4e92_a94e_f02e7edd81a9 </ci>
  <apply>
   <plus/>
   <cn type="integer"> 1 </cn>
   <apply>
    <divide/>
    <ci> mwd946c4b9_b1d9_4b6d_9616_2f03e37dc588 </ci>
    <ci> mw9415f414_35fe_45d1_a3c7_d7c0b56ef439 </ci>
   </apply>
  </apply>
  <apply>
   <plus/>
   <cn type="integer"> 1 </cn>
   <apply>
    <divide/>
    <ci> mw854ce178_2796_4118_8690_c13951a897c1 </ci>
    <ci> mwe0680e97_a6e6_4cca_94b1_395c1f98c3b8 </ci>
   </apply>
  </apply>
 </apply>
```

</apply>
</apply>
</math>
</kineticLaw>
</reaction>
<reaction id="mw68c00573_2e0a_4b32_998d_3c6b692e6579" name="PP_2c (phosphoglycolate phosphatase)" reversible="false" fast="false">
<listOfReactants>
<speciesReference species="mw04176f80_f815_4c8f_a0d2_7625e7c0868b"/>
</listOfReactants>
<listOfProducts>
<speciesReference species="mwd946c4b9_b1d9_4b6d_9616_2f03e37dc588"/>
<speciesReference species="mw854ce178_2796_4118_8690_c13951a897c1"/>
</listOfProducts>
<kineticLaw>
<math xmlns="http://www.w3.org/1998/Math/MathML">
<apply>
<divide/>
<apply>
<times/>
<ci> mw273ec436_6071_4799_870d_095c01364042 </ci>
<ci> mw04176f80_f815_4c8f_a0d2_7625e7c0868b </ci>
</apply>
<apply>
<plus/>
<ci> mw04176f80_f815_4c8f_a0d2_7625e7c0868b </ci>
<apply>
<times/>
<ci> mwe7cd87c9_87ad_458d_b881_b18d32ab3919 </ci>
<apply>
<plus/>

```
          <cn type="integer"> 1 </cn>
          <apply>
            <divide/>
            <ci> mwd946c4b9_b1d9_4b6d_9616_2f03e37dc588 </ci>
            <ci> mw9415f414_35fe_45d1_a3c7_d7c0b56ef439 </ci>
          </apply>
        </apply>
        <apply>
          <plus/>
          <cn type="integer"> 1 </cn>
          <apply>
            <divide/>
            <ci> mw854ce178_2796_4118_8690_c13951a897c1 </ci>
            <ci> mwe0680e97_a6e6_4cca_94b1_395c1f98c3b8 </ci>
          </apply>
        </apply>
      </apply>
    </apply>
  </apply>
</math>
</kineticLaw>
</reaction>
<reaction id="mw6e824a2f_599d_4d13_9df6_9ddfdde0ef99" name="PP_3 (glycolate oxidase)"
reversible="false" fast="false">
 <annotation>
  <COPASI xmlns="http://www.copasi.org/static/sbml">
   <rdf:RDF xmlns:dcterms="http://purl.org/dc/terms/"
xmlns:rdf="http://www.w3.org/1999/02/22-rdf-syntax-ns#">
    <rdf:Description rdf:about="#COPASI71">
     <dcterms:created>
      <rdf:Description>
       <dcterms:W3CDTF>2011-05-10T00:12:50Z</dcterms:W3CDTF>
```

```
          </rdf:Description>
        </dcterms:created>
      </rdf:Description>
    </rdf:RDF>
  </COPASI>
</annotation>
<listOfReactants>
  <speciesReference species="mwd946c4b9_b1d9_4b6d_9616_2f03e37dc588"/>
</listOfReactants>
<listOfProducts>
  <speciesReference species="mwb970288f_4109_4c97_90b7_cc60512dc613"/>
</listOfProducts>
<kineticLaw>
  <math xmlns="http://www.w3.org/1998/Math/MathML">
    <apply>
      <divide/>
      <apply>
        <times/>
        <ci> mw83c2564e_fce9_4d65_ad89_f4ec9d59e973 </ci>
        <ci> mwd946c4b9_b1d9_4b6d_9616_2f03e37dc588 </ci>
      </apply>
      <apply>
        <plus/>
        <ci> mwd946c4b9_b1d9_4b6d_9616_2f03e37dc588 </ci>
        <ci> mwfc079b4e_b465_4bbe_b1ae_11a68246902a </ci>
      </apply>
    </apply>
  </math>
</kineticLaw>
</reaction>
```

```
<reaction id="mwd6edecc4_529a_4a13_8618_e35957bfd4f4" name="PP_4 (serineglyoxylate
transaminase)" fast="false">
  &lt;annotation&gt;
    &lt;COPASI xmlns="http://www.copasi.org/static/sbml"&gt;
      <rdf:RDF xmlns:dcterms="http://purl.org/dc/terms/"
xmlns:rdf="http://www.w3.org/1999/02/22-rdf-syntax-ns#">
        &lt;rdf:Description rdf:about="#COPASI72"&gt;
          &lt;dcterms:created&gt;
            &lt;rdf:Description&gt;
              &lt;dcterms:W3CDTF&gt;2011-05-10T00:16:51Z&lt;/dcterms:W3CDTF&gt;
            &lt;/rdf:Description&gt;
          &lt;/dcterms:created&gt;
        &lt;/rdf:Description&gt;
      &lt;/rdf:RDF&gt;
    &lt;/COPASI&gt;
  &lt;/annotation&gt;
  &lt;listOfReactants&gt;
    &lt;speciesReference species="mwb970288f_4109_4c97_90b7_cc60512dc613"/&gt;
    &lt;speciesReference species="mw7bb27e61_6440_4c3b_af0a_6c1ecd36ff29"/&gt;
  &lt;/listOfReactants&gt;
  &lt;listOfProducts&gt;
    &lt;speciesReference species="mw21f7bed2_a8e0_4d16_a4a4_8718de3f1c6a"/&gt;
    &lt;speciesReference species="mw3d986236_5c95_4128_9ee4_95d616f64d6f"/&gt;
  &lt;/listOfProducts&gt;
  &lt;kineticLaw&gt;
    &lt;math xmlns="http://www.w3.org/1998/Math/MathML"&gt;
      &lt;apply&gt;
        &lt;divide/&gt;
        &lt;apply&gt;
          &lt;times/&gt;
          &lt;ci&gt; mw20d5178d_9286_49d1_8c12_d413a39a7d7a &lt;/ci&gt;
          &lt;apply&gt;
```

<minus/>
<apply>
<times/>
<ci> mwb970288f_4109_4c97_90b7_cc60512dc613 </ci>
<ci> mw7bb27e61_6440_4c3b_af0a_6c1ecd36ff29 </ci>
</apply>
<apply>
<divide/>
<apply>
<times/>
<ci> mw21f7bed2_a8e0_4d16_a4a4_8718de3f1c6a </ci>
<ci> mw3d986236_5c95_4128_9ee4_95d616f64d6f </ci>
</apply>
<ci> mw4c0a5f49_cf9c_4137_a4d1_a27a9c633c60 </ci>
</apply>
</apply>
</apply>
<apply>
<times/>
<apply>
<plus/>
<ci> mwb970288f_4109_4c97_90b7_cc60512dc613 </ci>
<ci> mw0bdbb111_5454_4f47_9918_8da9990953f9 </ci>
</apply>
<apply>
<plus/>
<ci> mw7bb27e61_6440_4c3b_af0a_6c1ecd36ff29 </ci>
<apply>
<times/>
<ci> mw14d17aaf_3016_4fb0_b90c_6b70ea2eeb64 </ci>
<apply>

<plus/>

<cn type="integer"> 1 </cn>

<apply>

<divide/>

<ci> mw3d986236_5c95_4128_9ee4_95d616f64d6f </ci>

<ci> mw48abd3c1_84c1_4bfe_9c74_0720c0b36bc2 </ci>

</apply>

</apply>

</apply>

</apply>

</apply>

</apply>

</math>

</kineticLaw>

</reaction>

<reaction id="mw09ad12f4_1084_4eb7_8920_88425d9850a9" name="PP_5 (serine hydroxymethyltransferase)" reversible="false" fast="false">

<annotation>

<COPASI xmlns="http://www.copasi.org/static/sbml">

<rdf:RDF xmlns:dcterms="http://purl.org/dc/terms/" xmlns:rdf="http://www.w3.org/1999/02/22-rdf-syntax-ns#">

<rdf:Description rdf:about="#COPASI73">

<dcterms:created>

<rdf:Description>

<dcterms:W3CDTF>2011-05-10T00:22:17Z</dcterms:W3CDTF>

</rdf:Description>

</dcterms:created>

</rdf:Description>

</rdf:RDF>

</COPASI>

</annotation>

<listOfReactants>

```xml
      <speciesReference species="mw3d986236_5c95_4128_9ee4_95d616f64d6f" stoichiometry="2"/>
    </listOfReactants>
    <listOfProducts>
      <speciesReference species="mw7bb27e61_6440_4c3b_af0a_6c1ecd36ff29"/>
    </listOfProducts>
    <kineticLaw>
      <math xmlns="http://www.w3.org/1998/Math/MathML">
        <apply>
          <divide/>
          <apply>
            <times/>
            <ci> mw2d5b6f12_0344_4f87_8e82_bc9605427f64 </ci>
            <ci> mw3d986236_5c95_4128_9ee4_95d616f64d6f </ci>
          </apply>
          <apply>
            <plus/>
            <ci> mw8b5162e2_5e83_4aa3_8271_8e2d38ca0494 </ci>
            <ci> mw3d986236_5c95_4128_9ee4_95d616f64d6f </ci>
          </apply>
        </apply>
      </math>
      <listOfParameters>
        <parameter id="mw8b5162e2_5e83_4aa3_8271_8e2d38ca0494" name="K1" value="6"/>
      </listOfParameters>
    </kineticLaw>
  </reaction>
  <reaction id="mwea5d8551_058c_4d6c_9dd9_2b43c029148e" name="PP_6 (hydroxypyruvate reductase)" reversible="false" fast="false">
    <annotation>
      <COPASI xmlns="http://www.copasi.org/static/sbml">
```

```
            <rdf:RDF xmlns:dcterms="http://purl.org/dc/terms/"
xmlns:rdf="http://www.w3.org/1999/02/22-rdf-syntax-ns#">
              <rdf:Description rdf:about="#COPASI74">
                <dcterms:created>
                  <rdf:Description>
                    <dcterms:W3CDTF>2011-05-10T01:04:31Z</dcterms:W3CDTF>
                  </rdf:Description>
                </dcterms:created>
              </rdf:Description>
            </rdf:RDF>
          </COPASI>
        </annotation>
        <listOfReactants>
          <speciesReference species="mw21f7bed2_a8e0_4d16_a4a4_8718de3f1c6a"/>
        </listOfReactants>
        <listOfProducts>
          <speciesReference species="mw3a8a1813_5005_474a_8fd1_d573b1ec121f"/>
        </listOfProducts>
        <kineticLaw>
          <math xmlns="http://www.w3.org/1998/Math/MathML">
            <apply>
              <divide/>
              <apply>
                <times/>
                <ci> mwf9b6aec2_50da_4731_963c_67dca8babe88 </ci>
                <apply>
                  <minus/>
                  <ci> mw21f7bed2_a8e0_4d16_a4a4_8718de3f1c6a </ci>
                  <apply>
                    <divide/>
                    <ci> mw3a8a1813_5005_474a_8fd1_d573b1ec121f </ci>
```

<ci> mw05e98de4_3951_43e5_ac00_ca56fb3f40d3 </ci>
</apply>
</apply>
</apply>
<apply>
<plus/>
<ci> mw21f7bed2_a8e0_4d16_a4a4_8718de3f1c6a </ci>
<apply>
<times/>
<ci> mw216d7d9c_5cbe_472f_a388_64d7d3889347 </ci>
<apply>
<plus/>
<cn type="integer"> 1 </cn>
<apply>
<divide/>
<ci> mw21f7bed2_a8e0_4d16_a4a4_8718de3f1c6a </ci>
<ci> mw55f32731_601d_4813_a73f_80bd3d7c7fa6 </ci>
</apply>
</apply>
</apply>
</apply>
</apply>
</math>
</kineticLaw>
</reaction>
<reaction id="mw58c999d8_b2e4_4aa3_b854_a7c56fd909a8" name="PP_7 (glycerate kinase)" reversible="false" fast="false">
<annotation>
<COPASI xmlns="http://www.copasi.org/static/sbml">
<rdf:RDF xmlns:dcterms="http://purl.org/dc/terms/" xmlns:rdf="http://www.w3.org/1999/02/22-rdf-syntax-ns#">
<rdf:Description rdf:about="#COPASI75">

```
        <dcterms:created>
          <rdf:Description>
            <dcterms:W3CDTF>2011-05-10T01:09:19Z</dcterms:W3CDTF>
          </rdf:Description>
        </dcterms:created>
      </rdf:Description>
    </rdf:RDF>
  </COPASI>
</annotation>
<listOfReactants>
  <speciesReference species="mw3a8a1813_5005_474a_8fd1_d573b1ec121f"/>
  <speciesReference species="mw8e2cc29c_c73e_4258_b3ba_2356620bb64e"/>
</listOfReactants>
<listOfProducts>
  <speciesReference species="mw372b8247_b11e_4d9b_be9b_e742fd2d3853"/>
  <speciesReference species="mwe8d1ae1f_35f9_45fd_bedf_7dbeef3972e2"/>
</listOfProducts>
<kineticLaw>
  <math xmlns="http://www.w3.org/1998/Math/MathML">
    <apply>
      <divide/>
      <apply>
        <times/>
        <ci> mwf1be6830_c2ee_4d96_a417_fb1e9115d10b </ci>
        <apply>
          <minus/>
          <apply>
            <times/>
            <ci> mw8e2cc29c_c73e_4258_b3ba_2356620bb64e </ci>
            <ci> mw3a8a1813_5005_474a_8fd1_d573b1ec121f </ci>
          </apply>
```

```
      <apply>
        <divide/>
        <apply>
          <times/>
          <ci> mwe8d1ae1f_35f9_45fd_bedf_7dbeef3972e2 </ci>
          <ci> mw372b8247_b11e_4d9b_be9b_e742fd2d3853 </ci>
        </apply>
        <ci> mwee6b22b8_8ca2_4b43_8638_750582325854 </ci>
      </apply>
    </apply>
  </apply>
  <apply>
    <times/>
    <apply>
      <plus/>
      <ci> mw8e2cc29c_c73e_4258_b3ba_2356620bb64e </ci>
      <apply>
        <times/>
        <ci> mwb4c4eaed_f8bb_4d91_bf8e_f0943ebabb45 </ci>
        <apply>
          <plus/>
          <cn type="integer"> 1 </cn>
          <apply>
            <divide/>
            <ci> mw372b8247_b11e_4d9b_be9b_e742fd2d3853 </ci>
            <ci> mwe32e388e_050d_4321_991e_7156fdc6d321 </ci>
          </apply>
        </apply>
      </apply>
    </apply>
    <apply>
```

```
            <plus/>
            <ci> mw3a8a1813_5005_474a_8fd1_d573b1ec121f </ci>
            <ci> mwbf3b18c6_626f_464e_8498_a175942a2b66 </ci>
          </apply>
        </apply>
      </apply>
    </math>
  </kineticLaw>
</reaction>
<reaction id="mwe4848368_1945_44c8_9b31_4e319b304d82" name="GC_1 (tartronate semialdehyde synthase)" reversible="false" fast="false">
  <annotation>
    <COPASI xmlns="http://www.copasi.org/static/sbml">
      <rdf:RDF xmlns:dcterms="http://purl.org/dc/terms/" xmlns:rdf="http://www.w3.org/1999/02/22-rdf-syntax-ns#">
        <rdf:Description rdf:about="#COPASI82">
          <dcterms:created>
            <rdf:Description>
              <dcterms:W3CDTF>2011-06-15T16:39:15Z</dcterms:W3CDTF>
            </rdf:Description>
          </dcterms:created>
        </rdf:Description>
      </rdf:RDF>
    </COPASI>
  </annotation>
  <listOfReactants>
    <speciesReference species="mwb970288f_4109_4c97_90b7_cc60512dc613" stoichiometry="2"/>
  </listOfReactants>
  <listOfProducts>
    <speciesReference species="mw7b17f46e_6246_48f0_9aeb_fb89cc666077"/>
  </listOfProducts>
```

<kineticLaw>
<math xmlns="http://www.w3.org/1998/Math/MathML">
<apply>
<divide/>
<apply>
<times/>
<ci> mw2ec57d34_aee7_4157_a680_a2a0ed50f517 </ci>
<ci> mwb970288f_4109_4c97_90b7_cc60512dc613 </ci>
</apply>
<apply>
<plus/>
<ci> mw17a7c44a_3eb3_4030_b0d2_f885efc0f98b </ci>
<ci> mwb970288f_4109_4c97_90b7_cc60512dc613 </ci>
</apply>
</apply>
</math>
</kineticLaw>
</reaction>
<reaction id="mwf0267a24_982c_4a2a_ba0e_eef348138918" name="GC_2 (tartronate semialdehyde reductase)" reversible="false" fast="false">
<annotation>
<COPASI xmlns="http://www.copasi.org/static/sbml">
<rdf:RDF xmlns:dcterms="http://purl.org/dc/terms/" xmlns:rdf="http://www.w3.org/1999/02/22-rdf-syntax-ns#">
<rdf:Description rdf:about="#COPASI84">
<dcterms:created>
<rdf:Description>
<dcterms:W3CDTF>2011-06-15T16:40:54Z</dcterms:W3CDTF>
</rdf:Description>
</dcterms:created>
</rdf:Description>
</rdf:RDF>

```
      </COPASI>
    </annotation>
    <listOfReactants>
      <speciesReference species="mw7b17f46e_6246_48f0_9aeb_fb89cc666077"/>
    </listOfReactants>
    <listOfProducts>
      <speciesReference species="mw3a8a1813_5005_474a_8fd1_d573b1ec121f"/>
    </listOfProducts>
    <kineticLaw>
      <math xmlns="http://www.w3.org/1998/Math/MathML">
        <apply>
          <divide/>
          <apply>
            <times/>
            <ci> mwaa87b632_3faa_428a_b731_6d7b21f7b26f </ci>
            <ci> mw7b17f46e_6246_48f0_9aeb_fb89cc666077 </ci>
          </apply>
          <apply>
            <plus/>
            <ci> mw92d1a502_da48_4a89_9ea0_8831de942be8 </ci>
            <ci> mw7b17f46e_6246_48f0_9aeb_fb89cc666077 </ci>
          </apply>
        </apply>
      </math>
    </kineticLaw>
  </reaction>
  <reaction id="mw4808f9af_cff4_44dc_9e63_3022ecc09276" name="OX_1 (glyoxylate oxidase)"
reversible="false" fast="false">
    <annotation>
      <COPASI xmlns="http://www.copasi.org/static/sbml">
        <rdf:RDF xmlns:dcterms="http://purl.org/dc/terms/"
xmlns:rdf="http://www.w3.org/1999/02/22-rdf-syntax-ns#">
```

```xml
      <rdf:Description rdf:about="#COPASI85">
        <dcterms:created>
          <rdf:Description>
            <dcterms:W3CDTF>2011-06-28T01:12:01Z</dcterms:W3CDTF>
          </rdf:Description>
        </dcterms:created>
      </rdf:Description>
    </rdf:RDF>
  </COPASI>
</annotation>
<listOfReactants>
  <speciesReference species="mwb970288f_4109_4c97_90b7_cc60512dc613"/>
</listOfReactants>
<listOfProducts>
  <speciesReference species="mw3885b0e0_19bc_4407_a41c_94c1f48987d7"/>
</listOfProducts>
<kineticLaw>
  <math xmlns="http://www.w3.org/1998/Math/MathML">
    <apply>
      <divide/>
      <apply>
        <times/>
        <ci> mw7bde3bd4_7b60_4626_a4cd_83436934369a </ci>
        <ci> mwb970288f_4109_4c97_90b7_cc60512dc613 </ci>
      </apply>
      <apply>
        <plus/>
        <ci> mwa61fcd80_7091_43db_bd8f_5686bc24a7ee </ci>
        <ci> mwb970288f_4109_4c97_90b7_cc60512dc613 </ci>
      </apply>
    </apply>
```

</math>
</kineticLaw>
</reaction>
<reaction id="mwd16b4bde_0943_4ea8_82b6_0fc305e8e73f" name="GLY_1 alpha (phosphofructokinase)" reversible="false" fast="false">
<listOfReactants>
<speciesReference species="mwfcedd013_9e1c_4e21_9a5a_93ddde2ffc11"/>
<speciesReference species="mw8e2cc29c_c73e_4258_b3ba_2356620bb64e"/>
</listOfReactants>
<listOfProducts>
<speciesReference species="mw2a09f89f_5936_4376_a2ab_4ff1bbbd4153"/>
<speciesReference species="mwe8d1ae1f_35f9_45fd_bedf_7dbeef3972e2"/>
</listOfProducts>
<kineticLaw>
<math xmlns="http://www.w3.org/1998/Math/MathML">
<apply>
<divide/>
<apply>
<divide/>
<apply>
<times/>
<ci> mw47ef96f3_f55b_4c2f_aeb6_7b00be2f7c33 </ci>
<ci> mwfcedd013_9e1c_4e21_9a5a_93ddde2ffc11 </ci>
<ci> mw8e2cc29c_c73e_4258_b3ba_2356620bb64e </ci>
</apply>
<apply>
<times/>
<ci> mwfab2bb7b_fa67_47d4_b71e_f36e49d4c2f7 </ci>
<ci> mwc92f9b3a_36c7_4c3f_a179_2eb02421e208 </ci>
</apply>
</apply>

```
            <apply>
              <plus/>
              <cn type="integer"> 1 </cn>
              <apply>
                <times/>
                <apply>
                  <divide/>
                  <ci> mw8e2cc29c_c73e_4258_b3ba_2356620bb64e </ci>
                  <ci> mwc92f9b3a_36c7_4c3f_a179_2eb02421e208 </ci>
                </apply>
                <apply>
                  <divide/>
                  <ci> mwfcedd013_9e1c_4e21_9a5a_93ddde2ffc11 </ci>
                  <ci> mwfab2bb7b_fa67_47d4_b71e_f36e49d4c2f7 </ci>
                </apply>
              </apply>
            </apply>
          </apply>
        </math>
      </kineticLaw>
    </reaction>
    <reaction id="mw113707e0_f8a9_438c_81c7_79135f7dab86" name="GLY_1 beta
(phosphofructokinase)" reversible="false" fast="false">
      <listOfReactants>
        <speciesReference species="mwfcedd013_9e1c_4e21_9a5a_93ddde2ffc11"/>
        <speciesReference species="mw8e2cc29c_c73e_4258_b3ba_2356620bb64e"/>
      </listOfReactants>
      <listOfProducts>
        <speciesReference species="mw2a09f89f_5936_4376_a2ab_4ff1bbbd4153"/>
        <speciesReference species="mwe8d1ae1f_35f9_45fd_bedf_7dbeef3972e2"/>
      </listOfProducts>
```

```
<kineticLaw>
  <math xmlns="http://www.w3.org/1998/Math/MathML">
    <apply>
      <divide/>
      <apply>
        <divide/>
        <apply>
          <times/>
          <ci> mwe08f3352_d986_4a9a_acea_33701d19a3b3 </ci>
          <ci> mwfcedd013_9e1c_4e21_9a5a_93ddde2ffc11 </ci>
          <ci> mw8e2cc29c_c73e_4258_b3ba_2356620bb64e </ci>
        </apply>
        <apply>
          <times/>
          <ci> mwd2e258fa_4740_4b3b_816e_0b0e55bdb005 </ci>
          <ci> mw9bc87645_2d3f_4a39_8dff_d0074e096984 </ci>
        </apply>
      </apply>
      <apply>
        <plus/>
        <cn type="integer"> 1 </cn>
        <apply>
          <times/>
          <apply>
            <divide/>
            <ci> mw8e2cc29c_c73e_4258_b3ba_2356620bb64e </ci>
            <ci> mw9bc87645_2d3f_4a39_8dff_d0074e096984 </ci>
          </apply>
          <apply>
            <divide/>
            <ci> mwfcedd013_9e1c_4e21_9a5a_93ddde2ffc11 </ci>
```

<ci> mwd2e258fa_4740_4b3b_816e_0b0e55bdb005 </ci>

</apply>

</apply>

</apply>

</apply>

</math>

</kineticLaw>

</reaction>

<reaction id="mw3df19da1_ab8d_4f5e_8d0c_c5165ecfd6db" name="GLY_2 (glyceraldehyde 3-phosphate dehydrogenase)" reversible="false" fast="false">

<listOfReactants>

<speciesReference species="mw5b528d37_b514_46e5_a018_0173afdae714"/>

<speciesReference species="mw7c834bb1_1865_4950_aa20_f3eceb92b21d"/>

</listOfReactants>

<listOfProducts>

<speciesReference species="mwc8b5cb3b_9f99_46df_a6e4_0126749144fc"/>

<speciesReference species="mwd2e6e004_23f5_4cd8_81e0_8385021ec904"/>

</listOfProducts>

<kineticLaw>

<math xmlns="http://www.w3.org/1998/Math/MathML">

<apply>

<divide/>

<apply>

<times/>

<ci> mw38ba402b_328c_454f_807b_2a5d33b8d48f </ci>

<ci> mw5b528d37_b514_46e5_a018_0173afdae714 </ci>

<ci> mw7c834bb1_1865_4950_aa20_f3eceb92b21d </ci>

</apply>

<apply>

<times/>

<apply>

<plus/>
<cn type="integer"> 1 </cn>
<apply>
<divide/>
<ci> mw5b528d37_b514_46e5_a018_0173afdae714 </ci>
<ci> mw4da83b47_6347_43ed_b1b6_c95ab9075f9d </ci>
</apply>
<apply>
<divide/>
<ci> mw372b8247_b11e_4d9b_be9b_e742fd2d3853 </ci>
<ci> mw02a594bf_b380_46b9_acac_e7344c88da82 </ci>
</apply>
</apply>
<apply>
<plus/>
<cn type="integer"> 1 </cn>
<apply>
<divide/>
<ci> mw7c834bb1_1865_4950_aa20_f3eceb92b21d </ci>
<ci> mw640c5b04_67dd_43d7_8b11_069cf242288b </ci>
</apply>
<apply>
<divide/>
<ci> mwd2e6e004_23f5_4cd8_81e0_8385021ec904 </ci>
<ci> mwebd2cdb3_d654_4e7e_a036_7180e1269833 </ci>
</apply>
</apply>
</apply>
</apply>
</math>
</kineticLaw>

```
</reaction>
<reaction id="mw5a71ef48_b0a8_4172_b0bc_922d96c83458" name="GLY_3a (phosphoglycerate mutase)" fast="false">
 <listOfReactants>
  <speciesReference species="mw372b8247_b11e_4d9b_be9b_e742fd2d3853"/>
 </listOfReactants>
 <listOfProducts>
  <speciesReference species="mwc25bd841_1335_4e94_9ffa_621b205bc0da"/>
 </listOfProducts>
 <kineticLaw>
  <math xmlns="http://www.w3.org/1998/Math/MathML">
   <apply>
    <divide/>
    <apply>
     <times/>
     <ci> mwa577376f_a6e1_47eb_bf4d_2b6dc53f1249 </ci>
     <apply>
      <minus/>
      <ci> mw372b8247_b11e_4d9b_be9b_e742fd2d3853 </ci>
      <apply>
       <divide/>
       <ci> mwc25bd841_1335_4e94_9ffa_621b205bc0da </ci>
       <ci> mw3f9f4337_1baa_4946_8e90_d13ca5c3adbf </ci>
      </apply>
     </apply>
    </apply>
    <apply>
     <times/>
     <ci> mw7d7f5782_23e4_4797_8dc7_355ec98f4d6a </ci>
     <apply>
      <plus/>
```

```
            <cn type="integer"> 1 </cn>
            <apply>
              <divide/>
              <ci> mw372b8247_b11e_4d9b_be9b_e742fd2d3853 </ci>
              <ci> mw7d7f5782_23e4_4797_8dc7_355ec98f4d6a </ci>
            </apply>
            <apply>
              <divide/>
              <ci> mwc25bd841_1335_4e94_9ffa_621b205bc0da </ci>
              <ci> mw28d0b788_21a0_4f17_ac4c_9b32d44ae058 </ci>
            </apply>
          </apply>
        </apply>
      </apply>
    </math>
  </kineticLaw>
</reaction>
<reaction id="mwde55d662_d291_4e0b_b8e1_c7b7b523d24f" name="GLY_3b
(phosphoglycerate mutase)" fast="false">
  <listOfReactants>
    <speciesReference species="mw372b8247_b11e_4d9b_be9b_e742fd2d3853"/>
  </listOfReactants>
  <listOfProducts>
    <speciesReference species="mwc25bd841_1335_4e94_9ffa_621b205bc0da"/>
  </listOfProducts>
  <kineticLaw>
    <math xmlns="http://www.w3.org/1998/Math/MathML">
      <apply>
        <divide/>
        <apply>
          <times/>
```

```
      <ci> mw4674ad99_0bbe_4aef_99fa_49937a6f49d7 </ci>
      <apply>
        <minus/>
        <ci> mw372b8247_b11e_4d9b_be9b_e742fd2d3853 </ci>
        <apply>
          <divide/>
          <ci> mwc25bd841_1335_4e94_9ffa_621b205bc0da </ci>
          <ci> mw3f9f4337_1baa_4946_8e90_d13ca5c3adbf </ci>
        </apply>
      </apply>
    </apply>
    <apply>
      <times/>
      <ci> mwc0011b36_3da6_4d34_9157_28ac5f77a5df </ci>
      <apply>
        <plus/>
        <cn type="integer"> 1 </cn>
        <apply>
          <divide/>
          <ci> mw372b8247_b11e_4d9b_be9b_e742fd2d3853 </ci>
          <ci> mwc0011b36_3da6_4d34_9157_28ac5f77a5df </ci>
        </apply>
        <apply>
          <divide/>
          <ci> mwc25bd841_1335_4e94_9ffa_621b205bc0da </ci>
          <ci> mw8a2c7a1e_c472_4f06_a4c7_38688feecabc </ci>
        </apply>
      </apply>
    </apply>
  </apply>
</math>
```

```
    </kineticLaw>
   </reaction>
   <reaction id="mw60673d20_0456_47ee_a3bd_9f9a3c999127" name="GLY_3c (phosphoglycerate mutase)" fast="false">
    <listOfReactants>
     <speciesReference species="mw372b8247_b11e_4d9b_be9b_e742fd2d3853"/>
    </listOfReactants>
    <listOfProducts>
     <speciesReference species="mwc25bd841_1335_4e94_9ffa_621b205bc0da"/>
    </listOfProducts>
    <kineticLaw>
     <math xmlns="http://www.w3.org/1998/Math/MathML">
      <apply>
       <divide/>
       <apply>
        <times/>
        <ci> mw11591b16_f15e_41a6_890c_7da51ea27d51 </ci>
        <apply>
         <minus/>
         <ci> mw372b8247_b11e_4d9b_be9b_e742fd2d3853 </ci>
         <apply>
          <divide/>
          <ci> mwc25bd841_1335_4e94_9ffa_621b205bc0da </ci>
          <ci> mw3f9f4337_1baa_4946_8e90_d13ca5c3adbf </ci>
         </apply>
        </apply>
       </apply>
       <apply>
        <times/>
        <ci> mw56023d39_08c8_49e5_a347_a28c871b8290 </ci>
        <apply>
```

```
              <plus/>
              <cn type="integer"> 1 </cn>
              <apply>
                <divide/>
                <ci> mw372b8247_b11e_4d9b_be9b_e742fd2d3853 </ci>
                <ci> mw56023d39_08c8_49e5_a347_a28c871b8290 </ci>
              </apply>
              <apply>
                <divide/>
                <ci> mwc25bd841_1335_4e94_9ffa_621b205bc0da </ci>
                <ci> mwc5650e27_4e7c_4064_8600_9ff3fd5f22ca </ci>
              </apply>
            </apply>
          </apply>
        </apply>
      </math>
    </kineticLaw>
  </reaction>
  <reaction id="mw00fd453f_e19f_408e_89bf_fc0e9fd43711" name="GLY_4 (enolase)"
fast="false">
    <annotation>
      <COPASI xmlns="http://www.copasi.org/static/sbml">
        <rdf:RDF xmlns:dcterms="http://purl.org/dc/terms/"
xmlns:rdf="http://www.w3.org/1999/02/22-rdf-syntax-ns#">
          <rdf:Description rdf:about="#COPASI78">
            <dcterms:created>
              <rdf:Description>
                <dcterms:W3CDTF>2011-05-10T15:12:27Z</dcterms:W3CDTF>
              </rdf:Description>
            </dcterms:created>
          </rdf:Description>
        </rdf:RDF>
```

</COPASI>
</annotation>
<listOfReactants>
<speciesReference species="mwc25bd841_1335_4e94_9ffa_621b205bc0da"/>
</listOfReactants>
<listOfProducts>
<speciesReference species="mw6951dd88_19cf_4045_95c4_0e8e2bf976c1"/>
</listOfProducts>
<kineticLaw>
<math xmlns="http://www.w3.org/1998/Math/MathML">
<apply>
<divide/>
<apply>
<times/>
<ci> mw20c2fea9_d469_4e35_bdf8_38cbe9f5bd92 </ci>
<apply>
<minus/>
<ci> mwc25bd841_1335_4e94_9ffa_621b205bc0da </ci>
<apply>
<divide/>
<ci> mw6951dd88_19cf_4045_95c4_0e8e2bf976c1 </ci>
<ci> mw963788fc_9773_4082_8fe4_0f8ea1e81867 </ci>
</apply>
</apply>
</apply>
<apply>
<times/>
<ci> mw8e5540c2_ecd1_4e3e_bb5a_651353fa0ec2 </ci>
<apply>
<plus/>
<cn type="integer"> 1 </cn>

```
            <apply>
              <divide/>
              <ci> mwc25bd841_1335_4e94_9ffa_621b205bc0da </ci>
              <ci> mw8e5540c2_ecd1_4e3e_bb5a_651353fa0ec2 </ci>
            </apply>
            <apply>
              <divide/>
              <ci> mw6951dd88_19cf_4045_95c4_0e8e2bf976c1 </ci>
              <ci> mwb8a92b6d_ce94_48d0_8b18_58660e599368 </ci>
            </apply>
          </apply>
        </apply>
      </apply>
    </math>
  </kineticLaw>
</reaction>
<reaction id="mw35c7c9f7_cdbb_4e03_b1c9_6e4e6f165be2" name="PKET1a (phosphoketolase)" fast="false">
  <listOfReactants>
    <speciesReference species="mwfcedd013_9e1c_4e21_9a5a_93ddde2ffc11"/>
    <speciesReference species="mw5b528d37_b514_46e5_a018_0173afdae714"/>
    <speciesReference species="mw7c70a014_6b69_42b3_9fbd_1d63cb722ae5"/>
  </listOfReactants>
  <listOfProducts>
    <speciesReference species="mw543ef166_8322_44f0_9435_270a9f9672da"/>
    <speciesReference species="mwaa8b93c8_308a_4d19_b732_22cb31f77620"/>
    <speciesReference species="mw5b528d37_b514_46e5_a018_0173afdae714"/>
    <speciesReference species="mw7c70a014_6b69_42b3_9fbd_1d63cb722ae5"/>
  </listOfProducts>
  <kineticLaw>
    <math xmlns="http://www.w3.org/1998/Math/MathML">
```

<apply>
<divide/>
<apply>
<divide/>
<apply>
<times/>
<ci> mw6e7fabe6_de80_41fa_9146_5e9b7ec4bc63 </ci>
<apply>
<minus/>
<ci> mwfcedd013_9e1c_4e21_9a5a_93ddde2ffc11 </ci>
<apply>
<divide/>
<apply>
<times/>
<ci> mw543ef166_8322_44f0_9435_270a9f9672da </ci>
<ci> mwaa8b93c8_308a_4d19_b732_22cb31f77620 </ci>
</apply>
<ci> mw066182b3_2182_44b1_b745_125e1968e0bc </ci>
</apply>
</apply>
</apply>
<ci> mw8e6b4f11_baec_4a0f_983d_3d1d46cb91cb </ci>
</apply>
<apply>
<times/>
<apply>
<plus/>
<cn type="integer"> 1 </cn>
<apply>
<divide/>
<ci> mwfcedd013_9e1c_4e21_9a5a_93ddde2ffc11 </ci>

<ci> mw8e6b4f11_baec_4a0f_983d_3d1d46cb91cb </ci>
</apply>
<apply>
<divide/>
<ci> mwaa8b93c8_308a_4d19_b732_22cb31f77620 </ci>
<ci> mw8b16f07a_00d8_489e_ae07_2dc92af4dfb9 </ci>
</apply>
</apply>
<apply>
<plus/>
<cn type="integer"> 1 </cn>
<apply>
<divide/>
<ci> mw543ef166_8322_44f0_9435_270a9f9672da </ci>
<ci> mw514d5907_3da1_4e17_8024_f637af4b38fa </ci>
</apply>
</apply>
<apply>
<plus/>
<cn type="integer"> 1 </cn>
<apply>
<divide/>
<ci> mw7c70a014_6b69_42b3_9fbd_1d63cb722ae5 </ci>
<ci> mw562c606a_6226_47bc_8fe9_db84aa73a146 </ci>
</apply>
<apply>
<divide/>
<ci> mw5b528d37_b514_46e5_a018_0173afdae714 </ci>
<ci> mw9d34e88a_6503_48b9_a787_58c326a932a5 </ci>
</apply>
</apply>

```
      </apply>
     </apply>
    </math>
   </kineticLaw>
  </reaction>
  <reaction id="mw3c50934d_90c0_4d15_aada_7268ed6ec29a" name="PKET1b" fast="false">
   <listOfReactants>
    <speciesReference species="mw7c70a014_6b69_42b3_9fbd_1d63cb722ae5"/>
    <speciesReference species="mwfcedd013_9e1c_4e21_9a5a_93ddde2ffc11"/>
    <speciesReference species="mwaa8b93c8_308a_4d19_b732_22cb31f77620"/>
   </listOfReactants>
   <listOfProducts>
    <speciesReference species="mw5b528d37_b514_46e5_a018_0173afdae714"/>
    <speciesReference species="mw543ef166_8322_44f0_9435_270a9f9672da"/>
    <speciesReference species="mwaa8b93c8_308a_4d19_b732_22cb31f77620"/>
    <speciesReference species="mwfcedd013_9e1c_4e21_9a5a_93ddde2ffc11"/>
   </listOfProducts>
   <kineticLaw>
    <math xmlns="http://www.w3.org/1998/Math/MathML">
     <apply>
      <divide/>
      <apply>
       <divide/>
       <apply>
        <times/>
        <ci> mw0ab3c1a9_2d85_4242_b2c8_8668592bc4b4 </ci>
        <apply>
         <minus/>
         <ci> mw7c70a014_6b69_42b3_9fbd_1d63cb722ae5 </ci>
         <apply>
          <divide/>
```

<apply>
<times/>
<ci> mw543ef166_8322_44f0_9435_270a9f9672da </ci>
<ci> mw5b528d37_b514_46e5_a018_0173afdae714 </ci>
</apply>
<ci> mw44c8ec20_4a1e_454f_a1a0_42d862b117ae </ci>
</apply>
</apply>
</apply>
<ci> mw057e7327_64bf_4b85_bb90_78da6ba5980f </ci>
</apply>
<apply>
<times/>
<apply>
<plus/>
<cn type="integer"> 1 </cn>
<apply>
<divide/>
<ci> mwfcedd013_9e1c_4e21_9a5a_93ddde2ffc11 </ci>
<ci> mw77007da8_e432_48eb_9c52_ed3c2c1b5f8c </ci>
</apply>
<apply>
<divide/>
<ci> mwaa8b93c8_308a_4d19_b732_22cb31f77620 </ci>
<ci> mw9580aa5c_a5df_472e_aad4_d7d8dfaf50c5 </ci>
</apply>
</apply>
<apply>
<plus/>
<cn type="integer"> 1 </cn>
<apply>

<divide/>
<ci> mw543ef166_8322_44f0_9435_270a9f9672da </ci>
<ci> mw6dff67cf_5c46_4621_9902_c290a3f63d5d </ci>
</apply>
</apply>
<apply>
<plus/>
<cn type="integer"> 1 </cn>
<apply>
<divide/>
<ci> mw7c70a014_6b69_42b3_9fbd_1d63cb722ae5 </ci>
<ci> mw057e7327_64bf_4b85_bb90_78da6ba5980f </ci>
</apply>
<apply>
<divide/>
<ci> mw5b528d37_b514_46e5_a018_0173afdae714 </ci>
<ci> mwa0585ea2_d52f_4310_adf2_e8de58065ae9 </ci>
</apply>
</apply>
</apply>
</apply>
</math>
</kineticLaw>
</reaction>
<reaction id="mw6674ad2e_e621_4437_bbf3_1e6eaa0ed85d" name="PKET2a (phosphoketolase)" fast="false">
<listOfReactants>
<speciesReference species="mwfcedd013_9e1c_4e21_9a5a_93ddde2ffc11"/>
<speciesReference species="mw5b528d37_b514_46e5_a018_0173afdae714"/>
<speciesReference species="mw7c70a014_6b69_42b3_9fbd_1d63cb722ae5"/>
</listOfReactants>

```
<listOfProducts>
  <speciesReference species="mw543ef166_8322_44f0_9435_270a9f9672da"/>
  <speciesReference species="mwaa8b93c8_308a_4d19_b732_22cb31f77620"/>
  <speciesReference species="mw5b528d37_b514_46e5_a018_0173afdae714"/>
  <speciesReference species="mw7c70a014_6b69_42b3_9fbd_1d63cb722ae5"/>
</listOfProducts>
<kineticLaw>
  <math xmlns="http://www.w3.org/1998/Math/MathML">
    <apply>
      <divide/>
      <apply>
        <divide/>
        <apply>
          <times/>
          <ci> mw9e5312fb_9ca1_4ae7_8fef_e40c70633af3 </ci>
          <apply>
            <minus/>
            <ci> mwfcedd013_9e1c_4e21_9a5a_93ddde2ffc11 </ci>
            <apply>
              <divide/>
              <apply>
                <times/>
                <ci> mw543ef166_8322_44f0_9435_270a9f9672da </ci>
                <ci> mwaa8b93c8_308a_4d19_b732_22cb31f77620 </ci>
              </apply>
              <ci> mw066182b3_2182_44b1_b745_125e1968e0bc </ci>
            </apply>
          </apply>
        </apply>
        <ci> mw7c84a538_b915_4a91_8cc2_04c534c01ab8 </ci>
      </apply>
```

<apply>
<times/>
<apply>
<plus/>
<cn type="integer"> 1 </cn>
<apply>
<divide/>
<ci> mwfcedd013_9e1c_4e21_9a5a_93ddde2ffc11 </ci>
<ci> mw7c84a538_b915_4a91_8cc2_04c534c01ab8 </ci>
</apply>
<apply>
<divide/>
<ci> mwaa8b93c8_308a_4d19_b732_22cb31f77620 </ci>
<ci> mw4d62dae0_b6d6_45ba_8fb6_caf10d3295dc </ci>
</apply>
</apply>
<apply>
<plus/>
<cn type="integer"> 1 </cn>
<apply>
<divide/>
<ci> mw543ef166_8322_44f0_9435_270a9f9672da </ci>
<ci> mw9f4be612_37b6_425f_863f_c3234aaafd9c </ci>
</apply>
</apply>
<apply>
<plus/>
<cn type="integer"> 1 </cn>
<apply>
<divide/>
<ci> mw7c70a014_6b69_42b3_9fbd_1d63cb722ae5 </ci>

```
          <ci> mw457a9b1f_ac25_45b4_adb7_c6c807be3474 </ci>
         </apply>
         <apply>
          <divide/>
          <ci> mw5b528d37_b514_46e5_a018_0173afdae714 </ci>
          <ci> mwa723f027_cc0e_426d_b99e_dd1f00ba7a3e </ci>
         </apply>
        </apply>
       </apply>
      </apply>
     </math>
    </kineticLaw>
   </reaction>
   <reaction id="mw840f3e7f_494a_48af_abeb_9edc6001b521" name="PKET2b" fast="false">
    <listOfReactants>
     <speciesReference species="mw7c70a014_6b69_42b3_9fbd_1d63cb722ae5"/>
     <speciesReference species="mwfcedd013_9e1c_4e21_9a5a_93ddde2ffc11"/>
     <speciesReference species="mwaa8b93c8_308a_4d19_b732_22cb31f77620"/>
    </listOfReactants>
    <listOfProducts>
     <speciesReference species="mw5b528d37_b514_46e5_a018_0173afdae714"/>
     <speciesReference species="mw543ef166_8322_44f0_9435_270a9f9672da"/>
     <speciesReference species="mwaa8b93c8_308a_4d19_b732_22cb31f77620"/>
     <speciesReference species="mwfcedd013_9e1c_4e21_9a5a_93ddde2ffc11"/>
    </listOfProducts>
    <kineticLaw>
     <math xmlns="http://www.w3.org/1998/Math/MathML">
      <apply>
       <divide/>
       <apply>
        <divide/>
```

<apply>
<times/>
<ci> mw5a9817b0_1b0d_4667_a467_59f6072696c8 </ci>
<apply>
<minus/>
<ci> mw7c70a014_6b69_42b3_9fbd_1d63cb722ae5 </ci>
<apply>
<divide/>
<apply>
<times/>
<ci> mw543ef166_8322_44f0_9435_270a9f9672da </ci>
<ci> mw5b528d37_b514_46e5_a018_0173afdae714 </ci>
</apply>
<ci> mw44c8ec20_4a1e_454f_a1a0_42d862b117ae </ci>
</apply>
</apply>
</apply>
<ci> mwff222c2b_bc40_4d59_bfcd_63aeb04c86bd </ci>
</apply>
<apply>
<times/>
<apply>
<plus/>
<cn type="integer"> 1 </cn>
<apply>
<divide/>
<ci> mwfcedd013_9e1c_4e21_9a5a_93ddde2ffc11 </ci>
<ci> mw08f2e5c2_6905_4c90_8f37_016935f5d8ec </ci>
</apply>
<apply>
<divide/>

```
          <ci> mwaa8b93c8_308a_4d19_b732_22cb31f77620 </ci>
          <ci> mw4c5adb36_23ae_4318_8b5f_6f80df2be00a </ci>
        </apply>
      </apply>
      <apply>
        <plus/>
        <cn type="integer"> 1 </cn>
        <apply>
          <divide/>
          <ci> mw543ef166_8322_44f0_9435_270a9f9672da </ci>
          <ci> mwc6a7cd20_1464_4521_88e6_145752ed585d </ci>
        </apply>
      </apply>
      <apply>
        <plus/>
        <cn type="integer"> 1 </cn>
        <apply>
          <divide/>
          <ci> mw7c70a014_6b69_42b3_9fbd_1d63cb722ae5 </ci>
          <ci> mwff222c2b_bc40_4d59_bfcd_63aeb04c86bd </ci>
        </apply>
        <apply>
          <divide/>
          <ci> mw5b528d37_b514_46e5_a018_0173afdae714 </ci>
          <ci> mw8d162e7c_404d_4423_8f50_9c55f37e0d2c </ci>
        </apply>
      </apply>
    </apply>
  </apply>
 </math>
</kineticLaw>
```

```
</reaction>
<reaction id="mw99147fb3_2e7b_4784_950b_aa774cf1edfa" name="Sink GAP"
reversible="false" fast="false">
<annotation>
<COPASI xmlns="http://www.copasi.org/static/sbml">
<rdf:RDF xmlns:dcterms="http://purl.org/dc/terms/"
xmlns:rdf="http://www.w3.org/1999/02/22-rdf-syntax-ns#">
<rdf:Description rdf:about="#COPASI67">
<dcterms:created>
<rdf:Description>
<dcterms:W3CDTF>2011-04-26T00:16:45Z</dcterms:W3CDTF>
</rdf:Description>
</dcterms:created>
</rdf:Description>
</rdf:RDF>
</COPASI>
</annotation>
<listOfReactants>
<speciesReference species="mw5b528d37_b514_46e5_a018_0173afdae714"/>
</listOfReactants>
<listOfProducts>
<speciesReference species="mwa5c286c8_0311_47bb_a72d_717e0cdfb845"
stoichiometry="3"/>
</listOfProducts>
<kineticLaw>
<math xmlns="http://www.w3.org/1998/Math/MathML">
<apply>
<divide/>
<apply>
<times/>
<ci> mwf7816b82_0693_49ca_9589_4c1ec5f35110 </ci>
<ci> mw5b528d37_b514_46e5_a018_0173afdae714 </ci>
```

</apply>
<apply>
<plus/>
<ci> mw9c17270d_1b98_4c92_8c4f_0bf91f94ac5c </ci>
<ci> mw5b528d37_b514_46e5_a018_0173afdae714 </ci>
</apply>
</apply>
</math>
</kineticLaw>
</reaction>
<reaction id="mwc952dd7d_b439_4379_adcc_1e4452ea436c" name="Sink E4P" reversible="false" fast="false">
<annotation>
<COPASI xmlns="http://www.copasi.org/static/sbml">
<rdf:RDF xmlns:dcterms="http://purl.org/dc/terms/" xmlns:rdf="http://www.w3.org/1999/02/22-rdf-syntax-ns#">
<rdf:Description rdf:about="#COPASI68">
<dcterms:created>
<rdf:Description>
<dcterms:W3CDTF>2011-04-26T00:17:24Z</dcterms:W3CDTF>
</rdf:Description>
</dcterms:created>
</rdf:Description>
</rdf:RDF>
</COPASI>
</annotation>
<listOfReactants>
<speciesReference species="mwaa8b93c8_308a_4d19_b732_22cb31f77620"/>
</listOfReactants>
<listOfProducts>
<speciesReference species="mwd6fab0c0_73f8_45ef_b32f_c7113be8258a" stoichiometry="4"/>

```
        </listOfProducts>
        <kineticLaw>
          <math xmlns="http://www.w3.org/1998/Math/MathML">
            <apply>
              <divide/>
              <apply>
                <times/>
                <ci> mwd116d1da_a4e2_47b1_b71c_643640c7faac </ci>
                <ci> mwaa8b93c8_308a_4d19_b732_22cb31f77620 </ci>
              </apply>
              <apply>
                <plus/>
                <ci> mw03716e78_6785_43c8_9249_0ab554cf9111 </ci>
                <ci> mwaa8b93c8_308a_4d19_b732_22cb31f77620 </ci>
              </apply>
            </apply>
          </math>
        </kineticLaw>
      </reaction>
      <reaction id="mw0d1877ca_271f_4893_9458_d6c989ad4a87" name="Sink Ri5P"
reversible="false" fast="false">
        <annotation>
          <COPASI xmlns="http://www.copasi.org/static/sbml">
            <rdf:RDF xmlns:dcterms="http://purl.org/dc/terms/"
xmlns:rdf="http://www.w3.org/1999/02/22-rdf-syntax-ns#">
              <rdf:Description rdf:about="#COPASI69">
                <dcterms:created>
                  <rdf:Description>
                    <dcterms:W3CDTF>2011-04-26T00:17:46Z</dcterms:W3CDTF>
                  </rdf:Description>
                </dcterms:created>
              </rdf:Description>
```

```
          </rdf:RDF>
        </COPASI>
      </annotation>
      <listOfReactants>
        <speciesReference species="mw26816e75_d092_459e_bb3d_70aafa096e4d"/>
      </listOfReactants>
      <listOfProducts>
        <speciesReference species="mw24e36765_3502_4855_9948_95a6bb63a7a1"
stoichiometry="5"/>
      </listOfProducts>
      <kineticLaw>
        <math xmlns="http://www.w3.org/1998/Math/MathML">
          <apply>
            <divide/>
            <apply>
              <times/>
              <ci> mw089625ae_78a0_4e60_953d_7bccf06a6d3f </ci>
              <ci> mw26816e75_d092_459e_bb3d_70aafa096e4d </ci>
            </apply>
            <apply>
              <plus/>
              <ci> mw4782b972_ca60_4b9e_96dd_9dba1be35a3e </ci>
              <ci> mw26816e75_d092_459e_bb3d_70aafa096e4d </ci>
            </apply>
          </apply>
        </math>
      </kineticLaw>
    </reaction>
    <reaction id="mw476b8efc_88f1_4d1d_9c20_c54b9cf9c438" name="Sink TSA" reversible="false"
fast="false">
      <annotation>
        <COPASI xmlns="http://www.copasi.org/static/sbml">
```

```
<rdf:RDF xmlns:dcterms="http://purl.org/dc/terms/"
xmlns:rdf="http://www.w3.org/1999/02/22-rdf-syntax-ns#">
  &lt;rdf:Description rdf:about="#COPASI83"&gt;
    &lt;dcterms:created&gt;
      &lt;rdf:Description&gt;
        &lt;dcterms:W3CDTF&gt;2011-06-15T16:40:30Z&lt;/dcterms:W3CDTF&gt;
      &lt;/rdf:Description&gt;
    &lt;/dcterms:created&gt;
  &lt;/rdf:Description&gt;
&lt;/rdf:RDF&gt;
&lt;/COPASI&gt;
&lt;/annotation&gt;
&lt;listOfReactants&gt;
  &lt;speciesReference species="mw7b17f46e_6246_48f0_9aeb_fb89cc666077"/&gt;
&lt;/listOfReactants&gt;
&lt;listOfProducts&gt;
  &lt;speciesReference species="mw5ef07c48_166e_4959_9987_d5fc6d58add4"/&gt;
&lt;/listOfProducts&gt;
&lt;kineticLaw&gt;
  &lt;math xmlns="http://www.w3.org/1998/Math/MathML"&gt;
    &lt;apply&gt;
      &lt;divide/&gt;
      &lt;apply&gt;
        &lt;times/&gt;
        &lt;ci&gt; mw4732b2f8_32c5_4514_b508_4dc1858f5f4c &lt;/ci&gt;
        &lt;ci&gt; mw7b17f46e_6246_48f0_9aeb_fb89cc666077 &lt;/ci&gt;
      &lt;/apply&gt;
      &lt;apply&gt;
        &lt;plus/&gt;
        &lt;ci&gt; mw5768ed07_e6fa_4e82_994f_f0f9a1a0a3a8 &lt;/ci&gt;
        &lt;ci&gt; mw7b17f46e_6246_48f0_9aeb_fb89cc666077 &lt;/ci&gt;
```

</apply>

</apply>

</math>

</kineticLaw>

</reaction>

<reaction id="mw2a701fba_46ed_4995_bea7_3a3434be45dc" name="Sink OXA" reversible="false" fast="false">

<annotation>

<COPASI xmlns="http://www.copasi.org/static/sbml">

<rdf:RDF xmlns:dcterms="http://purl.org/dc/terms/" xmlns:rdf="http://www.w3.org/1999/02/22-rdf-syntax-ns#">

<rdf:Description rdf:about="#COPASI86">

<dcterms:created>

<rdf:Description>

<dcterms:W3CDTF>2011-06-28T01:12:33Z</dcterms:W3CDTF>

</rdf:Description>

</dcterms:created>

</rdf:Description>

</rdf:RDF>

</COPASI>

</annotation>

<listOfReactants>

<speciesReference species="mw3885b0e0_19bc_4407_a41c_94c1f48987d7"/>

</listOfReactants>

<listOfProducts>

<speciesReference species="mw6b76fe71_4295_4311_999a_67179a3d5a74"/>

</listOfProducts>

<kineticLaw>

<math xmlns="http://www.w3.org/1998/Math/MathML">

<apply>

<divide/>

<apply>

<times/>

<ci> mwca5c5991_e869_4364_8240_76e056ea0d3a </ci>

<ci> mw3885b0e0_19bc_4407_a41c_94c1f48987d7 </ci>

</apply>

<apply>

<plus/>

<ci> mw1d4b50a5_6beb_46eb_8599_024c83c627b2 </ci>

<ci> mw3885b0e0_19bc_4407_a41c_94c1f48987d7 </ci>

</apply>

</apply>

</math>

</kineticLaw>

</reaction>

<reaction id="mwbf4d2b01_bff8_47d7_a5b4_205a375f7e38" name="Sink PEP" reversible="false" fast="false">

<annotation>

<COPASI xmlns="http://www.copasi.org/static/sbml">

<rdf:RDF xmlns:dcterms="http://purl.org/dc/terms/" xmlns:rdf="http://www.w3.org/1999/02/22-rdf-syntax-ns#">

<rdf:Description rdf:about="#COPASI79">

<dcterms:created>

<rdf:Description>

<dcterms:W3CDTF>2011-05-10T16:28:58Z</dcterms:W3CDTF>

</rdf:Description>

</dcterms:created>

</rdf:Description>

</rdf:RDF>

</COPASI>

</annotation>

<listOfReactants>

<speciesReference species="mw6951dd88_19cf_4045_95c4_0e8e2bf976c1"/>

</listOfReactants>

```
<listOfProducts>
  <speciesReference species="mwb7dd85d2_03b5_4642_acac_52cc771e3966"
stoichiometry="3"/>
</listOfProducts>
<kineticLaw>
  <math xmlns="http://www.w3.org/1998/Math/MathML">
    <apply>
      <divide/>
      <apply>
        <times/>
        <ci> mwbba6a282_e627_49c3_9f45_889c3993c575 </ci>
        <ci> mw6951dd88_19cf_4045_95c4_0e8e2bf976c1 </ci>
      </apply>
      <apply>
        <plus/>
        <ci> mw10c7c233_9065_48c8_8a5c_8a27f4ee15d6 </ci>
        <ci> mw6951dd88_19cf_4045_95c4_0e8e2bf976c1 </ci>
      </apply>
    </apply>
  </math>
</kineticLaw>
</reaction>
<reaction id="mw3c08c030_dd55_4443_8795_e07b05464e0b" name="Sink Pyruvate"
reversible="false" fast="false">
<listOfReactants>
  <speciesReference species="mw3c3b7565_7ecb_4d83_a206_818265293cb6"/>
</listOfReactants>
<listOfProducts>
  <speciesReference species="mwb7dd85d2_03b5_4642_acac_52cc771e3966"
stoichiometry="3"/>
</listOfProducts>
<kineticLaw>
```

```
          <math xmlns="http://www.w3.org/1998/Math/MathML">
            <apply>
              <divide/>
              <apply>
                <times/>
                <ci> mwb2bc9da8_6f54_4986_b142_b256c7b7bc82 </ci>
                <ci> mw3c3b7565_7ecb_4d83_a206_818265293cb6 </ci>
              </apply>
              <apply>
                <plus/>
                <ci> mwb7105ee5_87bd_444a_aa8c_74b7fadd678a </ci>
                <ci> mw3c3b7565_7ecb_4d83_a206_818265293cb6 </ci>
              </apply>
            </apply>
          </math>
        </kineticLaw>
      </reaction>
      <reaction id="mw6325f7cf_c3ea_4306_b124_255f236cfab7" name="Sink GLY" reversible="false"
fast="false">
        <listOfReactants>
          <speciesReference species="mw3d986236_5c95_4128_9ee4_95d616f64d6f"/>
        </listOfReactants>
        <listOfProducts>
          <speciesReference species="mw99ae2c3c_4850_462a_8eb9_29ce642d9a9c"/>
        </listOfProducts>
        <kineticLaw>
          <math xmlns="http://www.w3.org/1998/Math/MathML">
            <apply>
              <divide/>
              <apply>
                <times/>
```

<ci> mw3c3bc745_1bf5_4845_98a6_dbaa2aedd575 </ci>

<ci> mw3d986236_5c95_4128_9ee4_95d616f64d6f </ci>

</apply>

<apply>

<plus/>

<ci> mw84076d99_c4a9_4fcf_b194_46b4503635be </ci>

<ci> mw3d986236_5c95_4128_9ee4_95d616f64d6f </ci>

</apply>

</apply>

</math>

</kineticLaw>

</reaction>

<reaction id="mwf9ec828a_6ddf_4379_a61e_90283a4489ed" name="Sink SER" reversible="false" fast="false">

<listOfReactants>

<speciesReference species="mw7bb27e61_6440_4c3b_af0a_6c1ecd36ff29"/>

</listOfReactants>

<listOfProducts>

<speciesReference species="mw99ae2c3c_4850_462a_8eb9_29ce642d9a9c"/>

</listOfProducts>

<kineticLaw>

<math xmlns="http://www.w3.org/1998/Math/MathML">

<apply>

<divide/>

<apply>

<times/>

<ci> mw1a0030a7_698d_4add_adca_187e18575335 </ci>

<ci> mw7bb27e61_6440_4c3b_af0a_6c1ecd36ff29 </ci>

</apply>

<apply>

<plus/>

```
            <ci> mw7b8f321d_ca9c_4a2b_b7e7_44d5fca51f8d </ci>
            <ci> mw7bb27e61_6440_4c3b_af0a_6c1ecd36ff29 </ci>
          </apply>
        </apply>
      </math>
    </kineticLaw>
  </reaction>
  <reaction id="mw6bc808b2_8721_456e_a62b_9e0d81305585" name="Sink G6P"
reversible="false" fast="false">
    <listOfReactants>
      <speciesReference species="mw3a6830cd_1b04_49df_b4fe_fc7a6362142a"/>
    </listOfReactants>
    <listOfProducts>
      <speciesReference species="mw2e29aa28_0bc5_4b38_88a0_c885fd689c98"
stoichiometry="6"/>
    </listOfProducts>
    <kineticLaw>
      <math xmlns="http://www.w3.org/1998/Math/MathML">
        <apply>
          <divide/>
          <apply>
            <times/>
            <ci> mw6b34d5e4_8388_4d61_837c_26f3c9c09838 </ci>
            <ci> mw3a6830cd_1b04_49df_b4fe_fc7a6362142a </ci>
          </apply>
          <apply>
            <plus/>
            <ci> mwd7d873d8_453a_431d_a268_fff0b2981096 </ci>
            <ci> mw3a6830cd_1b04_49df_b4fe_fc7a6362142a </ci>
          </apply>
        </apply>
      </math>
```

```
        </kineticLaw>
      </reaction>
      <reaction id="mwab15ae0b_0fef_449f_b169_1d461dd7d888" name="Sink AceP"
reversible="false" fast="false">
        <listOfReactants>
          <speciesReference species="mw543ef166_8322_44f0_9435_270a9f9672da"/>
        </listOfReactants>
        <listOfProducts>
          <speciesReference species="mw92f0b5a5_287d_4b4e_8e20_756a2c2a2fd7"
stoichiometry="2"/>
        </listOfProducts>
        <kineticLaw>
          <math xmlns="http://www.w3.org/1998/Math/MathML">
            <apply>
              <divide/>
              <apply>
                <times/>
                <ci> mw72813c68_f9ad_4693_a385_08d49dcb3b9c </ci>
                <ci> mw543ef166_8322_44f0_9435_270a9f9672da </ci>
              </apply>
              <apply>
                <plus/>
                <ci> mwe2e6283d_1779_48e0_93fd_a0e7744d0f49 </ci>
                <ci> mw543ef166_8322_44f0_9435_270a9f9672da </ci>
              </apply>
            </apply>
          </math>
        </kineticLaw>
      </reaction>
      <reaction id="mw24ad13f9_fe17_4762_8dc4_ef19295e13f9" name="Mixotrophic growth - G6P
as a source" reversible="false" fast="false">
        <listOfReactants>
```

```
          <speciesReference species="mw19a9030f_8af8_485a_ba7c_5d89c93164cc"/>
        </listOfReactants>
        <listOfProducts>
          <speciesReference species="mw3a6830cd_1b04_49df_b4fe_fc7a6362142a"/>
        </listOfProducts>
        <kineticLaw>
          <math xmlns="http://www.w3.org/1998/Math/MathML">
            <apply>
              <times/>
              <ci> mwbc1c0f08_cf5f_4e06_a14b_d5c559bb147c </ci>
              <ci> mw19a9030f_8af8_485a_ba7c_5d89c93164cc </ci>
            </apply>
          </math>
        </kineticLaw>
      </reaction>
      <reaction id="mw95cd53b0_c815_4e27_b97f_4d1a4462695d" name="ED-P edd"
reversible="false" fast="false">
        <listOfReactants>
          <speciesReference species="mwb8027448_5c51_43ad_9f13_cd37b5418a54"/>
        </listOfReactants>
        <listOfProducts>
          <speciesReference species="mwf3e38952_b929_46f3_b15d_3e4a170b3863"/>
        </listOfProducts>
        <kineticLaw>
          <math xmlns="http://www.w3.org/1998/Math/MathML">
            <apply>
              <divide/>
              <apply>
                <divide/>
                <apply>
                  <times/>
```

<ci> mw518ffec0_9f71_433e_9dc8_9ca216769de1 </ci>
<ci> mwb8027448_5c51_43ad_9f13_cd37b5418a54 </ci>
</apply>
<apply>
<plus/>
<ci> mwb8027448_5c51_43ad_9f13_cd37b5418a54 </ci>
<ci> mw5ceb33ad_a5ce_4d67_8217_5ac758441102 </ci>
</apply>
</apply>
<apply>
<plus/>
<cn type="integer"> 1 </cn>
<apply>
<divide/>
<ci> mwf3e38952_b929_46f3_b15d_3e4a170b3863 </ci>
<ci> mw7f5b93ed_2736_43c7_9104_bc8302ad8c01 </ci>
</apply>
</apply>
</apply>
</math>
</kineticLaw>
</reaction>
<reaction id="mw86550d58_cdac_45b0_b3d9_8dc13a5d7a10" name="ED-P eda" reversible="false" fast="false">
<listOfReactants>
<speciesReference species="mwf3e38952_b929_46f3_b15d_3e4a170b3863"/>
</listOfReactants>
<listOfProducts>
<speciesReference species="mw5b528d37_b514_46e5_a018_0173afdae714"/>
<speciesReference species="mw3c3b7565_7ecb_4d83_a206_818265293cb6"/>
</listOfProducts>

```
        <kineticLaw>
          <math xmlns="http://www.w3.org/1998/Math/MathML">
            <apply>
              <divide/>
              <apply>
                <times/>
                <ci> mwf79253b7_efe2_42f5_bacb_32ceeaccf8cc </ci>
                <ci> mwf3e38952_b929_46f3_b15d_3e4a170b3863 </ci>
              </apply>
              <apply>
                <plus/>
                <ci> mw9674d404_dd5e_47e7_a284_60e128055d71 </ci>
                <ci> mwf3e38952_b929_46f3_b15d_3e4a170b3863 </ci>
              </apply>
            </apply>
          </math>
        </kineticLaw>
      </reaction>
    </listOfReactions>
  </model>
</sbml>
```
